# Transcriptome profiling of genes and pathways associated with arsenic toxicity and tolerance in *Arabidopsis*

**DOI:** 10.1186/1471-2229-14-94

**Published:** 2014-04-16

**Authors:** Shih-Feng Fu, Po-Yu Chen, Quynh Thi Thuy Nguyen, Li-Yao Huang, Guan-Ru Zeng, Tsai-Lien Huang, Chung-Yi Lin, Hao-Jen Huang

**Affiliations:** 1 Department of Biology, National Chunghua University of Education, No.1, Jin-De Road, 500, Changhua, Taiwan; 2 Department of Life Sciences, National Cheng Kung University, No.1 University Road 701, Tainan, Taiwan

**Keywords:** Arsenate, *Arabidopsis* accession, Microarray

## Abstract

**Background:**

Arsenic (As) is a toxic metalloid found ubiquitously in the environment and widely considered an acute poison and carcinogen. However, the molecular mechanisms of the plant response to As and ensuing tolerance have not been extensively characterized. Here, we report on transcriptional changes with As treatment in two *Arabidopsis* accessions, Col-0 and Ws-2.

**Results:**

The root elongation rate was greater for Col-0 than Ws-2 with As exposure. Accumulation of As was lower in the more tolerant accession Col-0 than in Ws-2. We compared the effect of As exposure on genome-wide gene expression in the two accessions by comparative microarray assay. The genes related to heat response and oxidative stresses were common to both accessions, which indicates conserved As stress-associated responses for the two accessions. Most of the specific response genes encoded heat shock proteins, heat shock factors, ubiquitin and aquaporin transporters. Genes coding for ethylene-signalling components were enriched in As-tolerant Col-0 with As exposure. A tolerance-associated gene candidate encoding Leucine-Rich Repeat receptor-like kinase VIII (LRR-RLK VIII) was selected for functional characterization. Genetic loss-of-function analysis of the *LRR-RLK VIII* gene revealed altered As sensitivity and the metal accumulation in roots.

**Conclusions:**

Thus, ethylene-related pathways, maintenance of protein structure and LRR-RLK VIII-mediated signalling may be important mechanisms for toxicity and tolerance to As in the species. Here, we provide a comprehensive survey of global transcriptional regulation for As and identify stress- and tolerance-associated genes responding to As.

## Background

Arsenic (As) is a ubiquitously present non-essential metalloid of serious environmental concern because of ever-increasing contamination [1,2]. Naturally high levels of As in drinking water have caused major human health problems in the United States, Argentina, Taiwan, and most notably Bangladesh and India, where tens of millions of people have been affected [3,4]. As finds its way into the food chain through irrigation with contaminated groundwater [5]. Uptake of As in plant tissues adversely affects the plant metabolism and leads to a significant reduction in crop yield [6-8]. Understanding of As-induced stress and ensuing tolerance would be beneficial for the development of As-resistance crops and other economically important plants [8].

Roots are involved in mineral acquisition by plants, and function at the interface with the rhizosphere. Alterations of root architecture and inhibition of root elongation are considered primary symptoms of As-toxicity [9,10]. In many circumstances, it is the As-sensitivity of the root that limits the productivity of the entire plant [11]. Hence, plants exposed to As show inhibited root growth and reduced photosynthetic rate [8,12]. While plant roots are the first organs in contact with As, assaying the processes occurring in the roots could provide potential strategies to determine how plants respond and adapt to the heavy metal stress.

As is present both as As (III) and As (V) in the environment, with As (V) being more prevalent than As (III) in soils [8,13]. The mechanism by which As is taken up by plants differs for As(III) and As(V) [14]. As (III) uptake is thought to occur through the aquaporins of roots [14,15]. Higher plants take up As (V) as the dominant form of phytoavailable As in aerobic soils. As (V) is a phosphate analogue and easily incorporated into plant cells through the high-affinity phosphate transport system [8,14]. As (V) replaces phosphate in ATP to form unstable “ADP-As” complexes and disrupts energy flow in cells [8]. As (III) reacts with the sulfhydryl groups of enzymes and proteins, thereby inhibiting cellular function and resulting in cell death [8,16]. In addition, As stimulates the formation of free radicals and reactive oxygen species, thus resulting in oxidative stress [9,10,17].

Detoxification and tolerance mechanisms often involve extrusion of the toxic ions from cells, sequestration within internal organelles, reduced toxin uptake, and chelation by metal-binding proteins such as phytochelatins (PCs) [18]. These mechanisms reduce the cellular content of the toxic agent, although the molecular basis may differ among metals and organisms [19-21]. Differences in As sensitivities exist among plant accessions or varieties [11,22,23]. Understanding the mechanisms underlying reduced As sensitivity and genes responsible is important in directing future breeding to counter As stress. The potential of As tolerance, based on such a concerted response of the various pathways, would also depend on an early perception of As-induced stress [10]. The precise mechanisms of perception of As-induced stress in plants remain to be elucidated.

Reducing As intake requires identifying the mechanisms implicated in As toxicity and tolerance in plants. A substantial number of genes are differentially regulated by As stress in various plant species [9,10,24]. However, genes identified as As responsive are in part related to a general stress response resulting from the toxic effects of As and are unlikely to play a significant role in As tolerance. High-throughput gene expression profiles with microarray technology and their application in comparative studies help to reveal the roles of differential gene regulation in As toxicity and tolerance. For more insight into the molecular basis of As toxicity and tolerance responses in plants, we performed transcriptional profiling of 2 contrasting accessions of *Arabidopsis thaliana*, Col-0 and Ws-2. We focused on genes that are commonly and specifically regulated by the two accessions and discuss the putative functions of identified genes in several biochemical pathways in terms of As toxicity and tolerance. New genes identified may provide more clues to understanding the molecular mechanism of response to As-induced stress in plants.

## Results

### Effects of As stress on root elongation in Arabidopsis accessions Col-0 and Ws-2

**Figure 1:**
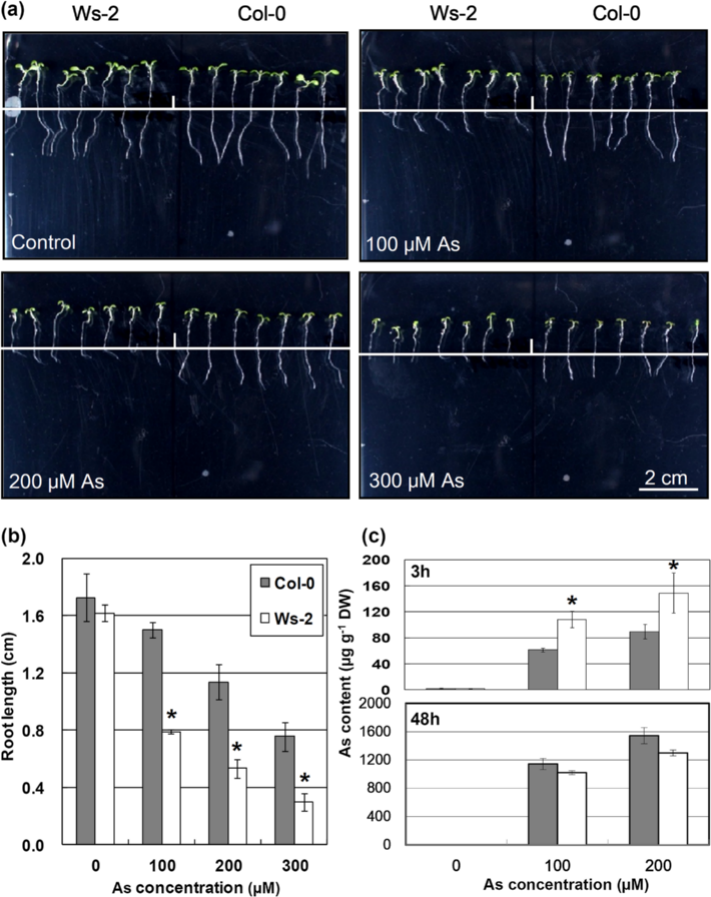
**Effect of As (V) treatment on the root elongation and metal accumulation of *Arabidopsis thaliana *accessions Col-0 and Ws-2. (a)** Seedlings with different accession backgrounds (Col-0 and Ws-2) were grown on quarter-strength MS medium for 4 d and then transferred to medium with 100, 200 and 300 μM As (V) and grown for an additional 2 d (Scale bar, 2 cm ). As tolerance was determined by relative root growth after treatment. **(b)** Root length of plants was measured after treatment with As. Root samples were collected from 3 independent experiments (each from a pool of 7 root samples). Data are mean±SD. *P ≤ 0.05 compared with Col-0 in each concentration of As. The difference in root elongation is significant according to Student's t test. **(c)** Accumulation of As (V) by *Arabidopsis* accessions Col-0 and Ws-2 was analyzed with ICP-AES. The 4-d-old seedlings with different accession backgrounds (Col-0 and Ws-2) were transferred to medium containing 100 and 200 μM As (V) for 3h and 48 h, then root tips were collected and measured. Data are mean±SD calculated from 3 biological replicates per treatment. *P ≤ 0.05 compared with Col-0.

Root growth inhibition is the primary response of the plant exposed to heavy metals. We analyzed the effect of As (V) exposure on primary root elongation to evaluate As toxicity and tolerance in *A. thaliana* accessions, including Col-0, Ws-2 and Ler-0. As a result, we examined accessions for As tolerance and exposed 1 tolerant (Col-0) and 1 sensitive (Ws-2) accession to As for 2 d for measuring root elongation (Figure 1a). At 100 μM, As significantly reduced root elongation in both *Arabidopsis* accessions as compared with normal conditions (Figure 1a). Of note, Col-0 showed less reduction in root elongation (15%) than Ws-2 (50%). The root elongation decreased with increasing As concentration (Figure 1b). At 200 μM As, the reduction in root elongation in Col-0 was 40% as compared with 60% in Ws-2. At 300 μM As, root growth was minimal in Col-0 and completely inhibited in Ws-2. As at 200 and 100 μM inhibited root elongation by approximately one-half in Col-0 and Ws-2, respectively. Col-0 was more tolerant to As than Ws-2. Therefore, we hereafter refer to Col-0 as As tolerant and Ws-2 as As sensitive.

### Accumulation of As concentration in Arabidopsis roots with As exposure

To investigate whether the differences in As tolerance in the two accessions was associated with As concentration in roots, we exposed root tips to As concentrations for 3 h and 48 h (Figure 1c). When exposed to As for 3 h, As was increased in roots of both accessions with increasing As concentration. The concentration of As was lower in the As-tolerant than As-sensitive accession (Figure 1c). Note that at 48 h, concentration of As was accumulated to a similar level in As-sensitive and As-tolerant accession, which possibly resulted from a more severely inhibited transpiration rate. Taken together, As accumulation in the roots of both accessions was associated with As concentration, with greater accumulation in Ws-2 than Col-0. The data may explain the differences in As toxicity and tolerance between the two accessions.

### Global expression profiles of Col-0 and Ws-2 in response to As

To identify genes associated with As toxicity and tolerance in *Arabidopsis*, we used large-scale expression profiling. The As exposure data (Figure 1) helped determine a suitable As concentration for microarray analysis. Treatment of Col-0 and Ws-2 with As at 200 and 100 μM, respectively, had similar effects on root growth inhibition, indicating equal toxic strength. RNA samples were collected from root tips early (1.5 to 3 h) after As treatment to examine rapid changes in global patterns of gene expression. We pooled RNA isolated from with 1.5- and 3-h As treatment to maximize gene discovery.

**Figure 2:**
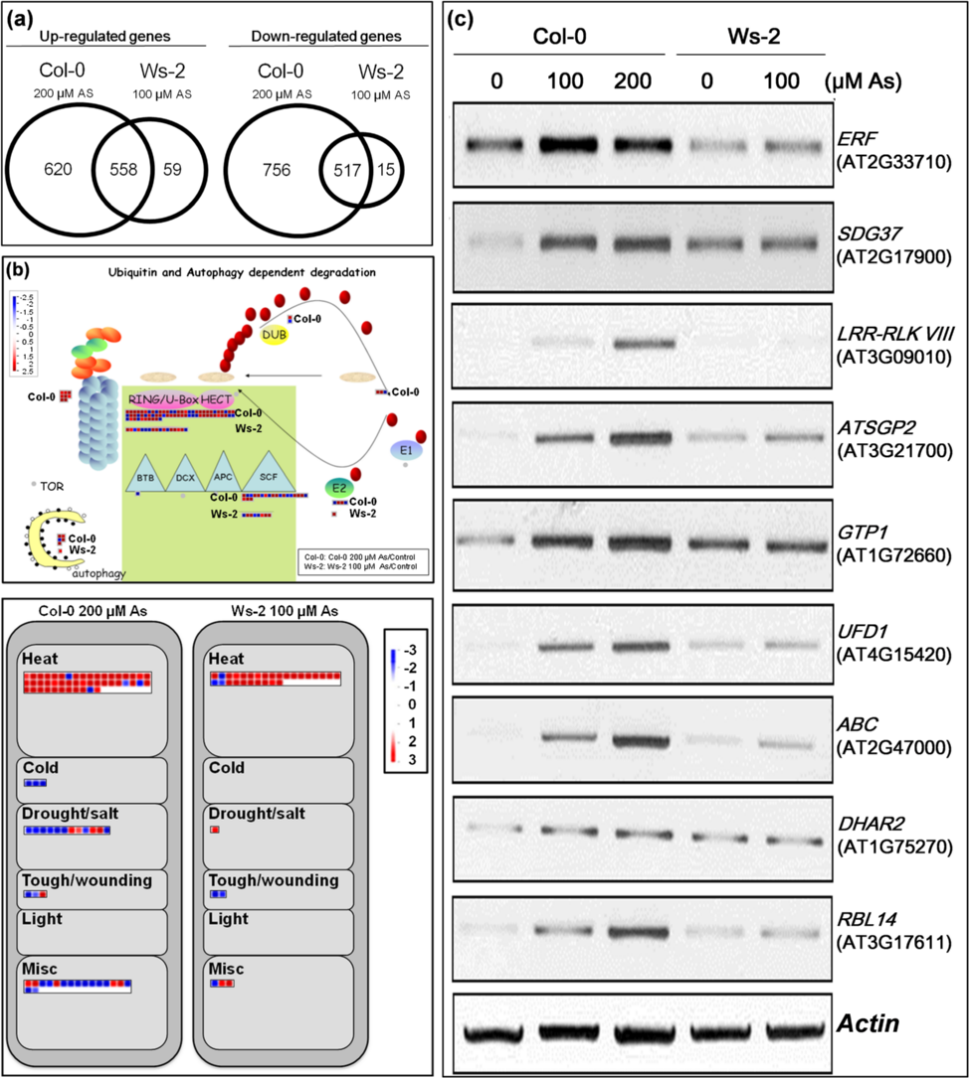
**Microarray expression of genes in *Arabidopsis *Col-0 and Ws-2 plants exposed to As stress. (a)** Venn diagram of regulated As-responsive genes extracted by comparing microarray probe sets of the 2 *Arabidopsis* accessions. The number of overlapping and non-overlapping genes early (1.5 to 3 h) after treatment with 100 or 200 μM As is shown. The probe sets were selected on the basis of an adjusted P-value of <0.05 and a >2-fold change in gene expression. The area of the diagram is proportional to the number of genes that are up- or downregulated in response to As stress. To clearly differentiate As-regulated genes in Col-0 from that in Ws-2, the Col-0-specific set contains only those genes with both >2-fold change in abundance (compared with control treatment) in Col-0 and <1.4-fold change in Ws-2. Likewise, the Ws-2-specific set contains genes with both > 2-fold change in abundance in Ws-2 and < 1.4-fold change in Col-0. The general As-regulated gene set contains genes displaying > 2-fold change in abundance in Col-0 or Ws-2 and >1.4-fold change in another accession. **(b)** Displayed are genes associated with ubiquitin pathways and abiotic stress responses using MapMan software. Both sets of material were harvested from roots tissues treated with or without As stress (100 and 200 μM for Ws-2 and Col-0, respectively). Red and blue signals represent a decrease and increase in transcript abundance, respectively, relative to water-treated control samples. The scale used for coloration of signals (log_2_ ratios) is presented. **(c)** Validation of representative As-induced genes by semi-quantitative RT-PCR analysis. Total RNA was extracted from root tissues of *Arabidopsis* plants with different accessions with As treatment. The As-treated roots were harvested at 1 and 3 h. The samples were pooled together. The transcript level of actin served as an equal loading control.

Fold-change values were compared with a control sample without As treatment. Differentially expressed genes were defined as those with at least 2-fold change in transcript abundance and with an adjusted P value less than 0.05 [25]. In general, the As-tolerant and As-sensitive accessions showed similar expression patterns in the control conditions. Therefore, constitutive gene expression was similar between the two accessions before As treatment. In total, 620 probe sets were specifically upregulated in As-tolerant Col-0 plants exposed to 200 μM As (Col-0 200 μM As versus Col-0 control) and 756 were downregulated (Figure 2a). These 1376 As-regulated genes were unique to Col-0. In Ws-2, 59 probes were upregulated in response to 100 μM As (Ws-2 100 μM As versus Ws-2 control) and 15 were downregulated. These 74 As-regulated genes were unique to Ws-2. A total of 558 probes were significantly upregulated in both Col-0 and Ws-2 and 517 were downregulated in both (Additional file 1: Table S1 for full list). Thus, a relatively larger number of genes showed changed expression with As in Col-0, which suggests differential cellular response with early As exposure.

### GO analysis of As-responsive genes

We subjected genes up- and downregulated with As stress to GO analysis using the EasyGO program to understand the metabolic and regulatory differences between the two accessions on As exposure. The major GO terms for biological processes for As-responsive genes are summarized (Table 1). Upregulated genes for both accessions were in functional categories corresponding to responses to heat, oxidative stress, and metal ion and carbohydrate stimulus. Col-0-specific genes were involved in responses to ethylene stimulus and abscisic acid (ABA) and heat acclimation. Ws-2-specific genes were involved in osmotic stress and toxin response. Downregulated genes in both accessions were predominately involved in cell wall organization, response to cytokinin, glycoside biosynthesis and transport (Table 1). Col-0-specific downregulated genes were related to receptor protein signaling pathway with As stress.

**Table 1:** **Gene ontology categories corresponding to genes differentially regulated by As in *Arabidopsis *** **accessions exposed to As**

**Biological process**	**Accession**^**a**^	**No. genes**^**a**^	**Background**	**False discovery rates**
**Upregulated generally by As (V)**				
Heat response	Col-0, Ws-2	49, 32	114	1.70E-27, 1.95E-25
Oxidative stress	Col-0, Ws-2	42, 29	258	1.06E-9, 2.87E-13
Chitin response	Col-0, Ws-2	41, 11	127	7.05E-19, 1,39E-5
Metal ion response	Col-0, Ws-2	44, 17	367	2.58E-06, 0.00799
Carbohydrate stimulus	Col-0, Ws-2	50, 14	203	5.80E-21, 0.000626
**Upregulated specifically by As (V)**				
Ethylene response	Col-0 (Ws-2)	16, ( 5 )	143	5.01E-05
ABA response	Col-0 (Ws-2)	14, ( 6 )	262	0.00773
Heat acclimation	Col-0 (Ws-2)	5 ( 2 )	15	0.00154
Osmotic stress	Ws-2 (Col-0)	16 ( 7 )	391	0.0346
Toxin response	Ws-2 (Col-0)	16 ( 10 )	66	5.54E-12
**Downregulated generally by As (V)**				
Cell wall organization	Col-0, Ws-2	49, 12	263	3.01E-12, 3.81E-05
Secondary metabolic process	Col-0, Ws-2	47, 18	359	2.50E-07, 9.42E-06
Cytokinin response	Col-0, Ws-2	8, 6	75	0.0196, 0.0066
Glycoside biosynthesis	Col-0, Ws-2	12,11	32	8.31E-06, 4.30E-11
Transport	Col-0, Ws-2	73,33	1732	0.0499, 0.0289
**Downregulated specifically by As (V)**				
Receptor protein signaling	Col-0	30	130	1,96E-09

^**a**^Accession specificity were identified according to false discovery rates (FDR < 0.05) of gene ontology analysis. Accession and no. of genes in parentheses refers to FDR > 0.05 in each gene ontology term.

To expand the functional significance of the As-responsive genes in Col-0 and Ws-2, we used MapMan representations to highlight microarray data and biochemical pathways. Col-0-specific genes with changed expression were involved in the proteasome and heat stress (Figure 2b). The sulfur assimilation pathway, which leads to phytochelatin biosynthesis, is affected by As stress in both accessions (Additional file 2: Figure S1). The gene encoding phytochelatin synthase was upregulated only in Col-0 plants after exposure to As stress.

### Oxidative stress-related genes regulated by As exposure

The As-responsive genes can be grouped into various biological processes such as oxidative stress, transporter, hormone homeostasis and signal transduction. First, the expression levels of the genes dealing with the responses to oxidative stress were compared between the two accessions (Additional file 1: Tables S2 and S3). Expression of genes coding for alternative oxidase, dehydroascorbate reductase, glutaredoxin, peroxidase were regulated in both accessions on exposure to As stress (Additional file 1: Table S2). In addition, a number of genes encoding thioredoxin were mostly induced in tolerant Col-0. Genes encoding glutathione S-transferases (GSTs) were predominately upregulated by As in both accessions. A total of 14 genes encoding for GST were upregulated in As-sensitive Ws-2, and 9 GST genes were induced in As-tolerant Col-0. Most of the As-responsive GST genes belong to the 40-member Tau class GSTs [26]. Fewer GST-related genes were upregulated in As-tolerant Col-0 than in As-sensitive Ws-2.

### Transporter genes regulated by As exposure

A significant number of genes encoding transporters were differentially expressed in the As-sensitive and As-tolerant accessions with As exposure (Additional file 1: Table S4). ATP-binding cassette (ABC) transporters comprised the largest group of transporter-related genes (Additional file 1: Table S2). A total of 9 and 14 ABC transporter genes were differentially regulated in the Ws-2 and Col-0 accessions, respectively. Transcripts for genes from the multidrug and toxic compound extrusion (MATE) transporters and antiporters were also regulated by As treatment. Genes annotated as aquaporin, LeOPT1 oligopeptide transporters (OPT) and sugar transporter were predominately downregulated in Col-0 with As exposure, which suggests the differential regulation of transporter-related genes between the two accessions in response to As stress.

### Hormonal genes regulated by As exposure

To understand the expression pattern of genes involved in hormone pathways between the As-sensitive and As-tolerant accessions, we analyzed transcripts related to hormone metabolism and found a potential role of ABA, brassinosteroid, cytokinin and ethylene in an early response to As stress (Additional file 1: Table S5). Genes encoding proteins involved in cytokinin homeostasis were mostly downregulated by As stress in both accessions (Additional file 1: Table S2). ABA-related genes were regulated (expression level 2 ~ 5 fold change) predominately in As-tolerant Col-0. In total, 14 and 5 ethylene-related genes (expression level 2 ~ 26 fold change) were regulated in the Col-0 and Ws-2 accessions, respectively. Ethylene biosynthesis- and signaling-related genes were regulated by As stress, especially in As-tolerant Col-0.

### Transcription factors and protein kinases regulated by As exposure

In total, 200 and 69 genes encoding transcription factors were differentially expressed with As exposure in As-tolerant Col-0 and As-sensitive Ws-2, respectively (Additional file 1: Table S6). Col-0 showed more genes induced: *APETALA2*/*ethylene-responsive-element-binding protein* (*AP2*/*EREBP*), *Aux*/*IAA*, *heat shock transcription factor* (*HSF*), and *WRKY* (Additional file 1: Table S2). Genes encoding bHLH, C2H2, GARP-G2-like, and MYB were predominately downregulated in Col-0. Further, members of the AP2/EREBP, HSF and WRKY families were upregulated specifically in Col-0 with As stress. Kinases may act as signals on the transcription factors, leading to the production of stress proteins and secondary metabolites that can act as either damage-causing or stress-countering agents. Genes involved in mitogen-activated protein kinase (MAPK) and SNF1-related kinases (SnRKs) and leucine-rich repeat receptor-like kinase VIII (LRR-RLK VIII) pathways were more regulated in Col-0 than in Ws-2 by As stress (Additional file 1: Tables S2 and S7).

### Identification of putative candidate genes for As tolerance in Arabidopsis

We aimed to isolate the *Arabidopsis* genes responsible for As tolerance. The microarray data corresponding to the 2 contrasting accessions and the dose–response effect were integrated to identify tolerance-associated genes. Accordingly, gene-filtering criteria were based on 2-fold change in gene expression in As-treated Col-0 versus Ws-2 (Col-0 200 μM As/Col-0 Control versus Ws-2 100 μM As/Ws-2 Control). Besides, a minimum 2-fold change in the dose–response effect in Col-0 (200 μM As/Control versus 100 μM As/Control) was included in the filtering criteria. We found 63 putative genes grouped according to biological processes by GO analysis (Table 2). These genes were further classified into regulatory genes, such as those encoding proteins responsible for signal transduction, transcriptional regulation, GTP binding and the proteasome-related pathway, and functional genes contributing to responses to heat, mitochondria electron transport and responses to oxidative, salt and other stresses (Table 2). We found 12 putative regulatory genes encoding the proteasome-related pathway associated with As tolerance (Table 2). These genes belong to various types of proteasome-related components such as *RING/U-box superfamily protein* (AT1G14200), *RING-H2 finger protein RHA1a* (AT4G11370), *ubiquitin fusion degradation UFD1 family protein* (AT4G1542) and *membrane-anchored ubiquitin-fold protein 4* (AT3G26980). In addition, tolerance to As was associated with the increased expression of genes related to heat stress, such as the *heat-shock-protein 20-like chaperone superfamily protein* (AT1G52560) and *HSF B2A* (AT5G62020).

**Table 2:** Selection of putative As tolerance-associated genes by comparing the expression ratio between the two accessions

**AGI number**	**Product encoded**	**Tolerance ratio**^**a**^	**Dosage ratio**^**b**^
		**Col-0 200/Ws-100**	**Col-0 200/100 (μM)**
***Transcription factor***			
AT3G17611	RHOMBOID-like protein 14 (RBL14)	2.84	2.12
AT1G76880	GT-like trihelix DNA-binding protein	6.41	2.11
AT2G17900	ATSDG37	3.39	2.50
AT5G62020	Heat shock transcription factor B2A	4.49	3.41
AT2G33710	ERF (ethylene response factor) transcription factor family	3.98	2.15
***Responses to heat***			
AT5G59720	ATHSP18.2	30.66	2.18
AT1G52560	HSP20-like chaperones superfamily protein	18.23	3.16
AT5G37670	HSP20-like chaperones superfamily protein	12.70	3.06
AT4G10250	ATHSP22	8.58	3.84
AT5G15450	HSP100/ClpB	8.12	2.42
AT2G32120	ATHSP70-2	4.51	2.89
AT4G25200	AtHSP23.6	4.33	3.46
AT4G21320	Heat-stress-associated 32-kD protein	4.12	2.18
AT2G26150	Heat shock transcription factor A2	3.79	3.98
AT2G25140	HSP100/ClpB4	3.35	3.38
AT2G19310	HSP20-like chaperones superfamily protein	4.14	2.10
AT3G12050	Aha1 domain-containing protein	2.77	2.25
AT3G09350	Encodes one of the *Arabidopsis* Hsp70-binding protein 1	4.12	2.39
AT2G24860	DnaJ/Hsp40 cysteine-rich domain superfamily protein	6.39	2.12
***Responses to oxidative, salt and other stresses***			
AT4G37370	CYP81D8, cytochrome P450 - like protein	13.24	2.81
AT2G34500	CYP710A1, putative cytochrome P450	5.73	2.66
AT1G73480	alpha/beta-Hydrolases superfamily protein	6.47	2.33
AT3G60140	DIN2, member of glycoside hydrolase family 1	5.49	2.22
AT5G37710	alpha/beta-Hydrolases superfamily protein	3.33	2.25
AT3G09640	ATAPX2	5.72	5.17
AT2G04040	AtDTX1, identified as a detoxifying efflux carrier	4.92	3.00
AT2G47710	Adenine nucleotide alpha hydrolases-like superfamily	2.35	2.09
***Signal transduction***			
AT4G21390	Protein serine/threonine kinase activity	17.03	2.00
AT3G09010	Protein kinase superfamily protein (LRR-RLK VIII)	3.23	2.58
AT3G22840	Encodes an early light-inducible protein	2.38	2.05
AT2G21940	Encodes a shikimate kinase	3.62	2.37
AT2G20900	diacylglycerol kinase 5 (DGK5)	3.67	2.17
***Proteasome related pathway***			
AT1G14200	RING/U-box superfamily protein	6.60	2.89
AT1G26800	RING/U-box superfamily protein	6.22	2.65
AT1G55530	RING/U-box superfamily protein	5.24	2.45
AT5G09800	ARM repeat superfamily protein	5.56	2.39
AT5G48655	RING/U-box superfamily protein	3.95	2.13
AT3G13430	RING/U-box superfamily protein	3.75	2.28
AT4G11370	Encodes a putative RING-H2 finger protein RHA1a	3.12	2.15
AT5G38895	RING/U-box superfamily protein	2.83	2.29
AT4G23570	AtSGT1a	3.56	2.49
AT4G15420	Ubiquitin fusion degradation UFD1 family protein	3.43	2.00
AT1G32940	ATSBT3.5, involved in proteolysis	3.65	2.05
AT3G26980	MUB4, membrane-anchored ubiquitin-fold protein 4	4.88	2.54
***GTP binding***			
AT3G21700	ATSGP2, Monomeric G protein	4.93	2.28
AT1G72660	GTP1, Small GTP-binding protein	16.12	3.01
***Mitochondria electron transport***			
AT5G25450	Cytochrome bd ubiquinol oxidase	3.91	2.07
AT4G28390	AAC3, ADP,ATP carrier-like protein	10.16	2.57
AT4G27940	manganese tracking factor for mitochondrial SOD2 (MTM1)	2.65	2.70
***Others***			
AT2G03430	Ankyrin repeat family protein	2.03	2.37
AT3G25900	ATHMT-1, Homocysteine S-methyltransferase	7.68	2.04
AT2G15490	ATUGT7, putative glucosyltransferase	4.86	2.07
AT4G00550	UDP-galactose-dependent digalactosyldiacylglycerol synthase	6.22	3.29
AT4G26270	phosphofructokinase 3 (ATPFK3)	5.38	3.64
AT3G16050	Encodes a protein with pyridoxal phosphate synthase	3.64	2.14
AT1G30070	SGS domain-containing protein	3.53	2.26
AT5G64170	dentin sialophosphoprotein-related protein	3.29	2.34
AT3G57810	Cysteine proteinases superfamily protein	2.94	2.27
AT1G24090	RNase H family protein	2.90	2.24
AT2G16900	*Arabidopsis* phospholipase-like protein family	2.80	2.08
AT4G32440	Plant Tudor-like RNA-binding protein	2.40	2.28
AT1G67360	Rubber elongation factor protein	2.24	2.73

^**a**^Col-0 200/Ws-100 refers to pair-wise comparison of expression ratio (Col-0 200 μM As vs Col-0 Control) / (Ws-2 200 μM As vs Ws-2 Control).

^**b**^Col-0 200/100 refers to pair-wise comparison of expression ratio (Col-0 200 μM As vs Col-0 Control) / (Col-0 100 μM As vs Col-0 Control).

A set of the tolerance-associated genes such as *HSF 2A* (AT2G26150), *HSF B2A* (AT5G62020), *ethylene response factor* (AT2G33710), *GT-like trihelix DNA-binding protein* (AT1G76880), *ATSDG37* (AT2G17900) and *LRR-RLK VIII* (AT3G09010) were selected for validation by semi-quantitative reverse-transcription polymerase chain reaction (RT-PCR). The expression level of these genes was increased with As concentration in Col-0 (Figure 2c). In addition, the induction of the genes by As treatment was higher in Col-0 than in Ws-2. The regulation of these genes in response to As stress demonstrated the does-response and accession-specific effects. These results were consistent with the microarray data.

### Transcriptional characteristics of putative As-tolerance associated genes encoding LRR-RLK VIII

**Figure 3:**
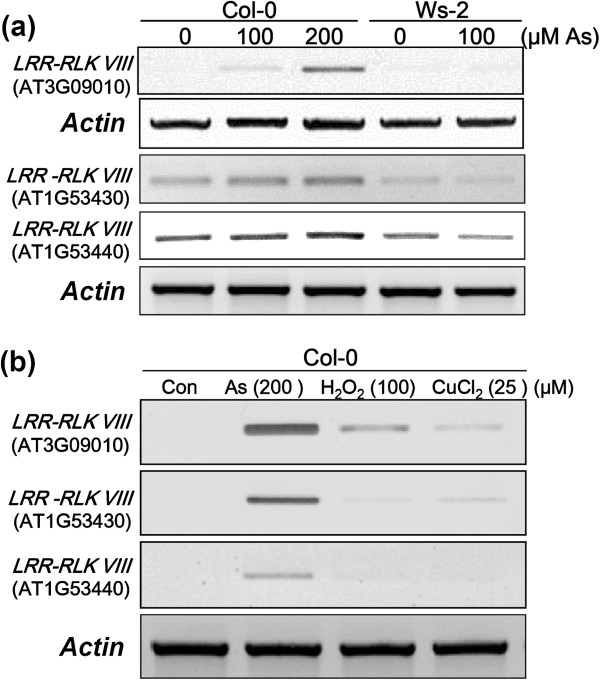
**Expression analysis of *Arabidopsis LRR-RLK VIII *gene in response to As stress. (a)** Semi-quantitative RT-PCR analysis of *LRR-RLK VIII* genes in two *Arabidopsis* accessions after exposure to As stress. The details in annotation of these selected As tolerance-associated genes are summarized in Table 2. **(b)** RT-PCR analysis of *LRR-RLK VIII* genes in response to various stresses from *Arabidopsis* Col-0 accession. The plants were treated with As (100 and 200 μM), Cu (25 μM) and H_2_O_2_ (100 μM) for 3, 12 and 24 h. The data are on the basis of three biological replicates.

Most of the As-tolerance associated genes were involved in signal transduction and regulatory mechanisms (47 out of 63, Table 2). Their up-regulation at the early stage endorsed the trigger of downstream components to cope with the stressful condition. These regulatory genes may act as the fate dominators of As tolerance. Very little is known about the role(s) of *LRR-RLK VIII* gene in regulation of plants responding to heavy metal stresses. To characterize an As tolerance-associated gene candidate encoding LRR-RLK VIII, we analyzed transcriptional regulation for *Arabidopsis LRR-RLK VIII* genes regarding its response to As stress. According to the microarray data, four genes (AT1G53430, AT1G53440, AT3G09010 and AT5G01950) belonging to the LRR-RLK VIII family were significantly upregulated by As stress (Table 2 and Additional file 1: Table S7). The data was further validated by semi-quantitative (Figure 3a). The expression level of the *LRR-RLK VIII* genes (AT1G53430, AT1G53440 and AT3G09010) was induced more significantly in As-treated Col-0 plants as compared to Ws-2. Next, we examined the expression of the *LRR-RLK VIII* genes (AT1G53430, AT1G53440 and AT3G09010) in response to various stresses (Figure 3b). The gene expression was strongly induced in root tissues with treatment of 200 μM As in comparison with H_2_O_2_ (100 μM) and Cu (25 μM) stresses (Figure 3b). Therefore, the increase in *LRR-RLK VIII* gene expression was regulated in root tissues by As. The data demonstrated the specificity of the *LRR-RLK VIII* gene expression in response to As stress.

### Functional analysis of the LRR-RLK VIII gene in response to As stress using Arabidopsis T-DNA mutants

**Figure 4:**
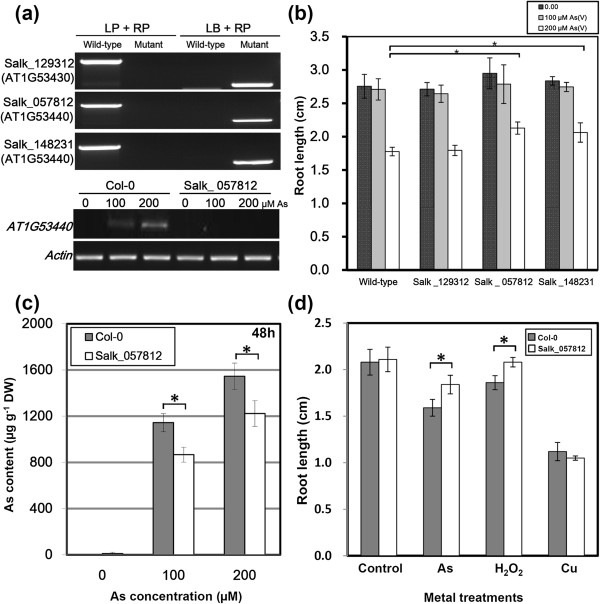
**Functional analysis of a tolerance-associated gene encoding LRR-RLK VIII in response to As stress. (a)** Identification of T-DNA insertion for *LRR-RLK VIII* mutations in the *Arabidopsis* genome by PCR Analysis. The left (LB) border primers for T-DNA insertion, and the left (LP) and right (RP) genomic primers for *LRR-RLK VIII* genes are presented in materials and methods. The sequences of junctions between T-DNA borders and the genomic target were detected in homologous *LRR-RLK VIII* mutants when compared to the wild-type plants. Two T-DNA mutant lines for locus AT1G53440 were characterized, and one for AT1G53430 (upper panel). The gene expression of *LRR-RLK VIII* (AT1G53440) in the mutant line (Salk_057812) was analyzed by RT-PCR (bottom panel). There was no characterized T-DNA mutant line for AT3G09010. **(b)** Effect of As (V) on the root elongation of *Arabidopsis* wild-type and *LRR-RLK VIII* mutant lines was assessed. Measurement of root elongation was similar to that described in Figure 1. Seedlings were grown on quarter-strength MS medium for 4 d and then transferred to medium with 100 and 200 μM As (V) and grown for an additional 4 d. As tolerance was determined by relative root growth after treatment. Root length of plants was measured after treatment with As. Root samples were collected from 3 independent experiments (each from a pool of 7 root samples). Data are mean±SD. *P ≤ 0.05 compared to As treatment (200 μM) from wild-type plants. The difference in root elongation is significant according to Student's t test. **(c)** Accumulation of As (V) by *Arabidopsis* wild-type and *LRR-RLK VIII* mutant lines was analyzed with the methods similar to Figure 1. Data are mean±SD calculated from 3 biological replicates per treatment. *P ≤ 0.05 compared with wild-type plants. **(d)** Effect of As (200 μM), Cu (50 μM) and H_2_O_2_ (100 μM) on the root elongation of *Arabidopsis* wild-type and *LRR-RLK VIII* mutant lines. Measurement of root elongation was similar to that described in Figure 1. At least three biological replicates were performed corresponding to each treatment. Data are the mean ± SE of three independent replicates.

To examine the function of an As tolerance-associated gene candidate encoding LRR-RLK VIII, we analyzed T-DNA mutant lines for *Arabidopsis LRR-RLK VIII* genes regarding its response to As stress. *Arabidopsis* mutants with a T-DNA insertion in the locus coding for LRR-RLK VIII was characterized and subjected to As treatment (Figure 4a). The insertion of the mutant line (AT1G53440: SALK_057812) located 403 bp upstream of the start codon. The insertion abolished As-induced *LRR-RLK VIII* gene expression (Figure 4a). Two of the mutant lines for AT1G53440 (SALK_057812 and SALK_148231) showed alternation in As sensitivity (Figure 4b). At 200 μM, As significantly inhibited root elongation in both the wild-type plants and the *LRR-RLK VIII* mutants when compared to untreated controls (Figure 4b). However, the root growth inhibition was less in the *LRR-RLK VIII* mutant lines as compared to the wild-type plants. Thus, knock-out of *LRR-RLK VIII* gene caused decrease in As sensitivity. The level of As concentration in roots was less in the *LRR-RLK VIII* mutant line (AT1G53440; SALK_057812) than wild-type plants (Figure 4c). In addition, the functional specificity of the *LRR-RLK VIII* gene was assessed by treatment of the *Arabidopsis* mutant lines with various stresses such as As, Cu, Cd, Zn, H_2_O_2_ and salt stress (Figure 4d and Additional file 3: Figure S2). Cu, Cd, Zn, and salt stresses inhibited root growth to a similar level between wild-type plants and the *LRR-RLK VIII* mutant line (AT1G53440: SALK_057812). Alleviation of As- and H_2_O_2−_ induced root growth inhibition was observed in the *LRR-RLK VIII* mutant line as compared to wild-type plants (Figure 4d). Thus, the loss-of-function mutation of *LRR-RLK VIII* caused As hyposensitivity, indicating its functional significance and specificity in response to As stress.

## Discussion

### Transcriptome profiling of As toxicity and tolerance

We aimed to compare changes at the physiological and global gene expression levels of *Arabidopsis* accessions tolerant and sensitive to As stress. *Arabidopsis* Col-0 accession plants were more tolerant to As stress than Ws-2 plants and accumulated significantly less As in root tissues. Microarray assay identified the early response of *Arabidopsis* to As stress and possible differences in the mechanisms to achieve tolerance in the accessions. GO analysis of the transcriptome data suggested that As stress significantly affects biological processes related to responses to heat, oxidative stress, metal ion and cell wall organization, and cytokinin and transport in both accessions. The differential expression levels of ABA- and ethylene-related genes may contribute to the As sensitivity and tolerance of the two accessions. Pathway analysis suggested that the As tolerance of the Col-0 accession was attributed to enhanced expression of regulatory and functional components such as proteasome-related mechanisms and responses to heat. The present work extends current knowledge of early transcriptional regulation by As stress in *Arabidopsis* roots and provides valuable insights into aspects of As toxicity, detoxification and acquired tolerance.

### Ecotypic variation in As tolerance

Toxicity and tolerance to heavy metals are closely related to the accumulation of heavy metals in plant tissues [9,16,27]. Plant species and even genotypes differ greatly in their ability to take up, transport and accumulate heavy metals. In maize, an Al-tolerant genotype accumulated significantly less Al in root tips than the Al-sensitive genotype [28]. *Holcus lanatus* has As-tolerant and -sensitive accessions, and relatively less As concentration accumulated in roots of As-tolerant accessions [23]. We selected *Arabidopsis* with different accessions to study As tolerance by root growth inhibition (Figures 1a and b) and As accumulation (Figure 1c). The *Arabidopsis* accession Col-0 was more tolerant to As stress than Ws-2 and accumulated less As in root tissues (Figure 1c). An As exclusion mechanism may operate in roots of As-tolerant Col-0. In As-sensitive Ws-2 plants, As toxicity was associated with relatively higher accumulation of As in roots (Figure 1). GO analysis of microarray data also suggested that the increased As accumulation of Ws-2 resulted in enhanced expression of toxin and osmotic stress-related genes (Table 1). Therefore, the ecotypic variation in As tolerance appeared to be associated with As level, as well as expression of toxin and osmotic stress-related genes.

### Comparisons of short- and long-term transcriptome responses to As stress

The transcriptional response of *Arabidopsis* Col-0 to As (V) stress has been reported [10]. As may induce genes involved in response to oxidative stress and repress that of genes induced by phosphate starvation [10]. The cysteine-rich metal-binding protein metallothionein (MT) was also induced. Previous work examined As toxicity in *Arabidopsis* in terms of root growth inhibition and transcriptional responses after 3 and 10 d [10]. Forty-six and 113 genes were induced and repressed, respectively. The physiological and metabolic parameters measured under these long treatment periods might be distorted by the severe toxic effects of As. Mechanisms of adaptation after long-term exposure are relevant. However, we aimed to understand the primary response to metal ion exposure as opposed to responses to unspecific cellular damage. Increased transcript abundance of heat shock protein and the ubiquitin-proteasome pathway (as reported in this study) are general responses to cellular stress and damage. We examined genomic gene expression profiles and biological pathways in *Arabidopsis* in response to short-term As stress (Figures 3 and 4): 1.5 and 3 h of As exposure resulted in 2451 and 1149 As-responsive genes in Col-0 and Ws-2, respectively. Only 16 genes were regulated in common after long-term [10] and short-term (this study) As exposure (Additional file 1: Table S8). Genes that have previously been reported to be As inducible or pathways were not affected by short-term As exposure (Table 1). We did not observe changes in expression of MT genes, which suggests a unique cellular response to short-term As exposure. Transcriptome data demonstrated that As stress significantly affects biological processes related to responses to heat, oxidative stress, metal ion, cell wall organization and cytokinin, as well as transport (Table 1). Therefore, *Arabidopsis* plants rapidly and simultaneously change the expression of specific sets of genes to cope with As stress in root tissues.

### As-responsive genes involved in the detoxification

Plants possess a range of potential cellular mechanisms that may be involved in the detoxification of heavy metals such as antioxidant and transporter systems [29,30]. Genes coding for proteins involved in oxidative stress such as thioredoxins and Class III peroxidase were highly represented in our microarray data of *Arabidopsis* plants exposed to As stress (Additional file 1: Table S2). Transcripts belonging to GST formed the largest group within the oxidative stress-related genes. Most of these transcripts are in the Tau subfamily (GST-Tau), and the remaining sequences are similar to the Phi and lambda subfamily (Additional file 1: Table S3). GSTs are induced by a number of intracellular and environmental factors such as oxidative stress and heavy metals [31,32] and are involved in detoxification of both endogenous and xenobiotic compounds with electrophilic centers by the nucleophilic addition of glutathione [31]. Some isoforms of GST show dual activity, additionally functioning as a glutathione peroxidase in the presence of reactive oxygen species [31]. This phenomenon provides further evidence for the role of GSTs in antioxidant metabolism. As–glutathione conjugates may be produced by GSTs in animal cells such as rat liver [15,33,34]. However, GST-mediated conjugation of glutathione with As has not been demonstrated in plants. The upregulation of Tau class GSTs has also been noted in transcriptomic and proteomic analysis of plant roots under As stress [9,32]. Thus, our results suggest that the Tau class of GSTs most likely functions at the root level to protect cells against heavy-metal-–induced oxidative damage. In addition, we found differential regulation of genes encoding GSTs between As-tolerant and -sensitive *Arabidopsis* accessions (Additional file 1: Table S2), with fewer As-induced GST genes regulated in As-tolerant Col-0 than As-sensitive Ws-2, which indicates the differential GST-based detoxification. The GST gene expression profile can therefore account for the tolerant *Arabidopsis* Col-0 accession being less affected by As stress.

### As-tolerance associated genes involved in ethylene signalling

The hormones ethylene participates in signaling cascades regulating both development and responses to stress [35]. Recently, cross-talk between ethylene signaling and sulfur assimilation in plants has been proposed [36]. It was demonstrated that sulfur can induce tolerance to Cd stress and alleviate photosynthetic inhibition through ethylene by maintaining high GSH levels [37]. However, their roles in *Arabidopsis* exposed to As has not been reported. In this study, genes coding for ethylene-signalling components were significantly enriched in As-tolerant Col-0 with short-term As exposure (Table 1 and Additional file 1: Table S2). The transcription factor gene encoding ERF (ethylene response factor) was identified as an As-tolerance associated gene (AT2G33710) (Table 2). The sulfur assimilation pathway was also affected by As stress (Additional file 2: Figure S1). Thus, regulatory interaction between sulfur assimilation and ethylene may contribute to tolerance to As in Col-0 accession. Furthermore, the predominant functional themes of the tolerance-associated genes are related to the ubiquitin/proteasome pathway and responses to heat (Table 2). These molecular signatures identified in the As-responsive transcriptome might work in concert for tolerance to As stress. A number of studies have suggested a role for ethylene in plants on exposure to heavy metals. For example, cadmium or copper induces the biosynthesis of ethylene in various plant species [38,39]. The hormone is transmittable signals, which invoke stress responses and tolerance in the shoot [40,41]. In this study, foliar necrosis was less severe in As-tolerant Col-0 than As-sensitive Ws-2 after exposure to As for 1 week (Additional file 4: Figure S3).

### As-tolerance associated genes involved in ubiquitin/proteasome pathways

The elevated expression of genes related to the proteasome pathway indicated that the oxidized or As-bound proteins in As stressed plant cells might be ubiquitinated for proteasome-mediated degradation. Expression levels of the components and intermediates of the ubiquitin/proteasome pathway were predominately induced in the Col-0 accession and identified as tolerant-associated genes under As stress (Table 2). A notable number of the proteasome-related genes belong to the component of E2 and E3 ubiquitin ligases (E3s) such as RING/U-box superfamily protein, RING-H2 finger protein and membrane-anchored ubiquitin-fold protein (Table 2). In addition, the ubiquitin-associated proteins present contained the ubiquitin fusion degradation UFD1 family protein. These are key intermediates in the tagging of proteins for proteasomal degradation. RING proteins can act as single components containing both the active site and the binding pocket for the E2-ubiquitin intermediate [42-44]. Emerging evidence suggests the potential roles of the proteasome-mediated pathway in plants with As exposure. Expression profiling of *Crambe abyssinica* under As stress identified gene networks involved in 20S proteasome degradation [27]. In *Lemna minor*, the response to As (III) involves cellular protease-chaperone machineries, including heat shock protein synthesis and the ubiquitin/proteasome pathway [45]. In addition, the As-tolerance associated genes identified in this study (Table 2) were compared with Cd-induced genes in *Arabidopsis*[46]. Gene encoding ubiquitin-specific protease (AT3G28220) was induced by short-term Cd treatment, indicating the roles of ubiquitin/proteasome pathways in heavy metal stress. Therefore, our results suggest that the proteasome-mediated protein degradation machinery is vital for As tolerance in plants.

### Potential roles of the As-tolerance associated genes encoding LRR-RLKs

RLKs consist of extracellular repeats that are linked by a transmembrane domain to either an intracellular adapter domain or a kinase domain [47]. LRR-RLKs represent the largest subfamily in the *Arabidopsis* genome with approximately 235 members, divided over 13 subfamilies (LRR I to XIII) [48]. The biological functions have been defined for only about 30 proteins, which play crucial roles in a variety of different physiological processes such as development, pathogen resistance and hormone perception [47]. In addition, it has been reported that target genes of the Cd-responsive miRNAs encode LRR-RLKs [49]. Exploration of *Arabidopsis* expression data with the *Arabidopsis* eFP Browser [50] revealed the *LRR-RLK VIII* genes were upregulated by salt and cold stresses (Additional file 5: Figure S4). Functional characterization of the LRR-RLK VIII gene (AT1G53430) using a T-DNA insertion line showed that the mutant was resistance to salt stress [48]. In this study, four genes (AT1G53430, AT1G53440, AT3G09010 and AT5G01950) belonging to the LRR-RLK VIII family were significantly upregulated by As stress in the tolerant Col-0 accession (Table 2 and Additional file 1: Table S7). The result was further validated by semi-quantitative and real-time RT-PCR analysis (Figure 3). Some members of the LRR-RLK VIII family may be functionally redundant in the regulation of As stress (Figure 4). Two of the mutant lines (AT1G53440: SALK_057812 and SALK_148231) showed alternation in As sensitivity and the metal accumulation (Figure 4). Thus, this study revealed a novel role for LRR-RLK VIII in sensitivity to As stress. Some mammalian toll-like receptors, which are the counterparts of plant LRR-RLKs, are involved in xenobiotic response such as drug metabolism [51,52]. It may be speculated that specific plant LRR-RLKs could have evolved into sensors under heavy metal stress.

### LRR-RLK VIII may involve in perception of As stress leading alternation in As sensitivity

In this study, we report the functional analysis of an *Arabidopsis LRR-RLK VIII* gene involved in the regulation of early root responses to As stress. The *Arabidopsis LRR-RLK VIII* mutants alleviated As-induced root growth inhibition, indicating that the mutant roots may be less sensitive to the heavy metal stress (Figure 4b). Accumulation of As level was decreased in the *LRR-RLK VIII* mutants as compared to wild-type plants (Figure 4c). It is suggested that the *LRR-RLK VIII* mutants are less sensitive to As stress due to reduced As accumulation in roots. Therefore, the *LRR-RLK VIII* gene appeared to function as a negative regulator of As tolerance. Similarly, knocking out expression of a *Medicago truncatula LRR-RLK* (*Srlk*) by TILLING failed to limit root growth when exposed to salt stress [53]. These *LRR-RLK* mutant plants accumulate fewer sodium ions than controls, and several early salt-regulated genes are downregulated after exposure to salt stress [53]. Taken together, the results may link the LRR-RLK receptor with perception of As stress and activation of a signaling pathway leading to alternation in As sensitivity.

## Conclusions

We performed a comparative investigation to reveal changes in gene expression that take place in roots of an As-tolerant and As-sensitive accessions of *Arabidopsis* under short-term As stress. We identified putative candidate genes for As tolerance in *Arabidopsis* to explore molecular mechanisms in response to As. Future studies involving *Arabidopsis* mutants or overexpressors with altered expression of these genes will be helpful to elucidate their biological significance and to clarify new pathways involved in As toxicity and tolerance. Analysis of As tolerance by quantitative trait locus (QTL) strategies in successive generations obtained from crosses between the two accessions will facilitate the identification of genes involved in As tolerance. The knowledge of how plants acquire tolerance to As is essential for developing novel strategies for efficient phytoremediation and As tolerant crops.

## Methods

### Plant materials and growth conditions

*Arabidopsis thaliana* Col-0 (accession no. CS22625) and Ws-2 (no. CS22659) were obtained from the *Arabidopsis* Biological Resource Center (Ohio State University, USA) and propagated for 1 generation. The seeds were vernalized for 2 d at 4°C before germination. About 500 seeds were sterilised by 10 min of incubation in 1 ml of 1.2% (v/v) sodium hypochlorite solution with a few drops of Tween 20, then rinsed 4 times with sterilized water. Surface-sterilized seeds were sown on quarter-strength MS (Murashige-Skoog) medium (M0222.0050, Duchefa Biochemie, The Netherlands) supplemented with 1.0% (w/v) sucrose, pH 5.7, with 0.05% (w/v) 2-morpholinoethanesulfonic acid monohydrate, and solidified with 1% (w/v) phytagel (Sigma, St. Louis, MO, USA). Seedlings were grown under 16-hr white light/8-hr dark at 25°C with an illumination intensity of 3,000 Lux by placing the Petri dish vertically.

### Measurement of root length

Seven 4-d-old seedlings were transferred to plates (12 × 12 × 1.7 cm) with medium supplemented with As from 100 to 300 μM. Control seedlings were incubated without As. Seedlings were allowed to grow for additional 2 d. The elongation of primary roots was measured by manually recording on the plate. Data were obtained from 3 biological replicates.

### Determination of As concentration in roots

After 3 h or 48 h of As treatment, roots of 4-d-old *Arabidopsis* seedlings were rinsed thoroughly with distilled water and oven dried for 2 d at 60°C, then digested in HNO_3_/H_2_O_2_, 3:2 (v/v), for 30 min. As concentration was determined by inductively coupled plasma atomic emission spectrometry (ICP-AES) (HORIBA Jobin Yvon 2000–2, France). Data were mean values from 3 independent biological replicates.

### RNA extraction

The medium was overlaid with a layer of nylon mesh and 4-d-old *Arabidopsis* seedlings grew on the nylon mesh to facilitate collection of root samples. Total RNA was isolated from roots with use of an RNeasy Plant Mini Kit (Qiagen, Hilden, Germany), treated with DNase (Roche, Germany) to eliminate genomic DNA contamination, then purified and concentrated by use of the RNeasy MinElute Cleanup Kit (Qiagen). Three biological replicates were grown in the same growth chamber to minimize experimental and sample-to-sample variation. For each accession, total RNA samples extracted from untreated (control) and As-treated (experimental) samples were subjected to microarray analysis.

### Microarray analysis

Microarray analysis involved Affymetrix microarrays (GeneChip *Arabidopsis* ATH1 genome array) containing 22,810 probe sets on a single chip. The data were analyzed by use of the Microarray Suite v5.0 with Affymetrix default settings and global scaling as the normalization method. The trimmed mean target intensity of each array was arbitrarily set to 100. The raw cell intensity data files (GeneChip CEL files) were analyzed by use of GeneSpring GX10 (Agilent Technologies, CA, USA). Data were normalized by the GeneChip RMA Robust Multichip Average (GC-RMA) algorithm and converted to log_2_ scale to allow for comparing the 3 biological replicates. As treated sample was compared to an untreated control sample harvested from each accession. Genes with statistically significant 2-fold change in expression in all experiments were detected by Student’s *t* test at P < 0.05. The Benjamini and Hochberg algorithm calculates false discovery rates (FDRs) that are inherently corrected for multiple testing [25]. Genes were considered as significantly up- or downregulated if the FDR value for the corresponding probe set was < 0.05. We focused our attention on the subset of these transcripts that differed by 2- fold between As-treated and untreated samples. Three biological replicates of each sample were carried out to achieve reproducibility of the chip hybridization. The microarray data have been deposited in Gene Expression Omnibus (no. GEO: GSE31977).

### Gene ontology (GO) analysis

The differentially expressed genes were classified by biological function by analysis with agriGO (http://bioinfo.cau.edu.cn/agriGO/) [54] and MapMan [55]. The gene lists in each functional categories were obtained from The Arabidopsis Information Center (http://www.arabidopsis.org/) and Arabidopsis thaliana Kinase Database (http://bioinformatics.cau.edu.cn/athKD/index.htm).

### Semi-quantitative RT-PCR

First-strand cDNA was synthesized from 2 μg of total RNA with 1 μl oligo (dT)15 primers by use of the ImProm-II Reverse Transcription System (Promega, WI, USA). The fragments of cell cycle-related genes were amplified by use of gene-specific primers (Additional file 6: Table S9). Actin was an internal control. The PCR cycling involved an initial denaturation step at 94°C for 2 min, 27–40 cycles of amplification and a final elongation step at 72°C for 5 min. PCR products were analyzed on a 2% (w/v) agarose gel. The experiments were repeated at least 2 times for each gene.

### Characterization of Arabidopsis LRR-RLK VIII T-DNA insertion mutants

The *Arabidopsis LRR-RLK VIII* T-DNA mutant lines (AT1G53430, SALK_129312; AT1G53440, SALK_148231 and SALK_057812; AT3G09010, SALK_019665) were obtained from *Arabidopsis* Biological Resource Center (ABRC; http://abrc.osu.edu/resources). The DNA samples were extracted from wild-type and *LRR-RLK VIII* T-DNA mutant lines and subject to PCR analysis. Homozygous plants were verified with *LRR-RLK VIII*-specific primers spanning the T-DNA insertion site (LP and RP) together with a T-DNA left-border primer (LB) (Additional file 6: Table S9). The PCR consisted of an initial denaturation step at 94°C for 2 min, 30 cycles of amplification with annealing temperature at 55°C and a final elongation step at 72°C for 5 min. The PCR product was separated on a 2% (w/v) agarose gel. The amplified PCR fragments were sequenced to confirm the presence of a *LRR-RLK VIII* sequence and to determine the T-DNA insertion site.

## Abbreviations

ABA: Absicisc acid; As: Arsenic; CK: Cytokinin; ET: Ethylene; GST: Glutathione S-transferase; LRRs-RLK: Leucine-Rich Repeats receptor like kinase; GO: Gene ontology; HSP: Heat shock protein; HSF: Heat shock transcription factor; PC: Phytochelatin; ABA: Abscisic acid; ABC: ATP-binding cassette; MATE: Multidrug and toxic compound extrusion; MAPK: Mitogen-activated protein kinase; MT: Metallothioneins; OPT: Oligopeptide; SnRK: Snf1-related kinases.

## Competing interest

The authors declare that they have no competing interests.

## Authors’ contributions

PYC, TTQN, GRZ and TLH designed the experiments, conducted microarray experiments, performed bioinformatic analysis and wrote the manuscript. SFF and HJH contributed to interpretation of the data, manuscript writing and modification. CYL and LYH contributed to analysis of relationship between hormone homeostasis in response to As stress. All authors read and approved the manuscript.

## Supplementary Material

Additional file 1: Table S1-S8 List of genes, expression intensity, and p-values corresponding to microarray data. Click here for file

Additional file 2: Figure S1 Transcriptional changes of genes involved in sulfur assimilation, cysteine biosynthesis and phytochelatin synthesis in As-treated roots. Click here for file

Additional file 3: Figure S2 Effects of NaCl (20 mM) , CuCl2 (25 μM) , ZnSO4 (200 μM) and CdCl2 (100 μM) on the root elongation of *Arabidopsis* wild-type and *LRR-RLK VIII* mutant lines was assessed. Click here for file

Additional file 4: Figure S3 Effects of As on the growth of shoots in 2 Arabidopsis accessions. Click here for file

Additional file 5: Figure S4 Exploring Arabidopsis gene expression data with the **(a)** eFP Browser (http://www.bar.utoronto.ca/) [50] and **(b)** CAU Bioinformatic Center (http://bioinformatics.cau.edu.cn/cgi-bin/gbrowse/arabidopsis/). Click here for file

Additional file 6: Table S9 List of primers used in this study. Click here for file

## References

[B1] Zhao FJ McGrath SP Meharg AA Arsenic as a Food Chain Contaminant: Mechanisms of Plant Uptake and Metabolism and Mitigation Strategies Annu Rev Plant Biol 2010 61 535 559 10.1146/annurev-arplant-042809-112152 20192735

[B2] Zhu YG Sun GX Lei M Teng M Liu YX Chen NC Wang LH Carey AM Deacon C Raab A Meharg AA Williams PN High percentage inorganic arsenic content of mining impacted and nonimpacted Chinese rice Environ Sci Technol 2008 42 5008 5013 10.1021/es8001103 18678041

[B3] Bhattacharjee Y Toxicology: a sluggish response to humanity's biggest mass poisoning Science 2007 315 1659 1661 10.1126/science.315.5819.1659 17379786

[B4] Carbonell-Barrachina AA Signes-Pastor AJ Vazquez-Araujo L Burlo F Sengupta B Presence of arsenic in agricultural products from arsenic-endemic areas and strategies to reduce arsenic intake in rural villages Mol Nutr Food Res 2009 53 531 541 10.1002/mnfr.200900038 19382147

[B5] Williams PN Islam MR Adomako EE Raab A Hossain SA Zhu YG Feldmann J Meharg AA Increase in rice grain arsenic for regions of Bangladesh irrigating paddies with elevated arsenic in groundwaters Environ Sci Technol 2006 40 4903 4908 10.1021/es060222i 16955884

[B6] Wei CY Chen TB Arsenic accumulation by two brake ferns growing on an arsenic mine and their potential in phytoremediation Chemosphere 2006 63 1048 1053 10.1016/j.chemosphere.2005.09.061 16297966

[B7] Zheng MZ Cai C Hu Y Sun GX Williams PN Cui HJ Li G Zhao FJ Zhu YG Spatial distribution of arsenic and temporal variation of its concentration in rice New Phytol 2011 189 200 209 10.1111/j.1469-8137.2010.03456.x 20840510

[B8] Tripathi RD Srivastava S Mishra S Singh N Tuli R Gupta DK Maathuis FJ Arsenic hazards: strategies for tolerance and remediation by plants Trends Biotechnol 2007 25 158 165 10.1016/j.tibtech.2007.02.003 17306392

[B9] Norton GJ Lou-Hing DE Meharg AA Price AH Rice-arsenate interactions in hydroponics: whole genome transcriptional analysis J Exp Bot 2008 59 2267 2276 10.1093/jxb/ern097 18453530 PMC2413274

[B10] Abercrombie JM Halfhill MD Ranjan P Rao MR Saxton AM Yuan JS Stewart CN Jr Transcriptional responses of Arabidopsis thaliana plants to As (V) stress BMC Plant Biol 2008 8 87 10.1186/1471-2229-8-87 18684332 PMC2547109

[B11] Srivastava S Srivastava AK Suprasanna P D'Souza SF Comparative biochemical and transcriptional profiling of two contrasting varieties of Brassica juncea L. in response to arsenic exposure reveals mechanisms of stress perception and tolerance J Exp Bot 2009 60 3419 3431 10.1093/jxb/erp181 19528528

[B12] Rahman F Naidu R The influence of arsenic speciation (AsIII & AsV) and concentration on the growth, uptake and translocation of arsenic in vegetable crops (silverbeet and amaranth): greenhouse study Environ Geochem Health 2009 31 Suppl 1 115 124 19225721 10.1007/s10653-008-9241-2

[B13] Oremland RS Stolz JF The ecology of arsenic Science 2003 300 939 944 10.1126/science.1081903 12738852

[B14] Mendoza-Cozatl DG Jobe TO Hauser F Schroeder JI Long-distance transport, vacuolar sequestration, tolerance, and transcriptional responses induced by cadmium and arsenic Curr Opin Plant Biol 2011 14 554 562 10.1016/j.pbi.2011.07.004 21820943 PMC3191310

[B15] Ali W Isayenkov SV Zhao FJ Maathuis FJ Arsenite transport in plants Cell Mol Life Sci 2009 66 2329 2339 10.1007/s00018-009-0021-7 19350206 PMC11115966

[B16] Bleeker PM Hakvoort HW Bliek M Souer E Schat H Enhanced arsenate reduction by a CDC25-like tyrosine phosphatase explains increased phytochelatin accumulation in arsenate-tolerant Holcus lanatus Plant J 2006 45 917 929 10.1111/j.1365-313X.2005.02651.x 16507083

[B17] Liu X Zhang S Shan XQ Christie P Combined toxicity of cadmium and arsenate to wheat seedlings and plant uptake and antioxidative enzyme responses to cadmium and arsenate co-contamination Ecotoxicol Environ Saf 2007 68 305 313 10.1016/j.ecoenv.2006.11.001 17239437

[B18] Briat JF Arsenic tolerance in plants: "Pas de deux" between phytochelatin synthesis and ABCC vacuolar transporters Proc Natl Acad Sci U S A 2010 107 20853 20854 10.1073/pnas.1016286107 21106757 PMC3000288

[B19] Clemens S Toxic metal accumulation, responses to exposure and mechanisms of tolerance in plants Biochimie 2006 88 1707 1719 10.1016/j.biochi.2006.07.003 16914250

[B20] Ma JF Yamaji N Mitani N Xu XY Su YH McGrath SP Zhao FJ Transporters of arsenite in rice and their role in arsenic accumulation in rice grain Proc Natl Acad Sci U S A 2008 105 9931 9935 10.1073/pnas.0802361105 18626020 PMC2481375

[B21] Da Silva NA Shah D Shen MWY Chen W Enhanced arsenic accumulation in Saccharomyces cerevisiae overexpressing transporters Fps1p or Hxt7p J Biotechnol 2010 150 101 107 20638426 10.1016/j.jbiotec.2010.07.012

[B22] Lou-Hing D Zhang B Price AH Meharg AA Effects of phosphate on arsenate and arsenite sensitivity in two rice (Oryza sativa L.) cultivars of different sensitivity Environ Exp Bot 2011 72 47 52 10.1016/j.envexpbot.2010.11.003

[B23] Quaghebeur M Rengel Z The distribution of arsenate and arsenite in shoots and roots of Holcus lanatus is influenced by arsenic tolerance and arsenate and phosphate supply Plant Physiol 2003 132 1600 1609 10.1104/pp.103.021741 12857839 PMC167097

[B24] Chakrabarty D Trivedi PK Misra P Tiwari M Shri M Shukla D Kumar S Rai A Pandey A Nigam D Tripathi RD Tuli R Comparative transcriptome analysis of arsenate and arsenite stresses in rice seedlings Chemosphere 2009 74 688 702 10.1016/j.chemosphere.2008.09.082 18996570

[B25] Benjamini Y Hochberg Y Controlling the False Discovery Rate - a Practical and Powerful Approach to Multiple Testing J R Stat Soc Series B 1995 57 289 300

[B26] Dixon DP Cummins L Cole DJ Edwards R Glutathione-mediated detoxification systems in plants Curr Opin Plant Biol 1998 1 258 266 10.1016/S1369-5266(98)80114-3 10066594

[B27] Paulose B Kandasamy S Dhankher OP Expression profiling of Crambe abyssinica under arsenate stress identifies genes and gene networks involved in arsenic metabolism and detoxification BMC Plant Biol 2010 10 108 10.1186/1471-2229-10-108 20546591 PMC3095275

[B28] Giannakoula A Moustakas M Syros T Yupsanis T Aluminum stress induces up-regulation of an efficient antioxidant system in the Al-tolerant maize line but not in the Al-sensitive line Environ Exp Bot 2010 67 487 494 10.1016/j.envexpbot.2009.07.010

[B29] Hall JL Cellular mechanisms for heavy metal detoxification and tolerance J Exp Bot 2002 53 1 11 10.1093/jexbot/53.366.1 11741035

[B30] Clemens S Molecular mechanisms of plant metal tolerance and homeostasis Planta 2001 212 475 486 10.1007/s004250000458 11525504

[B31] Marrs KA The Functions and Regulation of Glutathione S-Transferases in Plants Annu Rev Plant Physiol Plant Mol Biol 1996 47 127 158 10.1146/annurev.arplant.47.1.127 15012285

[B32] Ahsan N Lee DG Alam I Kim PJ Lee JJ Ahn YO Kwak SS Lee IJ Bahk JD Kang KY Renaut J Komatsu S Lee BH Comparative proteomic study of arsenic-induced differentially expressed proteins in rice roots reveals glutathione plays a central role during As stress Proteomics 2008 8 3561 3576 10.1002/pmic.200701189 18752204

[B33] Martinez-Finley EJ Aschner M Revelations from the Nematode Caenorhabditis elegans on the Complex Interplay of Metal Toxicological Mechanisms J Toxicol 2011 2011 895236 21876692 10.1155/2011/895236 PMC3157827

[B34] Liu J Chen H Miller DS Saavedra JE Keefer LK Johnson DR Klaassen CD Waalkes MP Overexpression of glutathione S-transferase II and multidrug resistance transport proteins is associated with acquired tolerance to inorganic arsenic Mol Pharmacol 2001 60 302 309 11455017 10.1124/mol.60.2.302

[B35] Yoo SD Cho Y Sheen J Emerging connections in the ethylene signaling network Trends Plant Sci 2009 14 270 279 10.1016/j.tplants.2009.02.007 19375376 PMC3063992

[B36] Iqbal NMA Khan MIR Asgher M Fatma M Khan NA Cross-talk between sulfur assimilation and ethylene signaling in plants Plant Signal Behav 2013 8 1 9 10.4161/psb.22478 PMC3745555 23104111

[B37] Masood A Iqbal N Khan NA Role of ethylene in alleviation of cadmium-induced photosynthetic capacity inhibition by sulphur in mustard Plant Cell Environ 2012 35 524 533 10.1111/j.1365-3040.2011.02432.x 21950968

[B38] Arteca RN Arteca JM Heavy-metal-induced ethylene production in Arabidopsis thaliana J Plant Physiol 2007 164 1480 1488 10.1016/j.jplph.2006.09.006 17215058

[B39] DalCorso G Farinati S Maistri S Furini A How plants cope with cadmium: Staking all on metabolism and gene expression J Integr Plant Biol 2008 50 1268 1280 10.1111/j.1744-7909.2008.00737.x 19017114

[B40] Liu Q Zhou GY Wen CK Ethylene signal transduction in Arabidopsis Zhi Wu Sheng Li Yu Fen Zi Sheng Wu Xue Xue Bao 2004 30 241 250 15599019

[B41] Johnson PR Ecker JR The ethylene gas signal transduction pathway: a molecular perspective Annu Rev Genet 1998 32 227 254 10.1146/annurev.genet.32.1.227 9928480

[B42] Vierstra RD The ubiquitin/26S proteasome pathway, the complex last chapter in the life of many plant proteins Trends Plant Sci 2003 8 135 142 10.1016/S1360-1385(03)00014-1 12663224

[B43] Mazzucotelli E Belloni S Marone D De Leonardis A Guerra D Di Fonzo N Cattivelli L Mastrangelo A The e3 ubiquitin ligase gene family in plants: regulation by degradation Curr Genomics 2006 7 509 522 10.2174/138920206779315728 18369404 PMC2269001

[B44] Dowil RT Lu X Saracco SA Vierstra RD Downes BP Arabidopsis membrane-anchored ubiquitin-fold (MUB) proteins localize a specific subset of ubiquitin-conjugating (E2) enzymes to the plasma membrane J Biol Chem 2011 286 14913 14921 10.1074/jbc.M110.158808 21345795 PMC3083199

[B45] Santos C Gaspar M Caeiro A Branco-Price C Teixeira A Ferreira RB Exposure of Lemna minor to arsenite: expression levels of the components and intermediates of the ubiquitin/proteasome pathway Plant Cell Physiol 2006 47 1262 1273 10.1093/pcp/pcj096 16926164

[B46] Jobe TO Sung DY Akmakjian G Pham A Komives EA Mendoza-Cozatl DG Schroeder JI Feedback inhibition by thiols outranks glutathione depletion: a luciferase-based screen reveals glutathione-deficient gamma-ECS and glutathione synthetase mutants impaired in cadmium-induced sulfate assimilation Plant J 2012 70 783 795 10.1111/j.1365-313X.2012.04924.x 22283708 PMC4688143

[B47] Gou X He K Yang H Yuan T Lin H Clouse SD Li J Genome-wide cloning and sequence analysis of leucine-rich repeat receptor-like protein kinase genes in Arabidopsis thaliana BMC Genomics 2010 11 19 10.1186/1471-2164-11-19 20064227 PMC2817689

[B48] ten Hove CA Bochdanovits Z Jansweijer VM Koning FG Berke L Sanchez-Perez GF Scheres B Heidstra R Probing the roles of LRR RLK genes in Arabidopsis thaliana roots using a custom T-DNA insertion set Plant Mol Biol 2011 76 69 83 10.1007/s11103-011-9769-x 21431781 PMC3097349

[B49] Ding Y Chen Z Zhu C Microarray-based analysis of cadmium-responsive microRNAs in rice (Oryza sativa) J Exp Bot 2011 62 3563 3573 10.1093/jxb/err046 21362738 PMC3130178

[B50] Winter D Vinegar B Nahal H Ammar R Wilson GV Provart NJ An "Electronic Fluorescent Pictograph" browser for exploring and analyzing large-scale biological data sets PLoS One 2007 2 e718 10.1371/journal.pone.0000718 17684564 PMC1934936

[B51] Ghose R Guo T Vallejo JG Gandhi A Differential role of Toll-interleukin 1 receptor domain-containing adaptor protein in Toll-like receptor 2-mediated regulation of gene expression of hepatic cytokines and drug-metabolizing enzymes Drug Metab Dispos 2011 39 874 881 10.1124/dmd.110.037382 21303924 PMC3082375

[B52] Ramel F Sulmon C Serra AA Gouesbet G Couee I Xenobiotic sensing and signalling in higher plants J Exp Bot 2012 63 3999 4014 10.1093/jxb/ers102 22493519

[B53] de Lorenzo L Merchan F Laporte P Thompson R Clarke J Sousa C Crespi M A novel plant leucine-rich repeat receptor kinase regulates the response of Medicago truncatula roots to salt stress Plant Cell 2009 21 668 680 10.1105/tpc.108.059576 19244136 PMC2660638

[B54] Du Z Zhou X Ling Y Zhang Z Su Z agriGO: a GO analysis toolkit for the agricultural community Nucleic Acids Res 2010 38 W64 70 10.1093/nar/gkq310 20435677 PMC2896167

[B55] Thimm O Blasing O Gibon Y Nagel A Meyer S Kruger P Selbig J Muller LA Rhee SY Stitt M MAPMAN: a user-driven tool to display genomics data sets onto diagrams of metabolic pathways and other biological processes Plant J 2004 37 914 939 10.1111/j.1365-313X.2004.02016.x 14996223

