# Supplementary material for: Transcriptome profiling of genes and pathways associated with arsenic toxicity and tolerance in Arabidopsis
Source: BMC Plant Biol. 2014 Apr 16;14:94. doi: 10.1186/1471-2229-14-94 (PMC4021232; doi:10.1186/1471-2229-14-94)
Supplement: Additional file 1: Table S1-S8 — List of genes, expression intensity, and p-values corresponding to microarray data. [file 1471-2229-14-94-S1.pdf]

Supplementary Table S1 As-responsive genes in *Arabidopsis* Col-0 and Ws-2 ecotypes (Col200 Specifically Down)

AGI	Col-200 q-value	Regulation	Col-200 FC	Ws-100 q-value	Regulation	Ws-100 FC	Gene Symbol	Target Description
AT2G01520	0.008773528	down	0.011294476	0.9521856	down	0.978533232	MLP328	unknown protein ;supported by full-length cDNA: Ceres:17603.
AT1G08430	0.001507366	down	0.011619336	0.42060366	down	0.912178717	ALMT1	hypothetical protein identical to hypothetical protein GB:AAF22890 GI:6664308 from [Arabidopsis thaliana]
AT3G25820 /// AT3G25830	0.007019117	down	0.015999146	0.5775432	down	0.792608765	ATTPS-CIN	limonene cyclase, putative similar to limonene cyclase GB:BAA08367 from [Perilla frutescens], contains Pfam profile: PF01397 terpene synthase family
AT5G09480	0.003494469	down	0.02616916	0.62483275	down	0.732168297		PEE-rich protein ;supported by full-length cDNA: Ceres:20407.
AT2G24762	0.00183422	down	0.030056564	0.22715873	down	0.747559145	ATGDU4	Expressed protein ; supported by full-length cDNA: Ceres: 19631.
AT3G45710	0.000470407	down	0.04511993	0.6177287	down	0.894495187		putative transporter protein peptide transport protein - Hordeum vulgare, PIR:T04378
AT3G25820 /// AT3G25830	0.004940283	down	0.048073849	0.5775432	down	0.792608765	ATTPS-CIN	terpene synthase-related protein contains Pfam profile: PF01397 terpene synthase family
AT3G08040	0.001799943	down	0.055137153	0.90637505	up	1.0243012	FRD3	MATE efflux family protein, putative contains TIGRfam profile: TIGR00797: MATE efflux family protein; supported by cDNA: gi_15912322_gb_AY056439.1_
AT4G35150 /// AT4G35160	0.006422833	down	0.056481038	0.12130084	down	0.777951849		O-methyltransferase - like protein caffeic acid O-methyltransferase, Pinus taeda, gb:U39301
AT5G09520	0.021792412	down	0.062388052	0.008154522	up	3.5036473		putative proline-rich protein surface protein C PspC, Streptococcus pneumoniae, EMBL:AF145055
AT1G74460	0.00996064	down	0.063743155	0.04199731	up	2.1712277		putative lipase/acylhydrolase contains Pfam profile: PF00657 lipase/acylhydrolase with GDSL-like motif; similar to anther-specific pro-rich protein APG GB:P40603 [Brassica napus] (Plant J 1993 Jan;3(1):111-20);supported by full-length cDNA: Ceres:30001.
AT3G46900	0.02780364	down	0.066245618	0.8770325	down	0.854215047	COPT2	copper transport protein - like protein copper transporter protein Arabidopsis thaliana,PID:g1082054
AT2G35770	0.004672398	down	0.068260033	0.010364546	up	1.973554	SCPL28	putative serine carboxypeptidase II
AT5G06330	0.004397515	down	0.07206826	0.90420675	up	1.0283283		harpin-induced protein-like
AT3G50400	0.021764142	down	0.076825662	0.015037157	up	2.7990916		putative protein RXF26, Arabidopsis thaliana, EMBL:AB008020
AT4G01440	0.000679271	down	0.078043908	0.29819286	down	0.725266613		predicted protein of unknown function similar to F3D13.4 similar to M. truncatula MtN21, GenBank accession number Y15293;supported by full-length cDNA: Ceres:40560.
AT5G36140	0.011543379	down	0.07888054	0.04883703	up	3.5131638	CYP716A2	putative protein contains similarity to cytochrome P450
AT2G42060	0.012803973	down	0.079643325	0.9339343	up	1.0148371		unknown protein
AT5G14130	0.0088989	down	0.080285927	0.3651273	up	1.1644521		peroxidase ATP20a (emb|CAA67338.1) ; supported by cDNA: gi_16209688_gb_AY057607.1_
AT5G10280	0.001382239	down	0.080311203	0.19920635	down	0.765266863	MYB92	putative transcription factor MYB92 ; supported by cDNA: gi_3941523_gb_AF062916.1_AF062916
AT5G03760	0.001167618	down	0.083389176	0.73022085	up	1.0593991	ATCSLA09	putative protein beta-(1-3)-glucosyl transferase, Bradyrhizobium japonicum, EMBL:AF047687; supported by cDNA: gi_16974551_gb_AY060563.1_
AT5G37690	0.019272916	down	0.085588615	0.7274738	down	0.783477402		putative protein predicted protein, Arabidopsis thaliana
AT4G31330	0.008997475	down	0.087756148	0.030665101	up	1.7115278		predicted protein ; supported by cDNA: gi_15293232_gb_AY051050.1_
AT2G23540	0.01664685	down	0.087967297	0.75859594	up	1.1475761		putative GDSL-motif lipase/hydrolase similar to APG proteins; pFAM domain PF00657
AT3G12500	0.007151266	down	0.08875878	0.5589554	down	0.882838113	ATHCHIB	basic chitinase identical to basic chitinase GB:AAA32769 GI:166666 [Arabidopsis thaliana] (Plant Physiol. 93, 907-914 (1990)); supported by cDNA: gi_15451095_gb_AY054628.1_
AT5G37990	0.03459874	down	0.08948075	0.93746984	down	0.970084157		putative protein AtPP protein, Brassica napus, EMBL:BNA245479
AT2G28960	0.005203857	down	0.090764314	0.36111468	down	0.824142836		putative receptor-like protein kinase
AT4G16370	0.010184507	down	0.091680928	0.6342574	up	1.1510923	ATOPT3	isp4 like protein ; supported by cDNA: gi_15451019_gb_AY054590.1_
AT4G19810	0.030709092	down	0.092226437	0.07003374	up	3.5503833		putative chitinase chitinase (EC 3.2.1.14) / lysozyme (EC 3.2.1.17) PZ precursor, pathogenesis-related, common tobacco, PIR2:S51591
AT3G26460	0.021210507	down	0.09233867	0.3981512	up	1.3488573		major latex protein, putative similar to major latex protein type1 GB:CAA63026 [Arabidopsis thaliana];supported by full-length cDNA: Ceres:20325.
AT1G23850	0.001322513	down	0.093563014	0.02758923	down	0.765914301		unknown protein ;supported by full-length cDNA: Ceres:37069.
AT2G16005	0.024114667	down	0.093655162	0.6340943	down	0.833085629		Expressed protein ; supported by full-length cDNA: Ceres: 994.
AT1G31710	0.002811891	down	0.094490692	0.16057102	down	0.758280959		copper amine oxidase, putative similar to copper amine oxidase GI:685197 from [Pisum sativum]
AT3G53980	0.001966837	down	0.098812145	0.019709116	down	0.724372183		putative protein hypothetical protein At2g37870 - Arabidopsis thaliana, EMBL:AC007661;supported by full-length cDNA: Ceres:5920.
AT4G11650	0.020340016	down	0.098855559	0.81325257	down	0.906032719	ATOSM34	osmotin precursor ;supported by full-length cDNA: Ceres:13796.
AT2G39380	0.001024829	down	0.099640189	0.042159356	down	0.722556039	ATEXO70H2	hypothetical protein predicted by grail
AT1G64780	0.001167618	down	0.101064747	0.7760804	down	0.942329438	ATAMT1;2	ammonium transporter, puitative similar to ammonium transporter GI:5880357 from [Arabidopsis thaliana]; supported by cDNA: gi_4324713_gb_AF110771.1_AF110771
AT4G23670	0.007496879	down	0.102932697	0.20213935	up	1.2421936		putative major latex protein major latex protein type 1 - Arabidopsis thaliana, EMBL:X91960;supported by full-length cDNA: Ceres:108949.
AT1G51840	0.00227261	down	0.103651551	0.6728277	down	0.882858456		light repressible receptor protein kinase, putative similar to light repressible receptor protein kinase GI:1321686 from [Arabidopsis thaliana]
AT4G28040	0.010100544	down	0.103795091	0.1459084	up	1.2533817		Medicago nodulin N21-like protein MtN21 gene, Medicago truncatula, Y15293; supported by cDNA: gi_13899060_gb_AF370525.1_AF370525
AT2G22510	0.028796768	down	0.110308835	0.13291158	up	2.0470135		unknown protein
AT1G01610	0.010323489	down	0.110348359	0.001810002	up	2.7479565	ATGPAT4	hypothetical protein similar to unknown protein GI:3335359 from [Arabidopsis thaliana]
AT1G31710	0.000881454	down	0.110698918	0.16057102	down	0.758280959		copper amine oxidase, putative similar to copper amine oxidase GI:685197 from [Pisum sativum]
AT5G05960	0.001573711	down	0.112102031	0.13414317	down	0.722849624		putative protein similar to unknown protein (emb|CAB88360.1);supported by full-length cDNA: Ceres:29476.
AT2G21100	0.00408383	down	0.113538269	0.04985921	up	1.6738546		putative disease resistance response protein
AT4G27560 /// AT4G27570	0.001982334	down	0.114776762	0.46040776	up	1.2206608		UDP rhamnose--anthocyanidin-3-glucoside rhamnosyltransferase - like protein UDP rhamnose--anthocyanidin-3-glucoside rhamnosyltransferase, Petunia x hybrida, PIR2:S36655
AT4G14440	0.014129692	down	0.117731521	0.011962639	up	2.3215673		carnitine racemase like protein
AT5G09530	0.028002966	down	0.122265382	0.043509297	up	2.1629276		periaxin - like protein periaxin, Rattus norvegicus, PIR:I58157; supported by cDNA: gi_15451135_gb_AY054648.1_
AT3G24290 /// AT3G24300	0.003231889	down	0.123158207	0.0165676	up	1.5706629		ammonium transporter, putative similar to GB:AAD54638 from [Arabidopsis thaliana] (Plant Cell (1999) 11 (5), 937-948)
AT4G38080	0.026976224	down	0.124882142	0.44819918	up	1.4551433		putative protein
AT1G28400	0.001259501	down	0.126037064	0.15145145	down	0.887756506		hypothetical protein predicted by genemark.hmm
AT1G44800	0.001118336	down	0.126954923	0.023916557	down	0.817301621		nodulin protein, putative similar to GB:2598575 from [Medicago truncatula];supported by full-length cDNA: Ceres:149985.
AT3G63470	0.004844041	down	0.127971148	0.01509288	down	0.837714861	SCPL40	serin carboxypeptidase - like protein carboxypeptidase D II-3 precursor, barley, PIR:S44191;supported by full-length cDNA: Ceres:36847.
AT5G06740	0.001912657	down	0.128833748	0.027046395	down	0.733379082		lectin-like protein kinase
AT2G28470	0.002089313	down	0.130692722	0.31239355	down	0.83503123	BGAL8	putative beta-galactosidase
AT3G16470	0.001952861	down	0.131460795	0.15182735	down	0.75572252	JR1	putative lectin contains Pfam profile: PF01419 jacalin-like lectin domain; similar to jasmonate inducible protein GB:Y11483 (Brassica napus), myrosinase binding protein GB:BAA84545 (Arabidopsis thaliana);supported by full-length cDNA: Ceres:30003.
AT3G47620	0.002331507	down	0.133391275	0.025187373	down	0.752071506		putative protein auxin-induced basic helix-loop-helix transcription factor - Gossypium hirsutum, EMBL:AF165924
AT4G30520	0.000674975	down	0.133712596	0.3520317	down	0.764995928		receptor-like kinase homolog somatic embryogenesis receptor-like kinase - Daucus carota,PID:g2224911
AT2G03200	0.014135016	down	0.135028955	0.78158593	down	0.877836036		putative chloroplast nucleoid DNA binding protein ;supported by full-length cDNA: Ceres:39349.
AT5G63590	0.001225199	down	0.135624244	0.100858666	down	0.810603274	FLS	flavonol synthase ;supported by full-length cDNA: Ceres:21505.
AT1G56320	0.025797915	down	0.136919749	0.17303972	up	1.6566701		hypothetical protein predicted by genemark.hmm
AT4G25100	0.006570482	down	0.13714603	0.9753538	down	0.976363509	FSD1	superoxide dismutase (EC 1.15.1.1) (Fe)(fragment) ;supported by full-length cDNA: Ceres:32935.
AT1G19550 /// AT1G19570	0.000592182	down	0.139393773	0.079694964	down	0.808483776	DHAR1	GSH-dependent dehydroascorbate reductase 1, putative similar to GB:BAA90672 from [Oryza sativa]; supported by full-length cDNA: Ceres: 15122.
AT4G37410	0.001885503	down	0.140113167	0.080636725	up	1.2701912	CYP81F4	cytochrome P450 monooxygenase - like protein cytochrome P450 monooxygenase CYP91A2, Arabidopsis thaliana, D78607;supported by full-length cDNA: Ceres:102007.
AT2G02100	0.001866582	down	0.140216879	0.08707665	down	0.884099115	LCR69	protease inhibitor II identical to GB:X69139, contains a gamma-thionin family signature (PDOC00725); supported by cDNA: gi_13878184_gb_AF370355.1_AF370355
AT1G72230	0.001337301	down	0.140583328	0.049068242	up	1.3858222		blue copper protein, putative similar to GI:562778 from [Pisum sativum] (Thesis (1994) BIOLOGICAL SCIENCES, DURHAM UNIVERSITY); supported by full-length cDNA: Ceres: 16991.
AT3G49860	0.002066824	down	0.144822248	0.05326612	down	0.838911325	ATARLA1B	putative protein various ADP-RIBOSYLATION FACTOR (ARF) - like proteins
AT4G20390	0.013792899	down	0.145005713	0.5639967	down	0.790594143		putative protein predicted proteins, Arabidopsis thaliana;supported by full-length cDNA: Ceres:248721.
AT5G24380	0.002679843	down	0.145635332	0.34976757	up	1.260589	YSL2	putative protein contains similarity to integral membrane protein
AT1G13830	0.002701426	down	0.147838659	0.05668389	down	0.712001005		hypothetical protein contains similarity to beta 1,3-glucanase (Glc1) GI:924952 from [Triticum aestivum]
AT5G40890	0.001085405	down	0.15087037	0.050420452	down	0.723816359	ATCLC-A	anion channel protein (gb|AAC05742.1) ; supported by cDNA: gi_1619955_gb_U72151.1_ATU72151
AT4G16260	0.003033525	down	0.152729546	0.47382528	up	1.0876298		beta-1,3-glucanase class I precursor ;supported by full-length cDNA: Ceres:3176.
AT1G78320	0.006324846	down	0.156180963	0.50916106	up	1.1366547	ATGSTU23	glutathione transferase, putative similar to glutathione transferase GI:2853219 from [Carica papaya];supported by full-length cDNA: Ceres:18518.
AT1G22330	0.007003678	down	0.156938799	0.11801012	down	0.769000838		glycine-rich RNA-binding protein, putative similar to glycine-rich RNA-binding protein GI:17818 from [Brassica napus]
AT1G22330	0.007003678	down	0.156938799	0.028254492	down	0.770916229		glycine-rich RNA-binding protein, putative similar to glycine-rich RNA-binding protein GI:17818 from [Brassica napus]
AT3G19370	0.000287634	down	0.158590818	0.49172115	down	0.928782017		unknown protein
AT3G51860	0.001377628	down	0.158933543	0.20415299	up	1.456134	CAX3	Ca2+/H+-exchanging protein-like Arabidopsis thaliana high affinity calcium antiporter CAX1 encoded by GenBank Accession Number U57411
AT5G24470	0.001466356	down	0.160962963	0.2755425	up	1.1809473	APRR5	putative protein contains similarity to two-component response regulator protein; supported by cDNA: gi_10281005_dbj_AB046955.1_AB046955
AT2G43290	0.001133895	down	0.162888973	0.09896758	down	0.770749383	MSS3	putative calcium binding protein ;supported by full-length cDNA: Ceres:31535.
AT1G28660 /// AT1G28670	0.001573711	down	0.16320229	0.16857886	down	0.839282095		lipase identical to lipase GB:AAA93262 GI:1145627 [Arabidopsis thaliana] (FEBS Lett. 377 (3), 475-480 (1995)); supported by cDNA: gi_1145626_gb_U38916.1_ATU38916
AT2G42320	0.000972307	down	0.163869561	0.066390626	down	0.771240384		unknown protein
AT4G12470	0.00777926	down	0.164474434	0.5406593	up	1.3597647		pEARLI 1-like protein Arabidopsis thaliana pEARLI 1 mRNA, PID:g871780
AT2G25790	0.001562901	down	0.164784615	0.9351975	down	0.994071754		putative receptor-like protein kinase ; supported by cDNA: gi_17063166_gb_AY062104.1_
AT5G43150	0.00082477	down	0.166724423	0.079835616	down	0.803550407		unknown protein
AT4G01140	0.001652505	down	0.167631658	0.41988695	down	0.890647859		hypothetical protein
AT3G47210	0.002487348	down	0.170340444	0.9314221	up	1.0100819		putative protein various predicted proteins, Arabidopsis thaliana
AT2G14100	0.004447613	down	0.171207849	0.09775277	down	0.736588455	CYP705A13	putative cytochrome P450
AT1G05000	0.002024347	down	0.171977548	0.09929064	down	0.76849715		unkown protein ESTs gb|H36249, gb|AA59732 and gb|AA651219 come from this gene
AT5G05860	0.004250747	down	0.178672497	0.122975715	down	0.794530074	UGT76C2	glucuronosyl transferase-like protein ; supported by cDNA: gi_15010631_gb_AY045617.1_
AT5G05160	0.006015687	down	0.179122486	0.18090868	up	1.1321013		receptor-like protein kinase
AT3G21230	0.001694575	down	0.181275377	0.16423926	down	0.772164221	4CL5	putative 4-coumarate:CoA ligase 2 similar to GB:AAD47192 from [Arabidopsis thaliana]
AT4G15920	0.011609195	down	0.181655848	0.48933542	down	0.883689652		cytochrome c oxidoreductase like protein
AT1G13930	0.000909821	down	0.182315116	0.030614566	down	0.721090699		unknown protein ;supported by full-length cDNA: Ceres:1505.
AT5G66815	0.010905291	down	0.185332373	0.2934407	down	0.863445789		Expressed protein ; supported by full-length cDNA: Ceres: 14679.
AT1G60680	0.001005777	down	0.18547135	0.94731283	down	0.992157983	AGD2	auxin-induced protein, putative similar to auxin-induced protein(atb2) GI:6562980 from [Arabidopsis thaliana]
AT2G15970	0.000757088	down	0.187022872	0.12667933	down	0.796670935	COR413-PM1	similar to cold acclimation protein WCOR413 [Triticum aestivum] ;supported by full-length cDNA: Ceres:7835.
AT5G59520	0.003643383	down	0.19032488	0.78283674	down	0.932089801	ZIP2	putative zinc transporter ZIP2 - like Z25114; supported by cDNA: gi_3252867_gb_AF033536.1_AF033536
AT4G25010	0.00993399	down	0.192501675	0.077079654	up	1.3266523		MtN3 - like protein MtN3 (induced during root nodule development), Medicago truncatula, gb:Y08726
AT1G38065 /// AT1G38131	0.001380261	down	0.193107066	0.6349401	down	0.956121299		axi 1 protein, putative similar to axi 1 protein GB:X80301 GI:559920 from [Nicotiana tabacum]
AT1G70850	0.003999229	down	0.197312159	0.06837191	down	0.751686691	MLP34	major latex protein (MLP149), putative similar to major latex protein (MLP149) GI:294061 from [Papaver somniferum];supported by full-length cDNA: Ceres:30081.
AT5G23920	0.001135186	down	0.197734618	0.22211123	down	0.862079147		unknown protein
AT4G12480	0.003425709	down	0.198127065	0.2226464	up	1.8491665	pEARLI 1	pEARLI 1 ; supported by cDNA: gi_871779_gb_L43080.1_ATHPEAR
AT3G25930	0.002387848	down	0.198221768	0.2019145	down	0.796963632		unknown protein ;supported by full-length cDNA: Ceres:2767.
AT1G08500	0.002101625	down	0.199014623	0.49363413	down	0.957370769		unknown protein identical to unknown protein GB:AAC32929 GI:3395758 from [Arabidopsis thaliana];supported by full-length cDNA: Ceres:123625.
AT1G14220	0.017246287	down	0.200640847	0.1645468	down	0.767223123		ribonuclease contains similarity to S-like ribonuclease PD1 GI:9957752 from [Prunus dulcis]; contains ribonuclease T2 family histidine protein motif
AT1G67030	0.003360893	down	0.20076098	0.1434917	up	1.6831114	ZFP6	zinc finger protein (ZFP6) identical to zinc finger protein GI:790683 from [Arabidopsis thaliana]; supported by cDNA: gi_15215716_gb_AY050387.1_
AT2G06925	0.001236387	down	0.20368868	0.048121832	down	0.707199775	ATSPLA2-ALPHA	Expressed protein ; supported by full-length cDNA: Ceres: 7600.
AT4G28940	0.020225408	down	0.204926356	0.5061981	down	0.791412228		putative protein vegetative storage protein win4.5 (fragment) -Populus,PID:g309839
AT4G17280	0.004605718	down	0.205091015	0.006399318	up	2.2692916		hypothetical protein
AT2G20750	0.001730385	down	0.205905213	0.08052491	down	0.746060539	ATEXPB1	beta-expansin ;supported by full-length cDNA: Ceres:109135.
AT5G38020	0.03251033	down	0.208027433	0.77879715	down	0.934552975		SAMT-like protein S-adenosyl-L-methionine:salicylic acid carboxyl methyltransferase (SAMT)- Clarkia breweri, EMBL:AF133053
AT3G20110	0.003274166	down	0.210593165	0.8812009	down	0.973654468	CYP705A20	cytochrome P450, putative contains Pfam profile: PF00067 cytochrome P450
AT5G47990	0.00451389	down	0.212247996	0.14034832	down	0.70557683	CYP705A5	cytochrome P450
AT2G24850	0.004369621	down	0.213944244	0.6674457	down	0.876598818	TAT3	putative tyrosine aminotransferase ;supported by full-length cDNA: Ceres:14570.
AT1G64370	0.001249441	down	0.214918804	0.07656067	down	0.79994522		unknown protein ;supported by full-length cDNA: Ceres:9231.
AT5G66690	0.001452713	down	0.214922296	0.023770722	down	0.74893666	UGT72E2	UTP-glucose glucosyltransferase ;supported by full-length cDNA: Ceres:151441.
AT4G35300	0.010370955	down	0.215033093	0.21990436	down	0.854973642	TMT2	putative sugar transporter protein sugar transporter, Arabidopsis thaliana, db_xref=PID:g1495273
AT5G05790	0.000876234	down	0.215306408	0.16343236	down	0.838840742		putative protein contains similarity to I-box binding factor
AT5G54960	0.003171321	down	0.218601482	0.8086712	down	0.953433452	PDC2	pyruvate decarboxylase (gb|AAB16855.1)
AT5G48000	0.00380895	down	0.218865779	0.08109387	down	0.709415727	CYP708A2	cytochrome P450-like protein
AT1G23760	0.000438549	down	0.220047372	0.33179647	up	1.1212987	JP630	putative polygalacuronase isoenzyme 1 beta subunit ; supported by cDNA: gi_1762427_gb_U59467.1_ATU59467
AT4G01390	0.02684985	down	0.220468518	0.17614335	up	1.3322809		hypothetical protein
AT1G71960	0.02388488	down	0.221327616	0.11348282	up	1.2784139		putative ABC transporter similar to breast cancer resistance protein GB:AAC97367 from [Homo sapiens]; supported by cDNA: gi_15292752_gb_AY050810.1_
AT3G23730	0.004825411	down	0.221704173	0.0628973	down	0.81515291		xyloglucan endotransglycosylase, putative similar to xyloglucan endotransglycosylase 1 GB:CAA10231 from [Fagus sylvatica] (Plant Physiol.(1999) 119, 1148-1148);supported by full-length cDNA: Ceres:10852.
AT2G26360	0.01234	down	0.22528456	0.07419185	down	0.719511889		putative mitochondrial carrier protein
AT2G07783	0.002577185	down	0.223422483	0.49555504	down	0.859869183		cytochrome c biogenesis orf382 Protein sequence is in conflict with the conceptual translation
AT1G24625	0.001801345	down	0.223524726	0.006211679	down	0.72865691	ZFP7	zinc finger protein 7, ZFP7 ; supported by cDNA: gi_790684_gb_L39650.1_ATHZFPG
AT5G45800	0.001148884	down	0.224637439	0.002648022	down	0.726008944	MEE62	receptor kinase-like protein
AT5G38030	0.001294623	down	0.226117268	0.30450368	up	1.1163758		putative transmembrane protein putative multidrug efflux protein NorM - Vibrio parahaemolyticus, EMBL:AB010463; supported by cDNA: gi_13937233_gb_AF372972.1_AF372972
AT4G31100	0.003340181	down	0.227446595	0.058906704	down	0.717415851		serine/threonine-specific protein kinase - like serine/threonine-specific protein kinase PRO25, Arabidopsis thaliana, PIR2:A46373
AT2G23610	0.001446053	down	0.23228944	0.32252854	down	0.930159616		putative acetone-cyanohydrin lyase
AT1G70690	0.001629261	down	0.232806051	0.20151474	down	0.825462889		unknown protein similar to hypothetical protein GB:AAD12705 [Arabidopsis thaliana];supported by full-length cDNA: Ceres:40506.
AT5G51550	0.000470407	down	0.234651867	0.0571395	down	0.704457922		putative protein similar to unknown protein (gb|AAD25141.1);supported by full-length cDNA: Ceres:33455.
AT4G00880	0.012070192	down	0.234750821	0.82648414	down	0.964358003		coded for by A. thaliana cDNA T43845 similar to auxin-induced protein
AT5G67160	0.013928049	down	0.237519028	0.33612964	up	1.1635404		anthranilate N-hydroxycinnamoyl/benzoyltransferase-like protein ;supported by full-length cDNA: Ceres:32856.
AT2G43535	0.001957762	down	0.239313194	0.7825583	down	0.969562336		Expressed protein ; supported by full-length cDNA: Ceres: 91704.
AT1G50560	0.004617967	down	0.240327422	0.10116743	down	0.760229303	CYP705A25	cytochrome P450, putative similar to CYTOCHROME P450 93A3 GB:O81973 from [Glycine max]
AT3G23820	0.001005777	down	0.240385309	0.017168464	down	0.743726887	GAE6	NAD dependent epimerase, putative contains Pfam profile: PF01370 NAD dependent epimerase/dehydratase family; supported by cDNA: gi_13877894_gb_AF370210.1_AF370210
AT2G28250	0.005204357	down	0.240615085	0.09399132	down	0.777975816		putative protein kinase contains a protein kinase domain profile (PDOC00100)
AT5G48010	0.003164308	down	0.241361666	0.961164	up	1.0122463		cycloartenol synthase
AT2G48040	0.001999927	down	0.24136889	0.10886978	down	0.826132078		unknown protein
AT1G13110	0.002821214	down	0.242067332	0.020913906	down	0.704855503	CYP71B7	putative cytochrome P450 monooxygenase identical to gb|X97864 cytochrome P450 from Arabidopsis thaliana and is a member of the PF|00067 Cytochrome P450 family. ESTs gb|T44875, gb|T04814, gb|R65111, gb|T44310 and gb|T04541 come from this gene
AT5G41040	0.009140977	down	0.242722629	0.00869683	up	2.482972		N-hydroxycinnamoyl/benzoyltransferase-like protein ; supported by cDNA: gi_14334525_gb_AY034954.1_
AT2G35470	0.001975953	down	0.244441283	0.85445803	up	1.0166621		unknown protein ;supported by full-length cDNA: Ceres:13821.
AT3G07600	0.010384273	down	0.244712228	0.26190057	down	0.783491152		unknown protein identical to residues 23 to 179 of ATFP4 (putative metal-binding protein) GB:AAD09508 [Arabidopsis thaliana]
AT3G19400	0.002457038	down	0.245943918	0.89905286	down	0.984811644		cysteine proteinase non-consensus AT acceptor site at exon 3; contains similarity to cysteine protease CYP1 GI:2828252, TDI-65 GI:5726641 from [Lycopersicon esculentum]
AT1G51830	0.002344782	down	0.246905655	0.027748335	down	0.776141955		light repressible receptor protein kinase, putative similar to light repressible receptor protein kinase GI:1321686 from [Arabidopsis thaliana]
AT5G24100	0.003774856	down	0.247228782	0.026823306	down	0.769614689		receptor-like protein kinase
AT4G19840	0.010784306	down	0.247481903	0.14974761	down	0.736832038	ATPP2-A1	lectin like protein lectin phloem protein PP2, winter squash, PIR2:S38462;supported by full-length cDNA: Ceres:17437.
AT1G55760	0.003157988	down	0.250283665	0.22613879	down	0.714861637		hypothetical protein predicted by genemark.hmm
AT1G33240	0.003295129	down	0.250820371	0.19076446	down	0.893962064	AT-GTL1	DNA-binding factor, putative similar to GT-2 factor GB: CAA51289 GI:416490 from [Arabidopsis thaliana]
AT5G44160	0.001562901	down	0.251033385	0.5546306	up	1.0598912	NUC	putative protein contains similarity to zinc finger protein ID1; supported by cDNA: gi_14335045_gb_AY037202.1_
AT2G17230	0.000787158	down	0.251715175	0.09079097	down	0.724619198		unknown protein ;supported by full-length cDNA: Ceres:641.
AT3G19930	0.000541825	down	0.255377268	0.43177855	down	0.875872489	STP4	monosaccharide transport protein, STP4 identical to GB:S25009 from [Arabidopsis thaliana]; supported by cDNA: gi_13605905_gb_AF367352.1_AF367352
AT4G38470	0.001237319	down	0.2571863	0.11462523	down	0.793192945		protein kinase like protein protein kinase 6 (EC 2.7.1.-) - soybean, PIR2:S29851
AT4G22120	0.000822181	down	0.257267022	0.3901171	down	0.861793489		putative protein hypothetical protein HYP1, Arabidopsis thaliana, PIR2:S51583
AT1G47480 /// AT2G05440 ///	0.006530769	down	0.258642848	0.7449429	down	0.896893564		putative glycine-rich protein
AT2G07708	0.021017548	down	0.258697025	0.7063962	down	0.929361293		hypothetical protein
AT4G15400	0.002599312	down	0.259023961	0.83945626	down	0.97929314		HSR201 like protein
AT3G43190	0.004223302	down	0.259380785	0.21155654	down	0.73540524	SUS4	sucrose synthase -like protein SUCROSE SYNTHASE (SUCROSE-UDP GLUCOSYLTRANSFERASE), Arabidopsis thalina, SWISSPROT:SUS1_ARATH; supported by cDNA: gi_14334569_gb_AY034958.1_
AT1G76890	0.003652526	down	0.261432372	0.15471278	down	0.764035504	GT2	trihelix DNA-binding protein (GT2) identical to GT2 factor [Arabidopsis thaliana] GI:416490, GI:2664202 (DNA binding factor GT-2 from Arabidopsis);supported by full-length cDNA: Ceres:3190.
AT4G05200	0.001490481	down	0.262705716	0.9856362	up	1.0026414		receptor protein kinase - like protein receptor-like protein kinase homolog RK20-1, Phaesolus vulgaris, gb:AAD21872
AT1G21310	0.001157079	down	0.262776254	0.27217302	down	0.942082821	ATEXT3	hypothetical protein
AT4G37430	0.008550385	down	0.269020293	0.16749284	up	1.2660105	CYP91A2	cytochrome P450 monooxygenase (CYP91A2) ;supported by full-length cDNA: Ceres:33365.
AT1G49310	0.010520349	down	0.270145383	0.5176115	down	0.881533452		hypothetical protein predicted by genscan+
AT1G64170	0.027494289	down	0.270789088	0.007676224	up	2.4809413	ATCHX16	hypothetical protein similar to hypothetical protein GB:AAF24561 GI:6692096 from [Arabidopsis thaliana]
AT4G13235	0.00135465	down	0.271021498	0.96860164	down	0.997686863	EDA21	putative protein late embryogenesis abundant protein -Picea glauca,PID:g1161171
AT1G72150	0.001004829	down	0.271213095	0.022597432	down	0.704191878	PATL1	cytosolic factor, putative similar to GI:807956 from [Saccharomyces cerevisiae]; supported by cDNA: gi_15028180_gb_AY045913.1_
AT4G08930	0.002180699	down	0.272199117	0.6185145	down	0.926131133	ATAPRL6	putative protein disulfide isomerase
AT2G19110	0.002034596	down	0.272341434	0.049351502	down	0.790063467	HMA4	putative cadmium-transporting ATPase T20K24.13 has been merged with T20K24.12 per suggestion of Dr. Kristian Axelsen (axe@biobase.dk)
AT3G14310	0.003334644	down	0.273330986	0.08698821	down	0.874611268	ATPME3	putative pectin methylesterase similar to pectin methylesterase GB:Q42534 from [Arabidopsis thaliana];supported by full-length cDNA: Ceres:13261.
AT1G74680	0.001985315	down	0.27394878	0.15950134	down	0.887835876		hypothetical protein predicted by genemark.hmm; supported by cDNA: gi_16209708_gb_AY057616.1_
AT3G14067	0.001562901	down	0.27402701	0.003779925	down	0.745361207		subtilisin-like serine protease, putative contains similarity to cucumisin-like serine protease GI:3176874 from [Arabidopsis thaliana]
AT3G26210	0.002939021	down	0.274180845	0.02520343	up	2.0633397	CYP71B23	cytochrome P450, putative contains Pfam profile: PF00067 cytochrome P450
AT5G20700	0.002512098	down	0.274760933	0.036932625	down	0.735993365		putative protein predicted proteins, Arabidopsis thaliana
AT1G11380	0.0030319	down	0.274764806	0.007676405	down	0.710178549		unknown protein ;supported by full-length cDNA: Ceres:113571.
AT5G59590	0.009867871	down	0.27498838	0.4483304	down	0.708718879		glucuronosyl transferase - like protein glucuronosyl transferase homolog, ripening-related, Lycopersicon esculentum, PIR:S39507
AT1G71692	0.001562901	down	0.275331837	0.010417465	down	0.737078715	AGL12	MADS-box protein identical to GB:AAC49085 GI:862650 from [Arabidopsis thaliana] (Plant Cell 7 (8), 1259-1269 (1995))
AT2G38320	0.007420145	down	0.276771023	0.29595122	down	0.91994179		hypothetical protein ; supported by cDNA: gi_13878094_gb_AF370310.1_AF370310
AT5G44530	0.004207532	down	0.277775741	0.13017692	down	0.750495853		subtilisin-like serine protease contains similarity to meiotic serine proteinase TMP GI:6468325 from [Lycopersicon esculentum]
AT3G15820 /// AT3G15830	0.011286099	down	0.278371947	0.34423757	down	0.895583876		unknown protein ; supported by full-length cDNA: Ceres: 21882.
AT2G02610	0.003824152	down	0.278484696	0.2705337	down	0.76410632		hypothetical protein
AT2G26730	0.001749253	down	0.279376978	0.13577428	down	0.927802406		putative receptor-like protein kinase ; supported by cDNA: gi_15292872_gb_AY050870.1_
AT3G27960	0.0007381	down	0.279724865	0.024510367	down	0.703970683		hypothetical protein similar to hypothetical protein GB:AAC80630 from [Arabidopsis thaliana]
AT5G63410	0.001353216	down	0.279918415	0.35366446	down	0.916998016		putative protein contains similarity to receptor-like protein kinase;supported by full-length cDNA: Ceres:31105.
AT2G30840	0.002862363	down	0.281332627	0.674941	up	1.0466207		putative dioxygenase
AT5G58300	0.007671428	down	0.282551836	0.65694016	down	0.905721625		receptor-like protein kinase
AT1G06640	0.001097499	down	0.283278142	0.25102696	down	0.887541561		oxidoreductase, putative contains Pfam profile: PF00671 Iron/Ascorbate oxidoreductase family; supported by cDNA: gi_13430545_gb_AF360185.1_AF360185
AT1G74770	0.001223511	down	0.284435359	0.003509492	up	1.9532945		hypothetical protein contains similarity to acetylcholine receptor-associated protein GI:200653 from [Mus musculus]
AT1G48480	0.000909821	down	0.286158649	0.47147623	down	0.920060879	RKL1	protein kinase, putative contains similarity to many predicted protein kinases; supported by cDNA: gi_13430527_gb_AF360176.1_AF360176
AT1G19835	0.000462561	down	0.286175945	0.09039386	down	0.864236037		unknown protein
AT5G24530	0.027840022	down	0.289480175	0.19635691	up	1.1617771		flavanone 3-hydroxylase-like protein ; supported by full-length cDNA: Ceres: 149654.
AT3G62660	0.001822558	down	0.289959391	0.96486604	down	0.995318222	GATL7	putative protein glycosyl transferase lgtC - Neisseria gonorrhoeae, EMBL:AF208062
AT1G23080	0.004408076	down	0.290098806	0.023412462	down	0.734827718	PIN7	putative auxin transport protein strongly similar to auxin transport protein GB:AAD52697; supported by cDNA: gi_15450508_gb_AY052356.1_
AT5G08330	0.001577863	down	0.290230496	0.23096915	down	0.876244256		putative protein auxin-induced basic helix-loop-helix transcription factor, Gossypium hirsutum, EMBL:AF165924; supported by cDNA: gi_15724267_gb_AF412074.1_AF412074
AT4G30290	0.005187015	down	0.291178059	0.08379417	down	0.814990213	ATXTH19	xyloglucan endo-1,4-beta-D-glucanase-like protein xyloglucan endo-1,4-beta-D-glucanase (EC 3.2.1.-) XTR-3 - Arabidopsis thaliana,PIR2:S71222; supported by cDNA: gi_15215689_gb_AY050373.1_
AT5G43180	0.001626333	down	0.291655628	0.38203815	down	0.903656565		putative protein similar to unknown protein (pir |T04500)
AT2G44380	0.001912657	down	0.291873015	0.2519521	down	0.852071885		unknown protein highly similar to GP|2435515|AF024504
AT2G35860	0.001708278	down	0.292328117	0.2890291	down	0.915718044	FLA16	unknown protein
AT5G09760	0.00093031	down	0.293300804	0.022462012	down	0.755767298		pectin methylesterase - like protein pectin methylesterase, Melandrium album, EMBL:MAPME
AT3G61220	0.00574219	down	0.294636814	0.0571395	down	0.758384471		putative protein carbonyl reductase (NADPH) - Rattus norvegicus, PIR:JC5285; supported by cDNA: gi_15028054_gb_AY045884.1_
AT1G70550	0.003751973	down	0.294815154	0.06379552	down	0.719405984		unknown protein similar to hypothetical protein GB:AAD31338 [Arabidopsis thaliana] and to putative putative carboxyl-terminal peptidase GB:AAC16072 [Arabidopsis thaliana]
AT1G08320	0.001085405	down	0.295067678	0.05228163	down	0.801877677		leucine zipper protein, putative similar to basic leucine zipper protein GI:2865394 from [Zea mays]
AT2G32990	0.006257873	down	0.295076446	0.21354029	down	0.793688651	ATGH9B8	putative glucanase
AT3G20370	0.00082477	down	0.296797246	0.042459674	down	0.749882362		unknown protein similar to GB:BAA87936 (Gene 239 (2), 309-316 (1999)), contains Pfam profile: (2) PF00917 MATH domains;supported by full-length cDNA: Ceres:40420.
AT1G30650	0.014503841	down	0.297064174	0.033198237	down	0.768937922	WRKY14	putative DNA-binding protein similar to DNA-binding protein NtWRKY3, GB:BAA77358 from [Nicotiana tabacum];supported by full-length cDNA: Ceres:153465.
AT1G16370	0.007962089	down	0.298474853	0.08514541	down	0.754224316	ATOCT6	putative transport protein may be a member of the PF|00083 sugar transporter family
AT2G38750	0.009867871	down	0.298497697	0.11371296	down	0.728274621	ANNAT4	putative annexin ;supported by full-length cDNA: Ceres:32721.
AT5G64410	0.002457038	down	0.299068256	0.047050662	down	0.786301434	ATOPT4	Isp4-like protein ; supported by full-length cDNA: Ceres: 3109.
AT4G33750	0.003254206	down	0.299184182	0.016954023	down	0.780831889		hypothetical protein
AT1G68490	0.003009056	down	0.301287628	0.19023193	down	0.899055407		unknown protein ;supported by full-length cDNA: Ceres:37060.
AT2G45470	0.000287634	down	0.301918821	0.039665874	down	0.711830408	FLA8	unknown protein ;supported by full-length cDNA: Ceres:7709.
AT3G27390	0.000851369	down	0.302872213	0.041745722	down	0.789363796		unknown protein
AT4G32410	0.00082477	down	0.303353402	0.44571772	down	0.943209097	CESA1	cellulose synthase catalytic subunit (RSW1)
AT4G35470	0.001713485	down	0.30566736	0.06779755	down	0.860925048		putative protein leucine-rich protein - Helianthus annuus (common sunflower),PIR:T12704
AT1G18880	0.000783487	down	0.305799316	0.401907	down	0.909229029		peptide transporter, putative similar to peptide transporter GI:2655098 from [Hordeum vulgare]
AT5G60520	0.000534029	down	0.30619537	0.5889334	down	0.941946517		late embryonic abundant protein - like late embryonic abundant protein EMB7, white spruce, PIR:T09288
AT5G27350	0.016824119	down	0.307049601	0.040544126	up	1.4584445	SFP1	sugar transporter-like protein putative sugar transporter (SUGTL2) - Arabidopsis thaliana, EMBL:AJ249968; supported by cDNA: gi_15294181_gb_AF410282.1_AF410282
AT5G49280	0.000881389	down	0.3071394	0.1760544	down	0.876904219		unknown protein ;supported by full-length cDNA: Ceres:147765.
AT4G18780	0.005230423	down	0.307988896	0.84176016	up	1.0636673	CESA8	cellulose synthase - like protein cellulose synthase, Gossypium hirsutum, U58283; supported by cDNA: gi_12836996_gb_AF267742.1_AF267742
AT2G06850	0.001050887	down	0.308234231	0.6606023	down	0.926164499	EXGT-A1	putative endoxyloglucan glycosyltransferase identical to GB:D16454;supported by full-length cDNA: Ceres:15276.
AT3G16340	0.000860154	down	0.309162513	0.34000313	up	1.064008	ATPDR1	putative ABC transporter similar to ABC transporter (atrB) GB:CAB52402 (Botryotinia fuckeliana); contains Pfam profile: PF00005 ABC transporter
AT1G07230	0.014832144	down	0.31073768	0.2552149	down	0.844728296		unknown protein
AT2G44010	0.001964642	down	0.311285103	0.12012886	down	0.80875428		unknown protein
AT1G03840	0.00026188	down	0.31195264	0.6486081	down	0.954685746	MGP	hypothetical protein contains Zinc finger,C2H2 type,domain
AT5G60300	0.001302828	down	0.312269155	0.26229143	up	1.1087272		receptor like protein kinase receptor like protein kinase, Arabidopsis thaliana, EMBL:AL138657; supported by cDNA: gi_15810556_gb_AY056317.1_
AT1G05700	0.02526545	down	0.313119762	0.08273333	down	0.755445059		putative light repressible receptor protein kinase Very Similar to light repressible receptor protein kinase, gi|2129635
AT5G42860	0.000652063	down	0.313703081	0.01609429	down	0.727844893		putative protein similar to unknown protein (emb|CAB53482.1)
AT1G16310	0.015758706	down	0.315632525	0.014019175	up	1.2304869		hypothetical protein predicted by genscan+
AT4G30650	0.001952861	down	0.317089474	0.9528228	up	1.0098104		low temperature and salt responsive protein homolog low temperature and salt responsive protein LTI6A - Arabidopsis thaliana,PID:g4039153
AT1G71040	0.001167618	down	0.319238323	0.029304264	up	1.3100308	LPR2	spore coat protein-like protein similar to SPORE COAT PROTEIN A GB:P07788 from [Bacillus subtilis] and BILIRUBIN OXIDASE GB:Q12737 [Myrothecium verrucaria];supported by full-length cDNA: Ceres:98973.
AT5G54380	0.005938433	down	0.320740186	0.08052491	down	0.806303552	THE1	receptor-protein kinase-like protein
AT5G54130	0.0318723	down	0.320917784	0.043432347	down	0.728448363		putative protein similar to unknown protein (gb|AAC24386.1)
AT2G05320	0.002142909	down	0.321340509	0.117536284	down	0.744586447		putative N-acetylglucosaminyltransferase
AT4G14580	0.002459923	down	0.321580271	0.869629	down	0.977130075	CIPK4	SNF1 like protein kinase ; supported by cDNA: gi_13249502_gb_AY007221.1_
AT2G48140	0.035528384	down	0.321757651	0.04023467	up	2.356584	EDA4	unknown protein ;supported by full-length cDNA: Ceres:4074.
AT3G60520	0.030088348	down	0.321855867	0.9506349	up	1.0143608		putative protein ;supported by full-length cDNA: Ceres:21518.
AT2G40150	0.001100788	down	0.322361335	0.048095644	down	0.732930202		unknown protein ; supported by cDNA: gi_13877598_gb_AF370500.1_AF370500
AT1G19370	0.000403589	down	0.32346752	0.02087873	down	0.764656652		unknown protein ; supported by cDNA: gi_15724243_gb_AF412062.1_AF412062
AT2G30520	0.004646393	down	0.324908953	0.5691496	down	0.87292423	RPT2	unknown protein
AT2G30520	0.004646393	down	0.324908953	0.17940487	down	0.815241627	RPT2	unknown protein
AT5G18690	0.010491824	down	0.325211438	0.10957088	down	0.748877657	AGP25	proline-richh protein predicted protein F24P17.17 - Arabidopsis thaliana, EMBL:AC011623
AT3G22968 /// AT3G22970	0.000628484	down	0.325372806	0.54659235	down	0.792323714		unknown protein
AT2G40160	0.006631438	down	0.32562874	0.014896033	up	1.83443		unknown protein ; supported by cDNA: gi_15983770_gb_AY056791.1_
AT5G45380	0.020197304	down	0.327038175	0.17335896	down	0.720263593		urea active transporter-like protein
AT2G38940 /// AT3G54700	0.012054575	down	0.327062028	0.12824588	down	0.760478423	ATPT2	phosphate transporter (AtPT2) identical to GB:U62331
AT4G36040	0.011542504	down	0.327668684	0.27065393	down	0.798637205		DnaJ-like protein DnaJ-like protein, Phaseolus vulgaris, PATX:G1684851
AT1G70670 /// AT1G70680	0.003787414	down	0.328316493	0.08466908	down	0.836329064		unknown protein similar to Ca+2-binding EF hand protein GB:AAB71227 [Glycine max];supported by full-length cDNA: Ceres:5143.
AT4G20270	0.007380267	down	0.32844638	0.037666675	down	0.769700879	BAM3	CLV1 receptor kinase like protein CLAVATA1 receptor kinase, Arabidopsis th., PATX:G2160756
AT5G16730	0.001479318	down	0.328615661	0.539901	down	0.872591973		putative protein predicted proteins - Arabidopsis thaliana and Oryza sativa
AT3G04350	0.00108618	down	0.329657379	0.03836413	down	0.741508449		unknown protein ; supported by cDNA: gi_15450690_gb_AY052713.1_
AT2G44300	0.007475053	down	0.330662353	0.14693189	up	1.165281		unknown protein
AT5G23220	0.003071624	down	0.332054404	0.5406593	down	0.925792372	NIC3	putative protein similar to unknown protein (pir||C72413)
AT5G57110	0.001322513	down	0.333450919	0.74814355	up	1.0746758	ACA8	Ca2+-transporting ATPase-like protein (emb|CAB79748.1)
AT5G57150	0.005494019	down	0.333628728	0.060522664	down	0.833531089		putative protein contains similarity to unknown protein (pir||T08554); supported by full-length cDNA: Ceres: 266884.
AT2G48130	0.01815654	down	0.334433978	0.007268087	up	2.2741556		unknown protein ;supported by full-length cDNA: Ceres:32411.
AT5G64740	0.001976618	down	0.335089032	0.198493	down	0.880138435	CESA6	cellulose synthase catalytic subunit
AT5G54770	0.022510247	down	0.335169863	0.24506135	down	0.890074151	THI1	thiazole biosynthetic enzyme precursor (ARA6) (sp Q38814) ; supported by cDNA: gi_1113782_gb_U17589.1_ATU17589
AT3G05900	0.002240606	down	0.33634819	0.3946149	up	1.0938914		unknown protein
AT4G01910	0.001097499	down	0.336434927	0.93825966	up	1.0061532		putative CHP-rich zinc finger protein similar to several A. thaliana CHP-rich proteins
AT1G72310	0.006868563	down	0.336657603	0.6238228	up	1.0405561	ATL3	RING-H2 zinc finger protein (ATL3) identical to RING-H2 zinc finger protein (ATL3) GB:AF132013 (Arabidopsis thaliana); supported by cDNA: gi_4928396_gb_AF132013.1_AF132013
AT5G24990 /// AT5G25020	0.004277283	down	0.337015157	0.23275805	down	0.754230801		putative protein hypothetical proteins - Arabidopsis thaliana
AT4G21230	0.005151962	down	0.337728647	0.08832339	down	0.805070463		receptor kinase - like protein SRK3 receptor kinase, Brassica oleracea, PATCHX:G624943
AT2G39360	0.000975366	down	0.337857128	0.16266063	down	0.740595803		putative protein kinase contains a protein kinase domain profile (PDOC00100)
AT1G35260	0.0092742	down	0.338395779	0.08244707	up	1.186518	MLP165	hypothetical protein similar to ripening-induced protein [Fragaria vesca] GI:2465015;supported by full-length cDNA: Ceres:16091.
AT3G12830	0.020400725	down	0.339015321	0.15908362	down	0.713433925		unknown protein contains similarity to auxin-induced protein GB:P33082 from [Glycine max]
AT3G45650 /// AT3G45660	0.001041476	down	0.339170802	0.75184464	down	0.949448873	NAXT1	putative protein different transporter proteins
AT3G14350	0.004454608	down	0.340502268	0.5391361	down	0.972350248	SRF7	putative leucine-rich repeat transmembrane protein kinase similar to leucine-rich repeat transmembrane protein kinase 1 GB:AAC27894 from [Zea mays]
AT2G29750	0.000730098	down	0.340546981	0.10570634	down	0.810021915		putative flavonol 3-O-glucosyltransferase
AT1G10380	0.001822438	down	0.3412676	0.42823693	up	1.0825671		Expressed protein ; supported by full-length cDNA: Ceres: 156978.
AT1G67080	0.002341945	down	0.34277143	0.010399547	down	0.77739763	ABA4	unknown protein ;supported by full-length cDNA: Ceres:11578.
AT2G46630	0.008594763	down	0.3446306	0.26944152	up	1.2656786		putative extensin
AT5G22850	0.002914863	down	0.344669371	0.014306657	down	0.79657879		protease-like protein
AT1G11260	0.003062179	down	0.34684397	0.28518817	down	0.863559574	STP1	glucose transporter almost identical to glucose transporter GB:P23586 from [Arabidopsis thaliana]; supported by full-length cDNA: Ceres: 33727.
AT5G65280	0.007804434	down	0.347229975	0.17158574	down	0.732590321	GCL1	G protein-coupled receptor-like protein ; supported by cDNA: gi_15451139_gb_AY054650.1_
AT4G16190	0.007680283	down	0.349393118	0.055308223	up	1.5102649		cysteine proteinase contains similarity to papain-like cysteine proteinase isoform I GI:7381219 from [Ipomoea batatas]
AT3G10760	0.001599981	down	0.350792314	0.20235485	down	0.900733332		unknown protein
AT2G05920	0.00127196	down	0.351196831	0.026149169	down	0.726953238		subtilisin-like serine protease, putative contains similarity to cucumisin-like serine protease GI:3176874 from [Arabidopsis thaliana]
AT1G01620	0.002979851	down	0.351791778	0.16490553	down	0.802574917	PIP1C	plasma membrane intrinsic protein 1c, putative similar to plasma membrane intrinsic protein 1c GI:472875 from [Arabidopsis thaliana]
AT3G26520	0.001380261	down	0.352520967	0.030462073	down	0.76608	TIP2	gamma tonoplast intrinsic protein nearly identical to gamma tonoplast intrinsic protein 2 (TIP2) GB:AF057137 [Arabidopsis thaliana]; supported by cDNA: gi_2246620_gb_AF004393.1_AF004393
AT2G26980	0.00170818	down	0.352881473	0.09632188	down	0.716915771	CIPK3	putative protein kinase contains a protein kinase domain profile (PDOC00100); supported by cDNA: gi_14194166_gb_AF367290.1_AF367290
AT4G17870	0.000263118	down	0.353697703	0.007542624	down	0.709237966		putative protein ; supported by cDNA: gi_14596204_gb_AY042890.1_
AT1G13260	0.001446127	down	0.35493186	0.033644382	down	0.709273682	RAV1	DNA-binding protein RAV1 identical to RAV1 GI:3868857 from [Arabidopsis thaliana]; supported by cDNA: gi_3868856_dbj_AB013886.1_AB013886
AT2G44940	0.016851155	down	0.355498676	0.114564106	up	1.4366784		putative AP2 domain transcription factor pFAM domain (PF00847)supported by full-length cDNA: Ceres:31044.
AT4G31920	0.003166563	down	0.356339375	0.18951865	down	0.896476989	ARR10	predicted protein RegA gene, Dictyostelium discoideum, PATCHX:G1405368

AT4G09020	0.030509556	down	0.356528736	0.0486931	down	0.768639981	ATISA3	isoamylase-like protein probable isoamylase (EC 3.2.1.68) su1 - Zea mays,PIR2:T01321
AT4G17880	0.010090958	down	0.356564662	0.7296698	down	0.922939448		bHLH protein - like bHLH protein, Arabidopsis thaliana, PATCHX:E255557
AT1G30360	0.000710608	down	0.356635696	0.04683967	down	0.75704531	ERD4	unknown protein ; supported by cDNA: gi_14334837_gb_AY035092.1_
AT2G28810	0.002718624	down	0.356900713	0.37794337	down	0.873136269		putative DOF zinc finger protein
AT4G04970	0.006749178	down	0.357408999	0.741432	down	0.947360573	ATGSL1	contains similarity to glucan synthases
AT1G13670	0.002450444	down	0.358582772	0.57748234	down	0.932485443		unknown protein
AT2G28900	0.001084539	down	0.358912114	0.21551976	down	0.835446526	OEP16	putative membrane channel protein ;supported by full-length cDNA: Ceres:10159.
AT1G22740	0.001612004	down	0.359867905	0.6214421	down	0.880659459	RAB7	RAS-related protein, RAB7 identical GB:O04157 RAS-RELATED PROTEIN RAB7 from [Arabidopsis thaliana]; supported by cDNA: gi_15718415_dbj_AB071850.1_AB071850
AT3G59100	0.007198097	down	0.360376633	0.67346597	down	0.928249644	ATGSL11	putative protein putative callose synthase catalytic subunit (CFL1) - Gossypium hirsutum, EMBL:AF085717
AT1G78060	0.001100269	down	0.361394365	0.12819974	down	0.937026655		xylosidase, putative similar to xylosidase GI:2102655 from [Aspergillus niger]
AT1G22540	0.007152865	down	0.361553997	0.246879	down	0.820258414		oligopeptide transporter, putative similar to oligopeptide transporter 1-1 GI:510238 from [Arabidopsis thaliana]
AT3G05880	0.004603774	down	0.361682254	0.24299264	up	1.125954	RCI2A	low temperature and salt responsive protein LTI6A identical to low temperature and salt responsive protein LTI6A GB:AAC97512 from [Arabidopsis thaliana]
AT2G39870	0.004718044	down	0.361994449	0.45402467	up	1.0999333		unknown protein ; supported by cDNA: gi_13877742_gb_AF370134.1_AF370134
AT1G49850	0.007954477	down	0.363352064	0.007676224	down	0.718566626		RING-H2 finger protein RHY1a identical to GI:3790593 from [Arabidopsis thaliana] (FEBS Lett. 436 (2), 283-287 (1998));supported by full-length cDNA: Ceres:22999.
AT5G42100	0.001005777	down	0.363884069	0.032825496	down	0.837588352	ATBG_PAP	beta-1,3-glucanase-like protein ;supported by full-length cDNA: Ceres:11988.
AT2G23630	0.002789792	down	0.363939995	0.029532442	down	0.77468947	SKS16	putative pectinesterase
AT3G61060	0.03607358	down	0.36395042	0.22828113	up	1.2856182	ATPP2-A13	putative protein hypothetical proteins - Arabidopsis thaliana; supported by cDNA: gi_14334587_gb_AY034967.1_
AT5G09870	0.000823975	down	0.364305759	0.54610574	down	0.954850924	CESA5	cellulose synthase catalytic subunit
AT3G22800	0.037348744	down	0.364359053	0.07749437	up	1.3791118		hypothetical protein similar to extensin-like protein GB:CAB40769 from [Arabidopsis thaliana]
AT3G16690	0.028429287	down	0.364420225	0.097073674	down	0.772094348		MtN3-like protein similar to MtN3 GB:CAA69976 from [Medicago truncatula]
AT1G65310	0.003143261	down	0.364907169	0.2901339	down	0.742153802	ATXTH17	xyloglucan endotransglycosylase, putative similar to xyloglucan endotransglycosylase 1 GI:3901012 from [Fagus sylvatica]
AT1G71980	0.003832129	down	0.365187281	0.5728267	down	0.887312785		unknown protein contains zinc finger, C3HC4 type (RING finger) domain
AT4G24340 /// AT4G24350	0.000975366	down	0.365314112	0.13731098	down	0.796344651		putative protein storage protein - Populus deltoides, PIR2:S31580;supported by full-length cDNA: Ceres:8772.
AT5G05250	0.007152865	down	0.366688626	0.16669442	up	1.3783823		unknown protein ; supported by cDNA: gi_15810044_gb_AY054291.1_
AT4G08685	0.000972307	down	0.366722164	0.089799196	down	0.82647934	SAH7	Expressed protein ; supported by full-length cDNA: Ceres: 1734.
AT3G45840	0.01163845	down	0.367978144	0.5562975	down	0.910656327		putative protein many predicted proteins, Arabidopsis thaliana
AT1G31060	0.008415733	down	0.368184134	0.5461092	up	1.1485362		unknown protein similar to EST gb|T43267
AT5G18860 /// AT5G18890	0.001110478	down	0.369224571	0.13933222	up	1.159798		putative protein
AT1G56700	0.001912657	down	0.369331209	0.08679889	down	0.738792736		unknown protein ;supported by full-length cDNA: Ceres:21415.
AT3G25290	0.004398441	down	0.369436326	0.73067784	down	0.94003445		unknown protein ; supported by cDNA: gi_16323199_gb_AY057704.1_
AT5G24410	0.008486399	down	0.370760218	0.100190684	down	0.715567961		6-phosphogluconolactonase-like protein
AT2G30520	0.005050456	down	0.371354377	0.5691496	down	0.87292423	RPT2	unknown protein
AT2G30520	0.005050456	down	0.371354377	0.17940487	down	0.815241627	RPT2	unknown protein
AT5G10430	0.004048255	down	0.372958801	0.22940886	down	0.862495603	AGP4	AtAGP4 ; supported by cDNA: gi_13926257_gb_AF372885.1_AF372885
AT5G20830	0.002293414	down	0.373013238	0.5145354	down	0.915498399	SUS1	sucrose-UDP glucosyltransferase
AT3G44510	0.015502231	down	0.373249674	0.18962978	up	1.2843169		putative protein predicted proteins, Arabidopsis thaliana
AT3G56590	0.014474782	down	0.373414459	0.055298295	down	0.779333206		putative protein hypothetical proteins - Arabidopsis thaliana
AT1G74950	0.01865371	down	0.375078832	0.11613953	down	0.730033049	JAZ2	unknown protein ;supported by full-length cDNA: Ceres:3024.
AT1G09460	0.00111891	down	0.375186022	0.19565533	down	0.850826757		unknown protein similar to beta-1,3-glucanase GB:AAD22663, location of EST 192N12T7, gb|R90355
AT1G29950 /// AT1G29951 ///	0.002843383	down	0.375284189	0.020450769	down	0.835645076	CPuORF34	unknown protein
AT1G73280	0.004223302	down	0.375621574	0.09260696	down	0.839315062	SCPL3	putative serine carboxypeptidase similar to serine carboxypeptidase I precursor GB:P07519 [Hordeum vulgare], glucose acyltransferase GB:AAD01263 [Solanum berthaultii]; contains Pfam profile: PF00450 Serine carboxypeptidase;
AT5G55550	0.002677375	down	0.375743644	0.00523089	down	0.769357003		RNA-binding protein-like ; supported by cDNA: gi_15292724_gb_AY050796.1_
AT3G04070	0.008509189	down	0.376319225	0.50630045	up	1.0906081	ANAC047	NAM-like protein (no apical meristem) similar to NAM GB:CAA63101 [Petunia x hybrida]
AT5G59150	0.000874251	down	0.376672705	0.09896758	down	0.810040812	AtRABA2d	GTP-binding protein rab11 - like GTP-binding protein rab11, Arabidopsis thaliana, PIR:T12965; supported by cDNA: gi_13877728_gb_AF370127.1_AF370127
AT1G76240	0.001578146	down	0.377177545	0.033165656	down	0.73040721		hypothetical protein predicted by genemark.hmm
AT5G20885	0.00054362	down	0.37846624	0.077113725	down	0.765791013		putative protein non-consensus donor splice site GA at exon 1; predicted proteins, Arabidopsis thaliana; supported by cDNA: gi_16209695_gb_AY057610.1_
AT1G54510	0.006724387	down	0.378702154	0.07262175	down	0.842710098	ATNEK1	hypothetical protein contains similarity to serine/threonine kinase GI:348244 from [Homo sapiens]
AT2G19130	0.031178659	down	0.380005104	0.18623856	up	1.3275622		putative receptor-like protein kinase
AT4G31470	0.005886588	down	0.380074416	0.31298608	down	0.880687613		pathogenesis-related protein homolog pathogenesis related protein-1 - Zea mays,PIR2:T02054
AT2G40480	0.005092472	down	0.380259367	0.40875846	down	0.892009177		hypothetical protein
AT4G14900	0.000897589	down	0.380292917	0.07759697	down	0.804177606		hydroxyproline-rich glycoprotein homolog ;supported by full-length cDNA: Ceres:25333.
AT3G52470	0.000628484	down	0.380397681	0.20659292	down	0.890642782		putative protein hin1 protein -Nicotiana tabacum,PID:e259431;supported by full-length cDNA: Ceres:92178.
AT1G62990	0.002382217	down	0.381814117	0.34367296	down	0.898123757	KNAT7	homeodomain-containing protein HD1, putative similar to GB:Z29073 from [Brassica napus] (Biochim. Biophys. Acta 1219 (1), 201-204 (1994)); supported by full-length cDNA: Ceres: 106887.
AT1G49740	0.005357799	down	0.381836919	0.20457664	down	0.729900209		unknown protein similar to MAP3K-like protein kinase GB:CAB16796 GI:4006878 from [Arabidopsis thaliana];supported by full-length cDNA: Ceres:17743.
AT4G12730	0.000534384	down	0.381931216	0.89399916	down	0.988130478	FLA2	putative pollen surface protein endosperm specific protein - Zea mays, PID:g2104712;supported by full-length cDNA: Ceres:4620.
AT5G01890	0.008429198	down	0.38259695	0.044967987	down	0.729147192		putative protein leucine-rich receptor-like protein (LRPKm1) - Malus domestica, EMBL:AF053127; supported by cDNA: gi_15983377_gb_AF424563.1_AF424563
AT3G06170	0.000787158	down	0.383190608	0.15786718	down	0.831306401		hypothetical protein
AT4G24060	0.001106535	down	0.383788998	0.078985095	down	0.845254307		putative protein Dof zinc finger protein - Oryza sativa,PID:d1042342;supported by full-length cDNA: Ceres:37663.
AT3G10910 /// AT3G10912	0.003972897	down	0.384254186	0.9056207	up	1.0169867		putative RING zinc finger protein contains Pfam profile: PF00097 Zinc finger, C3HC4 type (RING finger)
AT1G04200	0.004120271	down	0.386288653	0.96564287	up	1.005405		unknown protein Contains similarity to gb|Z69902 from C. elegans
AT1G30690	0.002741015	down	0.387062093	0.29381007	down	0.901748057		unknown protein Contains the PF|00650 CRAL/TRIO phosphatidyl-inositol-transfer protein domain. ESTs gb|T76582, gb|N06574 and gb|Z25700 come from this gene; supported by cDNA: gi_14334977_gb_AY035162.1_
AT4G34160	0.002632539	down	0.387567261	0.4741343	down	0.947875475	CYCD3;1	cyclin delta-3
AT4G31600	0.002211587	down	0.387589358	0.05633859	down	0.720319522		putative protein KIAA0260 gene, human, PID:g1665787; supported by cDNA: gi_14596060_gb_AY042818.1_
AT1G75540	0.001736286	down	0.388079441	0.34494612	down	0.881840436	STH2	zinc finger protein, putative similar to zinc finger protein GB:BAA33202 GI:3618312 from [Oryza sativa]
AT2G27550	0.004979617	down	0.388719606	0.8322841	up	1.0400444	ATC	similar to terminal flower ; supported by cDNA: gi_4521162_dbj_AB024715.1_AB024715
AT2G31360	0.008831373	down	0.388780495	0.007876929	down	0.824973873	ADS2	delta 9 desaturase ALMOST identical (4 aa diff't) to GP:2970036;supported by full-length cDNA: Ceres:21841.
AT5G67300	0.002632539	down	0.389090085	0.31386453	down	0.912871192	AtMYB44	myb-related protein, 33.3K (pir |S71284) ;supported by full-length cDNA: Ceres:33763.
AT5G10750	0.001611023	down	0.389694088	0.043980394	down	0.718241429		putative protein predicted proteins, Arabidopsis thaliana;supported by full-length cDNA: Ceres:2344.
AT3G16180	0.001612004	down	0.390339488	0.8973453	down	0.990840374		putative transport protein similar to transport protein GB:AAB00858 from [Arabidopsis thaliana]
AT1G03870	0.001803242	down	0.390704774	0.011256158	down	0.800982325	FLA9	unknown protein location of ESTs est VBVBD05, gb|Z30807 and VBVBD05 , gb|Z30808;supported by full-length cDNA: Ceres:39763.
AT5G39410	0.001140098	down	0.390758026	0.044141367	down	0.810386234		unknown protein ;supported by full-length cDNA: Ceres:101366.
AT4G30440	0.002177549	down	0.390966592	0.107275754	down	0.853140119	GAE1	nucleotide sugar epimerase-like protein nucleotide sugar epimerase - Vibrio vulnificus,PID:g3093975;supported by full-length cDNA: Ceres:154741.
AT5G60020	0.00697549	down	0.392450354	0.4786622	down	0.865493859	LAC17	laccase - like protein laccase LAC2-4, Liriodendron tulipifera, EMBL:LTU73106
AT5G65700	0.001440628	down	0.392877909	0.14476113	down	0.849439052	BAM1	receptor protein kinase-like protein
AT2G22770	0.010100544	down	0.393047617	0.0719886	down	0.739274021	NAI1	putative bHLH transcription factor
AT1G48300	0.002059976	down	0.393571405	0.4971858	up	1.0829668		hypothetical protein predicted by genemark.hmm
AT1G76090	0.001479318	down	0.394185309	0.07432232	down	0.770977983	SMT3	S-adenosyl-methionine-sterol-C-methyltransferase, putative almost identical to S-adenosyl-methionine-sterol-C-methyltransferase GI:2246456 from [Arabidopsis thaliana]; supported by cDNA: gi_2246455_gb_U71400.1_ATU71400
AT5G46890 /// AT5G46900	0.00346449	down	0.395060324	0.032110117	down	0.717650264		extA (emb CAA47807.1)
AT1G56210	0.001599981	down	0.395920279	0.23063146	up	1.1220136		hypothetical protein predicted by genemark.hmm;supported by full-length cDNA: Ceres:107869.
AT2G46100	0.003173786	down	0.396196231	0.061635487	down	0.763468811		unknown protein
AT5G23210	0.001790471	down	0.396996405	0.023593634	down	0.864138204	SCPL34	serine carboxypeptidase II-like protein
AT5G45460 /// AT5G45470	0.007141096	down	0.397667568	0.4567916	down	0.882678442		putative protein similar to unknown protein (pir||T04430)
AT5G06630	0.045210622	down	0.397681975	0.7975783	down	0.948874185		putative protein similar to unknown protein (gb|AAD23015.1)
AT1G20693	0.002633012	down	0.398824727	0.20067619	down	0.880911588	HMGB2	Expressed protein ; supported by full-length cDNA: Ceres: 11615.
AT3G13700 /// AT3G13710	0.034326345	down	0.399246574	0.5421832	down	0.849863389		unknown protein
AT1G11700	0.042329963	down	0.39925448	0.13291274	up	1.3128374		unknown protein ESTs gb|R65381 and gb|T44635 come from this gene
AT3G03550	0.003360657	down	0.399568035	0.85488474	up	1.0348649		expressed protein predicted by genefinder, single EST match; contains zinc finger domain, C3HC4 type (RING finger); supported by cDNA: Ceres: 152633.
AT3G45600	0.000728709	down	0.399771634	0.17362885	down	0.850332905	TET3	putative protein senescence-associated protein 5 (SA5) - Hemerocallis hybrid cultivar, EMBL:AF082030; supported by full-length cDNA: Ceres: 11427.
AT3G10340	0.005298386	down	0.399974818	0.07161653	up	1.3256371		putative phenylalanine ammonia-lyase similar to phenylalanine ammonia-lyase GB:S48726 [Petroselinum crispum]
AT5G16590	0.001057559	down	0.399989888	0.06399083	down	0.823783147		receptor-like protein kinase
AT3G52920	0.014980723	down	0.400177118	0.22599106	down	0.823876875		putative protein various predicted proteins, Arabidopsis thaliana;supported by full-length cDNA: Ceres:4829.
AT1G68070	0.001084539	down	0.400745981	0.16815832	down	0.907402563		putative RING zinc finger protein contains Pfam profile: PF00097 zinc finger, C3HC4 type (RING finger);supported by full-length cDNA: Ceres:109181.
AT5G10030	0.006050473	down	0.400810085	0.01719101	down	0.720105867	TGA4	transcription factor OBF4
AT5G01040 /// AT5G01050	0.000239694	down	0.401841398	0.6931825	up	1.0468571	LAC8	laccase -like protein lac110 laccase, Populus trichocarpa, EMBL:PTY13773
AT3G20130	0.003092247	down	0.401878896	0.03440632	down	0.85402683	CYP705A22	cytochrome P450, putative contains Pfam profile: PF00067 cytochrome P450
AT1G73590	0.010413127	down	0.402013719	0.3067403	down	0.86141711	PIN1	auxin transporter splice variant b, putative similar to GI:7109715 from [Arabidopsis thaliana]; supported by cDNA: gi_13937192_gb_AF372950.1_AF372950
AT2G28080	0.000206803	down	0.402119863	0.014865178	down	0.725753396		putative glucosyltransferase ; supported by full-length cDNA: Ceres: 13761.
AT2G44500	0.003640318	down	0.402596311	0.004097793	down	0.718181077		similar to axi 1 protein from Nicotiana tabacum
AT5G48290	0.000783487	down	0.402931374	0.12961125	down	0.759978961		ATFP4, putative
AT3G06590	0.001612004	down	0.403194754	0.20922574	down	0.758296829		unknown protein ;supported by full-length cDNA: Ceres:9391.
AT5G04770	0.006774846	down	0.403466715	0.19370764	down	0.778543648	ATCAT6	amino acid transport - like protein amino acid transport protein AAT1, Arabidopsis thaliana, PIR:S51171; supported by full-length cDNA: Ceres: 158156.
AT1G07260	0.010697598	down	0.403634144	0.0653391	down	0.761842363		UDP-glucose glucosyltransferase, putative similar to UDP-glucose glucosyltransferase GI:453245 from [Manihot esculenta]
AT3G12110	0.005773597	down	0.40462519	0.05057452	down	0.819989575	ACT11	actin 11 (ACT11) identical to actin 11 (ACT11) SP:P53496 (Plant J. 10:189-202(1996));supported by full-length cDNA: Ceres:38101.
AT5G26280	0.007252041	down	0.40465941	0.29239178	down	0.812917453		putative protein various predicted proteins, Arabidopsis thaliana;supported by full-length cDNA: Ceres:1360.
AT5G18640	0.000938459	down	0.405568804	0.20592736	up	1.2725015		triacylglycerol lipase-like protein triacylglycerol lipase (EC 3.1.1.3) precursor 1 - Rhizomucor miehei, PIR:A34959
AT5G66950	0.002784513	down	0.405582634	0.26032493	down	0.923295389		putative protein strong similarity to unknown protein (emb CAB16785.1)
AT2G27000	0.0217355	down	0.405981455	0.6578699	down	0.944577572	CYP705A8	putative cytochrome P450
AT5G44030	0.007969478	down	0.40654543	0.9778169	up	1.0048577	CESA4	cellulose synthase catalytic subunit-like protein
AT5G67360	0.000822181	down	0.406550554	0.015549773	down	0.795183605	ARA12	subtilisin-like serine protease ARA12 almost identical to cucumisin-like serine protease (ARA12) GI:3176874 from [Arabidopsis thaliana]
AT3G51670	0.002459923	down	0.407898498	0.02852598	down	0.776646971		putative protein polyphosphoinositide binding protein Ssh2 - Glycine max, PIR:T05952; supported by cDNA: gi_16930482_gb_AF419595.1_AF419595
AT2G38050	0.003337218	down	0.408622023	0.11557215	down	0.777813281	DET2	3-oxo-5-alpha-steroid 4-dehydrogenase identical to GB:U53860; contains a steroid 5-alpha reductase, C-terminal domain; supported by cDNA: gi_15028206_gb_AY045926.1_
AT3G56410	0.022866558	down	0.409252545	0.18468471	down	0.817947736		putative protein
AT4G40060	0.003774412	down	0.409500711	0.15846992	down	0.737823605	ATHB-16	homeodomain - like protein ; supported by cDNA: gi_15450981_gb_AY054571.1_
AT5G67470	0.003707468	down	0.409831094	0.26803774	down	0.887576617		formin-like protein
AT5G25820	0.002359667	down	0.410535574	0.07026826	up	1.3528305		putative protein various predicted proteins, Arabidopsis thaliana
AT2G46780	0.011383587	down	0.410641596	0.86153495	up	1.0331715		putative RNA-binding protein
AT5G43780	0.013512041	down	0.411693732	0.3903513	down	0.889955728	APS4	ATP sulfurylase precursor (gb|AAD26634.1)
AT2G38090	0.002181858	down	0.412105466	0.37625074	down	0.802212373		putative MYB family transcription factor
AT3G15530	0.002945866	down	0.412817726	0.7704981	down	0.949902611		unknown protein ;supported by full-length cDNA: Ceres:15303.
AT2G39210	0.016630841	down	0.413243854	0.7442973	down	0.961959134		nodulin-like protein ; supported by cDNA: gi_16930478_gb_AF419593.1_AF419593
AT5G01620	0.001218186	down	0.413609797	0.81399363	down	0.951953842		putative protein several hypothetical proteins - Arabidopsis thaliana;supported by full-length cDNA: Ceres:108209.
AT3G54770	0.004454608	down	0.413729994	0.17977236	down	0.852727774		RNA binding protein - like SEB4 protein, Mus musculus, EMBL:X75316; supported by full-length cDNA: Ceres: 1156.
AT5G55960	0.001041476	down	0.413814399	0.338802	down	0.918034822		putative protein similar to unknown protein (pir||T23656); supported by cDNA: gi_16648986_gb_AY059863.1_
AT1G20823	0.004507537	down	0.413936548	0.036190975	up	1.2724351		predicted protein ; supported by cDNA: gi_15027984_gb_AY045849.1_
AT1G70940	0.014697269	down	0.413960486	0.15271926	down	0.811319991	PIN3	auxin transport protein REH1, putative similar to auxin transport protein REH1 GI:3377509 from [Oryza sativa]; supported by cDNA: gi_5817300_gb_AF087818.1_AF087818
AT1G21680	0.007149316	down	0.414249298	0.080322206	up	2.42308		unknown protein ESTs gb|N96028, gb|F14286, gb|T20680, gb|F14443, gb|AA657300 and gb|N65244 come from this gene; supported by cDNA: gi_15028242_gb_AY046036.1_
AT3G02490	0.004689048	down	0.414575754	0.45955005	down	0.884727915		unknown protein
AT3G56090	0.001446554	down	0.415525081	0.023155449	down	0.701410719	ATFER3	putative protein ferritin 2 precursor - Vigna unguiculata, PIR:T08124;supported by full-length cDNA: Ceres:33282.
AT1G68050 /// AT5G23410 ///	0.007576091	down	0.415841637	0.17899874	up	1.3360612	FKF1	F-box protein FKF1/ADO3, AtFBX2a identical to FKF1 GI:6960305 and Adagio 3 GI:13487072 from [Arabidopsis thaliana]
AT5G42320	0.002359667	down	0.415911493	0.007593091	down	0.706363744		unknown protein
AT5G11650	0.011609195	down	0.415943618	0.07466894	up	1.2615062		lysophospholipase - like protein lysophospholipase homolog LPL1, Oryza sativa, EMBL:AF039531;supported by full-length cDNA: Ceres:15284.
AT1G58270	0.000938459	down	0.416733309	0.986865	up	1.0047219	ZW9	unknown protein contains Pfam profile: PF00917 MATH domain; supported by cDNA: gi_12083245_gb_AF332419.1_AF332419
AT1G10090	0.000889151	down	0.416773117	0.04075107	down	0.791331877		hypothetical protein similar to hypothetical protein GB:S51583
AT3G24240	0.004558383	down	0.418060166	0.053110093	down	0.859539696		receptor kinase, putative similar to CLV1 receptor kinase GB:AAB58929 from [Arabidopsis thaliana]
AT1G64920	0.004143002	down	0.418697349	0.4925226	up	1.1493201		anthocyanidin-3-glucoside rhamnosyltransferase, putative similar to anthocyanidin-3-glucoside rhamnosyltransferase GI:397567 from [Petunia hybrida]
AT2G20050	0.00513253	down	0.419311017	0.37037513	down	0.886965553		putative cAMP-dependent protein kinase
AT5G22940	0.001853667	down	0.419344443	0.74971426	up	1.0399761		putative protein strong similarity to unknown protein (gb|AAC98455.1)
AT2G17790	0.003756218	down	0.419472817	0.33033454	down	0.883717531		putative vacuolar sorting protein 35
AT4G37450	0.049208578	down	0.419651772	0.19519623	down	0.787428484	AGP18	putative protein probable arabinogalactan protein precursor, Lycopersicon esculentum, PIR2:S55925; supported by cDNA: gi_11935087_gb_AF305940.1_AF305940
AT3G26030	0.011352806	down	0.420297777	0.003779925	down	0.789985699	ATB' DELTA	protein phosphatase 2A regulatory subunit isoform B delta identical to GB:AAD02810 from [Arabidopsis thaliana]; supported by cDNA: gi_4106339_gb_AF062396.1_AF062396
AT2G21830	0.013790216	down	0.421292848	0.807219	down	0.960801231		hypothetical protein predicted by genscan; supported by cDNA: gi_16648990_gb_AY059865.1_
AT2G18980	0.003681218	down	0.421697162	0.05453878	down	0.823980672		peroxidase (ATP22a) identical to GB:Y08781
AT1G02430 /// AT1G02440	0.003975459	down	0.422470335	0.9914876	up	1.0023978	ATARFD1A	ADP-ribosylation factor, putative similar to ADP-ribosylation factor GI:166586 from [Arabidopsis thaliana]
AT4G37400	0.013625029	down	0.422578433	0.007051962	down	0.70153344	CYP81F3	cytochrome P450 monooxygenase -like protein cytochrome P450 monooxygenase CYP91A2, Arabidopsis thaliana, D78607;supported by full-length cDNA: Ceres:40095.
AT5G62000	0.007380267	down	0.422588273	0.8812009	up	1.040662	ARF2	auxin response factor - like protein auxin response factor 9, Arabidopsis thaliana, PIR:T08917
AT5G62000	0.007380267	down	0.422588273	0.042803843	down	0.761180524	ARF2	auxin response factor - like protein auxin response factor 9, Arabidopsis thaliana, PIR:T08917
AT2G30600	0.007569512	down	0.422760584	0.28701597	down	0.816782929		unknown protein
AT2G30600	0.007569512	down	0.422760584	0.21774538	down	0.832881703		unknown protein
AT1G20840	0.001389125	down	0.42284142	0.019457972	down	0.700389487	TMT1	putative sugar transporter protein nearly identical to Arabidopsis sugar transporter, gi|1495273
AT3G50350	0.011879887	down	0.424142676	0.1532984	down	0.778167423		hypothetical protein various predicted proteins, Arabidopsis thaliana
AT1G67870	0.007860921	down	0.424367955	0.8592478	up	1.0228224		unknown protein contains non-consensus GG donor splice site at exon2; modeled to est match;supported by full-length cDNA: Ceres:124169.
AT1G69450	0.000424695	down	0.424824447	0.12401699	down	0.735426927		unknown protein
AT2G24420	0.000960994	down	0.424824537	0.09584255	down	0.857168401		unknown protein predicted by genscan; supported by cDNA: gi_16930416_gb_AF419562.1_AF419562
AT5G53480	0.00026188	down	0.424930918	0.10047737	down	0.854249782		Expressed protein ; supported by full-length cDNA: Ceres: 2034.
AT5G53480	0.00026188	down	0.424930918	0.030843703	down	0.825732466		Expressed protein ; supported by full-length cDNA: Ceres: 2034.
AT1G74520	0.007165048	down	0.426219779	0.043980394	down	0.707606165	ATHVA22A	AtHVA22a identical to AtHVA22a GB:AF141659; homolog of HVA22 GB:A48892 (Hordeum vulgare)- (induced by abscisic acid (ABA), likely a regulatory protein J Biol Chem 1993 Nov 5;268(31):23652-60); supported by cDNA: gi_4884931_gb_AF141659.1_AF141659
AT3G21190	0.006300792	down	0.426645733	0.017363697	down	0.804127555		unknown protein ;supported by full-length cDNA: Ceres:40534.
AT3G54420	0.034394123	down	0.426902472	0.03715275	up	1.3375506	ATEP3	class IV chitinase (CHIV)
AT3G61580	0.006405892	down	0.427348418	0.58701915	down	0.978319558		delta-8 sphingolipid desaturase ;supported by full-length cDNA: Ceres:34427.
AT5G11790	0.004001373	down	0.428415644	0.021540338	down	0.800352731		putative protein SF21 protein, Helianthus annuus, EMBL: AF189148; supported by cDNA: gi_13605683_gb_AF361823.1_AF361823
AT2G45810	0.002059976	down	0.42862399	0.024163691	down	0.779314499		putative ATP-dependent RNA helicase ; supported by cDNA: gi_14532475_gb_AY039862.1_
AT3G61540	0.001940596	down	0.428892369	0.035652988	down	0.851956027		prolyl aminopeptidase-like protein prolyl aminopeptidase - Aeromonas sobria, PIR:JC4184; supported by cDNA: gi_15450666_gb_AY052701.1_
AT5G42650	0.008625654	down	0.428916173	0.07792385	down	0.750115124	AOS	allene oxide synthase (emb CAA73184.1) ; supported by cDNA: gi_6002956_gb_AF172727.1_AF172727
AT3G15810	0.000722367	down	0.429027706	0.2214815	down	0.826349441		unknown protein ; supported by full-length cDNA: Ceres: 2528.
AT3G07340	0.000975366	down	0.429624573	0.9015396	down	0.967953761		unknown protein contains helix-loop-helix DNA binding motif
AT5G19770 /// AT5G19780	0.001887413	down	0.430022595	0.024222719	down	0.778075997	TUA3	tubulin alpha-5 chain-like protein tubulin alpha-5 chain - Arabidopsis thaliana, EMBL:M84698; supported by cDNA: gi_13605804_gb_AF367301.1_AF367301
AT2G45960 /// AT5G07830	0.001611023	down	0.431210647	0.042532843	down	0.764915294	PIP1B	putative protein heparanase precursor, Homo sapiens, EMBL:AF155510
AT2G38120	0.005805201	down	0.431559666	0.03879427	down	0.74949793	AUX1	unknown protein ;supported by full-length cDNA: Ceres:31586.
AT1G58360	0.02245618	down	0.432096882	0.4632867	up	1.2310702	AAP1	amino acid permease I identical to amino acid permease I GI:22641 from [Arabidopsis thaliana]; supported by cDNA: gi_404018_gb_L16240.1_ATHAAT
AT1G54730	0.001016433	down	0.432154228	0.15973128	down	0.822800324		integral membrane protein, putative
AT3G01470 /// AT3G01472	0.007463012	down	0.432203855	0.17899874	up	1.1572471	ATHB-1	homeobox-leucine zipper protein HAT5 (HD-ZIP protein 5) (HD-ZIP protein ATHB-1) identical to homeobox-leucine zipper protein HAT5 (HD-ZIP protein 5) (HD-ZIP protein ATHB-1) GB:Q02283 [Arabidopsis thaliana]; supported by cDNA: gi_16648821_gb_AY058188.1_
AT1G26440	0.002240335	down	0.432240807	0.3356811	down	0.8402322	ATUPS5	fatty acid elongase 3-ketoacyl-CoA synthase 1 similar to putative integral membrane protein GB:AAD17424 GI:4335747 from [Arabidopsis thaliana];supported by full-length cDNA: Ceres:30412.
AT1G09090	0.008808441	down	0.432469386	0.6710089	down	0.914920198	ATRBOHB	putative respiratory burst oxidase protein B Strong similarity to Oryza NADPH oxidase (gb|X93301)
AT1G72510 /// AT2G09970	0.003774856	down	0.432654981	0.43553752	down	0.850016826		unknown protein
AT3G60320	0.001593226	down	0.432671435	0.06987526	down	0.810517009		bZIP protein ; supported by cDNA: gi_600854_gb_U17887.1_ATU17887
AT1G62800	0.002811891	down	0.433773294	0.05459442	down	0.782953474	ASP4	aspartate aminotransferase nearly identical to aspartate aminotransferase, cytoplasmic isozyme 2 SP:P46646 [Arabidopsis thaliana (Mouse-ear cress)]; supported by cDNA: gi_693693_gb_U15035.1_ATU15035
AT2G26600	0.003295129	down	0.434259439	0.8170612	up	1.0326124		putative beta-1,3-glucanase ;supported by full-length cDNA: Ceres:95083.
AT1G03380	0.001259501	down	0.434301176	0.09474235	down	0.782614256	AtATG18g	hypothetical protein predicted by genscan
AT1G22070	0.001562901	down	0.434445762	0.010576152	up	1.2275816	TGA3	transcription factor identical to transcription factor GI:304113 from [Arabidopsis thaliana];supported by full-length cDNA: Ceres:33016.
AT5G14120	0.002162813	down	0.434659392	0.012740123	down	0.721001379		nodulin-like protein
AT3G49880	0.018536128	down	0.435210307	0.56985617	down	0.942130128		putative protein transcribed gene of unknown function, Arabidopsis thaliana, EMBL:ATH132745
AT3G01720	0.002782701	down	0.435301318	0.128223	down	0.886767033		unknown protein
AT1G54090	0.006324846	down	0.435876659	0.7183307	down	0.943671226	ATEXO70D2	hypothetical protein predicted by genemark.hmm
AT3G56370	0.004825411	down	0.435886368	0.08693136	down	0.87605181		putative protein leucine-rich receptor-like protein kinase - Malus domestica, EMBL:AF053127
AT5G03510	0.009948741	down	0.437829081	0.85533065	down	0.965476771		putative protein
AT5G08130	0.00540168	down	0.438077792	0.11613953	down	0.75005211	BIM1	myc-like protein myc-like regulatory R gene product - Phyllostachys acuta, EMBL:U11448
AT4G33420	0.002958026	down	0.438081784	0.17107089	up	1.1733886		peroxidase ATP17a -like protein peroxidase ATP17a -A.thaliana,PID:e252638;supported by full-length cDNA: Ceres:32346.
AT5G13240	0.001990669	down	0.438654286	0.507761	down	0.904283083		putative protein prdeicted proteins, Schizosaccharomyces pombe
AT2G22370	0.021177657	down	0.438750516	0.21697442	down	0.850008084		hypothetical protein predicted by genefinder;supported by full-length cDNA: Ceres:26961.
AT3G24760	0.009459466	down	0.438883421	0.5677609	down	0.889318529		unknown protein
AT1G75760	0.001557723	down	0.439104157	0.82403594	up	1.025772		ER lumen protein retaining receptor, putative similar to ER LUMEN PROTEIN RETAINING RECEPTOR GB:O44017 GI:6685402 from [Entamoeba histolytica]
AT4G17220	0.039800372	down	0.439502097	0.12717004	down	0.706583849	ATMAP70-5	putative protein
AT5G15740	0.00646568	down	0.440146424	0.005686936	down	0.710976525		putative protein auxin-independent growth promoter - Nicotiana tabacum, PIR:A44226
AT1G78580	0.004250533	down	0.440216992	0.30774495	down	0.891689746	ATTPS1	trehalose-6-phosphate synthase, putative similar to trehalose-6-phosphate synthase GI:4468259 from [Pichia angusta]; supported by cDNA: gi_13878048_gb_AF370287.1_AF370287
AT2G47650 /// AT3G62830	0.003941794	down	0.440270233	0.8477106	up	1.0080637	UXS4	putative dTDP-glucose 4-6-dehydratase contains non-consensus AT donor splice site at exon 1 and non-consensus AC acceptor splice site at exon 2
AT4G11820	0.003693496	down	0.44027818	0.031960364	down	0.820095354	MVA1	hydroxymethylglutaryl-CoA synthase ;supported by full-length cDNA: Ceres:22413.
AT1G19553	0.00082477	down	0.440830419	0.9248197	up	1.00933		amino acid permease, putative contains Pfam profile: PF00324 amino acid permease
AT3G61990	0.00294077	down	0.441345921	0.48589572	up	1.079237		putative protein several O-methyltransferases - different species;supported by full-length cDNA: Ceres:40117.
AT3G16920	0.001443862	down	0.44154884	0.07045172	down	0.788109632		basic chitinase, putative similar to basic chitinase GB:CAA78843 [Lycopersicon esculentum] (Plant Mol. Biol. 22 (6), 1017-1029 (1993)); supported by cDNA: gi_15724261_gb_AF412071.1_AF412071
AT3G29250	0.016413338	down	0.442007764	0.043506384	up	1.2303332		short-chain alcohol dehydrogenase, putative similar to short-chain alcohol dehydrogenase GB:AAF04194 [Pisum sativum]; contains Pfam profile: PF00106 short chain dehydrogenase
AT3G26440	0.001209748	down	0.442085281	0.09185902	down	0.745263552		unknown protein ; supported by cDNA: gi_16612299_gb_AF439843.1_AF439843
AT1G04680	0.004572854	down	0.442584664	0.045105785	down	0.811353102		putative pectate lyase A11 similar to GB:CAB36835;supported by full-length cDNA: Ceres:37952.
AT3G49110 /// AT3G49120	0.010905291	down	0.443016651	0.28337732	down	0.862244239	ATPCA	peroxidase ;supported by full-length cDNA: Ceres:39678.
AT5G41810	0.001940596	down	0.443345106	0.12392032	down	0.897452259		unknown protein ;supported by full-length cDNA: Ceres: 126660.
AT5G12130	0.015004646	down	0.443840427	0.5068367	down	0.92426439	PDE149	putative protein contains similarity to transmembrane transport protein;supported by full-length cDNA: Ceres:249596.
AT3G61590	0.00116244	down	0.445416779	0.6556096	down	0.954609283	HS	putative protein stamina pistilloidia (Stp) - Pisum sativum, EMBL:AF004843; supported by cDNA: gi_14423513_gb_AF386994.1_AF386994
AT5G10020	0.005260802	down	0.446265465	0.8812009	up	1.0191026		receptor protein kinase -like(fragment) receptor-like protein kinase ERECTA, Arabidopsis thaliana, EMBL:AC004484
AT3G14570	0.005665364	down	0.447087027	0.09482469	up	1.1755642	ATGSL04	hypothetical protein contains similarity to glucan synthases
AT3G53668 /// AT3G53670	0.04532995	down	0.448024157	0.6502433	down	0.943877426		putative protein predicted protein, Arabidopsis thaliana
AT1G60740 /// AT1G65970	0.002283728	down	0.448204984	0.74575305	up	1.099764	TPX2	peroxiredoxin, putative similar to type 2 peroxiredoxin GI:4928472 from [Brassica rapa];supported by full-length cDNA: Ceres:10862.; supported by full-length cDNA: Ceres: 1810.
AT4G02340	0.000972307	down	0.448304767	0.91568774	down	0.990130478		coded for by A. thaliana cDNA T04215 similar to epoxide hydrolases
AT5G23050	0.008227198	down	0.448582569	0.86742955	down	0.952975609		acetyl-CoA synthetase-like protein
AT5G07250	0.004223302	down	0.448668308	0.15876238	down	0.764562176		membrane protein membrane protein - Saccharum hybrid cultivar H65-7052, EMBL:L13655; supported by cDNA: gi_16648761_gb_AY058157.1_
AT3G47400	0.011161139	down	0.449123674	0.71593213	up	1.0460565		pectinesterase-like protein pectinesterase (EC 3.1.1.11) - Citrus sinensis,PID:g2098709
AT1G12820	0.002455834	down	0.449162043	0.013695798	down	0.750942602	AFB3	transport inhibitor response 1 (TIR1), putative similar to transport inhibitor response 1 GI:2352492 from [Arabidopsis thaliana]
AT4G30670	0.004220344	down	0.44935122	0.11249143	down	0.842626734		Expressed protein ;supported by cDNA: Ceres:103916
AT1G29050	0.000652063	down	0.449765793	0.63510823	down	0.941522508		hypothetical protein similar to hypothetical protein GB:AAB67625 GI:2342727 from [Arabidopsis thaliana]; supported by full-length cDNA: Ceres: 30068.
AT2G37570	0.004431408	down	0.451061619	0.21425122	up	1.043494	SLT1	unknown protein ; supported by cDNA: gi_15451173_gb_AY054667.1_
AT5G14340	0.008233422	down	0.45186348	0.35380498	up	1.2506031	AtMYB40	MYB40 - putative transcription factor
AT5G19590	0.001259501	down	0.45196253	0.065168835	down	0.794502488		putative protein predicted proteins - Arabidopsis thaliana
AT3G28690	0.001057559	down	0.452112085	0.070784956	down	0.832900988		protein kinase, putative similar to PROTEIN KINASE APK1A GB:Q06548 from [Arabidopsis thaliana]
AT3G25540	0.003991637	down	0.45228205	0.01216687	down	0.743347412	LAG1	unknown protein ; supported by cDNA: gi_7658238_gb_AF198179.1_AF198179
AT1G07750	0.003310761	down	0.452396162	0.0794808	down	0.710516576		globulin-like protein similar to GB:7595348 from [Cucumis melo];supported by full-length cDNA: Ceres:40551.
AT3G61240	0.001898703	down	0.453287047	0.010217648	down	0.765719826		DEAD box RNA helicase RH12
AT3G22440	0.002535919	down	0.453290376	0.044967987	down	0.787446837		unknown protein ;supported by full-length cDNA: Ceres:13305.
AT4G18070	0.001131836	down	0.453483273	0.011300517	down	0.822740549		putative protein ;supported by full-length cDNA: Ceres:4345.
AT4G02600	0.005743616	down	0.453524756	0.0488685	down	0.857003484	ATMLO1	AtMlo-h1-like protein
AT1G13580	0.003468541	down	0.45396776	0.29982412	up	1.1318747	LAG13	longevity assurance protein, putative similar to longevity assurance protein GI:7658241 from [Arabidopsis thaliana]
AT1G65295	0.003774856	down	0.454026152	0.18003419	down	0.904404531		Expressed protein ; supported by full-length cDNA: Ceres: 5586.
AT4G04340	0.00178796	down	0.45413504	0.69118255	down	0.9662887		predicted protein of unknown function ; supported by cDNA: gi_15810532_gb_AY056305.1_
AT4G14940	0.006631438	down	0.454187885	0.39954135	up	1.0577794	ATAO1	amine oxidase like protein
AT4G13430	0.001759288	down	0.454569051	0.020722393	down	0.717541611		putative protein various predicted 3-isopropylmalate dehydratases/aconitate hydratases; supported by cDNA: gi_15027970_gb_AY045842.1_
AT5G35190	0.013510628	down	0.454786946	0.05320969	down	0.74304129		extensin -like protein extensin, soybean, PIR:T06782
AT3G19240	0.002061708	down	0.455611461	0.10068745	down	0.804196296		dem-like protein similar to dem GB:CAA73973 from [Lycopersicon esculentum] (Plant Cell (1998) 10, 1-12)
AT1G64560	0.044757504	down	0.455903945	0.50728863	down	0.784467296		unknown protein
AT5G57350	0.00507679	down	0.456721316	0.015549773	down	0.748339379	AHA3	plasma membrane ATPase 3 (proton pump) (sp|P20431) ; supported by cDNA: gi_15982712_gb_AY056780.1_
AT2G01830	0.008919309	down	0.456892073	0.039542343	down	0.777236364	WOL	putative histidine kinase ; supported by cDNA: gi_13359154_dbj_AB049934.1_AB049934
AT4G02680	0.004226876	down	0.45691915	0.1676469	down	0.800714686	EOL1	hypothetical protein similar to A. thaliana hypothetical protein from chromosome III, GenBank accession number 3068704
AT1G45160	0.013565453	down	0.457160788	0.1802698	down	0.885390142		hypothetical protein contains similarity to protein kinase Ukc1p GI:3005054 from [Ustilago maydis]
AT5G65790	0.044833545	down	0.457370926	0.05707689	down	0.760616321	MYB68	transcription factor-like protein ; supported by cDNA: gi_3941493_gb_AF062901.1_AF062901
AT1G31420	0.000686013	down	0.458007659	0.056914065	down	0.748125963		protein kinase, putative contains Pfam profile: PF00069: Eukaryotic protein kinase domain
AT5G55180	0.028855832	down	0.458342531	0.404292	up	1.3604344		beta-1,3-glucanase-like protein ;supported by full-length cDNA: Ceres:7119.
AT4G08410	0.019598631	down	0.458886226	0.71918875	up	1.0667412		extensin-like protein hydroxyproline-rich glycoprotein precursor - Phaseolus vulgaris,PID:g727264
AT3G52900	0.009517767	down	0.458970283	0.10303804	down	0.71800596		putative protein chromosome assembly protein homolog, Aquifex aeolicus, PIR:B70356;supported by full-length cDNA: Ceres:121911.
AT4G12420	0.001292665	down	0.459593399	0.056102026	down	0.780701009	SKU5	putative pollen-specific protein pollen-specific protein precursor - common tobacco, PIR2:S22495
AT5G60920	0.002229199	down	0.460088623	0.4238804	down	0.925970081	COB	phytochelatin synthetase - like protein putative phytochelatin synthetase, Arabidopsis thaliana, EMBL:ATH6787;supported by full-length cDNA: Ceres:17250.
AT1G17620	0.002168912	down	0.461028033	0.7784204	up	1.0332019		unknown protein ; supported by cDNA: gi_13358204_gb_AF325013.2_AF325013
AT1G04140	0.000556665	down	0.46138675	0.16360003	down	0.879801925		unknown protein Contains similarity to neural cell adhesion molecule 2, large isoform precursor gb|M76710 from Xenopus laevis, and beta transducin from S. cerevisiae gb|Q05946. ESTs gb|N65081 gb|Z30910, gb|Z34190, gb|Z34611, gb|R30101, gb|H36304, and gb|N
AT3G19990	0.010327619	down	0.461496494	0.084722154	down	0.801771659		hypothetical protein predicted by genemark.hmm
AT5G53160	0.001490481	down	0.462489126	0.03483798	down	0.736180078		putative protein similar to unknown protein (pir||T02893)
AT2G04280	0.001955964	down	0.46277676	0.004857321	down	0.760954163		unknown protein ; supported by cDNA: gi_15810336_gb_AY056207.1_
AT5G13300	0.025001392	down	0.462824458	0.74813694	down	0.959038414	SFC	putative protein GCN4-complementing protein, Arabidopsis thaliana, EMBL:AJ130878
AT5G22880	0.003968458	down	0.463468831	0.019510278	down	0.729219345	H2B	histone H2B like protein (emb|CAA69025.1)
AT4G29260	0.005270646	down	0.463648132	0.06868028	down	0.701013673		acid phosphatase-like protein acid phosphatase-1 (EC 3.1.3.-) - Lycopersicon esculentum,PIR2:T06587; supported by cDNA: gi_15293022_gb_AY050945.1_
AT3G22380	0.002229199	down	0.464009196	0.31941187	down	0.924719766	TIC	expressed protein supported by cDNA: gi_14194102
AT3G11410	0.001986831	down	0.464937548	0.32342663	down	0.843659728	AHG3	protein phosphatase 2C (PP2C) identical to protein phosphatase 2C (PP2C) GB:P49598 [Arabidopsis thaliana]
AT2G39130	0.010176925	down	0.46508474	0.26591504	down	0.831444085		hypothetical protein predicted by genscan
AT3G06380	0.01692258	down	0.465614768	0.117544256	down	0.731493097	ATTLP9	unknown protein similar to C-terminal half of tubby protein (A mutation in the tub gene causes maturity-onset obesity, insulin resistance, and sensory deficits) GB:P50586 [Mus musculus]
AT4G14760	0.008967559	down	0.466003029	0.06867841	down	0.900925981		centromere protein homolog
AT1G49660	0.000920433	down	0.466303205	0.022319306	down	0.765044095	ATCXE5	unknown protein similar to putative esterase GB:AAD17422 GI:4335745 from [Arabidopsis thaliana]
AT5G19130	0.001767955	down	0.466761516	0.0877512	down	0.870379514		GPAA1 - like protein glycosylphosphatidylinositol anchor attachment 1 (GPAA1), Homo sapiens, EMBL:AB002135
AT1G60660	0.001389125	down	0.467322694	0.067316845	down	0.748053322	B5 #5	cytochrome b5, putative similar to cytochrome b5 GI:4240120 from [Arabidopsis thaliana]; supported by cDNA: gi_15146311_gb_AY049297.1_
AT3G14240	0.003467166	down	0.467544925	0.99211603	up	1.004684		subtilisin-like serine protease contains similarity to SBT1 GI:1771160 from [Lycopersicon esculentum];supported by full-length cDNA: Ceres:106539.
AT4G27180	0.001769274	down	0.468126616	0.1805495	down	0.778077389	ATK2	kinesin-related protein katB ; supported by cDNA: gi_1438841_dbj_D21137.1_ATHKATB
AT3G13750	0.004412859	down	0.468387059	0.21774538	down	0.863967086	BGAL1	galactosidase, putative similar to BETA-GALACTOSIDASE PRECURSOR (LACTASE) GB:P48980 from [Lycopersicon esculentum]
AT5G49900	0.001005777	down	0.468674543	0.13928382	up	1.220773		putative protein strong similarity to unknown protein (pir||T04010)
AT3G47510	0.003887266	down	0.468783826	0.6330057	down	0.967374146		putative protein
AT4G20430	0.004223302	down	0.469023969	0.42020154	down	0.914752144		subtilisin-like serine protease contains similarity to meiotic serine proteinase TMP GI:6468325 from [Lycopersicon esculentum]
AT4G05590	0.015482306	down	0.469427479	0.58328044	down	0.949531634		putative protein hypothetical protein - Arabidopsis thaliana,PIR:F71409;supported by full-length cDNA: Ceres:22715.
AT3G13950	0.00330901	down	0.469437572	0.08999009	up	1.3250664		hypothetical protein predicted by genemark.hmm
AT2G03330 /// AT3G11810	0.001950325	down	0.46946814	0.81399363	down	0.978449168		unknown protein
AT1G66270 /// AT1G66280	0.02766811	down	0.469663275	0.8292858	down	0.978535625		beta-glucosidase, putative similar to GB:AAB64244 from [Arabidopsis thaliana] (Plant Mol. Biol. 34 (1), 57-68 (1997))
AT3G59370	0.004358161	down	0.469771496	0.12323602	down	0.771196609		putative protein hypothetical protein F12F1.4 - Arabidopsis thaliana, EMBL:AC002131
AT3G02910	0.004512572	down	0.469985261	0.9351975	up	1.0189239		unknown protein ;supported by full-length cDNA: Ceres:269248.
AT5G55920	0.003919134	down	0.470752766	0.31733257	down	0.863956039		nucleolar protein-like
AT1G31050	0.006460743	down	0.470853176	0.93790966	up	1.0099231		unknown protein similar to EST gb|AA395190
AT3G01930	0.011487905	down	0.470952875	0.025274463	down	0.711707807		unknown protein similar to nodule-specific protein Nlj70 GB:AAC39500 (Lotus japonicus)
AT1G10200	0.00259417	down	0.47116079	0.044580936	down	0.713094388	WLIM1	putative transcription factor similar to transcription factor SF3 (pir||S37656); similar to ESTs gb|T42207, gb|N37716, and emb|Z17491;supported by full-length cDNA: Ceres:148790.
AT5G56240	0.003832129	down	0.471394908	0.023770722	down	0.741012445		unknown protein
AT1G64890	0.016444067	down	0.472028377	0.06281277	up	1.2201276		unknown protein ; supported by cDNA: gi_15028136_gb_AY046018.1_
AT5G50200	0.027929565	down	0.472762498	0.9092647	up	1.0154798	WR3	putative protein similar to unknown protein (pir||T05562); supported by full-length cDNA: Ceres: 158397.
AT3G11490	0.001446053	down	0.472924348	0.23257245	down	0.918198605		putative rac GTPase activating protein similar to rac GTPase activating protein 1 GB:AAC62624 [Lotus japonicus]
AT1G45688	0.001135186	down	0.473403886	0.15194805	up	1.0910454		unknown protein ;supported by full-length cDNA: Ceres:8255.
AT5G15790	0.002077387	down	0.473444386	0.24444838	up	1.1107332		putative protein RING-H2 finger protein RHB1a - Arabidopsis thaliana, EMBL:AF079179
AT1G09330	0.002100152	down	0.473611616	0.044580936	down	0.782733341		unknown protein ;supported by full-length cDNA: Ceres:14886.
AT1G56680	0.0432134	down	0.473771579	0.08272612	down	0.847584334		chitinase, putative similar to chitinase GI:17798 from [Brassica napus]
AT2G24570	0.001320766	down	0.473798448	0.03851855	down	0.777812676	WRKY17	putative WRKY-type DNA binding protein ; supported by cDNA: gi_15991743_gb_AF425836.1_AF425836
AT2G13100	0.005312877	down	0.474136022	0.507761	down	0.930655465		putative membrane transporter

AT1G73630	0.010284251	down	0.474240287	0.3754357	down	0.902328694		putative calmodulin similar to calmodulin GB:P02596 [Renilla reniformis]; contains Pfam profile: PF00036 EF hand (4 copies);supported by full-length cDNA: Ceres:94584.
AT3G52500	0.005131756	down	0.474285767	0.057494733	down	0.760472351		expressed protein supported by cDNA: gi:15450891
AT5G23840	0.028410373	down	0.474442427	0.8237908	up	1.0621245		putative protein similar to unknown protein (pir||T00970); supported by full-length cDNA: Ceres: 4716.
AT4G20260	0.00128584	down	0.474698116	0.9433428	down	0.989592652		endomembrane-associated protein ; supported by full-length cDNA: Ceres: 20592.
AT5G01710 /// AT5G01712	0.003310761	down	0.475153826	0.0932056	down	0.806091797		putative protein putative protein F4P12.100 - Arabidopsis thaliana, EMBL:AL132966; supported by cDNA: gi_15810368_gb_AY056223.1_
AT1G28140	0.002001894	down	0.475343685	0.8946058	down	0.979853238		unknown protein contains similarity to cytochrome oxidase I GI:1289267 from (Xantholinus sp.);supported by full-length cDNA: Ceres:6875.
AT2G24170	0.003573971	down	0.475442356	0.062932834	down	0.764576732		putative multispanning membrane protein similar to MURA transposase of maize Mutator transposon
AT1G05650 /// AT1G05660	0.002811891	down	0.47554939	0.4304437	up	1.2341954		putative polygalacturonase similar to GB:AAC23398
AT1G63010	0.006697245	down	0.475985462	0.23426564	up	1.0768389		unknown protein ; supported by cDNA: gi_14334841_gb_AY035094.1_
AT1G72700	0.017042935	down	0.476202994	0.019610612	up	1.4565032		putative P-type transporting ATPase similar to ATPase II GB:AAD34706 [Homo sapiens]
AT3G17210	0.001026867	down	0.476351596	0.001810002	down	0.716543697		unknown protein similar to pop3 peptide GB:AAC26526 from [Populus balsamifera subsp. trichocarpa X Populus deltoides];supported by full-length cDNA: Ceres:268652.
AT1G16060	0.007034743	down	0.476356702	0.69331086	down	0.967835628		hypothetical protein contains similarity to AP2 DNA-binding domain protein GI:1732030 from [Zea mays]; supported by cDNA: gi_15028184_gb_AY045915.1_
AT5G63150	0.006017973	down	0.476374493	0.101799764	down	0.79766113		unknown protein ;supported by full-length cDNA: Ceres:16323.
AT4G27640	0.002813752	down	0.476465578	0.38628018	up	1.1180975		putative protein Ran_GTP binding protein 5, Homo sapiens, Y08890
AT5G47780	0.001563781	down	0.476616617	0.097073674	down	0.797635108	GAUT4	putative protein strong similarity to unknown protein (emb|CAB71043.1); supported by cDNA: gi_15810326_gb_AY056202.1_
AT1G23380	0.008509189	down	0.476875428	0.40924916	up	1.0099254	KNAT6	knotted-like homeobox protein, putative similar to knotted-like homeobox protein GB:AAA67882 GI:606952 from (Arabidopsis thaliana)
AT4G28890	0.009225825	down	0.476892667	0.02292448	down	0.739021503		putative protein various hypothetical and putative proteins in Arabidopsis thaliana
AT2G17150	0.0011561	down	0.477242182	0.038906638	up	1.4198371		unknown protein similar to A. thaliana hypothetical proteins T1O24.24 and T19F6.16
AT1G14670	0.005847378	down	0.47724291	0.31584463	down	0.920360049		endomembrane protein, putative similar to endomembrane protein emp70 precursor isolog GB:AAF67014 GI:7677068 [Homo sapiens]; supported by cDNA: gi_15450754_gb_AY053419.1_
AT1G68550 /// AT1G68552	0.004494373	down	0.477627886	0.026061216	down	0.812061847		putative AP2 domain transcription factor contains Pfam profile: PF00847 AP2 domain; supported by cDNA: gi_16604674_gb_AY059782.1_
AT4G40045	0.001435934	down	0.477917511	0.20704137	down	0.829097749		Expressed protein ; supported by full-length cDNA: Ceres: 22456.
AT3G06750	0.017409958	down	0.478326386	0.039319746	up	1.19701		unknown protein ; supported by cDNA: gi_14532647_gb_AY039948.1_
AT2G36410	0.001408073	down	0.478526025	0.5735958	down	0.963574662		unknown protein ;supported by full-length cDNA: Ceres:24885.
AT2G01910	0.001733911	down	0.47875597	0.030172104	down	0.750323652	ATMAP65-6	unknown protein
AT5G07110	0.002279755	down	0.478795878	0.3314562	down	0.859054056		putative protein prenylated rab acceptor 1 - Homo sapiens, EMBL:AF112202;supported by full-length cDNA: Ceres:22460.
AT4G18060	0.002934824	down	0.479094312	0.35895064	down	0.925015471		putative protein
AT3G11930	0.001805252	down	0.47944996	0.4528729	down	0.873633539		ethylene-responsive protein, putative similar to ER6 protein GB:AAD46412 GI:5669654 from (Lycopersicon esculentum); supported by full-length cDNA: Ceres: 7474.
AT3G63200	0.008425233	down	0.480794056	0.029557064	down	0.801806952	PLA IIIB	putative protein latex protein allergen Hev b 7 - Hevea brasiliensis, EMBL:AF113546; supported by cDNA: gi_15912226_gb_AY056391.1_
AT2G46520	0.002677148	down	0.481406632	0.012667717	down	0.731624804		putative cellular apoptosis susceptibility protein
AT1G68500	0.049920425	down	0.481432172	0.08889507	up	1.4273173		hypothetical protein predicted by genefinder
AT1G24170	0.002320241	down	0.481558524	0.19370764	down	0.883530375	GATL8	putative glycosyl transferase similar to GB:AAC78704
AT1G14080	0.004378399	down	0.481640862	0.78400993	up	1.0427275	FUT6	xyloglucan fucosyltransferase, putative similar to xyloglucan fucosyltransferase GI:5231145 from (Arabidopsis thaliana)
AT3G02170	0.035561226	down	0.481672506	0.8189446	up	1.0631177	LNG2	unknown protein ;supported by full-length cDNA: Ceres:22225.
AT1G07420	0.006226386	down	0.481694478	0.6080059	down	0.96590211	SMO2-2	unknown protein ;supported by full-length cDNA: Ceres:33426.
AT4G08160	0.011999346	down	0.481906995	0.21239312	down	0.896544502		putative xylan endohydrolase
AT4G02370	0.001377628	down	0.482021467	0.91726506	up	1.0088823		coded for by A. thaliana cDNA R64991 ;supported by full-length cDNA: Ceres:19543.
AT3G16760	0.001237319	down	0.482346806	0.042638727	down	0.771194408		unknown protein
AT5G61340	0.012868946	down	0.482464863	0.78283674	up	1.031622		putative protein ; supported by cDNA: gi_14596034_gb_AY042805.1_
AT3G03700 /// AT3G04440	0.023471208	down	0.482589149	0.03005456	up	1.4814023		hypothetical protein predicted by genefinder
AT1G74720	0.007028583	down	0.482714011	0.11539457	down	0.86113545		hypothetical protein contains similarity to protien kinase C (PRKC) GI:155789 from [Aplysia californica]
AT5G62900	0.003295843	down	0.482728948	0.28896046	up	1.1270925		putative protein predicted proteins, Arabidopsis thaliana; supported by cDNA: gi_13877822_gb_AF370174.1_AF370174
AT1G08880	0.006464917	down	0.483909337	0.77689093	up	1.0233555	G-H2AX	putative histone H2A Strong similarity to Picea histone H2A (gb|X67819). ESTs gb|ATTS3874,gb|T46627,gb|T14194 come from this gene;supported by full-length cDNA: Ceres:39667.
AT1G02080	0.000877385	down	0.484426981	0.41048783	down	0.910152478		unknown protein Similar to yeast general negative regulator of transcription subunit 1; Location of ESTs gb|T44328 and gb|AA395265
AT5G35160	0.004712822	down	0.485303176	0.04944753	down	0.768228999		putative protein p76, Homo sapiens, EMBL:HSU81006
AT3G14400	0.005680134	down	0.485330804	0.045015905	down	0.71781717	UBP25	ubiquitin-specific protease 25 (UBP25) similar to GI:11993490; supported by cDNA: gi_11993489_gb_AF302673.1_AF302673
AT5G20660	0.001225199	down	0.486236871	0.06052114	down	0.810590724		24 kDa vacuolar protein - like 24 kDa vacuolar protein VP24, Ipomoea batatas, EMBL:AB025531
AT5G48930	0.006076306	down	0.487859471	0.06867841	up	1.4146938		anthranilate N-benzoyltransferase ;supported by full-length cDNA: Ceres:114130.
AT1G68880	0.025569398	down	0.48848399	0.009533367	up	2.9652605	ATBZIP	putative bZIP transcription factor similar to bZIP DNA-binding protein GB:AAD55394 [Lycopersicon esculentum]; contains Pfam profile: PF00170 bZIP transcription factor; supported by cDNA: gi_15278043_gb_AF400621.1_AF400621
AT4G29270	0.01988208	down	0.488728312	0.15040794	down	0.77947268		acid phosphatase-like protein acid phosphatase-1 (EC 3.1.3.-) - Lycopersicon esculentum,PIR2:T06587;supported by full-length cDNA: Ceres:108301.
AT3G11420	0.022701401	down	0.488731561	0.4349006	up	1.1586186		unknown protein similar to hypothetical protein GB:AAC23643 [Arabidopsis thaliana]
AT3G03040	0.005490577	down	0.488758959	0.09420731	down	0.712918947		unknown protein
AT2G46920	0.003033525	down	0.488874488	0.014208565	up	1.147553	POL	unknown protein
AT3G11620	0.006071769	down	0.489232722	0.43553752	down	0.908475458	BAS1	unknown protein similar to unknown protein GB:AAF00672 (Arabidopsis thaliana);supported by full-length cDNA: Ceres:115965.
AT1G27030	0.005064013	down	0.489240285	0.39883858	down	0.866213348		unknown protein ; supported by cDNA: gi_14517457_gb_AY039564.1_
AT5G65450	0.006775771	down	0.489278992	0.1506298	down	0.865160198		putative protein contains similarity to ubiquitin carboxyl-terminal hydrolase
AT4G16120	0.018294564	down	0.489484598	0.27242666	down	0.816789934	ATSEB1	hypothetical protein ;supported by full-length cDNA: Ceres:17019.
AT3G13290 /// AT3G13300	0.007055509	down	0.489496387	0.06852717	down	0.850225182	VCR	unknown protein contains Pfam profile: PF00400 2 WD domains, G-beta repeats
AT1G13860 /// AT2G03480	0.00082477	down	0.489901561	0.9484917	down	0.994334679		expressed protein non-consensus GA donor site at exon 4
AT2G25520	0.001084539	down	0.48994695	0.8147103	up	1.0358994		putative phosphate/phosphoenolpyruvate translocator protein ; supported by cDNA: gi_15809997_gb_AY054267.1_
AT1G78670	0.003625772	down	0.490278705	0.46589005	up	1.0732995	ATGGH3	gamma glutamyl hydrolase, putative similar to gamma glutamyl hydrolase GI:1680711 from [Glycine max];supported by full-length cDNA: Ceres:41322.
AT5G56760	0.005434568	down	0.49046136	0.16810976	down	0.846335945	AtSerat1;1	serine O-acetyltransferase (EC 2.3.1.30) Sat-52 (pir||S71207) ;supported by full-length cDNA: Ceres:119783.
AT5G44670	0.007282848	down	0.490713565	0.5561327	up	1.1233833		putative protein strong similarity to unknown protein (pir||T05325); supported by cDNA: gi_13605502_gb_AF361577.1_AF361577
AT1G43190	0.001562901	down	0.490733143	0.2857171	down	0.919132177		nuclear ribonucleoprotein, putative similar to nuclear ribonucleoprotein GI:32353 from [Homo sapiens]
AT2G28890	0.003126527	down	0.490794174	0.20205002	down	0.856183956	PLL4	unknown protein
AT1G22910	0.001667279	down	0.490811518	0.29046386	up	1.0850625		putative RNA-binding protein contains Procite 'RNP1' putative RNA-binding region, similar to GB:AAC33496; supported by cDNA: gi_13605870_gb_AF367334.1_AF367334
AT2G01570	0.004908105	down	0.491126812	0.052634854	down	0.812392231	RGA1	putative RGA1, giberellin repsonse modulation protein identical to GB:Y11336, member of SCARECROW family; supported by cDNA: gi_15529229_gb_AY052239.1_
AT5G03470	0.032315984	down	0.491223144	0.9146329	down	0.985995025	ATB' ALPHA	putative protein ; supported by cDNA: gi_2160689_gb_U73526.1_ATU73526
AT3G49220	0.010038705	down	0.491459153	0.06579028	down	0.703109883		pectinesterase - like protein pectinesterase, Citrus sinensis,gb:AAB57670; supported by cDNA: gi_16648928_gb_AY059834.1_
AT4G22756	0.008607151	down	0.491657265	0.07045716	down	0.767974599	SMO1-2	Expressed protein ; supported by full-length cDNA: Ceres: 39135.
AT3G19540	0.001912657	down	0.491787155	0.22798137	down	0.917868401		unknown protein ; supported by cDNA: gi_15809985_gb_AY054261.1_
AT5G09980	0.00501733	down	0.492053387	0.9101215	up	1.0153258	PROPEP4	unknown protein ; supported by full-length cDNA: Ceres: 6477.
AT3G52190	0.012666835	down	0.492199015	0.008618869	down	0.723906274	PHF1	unknown protein contains Pfam profile: PF00400 WD domain, G-beta repeat; supported by full-length cDNA: Ceres: 124263.
AT1G22770	0.003892675	down	0.492700009	0.16667445	up	1.1194565	GI	putative gigantea protein strong similarity to GB:CAB56039 from [Arabidopsis thaliana]; supported by cDNA: gi_6002679_gb_AF105064.1_AF105064
AT5G22740	0.004856259	down	0.493231094	0.61160326	up	1.0340196	ATCSLA02	glucosyltransferase-like protein ; supported by cDNA: gi_16648763_gb_AY058158.1_
AT1G33700	0.014597782	down	0.49354517	0.20489928	down	0.866378001		hypothetical protein predicted by genemark.hmm
AT4G37470	0.001585624	down	0.493568725	0.8023847	up	1.0212563		putative protein beta-ketoadipate enol-lactone hydrolase, Acinetobacter sp., L05770; supported by cDNA: gi_15810302_gb_AY056190.1_
AT4G03390	0.002336344	down	0.494184096	0.13616902	up	1.17133	SRF3	putative LRR receptor-like protein kinase similar to Z. mays leucine-rich repeat transmembrane protein kinase LRRTPK 1, GenBank accession number AF023164
AT3G46000	0.000509715	down	0.494451439	0.35583335	down	0.91010137	ADF2	actin depolymerizing factor 2 (ADF2) ; supported by full-length cDNA: Ceres: 99657.
AT1G73170	0.002632539	down	0.495055068	0.023384077	down	0.761345108		putative ATPase contains Pfam profile: PF00004 ATPases associated with various cellular activities (AAA)
AT4G23510	0.011740177	down	0.495139463	0.096495986	down	0.735776863		putative protein various predicted proteins, Arabidopsis thaliana
AT1G49720	0.009410014	down	0.495146941	0.6556096	down	0.965144296	ABF1	abscisic acid responsive elements-binding factor identical to abscisic acid responsive elements-binding factor GB:AAF27179 GI:6739274 from [Arabidopsis thaliana]; supported by cDNA: gi_6739273_gb_AF093544.1_AF093544
AT1G21670	0.003347591	down	0.495211552	0.3067403	down	0.808601253		hypothetical protein predicted by genscan; supported by cDNA: gi_14334811_gb_AY035079.1_
AT1G44750	0.003037738	down	0.495475368	0.044967987	down	0.73143665	ATPUP11	hypothetical protein predicted by genemark.hmm
AT2G46080	0.002142909	down	0.496129028	0.11358307	down	0.825543437		unknown protein ; supported by cDNA: gi_16604341_gb_AY058069.1_
AT1G18980	0.01815654	down	0.496193994	0.9814601	down	0.997201553		germin, putative similar to germin GI:170697 from [Triticum aestivum];supported by full-length cDNA: Ceres:119925.
AT3G45930 /// AT3G46320	0.00249602	down	0.496359649	0.4919115	down	0.910265317		Histone H4 - like protein histone H4, Zea mays, PIR:HSZM4;supported by full-length cDNA: Ceres:22146.
AT4G40030	0.001999927	down	0.496839184	0.026795128	down	0.86083315		histone H3.3 ; supported by cDNA: gi_14326561_gb_AF385735.1_AF385735
AT1G31730	0.002181858	down	0.497961644	0.2884799	down	0.906050533		epsilon-adaptin, putative similar to epsilon-adaptin GI:5689377 from [Homo sapiens]
AT1G49580	0.001612004	down	0.498195288	0.022239825	down	0.713298865		CDPK-related protein kinase, putative similar to CDPK-related protein kinase GB:X83869 GI:1103385 from [Daucus carota]
AT5G16000	0.001611023	down	0.498241779	0.17053163	down	0.777372551	NIK1	receptor protein kinase-like protein probable somatic embryogenesis receptor-like kinase - Daucus carota, EMBL:U93048;supported by full-length cDNA: Ceres:40594.
AT3G24503	0.001520919	down	0.498270553	0.04893973	down	0.802460601	ALDH2C4	Expressed protein ; supported by cDNA: gi_15912240_gb_AY056398.1_
AT3G03780 /// AT5G17920	0.003224576	down	0.498831985	0.8270079	up	1.0322082	AtMS2	putative methionine synthase similar to cobalamin-independent methionine synthase GB:AAC50037 [Arabidopsis thaliana];supported by full-length cDNA: Ceres:111720.
AT3G25140	0.005564821	down	0.498866924	0.007356134	down	0.76574627	QUA1	glycosyl transferase, putative contains Pfam profile: PF01501 glycosyl transferase family 8
AT1G22610	0.007631728	down	0.499333091	0.052799337	up	1.283705		phosphoribosylanthranilate transferase, putative similar to phosphoribosylanthranilate transferase GI:1396053 from [Pisum sativum]
AT4G21960	0.009291781	down	0.49933344	0.07299163	down	0.827838075	PRXR1	peroxidase prxr1 ;supported by full-length cDNA: Ceres:20758.
AT3G07140	0.003066319	down	0.499365132	0.030838741	down	0.729418118		unknown protein ; supported by cDNA: gi_15292900_gb_AY050884.1_
AT1G51070	0.006180713	down	0.499900945	0.08719576	down	0.843793703		bHLH transcription factor, putative similar to bHLH transcription factor GI:3757520 from [Arabidopsis thaliana];supported by full-length cDNA: Ceres:16284.
AT4G13860	0.000471695	down	0.260690194				AT4G13860	RNA-binding protein like
AT2G07739	0.002951506	down	0.300636264				AT2G07739	hypothetical protein
AT1G04240	0.027633926	down	0.430179464				SHY2	putative auxin-induced protein AUX2-11 Match to Arabidopsis IAA3 (gb|U18406). EST gb|T04296 comes from this gene; supported by cDNA: gi_972910_gb_U18406.1_ATU18406
AT1G12010	0.02163214	down	0.346447923					putative amino-cyclopropane-carboxylic acid oxidase (ACC oxidase) Strong similarity to amino-cyclopropane-carboxylic acid oxidase gb|L27664 from Brassica napus. ESTs gb|Z48548 and gb|Z48549 come from this gene; supported by cDNA: gi_15450652_gb_AY052694.1_
AT1G23120	0.016287047	down	0.299652367					unknown protein similar to ripening-induced protein, gp|AJ001449|2465015 and major latex protein, gp|X91961|1107495; Location of EST gb|T88564
AT1G24530	0.007287617	down	0.121419093					hypothetical protein predicted by genefinder; supported by cDNA: gi_15028340_gb_AY045973.1_
AT1G24793 /// AT1G24800 ///	0.0008626	down	0.338642984					F5A9.18 unknown protein; similar to ESTs dbj|AV442495.1, gb|BE522756.1, gb|T42945.1, gb|BE525268.1, gb|BE523201.1, gb|BE526298.1, gb|T42945.1, gb|AA651584.1, dbj|AV552951.1, dbj|AV547151.1, dbj|AV563142.1
AT1G34040 /// AT1G34060	0.000963065	down	0.086100933					allinase, putative similar to allinase GI:1044969 from [Allium cepa]
AT1G44050	0.001993073	down	0.052862468					unknown protein similar to hypothetical protein GI:6598598 from [Arabidopsis thaliana]
AT1G48690	0.000882553	down	0.086940661					auxin-regulated protein, putative similar to auxin-regulated protein GI:18590 from [Glycine max]
AT1G51860	0.019388115	down	0.475405062					receptor-like protein kinase, putative similar to receptor-like protein kinase GI:9758831 from [Arabidopsis thaliana]
AT1G53885 /// AT1G53903	0.025457311	down	0.144427461					Expressed protein ; supported by full-length cDNA: Ceres: 17598.
AT1G62510	0.019271728	down	0.417518701					similar to 14KD proline-rich protein DC2.15 precursor (sp|P14009); similar to ESTs emb|Z17709 and emb|Z47685 similar to hybrid proline-rich protein GB:CAA59472 GI:4454097 from [Catharanthus roseus]
AT1G64628 /// AT1G64630	0.011913031	down	0.47437025				CPuORF57	protein kinase, putative similar to mitogen activated protein kinase kinase GB:AAC32599 GI:3450842 from [Oryza sativa]
AT1G72140	0.00093031	down	0.139096916					peptide transporter PTR2-B, putative similar to SP:P46032 from [Arabidopsis thaliana]
AT1G80050	0.00455277	down	0.279161527				APT2	adenine phosphoribosyltransferase almost identical to adenine phosphoribosyltransferase GI:1402894 from [Arabidopsis thaliana]
AT1G80440	0.010828464	down	0.192592811					unknown protein contains two Kelch motifs; supported by full-length cDNA: Ceres: 32885.
AT2G02020	0.002108652	down	0.128075091					putative peptide/amino acid transporter
AT2G04800	0.001730582	down	0.250966623					unknown protein
AT2G12550	0.018589519	down	0.482230947					hypothetical protein predicted by genscan
AT2G15350 /// AT2G15370	0.001057559	down	0.216452901				FUT10	unknown protein
AT2G16400	0.007380267	down	0.210564971				BLH7	putative homeodomain transcription factor ; supported by cDNA: gi_15983780_gb_AY056796.1_
AT2G19970	0.003462096	down	0.076172256					putative pathogenesis-related protein
AT2G24230	0.006359237	down	0.466504336					putative receptor-like protein kinase
AT2G26760	0.001296873	down	0.215218047				CYCB1;4	putative cyclin ; supported by cDNA: gi_15292694_gb_AY050781.1_
AT2G28410	0.010813752	down	0.404436359					unknown protein ;supported by full-length cDNA: Ceres:119200.
AT2G32450	0.008857635	down	0.417888992					putative O-GlcNAc transferase
AT2G39510	0.007568589	down	0.040776012					nodulin-like protein
AT2G44070 /// AT5G38640	0.02426759	down	0.403044859					putative translation initiation factor eIF-2B delta subunit
AT3G01860	0.005674859	down	0.3383321					hypothetical protein predicted by genscan
AT3G11880	0.003950286	down	0.431333069					unknown protein ; supported by cDNA: gi_16226859_gb_AF428353.1_AF428353
AT3G14680	0.015204976	down	0.385626616				CYP72A14	putative cytochrome P450 similar to GB:Q05047 from [Catharanthus roseus]; supported by cDNA: gi_13605896_gb_AF367347.1_AF367347
AT3G16450	0.001935031	down	0.234866715					putative lectin contains Pfam profile: PF01419 jacalin-like lectin domain; similar to jasmonate inducible protein GB:Y11483 (Brassica napus), myrosinase binding protein GB:BAA84545 (Arabidopsis thaliana); supported by full-length cDNA: Ceres: 38051.
AT3G20100	0.000823975	down	0.329840477				CYP705A19	cytochrome P450, putative contains Pfam profile: PF00067 cytochrome P450;supported by full-length cDNA: Ceres:149380.
AT3G20120	0.010171035	down	0.269464716				CYP705A21	cytochrome P450, putative contains Pfam profile: PF00067 cytochrome P450
AT3G26290	0.00053161	down	0.073583766				CYP71B26	cytochrome P450, putative contains Pfam profile: PF00067 cytochrome P450
AT3G26300	0.046919372	down	0.418874309				CYP71B34	cytochrome P450, putative contains Pfam profile: PF00067 cytochrome P450
AT3G27350	0.013835421	down	0.443918647					unknown protein ;supported by full-length cDNA: Ceres:20618.
AT3G32040	0.000722313	down	0.068336805					geranylgeranyl pyrophosphate synthase, putative similar to GB:P34802 from [Arabidopsis thaliana] (Plant Physiol. 104 (4), 1469-1470 (1994))
AT3G45780	0.004366457	down	0.164149166				PHOT1	nonphototropic hypocotyl 1 ; supported by cDNA: gi_14532875_gb_AY040062.1_
AT3G46130	0.006097663	down	0.087932272				MYB111	Myb DNA binding protein -like MYB59, Arabidopsis thaliana, EMBL:AF062894; supported by cDNA: gi_9864078_gb_AF272733.1_AF272733
AT3G46700	0.002283728	down	0.127799725					glucuronosyl transferase-like protein glucuronosyl transferase homolog, ripening-related - Lycopersicon esculentum,PIR2:S39507
AT3G46720	0.021772893	down	0.325765518					glucuronosyl transferase-like protein glucuronosyl transferase homolog, ripening-related - Lycopersicon esculentum,PIR2:S39507
AT3G48360	0.006530769	down	0.146578842				BT2	putative protein MEL-26, Caenorhabditis elegans, U67737; supported by cDNA: gi_14532781_gb_AY040015.1_
AT3G49360	0.003535302	down	0.136945955					6-phosphogluconolactonase - like protein 6-phosphogluconolactonase (6PGL), Homo sapiens, EMBL:HSA243972;supported by full-length cDNA: Ceres:108207.
AT3G52480	0.005332818	down	0.26784893					putative protein ;supported by full-length cDNA: Ceres:11782.
AT3G63110	0.001172532	down	0.099130448				ATIPT3	tRNA isopentenyl transferase -like protein tRNA isopentenyl transferase, Arabidopsis thaliana, EMBL:AF109376; supported by cDNA: gi_14278981_dbj_AB061401.1_AB061401
AT4G02540	0.02054993	down	0.477816646					putative protein similar to A. thaliana hypothetical protein F8K4.22, GenBank accession number 3367535
AT4G15330	0.002283728	down	0.222870528				CYP705A1	cytochrome P450 like protein
AT4G15340	0.002299268	down	0.087033149				ATPEN1	lupeol synthase like protein ; supported by cDNA: gi_6650207_gb_AF062513.1_AF062513
AT4G15500	0.002112881	down	0.179117898				UGT84A4	indole-3-acetate beta-glucosyltransferase like protein
AT4G16563	0.000706198	down	0.160757709					predicted protein ; supported by cDNA: gi_15809799_gb_AY054167.1_
AT4G18510	0.001083083	down	0.046342435				CLE2	CLE2, putative CLAVATA3/ESR-Related-2 (CLE2)
AT4G22460	0.00082477	down	0.34461661					extensin like protein extensin like protein, Populus nigra, gb:D83226
AT4G25835	0.001982334	down	0.273518951					putative mitochondrial protein Homo sapiens h-bcs1 (BCS1) mRNA, nuclear gene encoding mitochondrial protein which is involved in the expression of functional mitochondrial ubiquinol-cytochrome c reductase complex probably via the control of expression of
AT4G27410	0.018592509	down	0.461060278				RD26	putative protein Arabidopsis thaliana nap gene,PID:e1234813;supported by full-length cDNA: Ceres:38344.
AT4G27970	0.008410519	down	0.298755046				SLAH2	putative protein predicted protein, Arabidopsis thaliana; supported by cDNA: gi_13605876_gb_AF367337.1_AF367337
AT4G30610	0.006871474	down	0.423279742				BRS1	SERINE CARBOXYPEPTIDASE II-like protein probable SERINE CARBOXYPEPTIDASE II-2 PRECURSOR - HORDEUM VULGARE,PIR2:T05701; supported by cDNA: gi_13877870_gb_AF370198.1_AF370198
AT4G31540	0.044274993	down	0.40430043				ATEXO70G1	putative protein tomato leucine zipper-containing protein - Lycopersicon esculentum, PIR2:S21495
AT4G37150	0.004223302	down	0.089825195					hydroxynitrile lyase like protein
AT4G37890	0.000787158	down	0.177409227				EDA40	putative protein retrotransposon -like protein -Arabidopsis thaliana,PID:e1248853
AT4G39780	0.003115624	down	0.312617104					putative protein AP2 domain containing protein RAP2.4,Arabidopsis thaliana
AT5G01360	0.004358316	down	0.314106498					putative protein several hypothetical proteins - Arabidopsis thaliana;supported by full-length cDNA: Ceres:153592.
AT5G02410	0.007003678	down	0.172703211					putative protein potassium channel regulator 1, Rattus norvegicus, EMBL:U78090
AT5G02580	0.005126215	down	0.421360944					putative protein ;supported by full-length cDNA: Ceres:16476.
AT5G03140	0.004428263	down	0.239338831					receptor like protein kinase receptor like protein kinase, Arabidopsis thaliana, EMBL:ATLECGENE
AT5G05320	0.017456276	down	0.439945677					monooxygenase ;supported by full-length cDNA: Ceres:120478.
AT5G10230	0.005332818	down	0.037786844				ANN7	annexin -like protein annexin (AnnAt2), Arabidopsis thaliana, EMBL:AF083914; supported by cDNA: gi_12667521_gb_AY014799.1_
AT5G19040	0.00513253	down	0.14434351				ATIPT5	tRNA isopentenyltransferase -like protein tRNA isopentenyltransferase, Saccharomyces cerevisiae, PIR:S67176; supported by cDNA: gi_14278985_dbj_AB061403.1_AB061403
AT5G20640	0.004143002	down	0.197426245					putative protein predicted proteins, Arabidopsis thaliana
AT5G20790	0.03772381	down	0.143536287					putative protein predicted protein, Arabidopsis thaliana;supported by full-length cDNA: Ceres:267031.
AT5G26270	0.010219285	down	0.138997287					putative protein
AT5G37540	0.00927161	down	0.308590544					putative protein nucleoid DNA-binding protein cnd41, chloroplast, common tobacco, PIR:T01996; supported by cDNA: gi_15809849_gb_AY054192.1_
AT5G42600	0.007420145	down	0.103431752				MRN	cycloartenol synthase
AT5G43520	0.005265737	down	0.343881438					putative protein strong similarity to unknown protein (pir||T02393)
AT5G44920	0.000479305	down	0.247782901					putative protein contains similarity to disease resistance protein
AT5G45070	0.00376113	down	0.158843644				ATPP2-A8	putative protein contains similarity to disease resistance protein
AT5G45940	0.002125386	down	0.27335645				ATNUDT11	putative protein similar to unknown protein (gb|AAF24540.1);supported by full-length cDNA: Ceres:141753.
AT5G47940	0.007583987	down	0.389625853					unknown protein
AT5G47980	0.006513093	down	0.121677164					acetyl-CoA:benzylalcohol acetyltranferase-like protein
AT5G53110	0.002545516	down	0.251749603					putative protein similar to unknown protein (gb AAF25996.1)
AT5G56080	0.001899881	down	0.108269213					nicotianamine synthase
AT5G57785	0.02946298	down	0.245074931					Expressed protein ; supported by full-length cDNA: Ceres: 267411.
AT5G58784	0.039644577	down	0.315428776					dehydrodolichyl diphosphate synthase - like protein dehydrodolichyl diphosphate synthase, Arabidopsis thaliana, EMBL:AJ277136
AT5G64110	0.004747035	down	0.308340582					peroxidase ATP3a homolog ;supported by full-length cDNA: Ceres:2577.
AT5G67520	0.030480793	down	0.459984224					adenylylsulfate kinase-like protein ;supported by full-length cDNA: Ceres:125039.

Supplementary Table S1 As-responsive genes in *Arabidopsis* Col-0 and Ws-2 ecotypes (Col200 Specifically Up)

AGI	Col-200 q-value	Regulation	Col-200 FC	Ws-100 q-value	Regulation	Ws-100 FC	Gene Symbol	Target Description
AT4G28085	0.000722973	up	43.287064	0.2552149	up	1.2236241		Expressed protein ; supported by cDNA: gi_15028040_gb_AY045877.1_
AT3G09410	0.000734108	up	18.406157	0.030665101	down	0.79349178		putative pectinacetylesterase similar to pectinacetylesterase precursor GB:CAA67728 [Vigna radiata]
AT1G67920	0.001005777	up	13.604115	0.3267692	up	1.1327733		unknown protein ;supported by full-length cDNA: Ceres:13102.
AT5G27660	0.000860958	up	12.513727	0.05624612	down	0.734410072		serine protease-like protein serine protease OMI - Homo sapiens, EMBL:AF020760
AT3G55470	0.003838428	up	10.144508	0.08719551	up	1.3891475		elicitor responsive/phloem -like protein FIERG2 protein, Oryza sativa, PIR:T04363
AT2G24860	0.00093031	up	9.255096	0.045071676	up	1.2796516		unknown protein ; supported by full-length cDNA: Ceres: 97914.
AT1G16040	0.001215955	up	9.003719	0.9450781	up	1.0091213		unknown protein
AT4G21390	0.002497604	up	8.105813	0.019526845	down	0.576746271	B120	serine/threonine kinase - like protein serine/threonine kinase BRLK, Brassica oleracea, gb:Y12531
AT1G14040	0.000975366	up	7.7601357	0.0512573	up	1.3546817		unknown protein contains similarity to xenotropic and polytropic retrovirus receptor GB:4759334
AT1G16040	0.002767067	up	7.3882732	0.9450781	up	1.0091213		hypothetical protein contains similarity to phosphatidyl-inositol-glycan protein GI:303615 from [Homo sapiens]
AT1G53430	0.005730347	up	7.1275873	0.050474413	up	1.3559983		receptor-like serine/threonine kinase, putative similar to receptor-like serine/threonine kinase GB:AAC50043 GI:2465923 from [Arabidopsis thaliana]
AT3G07390	0.004589277	up	7.1009064	0.06455973	up	1.2715667	AIR12	unknown protein similar to unknown protein GB:AAC62613 [Arabidopsis thaliana]; supported by cDNA: gi_14532567_gb_AY039908.1_
AT1G10230	0.001887413	up	6.912358	0.056638017	up	1.2607788	ASK18	SKP1/ASK1 (At18), putative similar to Skp1 homolog Skp1a GI:3068807 [Arabidopsis thaliana];supported by full-length cDNA: Ceres:27203.
AT1G50740	0.000470407	up	6.8823934	0.1058553	up	1.3041416		unknown protein ; supported by full-length cDNA: Ceres: 34587.
AT1G76880	0.00021261	up	6.8112373	0.019091593	down	0.656850906		GT-like trihelix DNA-binding protein, putative similar to GT2 [Arabidopsis thaliana] GI:2664202, GI:416490
AT1G77000	0.001010155	up	6.54112	0.19891936	up	1.2599623	ATSKP2;2	F-box protein family, AtFBL5 contains similarity to F-box protein FBL2 GI:6063090 from [Homo sapiens];supported by full-length cDNA: Ceres:3549.
AT2G30000	0.000787158	up	6.5265207	0.11788822	up	1.1294467		unknown protein ;supported by full-length cDNA: Ceres:35872.
AT5G64905	0.00204376	up	6.1782947	0.0976535	up	1.3972206	PROPEP3	Expressed protein ; supported by full-length cDNA: Ceres: 3657.
AT4G02430	0.000920473	up	6.0035543	0.13955872	up	1.2193196		82.09 and 30.80) similar to alternative splicing factor ASF
AT5G62200	0.000878635	up	5.8481326	0.2552149	up	1.1490738		putative protein embryo-specific protein 3, Arabidopsis thaliana, PIR:T48490
AT4G33950	0.00081214	up	5.824634	0.3569852	up	1.1374819	OST1	protein kinase - like protein protein kinase, 41K, Arabidopsis thaliana,PIR2:S71172; supported by cDNA: gi_15451087_gb_AY054624.1_
AT4G40080	0.002438627	up	5.7676425	0.21527213	down	0.781114098		putative protein CLATHRIN COAT ASSEMBLY PROTEIN AP180 - Mus musculus, SWISSPROT:Q61548;supported by full-length cDNA: Ceres:8970.
AT2G35710	0.005449038	up	5.4616947	0.26342568	up	1.2940189		Expressed protein ; supported by full-length cDNA: Ceres: 9996.
AT5G16990	0.008848785	up	5.376313	0.4195566	up	1.0652765		quinone oxidoreductase - like protein probable quinone oxidoreductase (EC 1.6.5.5) P1, Arabidopsis thaliana, PIR:S57612;supported by full-length cDNA: Ceres:205489.
AT1G32120 /// AT4G26630	0.001466356	up	5.3217015	0.047050662	up	1.3123958		hypothetical protein similar to hypothetical protein GB:AAD27684 GI:4680504 from [Oryza sativa]
AT5G54490	0.004637211	up	5.2371373	0.8918943	up	1.035878	PBP1	putative protein similar to unknown protein (pir||T05752);supported by full-length cDNA: Ceres:109272.
AT5G28630	0.001005777	up	5.2336283	0.48740888	down	0.917637703		putative protein retinal glutamic acid-rich protein, bovine, PIR:A40437;supported by full-length cDNA: Ceres:24151.
AT3G46200	0.001255612	up	5.0474463	0.101799764	up	1.20215	ATNUDT9	putative protein predicted protein, Saccharomyces cerevisiae, PIR:S65000
AT3G62150	0.00227261	up	4.968078	0.3634016	up	1.1691364	PGP21	P-glycoprotein-like proetin P-glycoprotein-2 - Arabidopsis thaliana, EMBL:Y10228
AT3G04630	0.002718624	up	4.953912	0.42345765	up	1.2186316	WDL1	unknown protein ;supported by full-length cDNA: Ceres:39706.
AT5G54860	0.001514112	up	4.8214054	0.82036585	up	1.0151469		putative protein contains similarity to integral membrane protein
AT5G62540	0.000734108	up	4.8008366	0.061022207	up	1.3442428	UBC3	E2, ubiquitin-conjugating enzyme 3 (UBC3) identical to gi:431261, SP:P42746;supported by full-length cDNA: Ceres:148141.
AT4G17650	0.002892027	up	4.793883	0.07604891	up	1.3749557		putative protein ;supported by full-length cDNA: Ceres:39872.
AT5G66090	0.001140098	up	4.692794	0.016745841	up	1.3448453		unknown protein ;supported by full-length cDNA: Ceres:124621.
AT1G13690	0.001003099	up	4.6925006	0.9325804	up	1.0087868	ATE1	hypothetical protein contains similarity to peptidyl-prolyl cis-trans isomerase E GI:4406227 from [Homo sapiens]; supported by cDNA: gi_1498063_gb_U64825.1_ATU64825
AT5G59550	0.002652926	up	4.676453	0.025831759	up	1.2260379		putative protein COP1-interacting protein CIP8, Arabidopsis thaliana, EMBL:AF162150; supported by cDNA: gi_15450686_gb_AY052711.1_
AT5G01830	0.000864591	up	4.582655	0.25872257	up	1.2100445		putative protein hypothetical protein ARC1 - Brassica napus, PIR:T08872
AT5G64170	0.00127196	up	4.5327096	0.03530303	up	1.3831867		putative protein similar to unknown protein (emb|CAB77570.1)
AT3G11670	0.000924975	up	4.4755716	0.24766155	up	1.2686912	DGD1	digalactosyldiacylglycerol synthase identical to digalactosyldiacylglycerol synthase GB:AAD42378 [Arabidopsis thaliana]; supported by cDNA: gi_5354157_gb_AF149841.1_AF149841
AT4G10955 /// AT4G10960	0.003115624	up	4.4739356	0.64224285	down	0.935920955	UGE5	UDP-galactose 4-epimerase - like protein UDP-galactose 4-epimerase, Cyamopsis tetragonoloba, AJ005082;supported by full-length cDNA: Ceres:15608.
AT5G42150	0.003007015	up	4.4702625	0.7837506	down	0.965983845		putative protein contains similarity to glutathione-S-transferase/glutaredoxin;supported by full-length cDNA: Ceres:4645.
AT3G51910	0.001877999	up	4.4491777	0.29106015	up	1.2268379	AT-HSFA7A	putative heat shock transcription factor heat shock transcription factor HSF30 - Peruvian tomato, PIR2:S25480
AT5G46410	0.000534384	up	4.4077935	0.09534992	up	1.2905618		putative protein contains similarity to (pir||T14814)
AT1G12520	0.012045704	up	4.3618665	0.8350503	up	1.0651696	CCS1	unknown protein contains similarity to copper zinc superoxide dismutase GI:5689611 from (Arabidopsis thaliana); supported by cDNA: gi_15215657_gb_AY050357.1_
AT5G53070	0.001855737	up	4.3290195	0.47123975	down	0.864381484		putative protein contains similarity to ribosomal protein L9
AT2G26190	0.001523759	up	4.3175783	0.56985617	up	1.0989211		unknown protein ; supported by cDNA: gi_16930468_gb_AF419588.1_AF419588
AT5G38930 /// AT5G38940	0.033412267	up	4.3042774	0.10909683	up	1.3029916		germin - like protein probable germin type 2, Arabidopsis thaliana, PIR:H71408
AT4G21090	0.001502273	up	4.248756	0.80999976	up	1.012901		adrenodoxin - like protein adrenodoxin precursor, Homo sapiens, PIR1:AXHU
AT1G68140	0.001178052	up	4.212691	0.036193896	up	1.2083293		unknown protein
AT5G62150	0.002494718	up	4.208051	0.1338763	down	0.627292952		putative protein predicted protein, Arabidopsis thaliana
AT3G09000	0.002882038	up	4.1432543	0.0575539	up	1.3275697		hypothetical protein ; supported by cDNA: gi_16226224_gb_AF428276.1_AF428276
AT1G09420	0.004454608	up	4.141397	0.14014834	down	0.888743214	G6PD4	putative glucose-6-phosphate dehydrogenase Similar to gi|2276344, gi|2829880, gi|2352919 and others; Location of EST gb|N37552, gc exon splice site at 20574 is based on protein alignment, and is not confirmed experimentally; supported by cDNA: gi_15810386
AT4G25720	0.001030927	up	4.093123	0.71979356	down	0.954425423		glutamine cyclotransferase precursor - like protein glutamine cyclotransferase precursor, Carica papaya, AF061240;supported by full-length cDNA: Ceres:114197.
AT2G29740	0.000921073	up	4.052352	0.1750448	down	0.74591062		putative flavonol 3-O-glucosyltransferase
AT1G03370	0.003606838	up	4.036883	0.08652659	up	1.2858908		unknown protein Contains similarity to gb|AB011110 KIAA0538 protein from Homo sapiens brain and to phospholipid-binding domain C2 PF|00168. ESTs gb|AA585988 and gb|T04384 come from this gene
AT1G36640	0.013773629	up	4.0320807	0.3407676	up	1.2759185		unknown protein
AT4G02920	0.001030927	up	3.99695	0.044042446	up	1.334639		hypothetical protein ;supported by full-length cDNA: Ceres:6957.
AT1G21100	0.001822558	up	3.9925182	0.94177425	down	0.973072931		O-methyltransferase, putative similar to GB:AAF28353 from [Fragaria x ananassa]; supported by cDNA: gi_15982843_gb_AY057529.1_
AT5G60710	0.003774856	up	3.973912	0.059652805	up	1.3759475		putative protein predicted proteins
AT3G12740	0.000877574	up	3.9573195	0.015439187	up	1.3908995		unknown protein similar to unknown protein GB:AAD25612 from [Arabidopsis thaliana];supported by full-length cDNA: Ceres:37019.
AT5G44820	0.006221771	up	3.942224	0.34412163	up	1.1128645		putative protein similar to unknown protein (gb|AAC79139.1)
AT2G26140	0.001399375	up	3.8633454	0.018036846	up	1.3547832	FTSH4	FtsH protease, putative contains similarity to YME1 GI:295582, a member of the ftsH-SEC18-PAS1-CDC48 family of putative ATPase-encoding genes from [Saccharomyces cerevisiae]
AT2G46260	0.000902111	up	3.8326123	0.023593634	up	1.3854607		unknown protein ;supported by full-length cDNA: Ceres:111153.
AT3G51890	0.002224746	up	3.8104074	0.122228116	up	1.3650888		putative protein protein At2g40060 - Arabidopsis thaliana, EMBL:AF002109;supported by full-length cDNA: Ceres:93427.
AT2G25850	0.001665289	up	3.7687898	0.2474497	up	1.145908		putative poly(A) polymerase ; supported by cDNA: gi_14532699_gb_AY039974.1_
AT3G53000	0.000989057	up	3.7325165	0.33751684	up	1.2277185	ATPP2-A15	putative protein various predicted proteins, Arabidopsis thaliana
AT3G26680	0.000831371	up	3.7297282	0.093160406	up	1.3040514	SNM1	unknown protein
AT2G43140	0.005130511	up	3.7232385	0.16174762	up	1.3435532		hypothetical protein predicted by genefinder
AT5G14310	0.000722367	up	3.7209961	0.14720209	up	1.1972414	ATCXE16	putative protein esterase HDE, Petroleum-degrading bacterium HD-1, EMBL:AB029896;supported by full-length cDNA: Ceres:153235.
AT4G21590	0.004566099	up	3.691512	0.32213923	up	1.2380733	ENDO3	putative bifunctional nuclease bifunctional nuclease, Zinnia elegans, gb:U90266;supported by full-length cDNA: Ceres:17760.
AT4G20780	0.003607981	up	3.6910787	0.018669752	up	1.3612856		calcium-binding protein - like calcium-binding protein, Solanum tuberosum, gb:L02830
AT5G62280	0.007317817	up	3.680639	0.20920514	down	0.851139165		putative protein predicted proteins, Arabidopsis thaliana
AT5G22480 /// AT5G37340	0.001005777	up	3.6566267	0.033373956	up	1.3025572		zinc finger protein-like ; supported by full-length cDNA: Ceres: 16392.
AT4G37790	0.011953034	up	3.6558454	0.31187212	up	1.2033141	HAT22	homeobox protein HAT22 ;supported by full-length cDNA: Ceres:36691.
AT1G15940	0.001523759	up	3.6294117	0.1068634	up	1.3398358		hypothetical protein predicted by genemark.hmm
AT2G40090	0.00127196	up	3.6098545	0.05214198	up	1.3157113	ATATH9	putative ABC1 protein identical to EGAD|110384|118464supported by full-length cDNA: Ceres:141861.
AT2G35930	0.005773597	up	3.530346	0.03260117	up	1.3589234		unknown protein
AT1G07000	0.001083083	up	3.512786	0.0359216	up	1.3917602	ATEXO70B2	leucine zipper protein, putative similar to leucine zipper protein GI:10177020 from [Arabidopsis thaliana]
AT1G74240	0.001091397	up	3.450391	0.084737554	up	1.221371		putative mitochondrial carrier protein similar to GB:CAA21437 from [Schizosaccharomyces pombe]
AT5G44820	0.026694091	up	3.4478822	0.34412163	up	1.1128645		putative protein similar to unknown protein (pir||T04881)
AT5G18400	0.000734108	up	3.432735	0.027324757	up	1.2647977		putative protein rsc43, Dictyostelium discoideum, EMBL:AF011338;supported by full-length cDNA: Ceres:6084.
AT2G02730	0.002283728	up	3.386426	0.19243367	up	1.3930328		expressed protein ; supported by full-length cDNA: Ceres: 93828.
AT3G05170	0.004453586	up	3.3704028	0.114636585	up	1.3404534		unknown protein
AT4G02980	0.000708953	up	3.3386533	0.09128376	up	1.303929	ABP1	auxin-binding protein 1 precursor ;supported by full-length cDNA: Ceres:34126.
AT4G28400	0.003019853	up	3.3363805	0.6614436	up	1.0356891		protein phosphatase 2C-like protein protein phosphatase 2C-fission yeast, PIR2:S54297; supported by cDNA: gi_16604584_gb_AY059737.1_
AT4G33440	0.007163932	up	3.322931	0.4491684	up	1.0797155		putative protein polygalacturonase(EC 3.2.1.15) precursor - Erwinia carotovora,PID:g42330
AT5G47730	0.001418979	up	3.32004	0.034927644	up	1.3827672		putative protein strong similarity to unknown protein (pir||T05949)
AT3G13235	0.001507366	up	3.3139155	0.0706589	up	1.31536		Expressed protein ; supported by full-length cDNA: Ceres: 12734.
AT5G53360	0.000941794	up	3.286787	0.06664099	up	1.3999943		developmental protein SINA (seven in absentia)
AT1G14540	0.029231686	up	3.2177277	0.4304437	up	1.315416		anionic peroxidase, putative similar to anionic peroxidase GI:170202 from [Nicotiana sylvestris]
AT5G48970	0.001178052	up	3.216025	0.11324964	up	1.1082152		mitochondrial carrier protein-like
AT2G35720	0.002997228	up	3.2149475	0.84996533	up	1.032862		putative DnaJ protein
AT5G48655	0.001626333	up	3.213055	0.019522712	up	1.3921353		Expressed protein ; supported by full-length cDNA: Ceres: 12974.
AT5G58787	0.003153228	up	3.1807992	0.21071547	down	0.919746253		Expressed protein ; supported by full-length cDNA: Ceres: 99033.
AT1G53030	0.007996882	up	3.1764088	0.7203313	up	1.1791037		cytochrome C oxidase assembly protein, putative similar to GB:5851903 from [Rattus norvegicus];supported by full-length cDNA: Ceres:11496.
AT5G57500	0.001996296	up	3.1656392	0.22779053	down	0.786130025		putative protein similar to unknown protein (gb|AAF08572.1);supported by full-length cDNA: Ceres:118484.
AT1G03180	0.001507366	up	3.1592762	0.20245016	up	1.1464146		hypothetical protein predicted by genefinder
AT3G28850	0.007336048	up	3.1569364	0.06352676	up	1.315368		hypothetical protein
AT5G61510	0.001479318	up	3.1504626	0.32411045	up	1.1695435		quinone oxidoreductase - like protein quinone oxidoreductase homolog, Vigna unguiculata, PIR:T11672;supported by full-length cDNA: Ceres:37197.
AT2G20940	0.001046489	up	3.1444247	0.21511282	up	1.1971445		hypothetical protein predicted by genefinder;supported by full-length cDNA: Ceres:15103.
AT2G01100	0.000922613	up	3.123972	0.06867841	up	1.3153446		unknown protein ; supported by full-length cDNA: Ceres: 39741.
AT1G31930	0.006643208	up	3.1234329	0.23405086	up	1.1328875	XLG3	G-protein, putative contains Pfam profile: PF00503: G-protein alpha subunit
AT3G23030	0.003317516	up	3.1038325	0.09352766	up	1.274043	IAA2	auxin-inducible gene (IAA2) identical to auxin-inducible gene (IAA2) GB:AF027157 [Arabidopsis thaliana] (Plant Physiol. 115, 1730 (1997))
AT1G10700	0.001439311	up	3.0967388	0.028233318	up	1.3923374		phosphoribosyl diphosphate synthase identical to phosphoribosyl diphosphate synthase GI:4902470 from (Arabidopsis thaliana); supported by cDNA: gi_16604385_gb_AY058091.1_
AT5G22250	0.007475053	up	3.0859368	0.4372505	up	1.1437898		CCR4-associated factor-like protein
AT1G11660	0.001005777	up	3.0309784	0.18453911	up	1.243959		putative heat-shock protein strong similarity to gb|Z70314 heat-shock protein from Arabidopsis thaliana and is a member of the PF|00012 Hsp70 protein family
AT1G76080	0.001005777	up	3.0218022	0.9029165	down	0.976565933	ATCDSP32	chloroplast drought-induced stress protein, putative similar to chloroplast drought-induced stress protein GI:2582821 from [Solanum tuberosum];supported by full-length cDNA: Ceres:20321.
AT4G34290	0.001057559	up	3.009862	0.09783183	down	0.741848733		putative protein various predicted proteins; supported by full-length cDNA: Ceres: 17912.
AT5G17310	0.002463043	up	2.9947422	0.741748	up	1.0348336		UDP-glucose pyrophosphorylase ; supported by cDNA: gi_13430663_gb_AF360244.1_AF360244
AT1G23710	0.004253282	up	2.983323	0.16792263	up	1.1194744		unknown protein ;supported by full-length cDNA: Ceres:36437.
AT2G32250	0.00082477	up	2.975282	0.010260057	up	1.3826102	FRS2	Mutator-like transposase similar to MURA transposase of maize Mutator transposon
AT4G19200	0.002724033	up	2.972267	0.1807122	up	1.229619		putative protein glycine/proline-rich protein GPRP, Arabidopsis thaliana, PIR2:S65780; supported by full-length cDNA: Ceres: 8188.
AT1G77810	0.001599981	up	2.9577198	0.037841383	down	0.82233853		Avr9 elicitor response protein, putative similar to Avr9 elicitor response protein GI:4138265 from [Nicotiana tabacum]
AT1G78895	0.004245726	up	2.9443913	0.090668544	up	1.3407828		Expressed protein ; supported by full-length cDNA: Ceres: 13607.
AT3G62920	0.010651741	up	2.939377	0.30676126	up	1.1238593		putative protein
AT5G49448 /// AT5G49450	0.007667923	up	2.937117	0.4814161	down	0.831910697	ATBZIP1	putative protein contains similarity to bZIP transcription factor;supported by full-length cDNA: Ceres:43004.
AT1G65440	0.00135076	up	2.9182715	0.872685	down	0.971326255	GTB1	unknown protein
AT5G66070	0.006880031	up	2.9096265	0.08009926	up	1.2963097		putative protein contains similarity to unknown protein (gb|AAF18680.1)
AT1G06180	0.009472011	up	2.9043186	0.28327307	up	1.2629335	ATMYB13	MYB-related protein identical to GB:CAA90748 GI:1263093 from [Arabidopsis thaliana]
AT3G23910 /// AT3G24255	0.014131472	up	2.8852053	0.054155152	down	0.747612406		HAPp48,5 protein identical to HAPp48,5 GB:Y09562 [Arabidopsis thaliana]
AT5G60410	0.006722262	up	2.873776	0.06105071	up	1.2838757	ATSIZ1	putative protein
AT5G60410	0.006722262	up	2.873776	0.011410872	up	1.2480712	ATSIZ1	putative protein
AT4G39640	0.005670114	up	2.872218	0.4181792	up	1.210709	GGT1	putative gamma-glutamyltransferase gamma-glutamyltransferase, Arabidopsis thaliana, PIR2:S58286
AT3G57550	0.000556665	up	2.8716607	0.07232207	up	1.1394209	AGK2	guanylate kinase-like protein guanylate kinase - Mus musculus, TREMBL:MMU53514_1; supported by cDNA: gi_7861797_gb_AF204676.1_AF204676
AT3G03060	0.000667495	up	2.8676722	0.019056853	down	0.790602581		putative 26S proteosome regulatory subunit
AT2G38880	0.004732853	up	2.860384	0.19731237	up	1.241253	HAP3A	putative CCAAT-binding transcription factor subunit identical to GB:Y13723; contains a CBF/NF-Y subunit signature (PDOC00578) presernt in members of histone-like transcription factor family; supported by full-length cDNA: Ceres: 7805.
AT1G62730	0.005382929	up	2.8461769	0.8987438	down	0.975530667		unknown protein ;supported by full-length cDNA: Ceres:153154.
AT2G40140	0.002677135	up	2.809268	0.05988935	up	1.277632	CZF1	putative CCCH-type zinc finger protein also an ankyrin-repeat protein
AT2G26570	0.000975366	up	2.790529	0.6796862	up	1.0196836		unknown protein
AT5G66310	0.01253358	up	2.781958	0.4772595	up	1.2206882		kinesin heavy chain DNA binding protein-like
AT4G24020	0.005977382	up	2.7810583	0.103285484	up	1.1963758		putative protein various predicted proteins, Arabidopsis thaliana
AT1G74970	0.010424176	up	2.779885	0.79173535	down	0.95450304	RPS9	ribosomal protein S9, putative similar to ribosomal protein S9 GI:5456946 from [Arabidopsis thaliana]; supported by full-length cDNA: Ceres: 250383.
AT5G01950	0.004085849	up	2.7736115	0.5573129	up	1.0958446		putative protein receptor protein kinases
AT3G15352	0.002163567	up	2.7711873	0.0366007	up	1.3940727	ATCOX17	Expressed protein ; supported by cDNA: gi_14794881_gb_AF349684.1_AF349684
AT1G01930	0.00196319	up	2.7662811	0.08749017	up	1.2485226		hypothetical protein predicted by genemark.hmm;supported by full-length cDNA: Ceres:24737.
AT4G27070 /// AT5G54810	0.001424769	up	2.7328825	0.020342216	down	0.826050596	TSB2	tryptophan synthase beta-subunit (TSB2) ;supported by full-length cDNA: Ceres:104687.
AT5G13490	0.00183881	up	2.7160473	0.10439992	up	1.361187	AAC2	adenosine nucleotide translocator ; supported by cDNA: gi_15292846_gb_AY050857.1_
AT5G66750	0.001568571	up	2.7131832	0.096700005	down	0.738551946	DDM1	SWI2/SNF2-like protein (gb|AAD28303.1)
AT4G23270	0.00460739	up	2.6804175	0.1145055	up	1.3235388		serine/threonine kinase serine/threonine kinase, Brassica oleracea
AT4G30530	0.000822181	up	2.6760397	0.029704686	up	1.3371437		putative protein component of aniline dioxygenase (GMP synthase like protein - Acinetobacter sp.,PID:d1013698; supported by full-length cDNA: Ceres: 37987.
AT2G39730	0.014513354	up	2.6638525	0.6809582	down	0.807597881	RCA	hypothetical protein ;supported by full-length cDNA: Ceres:7114.
AT1G24807 /// AT1G24909	0.004146509	up	2.6620276	0.068037994	up	1.3729239		anthranilate synthase beta chain ;supported by full-length cDNA: Ceres:6495.
AT4G17070	0.003467166	up	2.6415262	0.0605399	up	1.2884377		hypothetical protein ; supported by cDNA: gi_15912320_gb_AY056438.1_
AT4G34460	0.001398458	up	2.6369538	0.096700005	up	1.26091	AGB1	GTP binding protein beta subunit ; supported by cDNA: gi_15028006_gb_AY045860.1_
AT1G57820	0.001664712	up	2.6352177	0.124249734	up	1.1961052	ORTH2	transcription factor, putative similar to transcription factor ICBP90 GI:6815251 from [Homo sapiens]
AT1G02170	0.00362326	up	2.634469	0.1805495	up	1.2526367	LOL3	latex-abundant protein, putative similar to latex-abundant protein GI:4235430 from (Hevea brasiliensis)
AT5G18100	0.00214195	up	2.6308188	0.059431165	up	1.3473322	CSD3	Cu/Zn superoxide dismutase-like protein
AT5G01990	0.002237805	up	2.6259816	0.023074143	up	1.3785715		putative protein hypothetical proteins - Arabidopsis thaliana
AT4G18140	0.013092554	up	2.620602	0.36099073	up	1.1635423		putative protein HYA22 protein, Homo sapiens, PIR2:JC5707
AT2G28710	0.039851286	up	2.6204646	0.17905521	up	1.282851		putative C2H2-type zinc finger protein likely a nucleic acid binding protein
AT2G38860	0.013067	up	2.6090775	0.13067558	up	1.3709996	YLS5	unknown protein ;supported by full-length cDNA: Ceres:114031.
AT4G35230	0.001532101	up	2.5930893	0.08722916	up	1.1367382		putative protein protein kinase APK1, Arabidopsis thaliana, PIR2:S28615; supported by cDNA: gi_16612248_gb_AF439824.1_AF439824
AT1G62045	0.005777321	up	2.5921035	0.7447244	down	0.934800996		Expressed protein ; supported by full-length cDNA: Ceres: 2843.
AT5G63135	0.001629261	up	2.58555	0.84931636	up	1.0225934		Expressed protein ; supported by full-length cDNA: Ceres: 28326.
AT3G09085	0.003297276	up	2.5754058	0.0794808	up	1.1478847		Expressed protein ; supported by full-length cDNA: Ceres: 20097.
AT5G02550	0.005094098	up	2.5687194	0.07262175	down	0.744768393		putative protein ;supported by full-length cDNA: Ceres:7731.
AT4G02195	0.00214195	up	2.5685678	0.39799505	up	1.1169771	SYP42	Expressed protein ; supported by cDNA: gi_5059351_gb_AF154574.1_AF154574
AT4G16760	0.003640319	up	2.5620205	0.03807527	up	1.3818592	ACX1	acyl-CoA oxidase like protein ; supported by cDNA: gi_3044213_gb_AF057044.1_AF057044
AT4G25030	0.004940283	up	2.5588307	0.020342216	up	1.3449868		putative protein ;supported by full-length cDNA: Ceres:16463.
AT3G08670	0.010778542	up	2.5559702	0.3661293	up	1.0847003		hypothetical protein
AT5G04910	0.002784958	up	2.5523396	0.3832014	up	1.2476087		putative protein similar to unknown protein (dbj|BAA90353.1)
AT5G11680	0.001030927	up	2.5476112	0.043980394	up	1.2629123		putative protein predicted proteins, Arabidopsis thaliana;supported by full-length cDNA: Ceres:403.
AT1G08570	0.00195474	up	2.5396066	0.9979526	up	1.0002891		putative thioredoxin similar to ESTs gb|T46281, gb|R83933, gb|N65879, emb|F14466, gb|N96726, gb|AA042340, and emb|Z18150; supported by cDNA: gi_14334493_gb_AY034938.1_
AT3G18690	0.010730572	up	2.5348904	0.6252886	down	0.930917367	MKS1	hypothetical protein predicted by genemark.hmm;supported by full-length cDNA: Ceres:104278.
AT3G59280	0.003277496	up	2.532047	0.13619031	up	1.220722	TXR1	putative protein CGI-136 protein - Homo sapiens, EMBL:AF151894; supported by cDNA: gi_14190486_gb_AF380643.1_AF380643
AT5G17710	0.001157079	up	2.5278666	0.070851356	up	1.3401426	EMB1241	chloroplast GrpE protein ; supported by cDNA: gi_13878046_gb_AF370286.1_AF370286
AT2G44080	0.02417135	up	2.5275488	0.4914698	down	0.909796663	ARL	unknown protein
AT5G58590	0.000822181	up	2.5268052	0.061022207	up	1.2617068	RANBP1	Ran binding protein 1 homolog - like Ran binding protein 1 homolog, Arabidopsis thaliana, EMBL:ATU62742
AT5G61910	0.00219493	up	2.5255606	0.05845174	up	1.287797		putative protein B2 protein, Daucus carota, PIR:S32124
AT4G16146	0.014487058	up	2.523796	0.31298608	up	1.2503855		Expressed protein ; supported by full-length cDNA: Ceres: 30227.
AT3G15180	0.001779904	up	2.521883	0.39930648	up	1.0746804		hypothetical protein predicted by genemark.hmm;supported by full-length cDNA: Ceres:39566.
AT5G03380	0.003184806	up	2.5177796	0.024017146	down	0.713452096		putative protein
AT3G55560	0.001686486	up	2.5009098	0.054051597	down	0.707327982	AGF2	putative protein AT-hook protein 1 (AHP1), Arabidopsis thaliana, EMBL:ATAHP1;supported by full-length cDNA: Ceres:207314.
AT5G44070	0.003838428	up	2.4907358	0.025619788	up	1.358041	CAD1	phytochelatin synthase (gb|AAD41794.1) ; supported by cDNA: gi_14532653_gb_AY039951.1_
AT2G39480	0.002435544	up	2.4895666	0.04141387	up	1.2346042	PGP6	putative ABC transporter related to multi drug resistance proteins and P-glycoproteins
AT1G02110	0.004412205	up	2.484657	0.30240706	down	0.906672484		bZIP-like protein similar to bZIP (leucine zipper) protein, GenBank Accession Number 600855
AT5G48030	0.002281992	up	2.4795363	0.5009584	down	0.833593276	GFA2	DnaJ protein-like ;supported by full-length cDNA: Ceres:14006.
AT4G32870	0.000424695	up	2.4673672	0.11543408	down	0.87190868		putative protein hypothetical protein F17H15.20 Arabidopsis thaliana chromosome II BAC F17H15, PID:g3643606
AT1G56440	0.031975083	up	2.4634464	0.24331832	up	1.2877904		hypothetical protein contains Pfam profile: PF00515: TPR Domain
AT3G49310	0.001309886	up	2.4574945	0.3111457	down	0.905952361		putative protein sugar transporter 2, Drosophila melanogaster, EMBL:AF199484
AT1G78310	0.006922244	up	2.452757	0.0087585	up	1.3316668		hypothetical protein predicted by genemark.hmm;supported by full-length cDNA: Ceres:36448.
AT3G57330	0.00218054	up	2.4449165	0.0687089	up	1.131519	ACA11	Ca2+-transporting ATPase-like protein Ca2+-transporting ATPase, calmodulin-stimulated - Brassica oleracea, PIR:T14453
AT3G27930	0.007680283	up	2.4325836	0.08468962	down	0.768688548		hypothetical protein predicted by genemark.hmm;supported by full-length cDNA: Ceres:103959.
AT5G18150	0.001452713	up	2.4198296	0.112919725	down	0.834390715		putative protein similar to unknown protein (emb|CAB87627.1)
AT1G45145	0.01083229	up	2.4194837	0.0486931	up	1.2921976	ATTRX5	thioredoxin, putative similar to thioredoxin GI:992966 from [Arabidopsis thaliana]; supported by full-length cDNA: Ceres: 3236.
AT3G17410	0.008256495	up	2.4145765	0.040636476	up	1.3019526		putative protein kinase similar to Pto kinase interactor 1 GB:AAC61805 from [Lycopersicon esculentum]
AT5G24430	0.001259501	up	2.413365	0.07939206	up	1.2736388		calcium dependent protein kinase-like protein
AT5G11070	0.012939228	up	2.405541	0.4859393	down	0.854284154		putative protein ;supported by full-length cDNA: Ceres:42742.
AT1G34300	0.004085849	up	2.4008102	0.019492567	up	1.248634		hypothetical protein contains similarity to receptor-like protein kinase GI:6979335 from [Oryza sativa]
AT5G46780	0.010775488	up	2.399152	0.81399363	up	1.0197122		putative protein similar to unknown protein (gb|AAC61815.1);supported by full-length cDNA: Ceres:254442.
AT2G39725	0.021970902	up	2.3964202	0.3492379	up	1.1614484		Expressed protein ; supported by full-length cDNA: Ceres: 5605.
AT1G03730	0.009116313	up	2.3771517	0.19235177	up	1.2387018		unknown protein similar to ESTs gb|AA605440 and gb|H37232;supported by full-length cDNA: Ceres:30716.
AT4G39520	0.000242192	up	2.372865	0.110117495	up	1.2041745		GTP-binding - like protein GTP-binding protein DRG, Xenopus laevis, SWISS-PROT:P43690; supported by cDNA: gi_14334721_gb_AY035034.1_
AT3G10640	0.000833776	up	2.372139	0.02564413	up	1.3035762	VPS60.1	unknown protein similar to hypothetical protein GB:CAA19586 [Schizosaccharomyces pombe]
AT3G47630	0.009042825	up	2.3699162	0.5426079	down	0.842969031		putative protein proline transport helper PTH1 - Candida albicans, EMBL:Y18210
AT4G27600	0.003164308	up	2.3682997	0.38967663	up	1.1327823		carbohydrate kinase - like protein phosphofructokinase, Babesia canis, AJ223322;supported by full-length cDNA: Ceres:799.
AT1G50440	0.003552384	up	2.3661268	0.8392574	up	1.029592		unknown protein ;supported by full-length cDNA: Ceres:126074.
AT4G27350	0.003468541	up	2.36481	0.101799764	up	1.2803019		putative protein ; supported by full-length cDNA: Ceres: 35749.
AT3G08710	0.001869104	up	2.3642504	0.06322342	up	1.3024608	ATH9	thioredoxin-like protein similar to thioredoxin H-type GB:P29448 [Arabidopsis thaliana]
AT5G58110	0.003022392	up	2.362732	0.009302547	up	1.3122455		putative protein predicted proteins, Homo sapiens and Drosophila melanogaster
AT5G09300	0.002309162	up	2.350839	0.2834378	up	1.1465414		branched-chain alpha keto-acid dehydrogenase E1 alpha subunit-like protein branched-chain alpha keto-acid dehydrogenase E1 alpha subunit - Arabidopsis thaliana, EMBL:AF077955;supported by full-length cDNA: Ceres:3693.
AT1G80780	0.00520125	up	2.3459654	0.010458462	up	1.1926377		CCR4-associated factor, putative¥0CCR4-associated factor, putative similar to CCR4-ASSOCIATED FACTOR 1 GB:Q60809 from [Mus musculus]¥0similar to CCR4-ASSOCIATED FACTOR 1 GB:Q60809 from [Mus musculus]
AT4G24280	0.000787158	up	2.3433337	0.005191353	up	1.1851074	CPHSC70-1	hsp 70-like protein heat shock 70 protein - Spinacia oleracea,PID:g2654208
AT2G47270	0.017456276	up	2.3384502	0.9690387	up	1.0079942		unknown protein ;supported by full-length cDNA: Ceres:20378.
AT4G28600	0.002217041	up	2.3374858	0.002705098	up	1.3984303	NPGR2	putative protein predicted protein, Arabidopsis thaliana
AT4G33940	0.000438549	up	2.336583	0.40159014	up	1.0966172		Expressed protein ; supported by full-length cDNA: Ceres: 14385.
AT1G09020	0.001578146	up	2.3173249	0.2037939	down	0.902885296	SNF4	hypothetical protein Contains similarity to Rattus AMP-activated protein kinase (gb|X95577); supported by cDNA: gi_9965728_gb_AF250335.1_AF250335
AT3G02750	0.000972307	up	2.3161552	0.53688174	down	0.966466885		putative protein phosphatase-2C (PP2C) similar to putative protein phosphatase-2C (PP2C) GB:AAC36699 [Mesembryanthemum crystallinum]
AT4G08590	0.01688927	up	2.313871	0.0238094	up	1.3449368		putative zinc finger protein
AT2G24830	0.014269949	up	2.3127112	0.060600605	up	1.2973604		hypothetical protein predicted by genscan
AT3G54850	0.001612004	up	2.3073747	0.52799046	up	1.1857433		putative protein arm repeat containing protein, Brassica napus, AF024625
AT2G33740	0.000693663	up	2.3063912	0.091011584	up	1.2898992	CUTA	putative related to microbial divalent cation tolerance proteins ; supported by cDNA: gi_12963360_gb_AF327524.1_AF327524
AT2G46230	0.002563168	up	2.305959	0.33603752	down	0.93125616		hypothetical protein predicted by genscan and genefinder; supported by cDNA: gi_13605903_gb_AF367351.1_AF367351
AT2G36835	0.00195474	up	2.3056195	0.95219934	up	1.0103945		Expressed protein ; supported by full-length cDNA: Ceres: 97088.
AT3G55950	0.010221385	up	2.303332	0.28839433	down	0.890750121		receptor kinase - like protein receptor kinase homolog CRINKLY4, maize, PIR:T04108
AT1G02090	0.004561557	up	2.3016152	0.084056914	up	1.2791157	FUS5	unknown protein similar to FUS5 protein of the COP9 complex, GB:AAC25563;supported by full-length cDNA: Ceres:40042.
AT3G13860	0.002951506	up	2.296957	0.85578454	up	1.0115584		chaperonin, putative similar to CHAPERONIN CPN60-2, MITOCHONDRIAL PRECURSOR GB:Q05046 from [Cucurbita maxima];supported by full-length cDNA: Ceres:38045.
AT2G42400	0.003143723	up	2.290031	0.104269825	up	1.1278989		hypothetical protein predicted by genefinder; supported by cDNA: gi_13605699_gb_AF361831.1_AF361831
AT5G13280	0.002677135	up	2.2892692	0.016954023	up	1.3425416	AK-LYS1	aspartate kinase ;supported by full-length cDNA: Ceres:94688.
AT5G64080	0.019047746	up	2.2881544	0.9967606	up	1.0008267		putative protein contains similarity to nonspecific lipid-transfer protein;supported by full-length cDNA: Ceres:98459.
AT3G16720	0.011755955	up	2.282733	0.7358241	up	1.0446932	ATL2	putative RING zinc finger protein similar to RING-H2 zinc finger protein ATL6 GB:AAD33584 from [Arabidopsis thaliana];supported by full-length cDNA: Ceres:4581.
AT3G21280	0.004410374	up	2.281813	0.94007885	up	1.0108334	UBP7	ubiquitin-specific protease 7 (UBP7), putative similar to GI:11993467; supported by cDNA: gi_11993466_gb_AF302661.1_AF302661
AT5G19990 /// AT5G20000	0.000826175	up	2.2795405	0.15687785	up	1.308789	RPT6A	26S proteasome AAA-ATPase subunit RPT6a - like protein 26S proteasome AAA-ATPase subunit RPT6a, Arabidopsis thaliana, EMBL:AF123395;supported by full-length cDNA: Ceres:36085.
AT1G62740	0.004520424	up	2.273812	0.01966087	up	1.1801788		TPR-repeat protein contains multiple TPR domains: Pfam profile: PF00515
AT3G12200	0.002846189	up	2.2721336	0.021848038	up	1.272598	ATNEK7	protein kinase, putative contains Pfam profile: PF00069 Eukaryotic protein kinase domain
AT5G53000	0.003912679	up	2.2710412	0.37462845	up	1.045034	TAP46	PP2A regulatory subunit ;supported by full-length cDNA: Ceres:19116.
AT5G51430	0.001259501	up	2.2693768	0.012150354	up	1.2499292		putative protein contains similarity to unknown protein (gb|AAF56975.1)
AT5G17270	0.04171297	up	2.2690601	0.878208	up	1.0219473		putative protein contains similarity to unknown protein (dbj|BAA91048.1)
AT5G40370	0.002197085	up	2.2537131	0.7274738	up	1.0467678		glutaredoxin -like protein glutaredoxin, castor bean, PIR:S54825; supported by full-length cDNA: Ceres: 23800.
AT1G48450	0.001653551	up	2.2503607	0.005592544	up	1.2557341		unknown protein
AT1G55460	0.001148884	up	2.2491415	0.37547603	up	1.1235564		hypothetical protein contains similarity to zinc finger protein rts2 GB:U16133 GI:563244 from [Saccharomyces cerevisiae]; supported by cDNA: gi_13430439_gb_AF360132.1_AF360132
AT5G09250	0.006305664	up	2.2480977	0.12789942	down	0.831548138	KIWI	putative transcriptional co-activator (KIWI) ; supported by cDNA: gi_2997683_gb_AF053302.1_AF053302
AT4G15880	0.002371014	up	2.2450528	0.67346597	down	0.944306591	ESD4	Expressed protein ;supported by cDNA: gi:14423393
AT4G14385	0.003596628	up	2.241841	0.08030652	up	1.2198542		Expressed protein ; supported by cDNA: gi_13877886_gb_AF370206.1_AF370206
AT3G12800	0.004211289	up	2.2410285	0.043980394	up	1.3491762		unknown protein similar to unknown protein GB:AAD37022 from [Arabidopsis thaliana]
AT2G40000	0.008176842	up	2.2406948	0.18731736	down	0.68782338		putative nematode-resistance protein ;supported by full-length cDNA: Ceres:35056.
AT5G42740	0.001990669	up	2.235045	0.14036568	up	1.1724744		glucose-6-phosphate isomerase, cytosolic (GPI) (phosphoglucose isomerase) (PGI) (phosphohexose isomerase) (PHI) (sp|P34795) ; supported by cDNA: gi_13937229_gb_AF372970.1_AF372970
AT1G20140 /// AT2G25700	0.001585624	up	2.2320843	0.9559815	up	1.0093768	ASK4	SKP1/ASK1 (At3), putative similar to fimbriata-associated protein fap1 GI:2673868 from [Antirrhinum majus]
AT5G60750	0.001415091	up	2.2284458	0.009533367	up	1.3628805		putative protein predicted protein, Synechocystis sp.;supported by full-length cDNA: Ceres:25855.
AT5G45110	0.017536307	up	2.2223337	0.086683616	up	1.3782494	NPR3	regulatory protein NPR1-like; transcription factor inhibitor I kappa B-like
AT1G55830	0.008584567	up	2.220633	0.22064036	up	1.2014263		hypothetical protein predicted by genemark.hmm
AT5G47910	0.011985579	up	2.2205114	0.09733662	up	1.1499012	RBOHD	respiratory burst oxidase protein ; supported by cDNA: gi_3242788_gb_AF055357.1_AF055357
AT1G22510	0.001934244	up	2.2203252	0.34271315	up	1.2957433		hypothetical protein predicted by genscan+;supported by full-length cDNA: Ceres:1981.
AT3G21650	0.012971971	up	2.2197218	0.2538391	up	1.1417214		B regulatory subunit of PP2A, putative similar to B' regulatory subunit of PP2A GB:AAB58902 [Arabidopsis thaliana]
AT1G13960	0.00564791	up	2.217946	0.005459929	up	1.2262108	WRKY4	putative DNA-binding protein similar to WKRY DNA-binding protein GB:AAD17441; supported by cDNA: gi_15010749_gb_AY045676.1_
AT5G25040 /// AT5G25050	0.006108936	up	2.2159746	0.22593026	up	1.1104244		putative membrane protein hypothetical protein F19K16.31 - Arabidopsis thaliana, EMBL:AC011717
AT2G34730	0.002863155	up	2.2133675	0.30934545	up	1.140488		putative myosin heavy chain
AT2G18510	0.004454608	up	2.2056522	0.29886428	up	1.1516553	EMB2444	putative spliceosome associated protein ;supported by full-length cDNA: Ceres:38968.
AT1G08315	0.000470407	up	2.2056308	0.7389932	up	1.0354644		Expressed protein ; supported by full-length cDNA: Ceres: 40238.
AT2G14750	0.001294094	up	2.2028043	0.9525215	down	0.996128844	APK	putative adenosine phosphosulfate kinase identical to GB:U05238;supported by full-length cDNA: Ceres:14216.
AT1G36060	0.005041556	up	2.1951423	0.030512834	down	0.745119171		AP2 domain transcription factor, putative similar to AP2 domain transcription factor GI:4567204 from [Arabidopsis thaliana]
AT2G18210	0.012783155	up	2.1921146	0.9049837	up	1.0177968		unknown protein
AT4G10330	0.007053774	up	2.1908214	0.17882377	up	1.1349063		hypothetical protein
AT3G51790	0.002667186	up	2.190802	0.018669752	down	0.882831254	ATG1	putative transmembrane protein G1p Bradyrhizobium japonicum CycJ: PIR Accession Number S54745; supported by cDNA: gi_1657614_gb_U72502.1_ATU72502
AT5G47840	0.002991698	up	2.1897678	0.03178543	up	1.242138	AMK2	putative protein contains similarity to adenylate kinase; supported by cDNA: gi_15146327_gb_AY049305.1_
AT1G29120	0.009341002	up	2.1894193	0.39492494	up	1.1333321		hypothetical protein predicted by genemark.hmm
AT3G15190	0.002956729	up	2.1890297	0.18406259	down	0.826549975		30S ribosomal protein S20 contains Pfam profile: PF01649 ribosomal protein S20;supported by full-length cDNA: Ceres:24271.
AT1G72770	0.001759288	up	2.1867292	0.038224865	up	1.2003968	HAB1	protein phosphatase 2C (AtP2C-HA) identical to protein phosphatase 2C (AtP2C-HA) GB:AJ003119 [Arabidopsis thaliana] (Plant Mol. Biol. 38 (5), 879-883 (1998))
AT3G09480	0.002677375	up	2.1862123	0.28406113	down	0.834163674		putative histone H2B similar to histone H2B-3 GB:CAA12231 from [Lycopersicon esculentum]
AT4G38060	0.004566099	up	2.185673	0.06575137	up	1.3556119		putative protein
AT4G01650	0.001952861	up	2.1856313	0.94479823	down	0.990515124		hypothetical protein
AT5G49910	0.004246674	up	2.1653614	0.017168464	up	1.3723214	cpHSC70-2	heat shock protein 70 (gb|AAF27639.1) ; supported by cDNA: gi_6746591_gb_AF217459.1_AF217459
AT2G13650	0.004685331	up	2.1559172	0.07203564	down	0.803139891	GONST1	putative vanadate resistance protein
AT2G35510	0.005204357	up	2.1518579	0.22276577	up	1.2848186	SRO1	unknown protein ; supported by cDNA: gi_15810142_gb_AY056136.1_
AT1G25682 /// AT1G25988	0.00111891	up	2.144805	0.51422155	up	1.2289554		hypothetical protein contains similarity to virulence regulator GI:9106522 from [Xylella fastidiosa]
AT5G64650	0.001084539	up	2.1444304	0.22763218	down	0.881922174		50S ribosomal protein L17 ;supported by full-length cDNA: Ceres:2806.
AT3G54380	0.002141383	up	2.1443684	0.11920269	up	1.3924308		putative protein
AT3G54380	0.002141383	up	2.1443684	0.16595276	up	1.1197824		putative protein
AT1G28200	0.000979746	up	2.1415343	0.01212136	up	1.2015164	FIP1	FH protein interacting protein FIP1 identical to FH protein interacting protein FIP1 GI:6503012 from [Arabidopsis thaliana]; supported by full-length cDNA: Ceres: 30245.
AT3G02140	0.004447613	up	2.1382742	0.118513726	up	1.36244	TMAC2	hypothetical protein predicted by genscan; supported by cDNA: gi_14334515_gb_AY034949.1_
AT3G57450	0.002100152	up	2.1353223	0.110238194	up	1.2108641		putative protein ;supported by full-length cDNA: Ceres:12522.
AT1G77500	0.009341002	up	2.1269598	0.16244501	up	1.3221505		unknown protein similar to GB:AAD41429
AT5G49570	0.002299141	up	2.1187224	0.034977432	up	1.2093819	ATPNG1	putative protein contains similarity to peptide:N-glycanase
AT5G15870	0.013315696	up	2.1173239	0.03836413	up	1.249118		putative protein beta-glucan-elicitor receptor - Glycine max, EMBL:D78510
AT5G04540	0.008006978	up	2.113417	0.040743794	up	1.2573425		myotubularin - like protein myotubularin, Homo sapiens, EMBL:U46024
AT5G43430	0.000881454	up	2.1098142	0.24928382	up	1.2396808	ETFBETA	electron transfer flavoprotein beta-subunit-like ;supported by full-length cDNA: Ceres:120707.
AT3G16310	0.001016433	up	2.1046922	0.041375306	up	1.278933		unknown protein supported by full-length cDNA: Ceres:29750.
AT1G65220	0.001618492	up	2.1043286	0.22844465	up	1.1231172		unknown protein ;supported by full-length cDNA: Ceres:21223.
AT3G08740	0.020087924	up	2.1038358	0.26502615	up	1.1491836		putative elongation factor P (EF-P) similar to elongation factor P (EF-P) GB:Q54760 [Synechococcus PCC7942];supported by full-length cDNA: Ceres:2242.
AT1G29820	0.001912657	up	2.103586	0.8555225	down	0.989496397		unknown protein
AT3G54620	0.004462041	up	2.1016684	0.11309255	up	1.3783766	BZO2H4	Expressed protein ; supported by cDNA: gi_15451129_gb_AY054645.1_
AT3G23325	0.003810338	up	2.0999012	0.19235177	up	1.165795		Expressed protein ; supported by full-length cDNA: Ceres: 29124.
AT2G01600	0.007331064	up	2.0974498	0.034500066	up	1.2949481		unknown protein ; supported by full-length cDNA: Ceres: 32861.
AT5G55400	0.004378399	up	2.0876515	0.23119017	up	1.287917		fimbrin
AT1G71100	0.004333457	up	2.0821548	0.8900884	up	1.0319251	RSW10	putative ribose 5-phosphate isomerase similar to ribose 5-phosphate isomerase GB:6677767 from [Mus musculus];supported by full-length cDNA: Ceres:3116.

AT3G23050	0.003707468	up	2.081999	0.07224426	up	1.250493	IAA7	indoleacetic acid (IAA)-inducible gene (IAA7) identical to (IAA7) gene GB:U18409 [Arabidopsis thaliana] (J. Mol. Biol. 251 (4), 533-549 (1995));supported by full-length cDNA: Ceres:38286.
AT5G02800	0.004562977	up	2.081946	0.27342653	up	1.2264272		protein kinase - like protein kinase APK2a, Arabidopsis thaliana, EMBL:D88206
AT5G18420	0.002336344	up	2.0783234	0.1679313	up	1.171316		putative protein non-consensus GC donor splice site at exon 1, unknown (C40) protein, Homo sapiens, EMBL:AF103798
AT5G14530	0.002217916	up	2.0758085	0.41501245	up	1.0515006		putative protein various predicted proteins, Drosophila melanogaster, Caenorhabditis elegans, Schizosaccharomyces pombe, Saccharomyces cerevisiae, Oryza sativa;supported by full-length cDNA: Ceres:232187.
AT1G16670	0.000858013	up	2.073971	0.03484242	up	1.2462773		receptor-like serine/threonine kinase, putative similar to receptor-like serine/threonine kinase GI:2465923 from [Arabidopsis thaliana]; supported by cDNA: gi_16649102_gb_AY059921.1_
AT4G34180	0.003646828	up	2.0727293	0.037883233	up	1.372471		putative protein hypothetical protein slr2121, Synechocystis sp., PIR2:S75497;supported by full-length cDNA: Ceres:8686.
AT1G08450	0.00272414	up	2.072429	0.5434295	up	1.0474231	CRT3	calreticulin, putative similar to calreticulin GB:AAC49697 GI:2052383 from [Arabidopsis thaliana]; supported by cDNA: gi_2052382_gb_U66345.1_ATU66345
AT3G26000	0.003204085	up	2.0709164	0.06747672	up	1.3004735		unknown protein ; supported by cDNA: gi_16604357_gb_AY058077.1_
AT5G59490	0.011405552	up	2.0698593	0.2243016	up	1.1527405		putative ripening-related protein - like putative ripening-related protein Vitis vinifera EMBL:VVI237988
AT5G06560	0.000897589	up	2.0686724	0.08652659	up	1.262944		putative protein strong similarity to unknown protein (gb|AAF23201.1); supported by cDNA: gi_15293220_gb_AY051044.1_
AT1G01350 /// AT1G65840	0.000862464	up	2.067241	0.019147428	up	1.1859338	ATPAO4	putative protein contains similarity to zinc finger protein;supported by full-length cDNA: Ceres:3542.
AT1G67800	0.020795824	up	2.0654383	0.20064454	up	1.2100936		F12A21.7 hypothetical protein;supported by full-length cDNA: Ceres:34552.
AT1G77250	0.004317884	up	2.064737	0.09890249	up	1.2332743		hypothetical protein predicted by genemark.hmm
AT3G03950	0.002501207	up	2.0622115	0.8393618	up	1.0202847	ECT1	unknown protein similar to GB:AAD10646 [Arabidopsis thaliana];supported by full-length cDNA: Ceres:117342.
AT3G23990	0.001084539	up	2.0583274	0.12079283	up	1.185977	HSP60	mitochondrial chaperonin hsp60 identical to mitochondrial chaperonin hsp60 GB:Z11547 [Arabidopsis thaliana] (Plant Mol. Biol. 18 (5), 873-885 (1992))
AT5G53940	0.002772231	up	2.0535479	0.4694278	down	0.938974013		zinc-binding protein-like ; supported by full-length cDNA: Ceres: 21629.
AT1G32190	0.004752826	up	2.0527947	0.3179648	up	1.1398727		hypothetical protein similar to hypothetical protein GB:AAD18105 GI:4337191 from [Arabidopsis thaliana]
AT2G35710	0.014181308	up	2.0508091	0.26342568	up	1.2940189		putative glycogenin
AT3G15351	0.009812536	up	2.048326	0.19235177	up	1.1667383		Expressed protein ; supported by full-length cDNA: Ceres: 35376.
AT5G05730	0.006598756	up	2.0482244	0.46554396	down	0.893655209	ASA1	anthranilate synthase component I-1 precursor (sp|P32068)
AT1G71790	0.007658594	up	2.0400007	0.09399132	up	1.1865658		actin capping protein beta-2, putative similar to actin capping protein beta-2 GB:U07826 GI:565063 from [Gallus gallus]
AT1G14060	0.008768661	up	2.0377796	0.5912191	up	1.101109		Expressed protein ; supported by full-length cDNA: Ceres: 29867.
AT5G63130	0.043109983	up	2.0364425	0.31207505	up	1.2178016		unknown protein
AT1G19380	0.010866781	up	2.0342255	0.7501045	up	1.1018722		hypothetical protein predicted by genemark.hmm
AT5G48150	0.005168512	up	2.033931	0.07433224	up	1.3528765	PAT1	SCARECROW gene regulator-like ; supported by cDNA: gi_8132288_gb_AF153443.1_AF153443
AT4G30910 /// AT4G30920	0.002515898	up	2.0315623	0.8263813	down	0.979482199		leucyl aminopeptidase - like protein leucyl aminopeptidase, A. thaliana, PIR2:S22399
AT5G64050	0.015513823	up	2.0297656	0.9064886	up	1.0283369	ATERS	glutamate-tRNA ligase ; supported by cDNA: gi_11078545_gb_AF241841.1_AF241841
AT3G24450	0.005111816	up	2.0285604	0.22043444	up	1.3774072		unknown protein ; supported by full-length cDNA: Ceres: 20836.
AT4G35600	0.004423013	up	2.0248365	0.026149169	up	1.3716931	CONNEXIN 32	protein kinase - like protein serine/threonine-specific protein kinase APK1, Arabidopsis thaliana, PIR2:S28615
AT4G39955	0.003994003	up	2.0232425	0.3710152	down	0.94160274		Expressed protein ; supported by full-length cDNA: Ceres: 114014.
AT5G47430	0.001353216	up	2.0228746	0.2602267	up	1.2073501		DNA-binding protein-like
AT5G03905	0.008256256	up	2.0213985	0.04913365	up	1.3562913		Expressed protein ; supported by full-length cDNA: Ceres: 24058.
AT1G80480	0.002882038	up	2.0184042	0.8530558	down	0.982712611	PTAC17	hypothetical protein predicted by genscan+, contains Viral RNA helicase domain
AT1G55310	0.007003678	up	2.0170321	0.20230114	up	1.1813983	SR33	unknown protein ; supported by cDNA: gi_15293080_gb_AY050974.1_
AT3G48070	0.002318453	up	2.0155122	0.16120678	up	1.1313281		putative protein finger protein SIG1, Saccharomyces cerevisiae, PIR2:A56015;supported by full-length cDNA: Ceres:122162.
AT1G08210	0.021503331	up	2.0154507	0.98627895	down	0.996011968		hypothetical protein similar to putative protease GI:4415912 from [Arabidopsis thaliana]; supported by cDNA: gi_11993876_gb_AF329505.1_AF329505
AT5G17440	0.000760238	up	2.0147767	0.6819741	up	1.040162		putative protein cisplatin resistance-associated overexpressed protein - Homo sapiens, EMBL:AB034205; supported by cDNA: gi_15450598_gb_AY052667.1_
AT1G50300	0.004120271	up	2.0119116	0.31944028	up	1.106762	TAF15	hypothetical protein contains similarity to RNA-binding glycine-rich protein GB:D16205 GI:436790 from [Nicotiana sylvestris]; supported by cDNA: gi_13605792_gb_AF367295.1_AF367295
AT5G03440	0.001993154	up	2.0109797	0.2159259	up	1.3730907		putative protein ;supported by full-length cDNA: Ceres:3248.
AT5G66180	0.010410735	up	2.010607	0.21596955	up	1.1985954		putative protein contains similarity to nucleolar protein
AT2G47130	0.004203639	up	2.0103517	0.80999976	down	0.946462049		putative alcohol dehydrogenase
AT4G24730	0.012686064	up	2.009632	0.13892467	up	1.399219		hypothetical protein
AT2G18700	0.034525573	up	2.0045571	0.7272718	up	1.157241	ATTPS11	putative trehalose-6-phosphate synthase
AT5G67600	0.001562901	up	2.0031734	0.018036846	up	1.3410131		unknown protein ;supported by full-length cDNA: Ceres:9946.
AT1G77440	0.00346499	up	2.0026429	0.037010748	up	1.2104615	PBC2	putative 20S proteasome beta subunit PBC2 identical to residues 14-204 of 20S proteasome beta subunit PBC2 GB:AAC32069 [Arabidopsis thaliana]; supported by cDNA: gi_14326551_gb_AF385730.1_AF385730
AT5G65940	0.00597662	up	2.0023768	0.117536284	down	0.924636486	CHY1	3-hydroxyisobutyryl-coenzyme A hydrolase ; supported by cDNA: gi_8572759_gb_AF276301.1_AF276301
AT1G69920	0.000182259	up	745.64				ANAC003	NAM (no apical meristem)-like protein, putative similar to NAM (no apical meristem)-like protein GI:9759589 from (Arabidopsis thaliana)
AT2G28210	0.000245147	up	620.09326					hypothetical protein contains Zinc finger,C3HC4 type (RING finger), motif
AT4G27670	0.000150128	up	503.34335				IAA10	putative IAA1 protein Match to IAA10 protein gb|U18412 from A. thaliana; supported by cDNA: gi_12083201_gb_AF332396.1_AF332396
AT5G53680	2.13343E-05	up	226.3581				ERS2	putative ethylene receptor (ERS2) EST gb|W43451 comes from this gene
AT2G02000 /// AT2G02010	0.000470407	up	184.344				EMB2394	putative chloroplast 50S ribosomal protein, L6 Similar to Mycobacterium RlpF (gb|Z84395). ESTs gb|T75785,gb|R30580,gb|T04698 come from this gene;supported by full-length cDNA: Ceres:1730.
AT3G60120	0.00050108	up	119.7199					hypothetical protein predicted by genemark.hmm
AT3G14620	0.00026188	up	113.80484					lipase, putative similar to lipase GB:AAD01804 GI:4103627 from [Dianthus caryophyllus]; supported by cDNA: gi_15529150_gb_AY052199.1_
AT1G17870	0.000182259	up	77.54971				ATBZIP52	b-Zip DNA binding protein, putative similar to b-Zip DNA binding protein GB:CAB06697 GI:2246376 from [Arabidopsis thaliana]
AT1G71000	0.000307524	up	72.144104					protein phosphatase 2C, putative similar to protein phosphatase 2C GI:2582800 from [Medicago sativa]
AT2G29720 /// AT2G35660	0.000609045	up	66.02526					hypothetical protein predicted by genemark.hmm
AT4G17250	0.000470407	up	65.28991					gunknown protein contains non-consensus TG donor splice site at exon 1
AT1G72060	0.000628484	up	63.924526				ATCPNIFS	nitrogen fixation protein (nifS), putative similar to nitrogen fixation protein (nifS) GB:D64004 GI:1001701 from [Synechocystis sp]
AT4G04540 /// AT4G04570	0.001435934	up	52.154644					putative cinnamyl alcohol dehydrogenase similar to cinnamyl alcohol dehydrogenase, gi|1143445
AT1G44414	2.24457E-05	up	46.268063					unknown protein similar to hypothetical protein GB:AAD39330
AT4G19720	0.005558234	up	43.624054					transcription factor RUSH-1alpha isolog
AT3G55840	0.002549788	up	39.772995					anionic peroxidase, putative similar to anionic peroxidase GI:170202 from [Nicotiana sylvestris]
AT1G80820	0.001003029	up	39.209927					hypothetical protein contains Pfam profile: PF00117 Glutamine amidotransferase class-I
AT3G07150	1.9774E-07	up	37.198574					unknown protein
AT1G22810	0.002179487	up	37.17402					myb-related DNA-binding protein, putative similar to GB:U67132 from [Petroselinum crispum] (Plant J. 11 (5), 1079-1093 (1997))
AT4G16820	0.000471695	up	35.812614				ATEGY3	hypothetical protein predicted by genscan+
AT3G18250	0.012402414	up	35.609566					unknown protein contains similarity to DNA dependent reverse transcriptase GI:2920563 from [Spraguea lophii]
AT4G02550	0.000608712	up	35.132496					putative ATPase similar to GB:AAF28353 from [Fragaria x ananassa]
AT1G07160	0.00026188	up	34.5471					O-methyltransferase, putative similar to GB:AAF28353 from [Fragaria x ananassa];supported by full-length cDNA: Ceres:101583.
AT1G67980	0.000938459	up	34.43793					transcription factor, putative similar to GB:AAF36532 from [Drosophila melanogaster] (Mol. Cell 5 (1), 181-187 (2000))
AT5G52750	0.001386636	up	32.63614					hypothetical protein predicted by genemark.hmm
AT5G65600	0.000556665	up	32.393467					TINY-like transcription factor Contains similarity to transcription factor (TINY) isolog T02O04.22 gb|2062174 from A. thaliana BAC gb|AC001645;supported by full-length cDNA: Ceres:26867.
AT4G39235	0.00042212	up	31.751406					unknown protein
AT3G02840	0.000975366	up	31.58588					unknown protein ; supported by cDNA: gi_16604403_gb_AY058100.1_
AT4G28350	0.000817772	up	28.104809					hypothetical protein predicted by genemark.hmm
AT5G16970	0.001016433	up	27.451977					hypothetical protein predicted by genscan
AT5G42380	0.001191083	up	26.314348					lipase, putative contains Pfam profile: PF00657 Lipase/Acylhydrolase with GDSL-like motif;supported by full-length cDNA: Ceres:37307.
AT3G54150	0.000796205	up	24.566938					lipase, putative contains Pfam profile: PF01764: Lipase
AT4G35180	0.000242192	up	23.988483				AGO2	unknown protein contains similarity to argonaute protein GB:AAC18440 GI:2149640 from [Arabidopsis thaliana]
AT1G21130	0.002892027	up	23.947771					hypothetical protein similar to putative disease resistance protein GB:AAC14512 GI:2739389 from [Arabidopsis thaliana]
AT1G42970 /// AT5G22300	0.000644954	up	23.81227					hypothetical protein predicted by genemark.hmm;supported by full-length cDNA: Ceres:42217.
AT2G46830	0.000826175	up	22.799437				GAPB	Nitrilase 4 (sp|P46011) ; supported by full-length cDNA: Ceres: 6220.
AT5G05220	0.000242192	up	22.379799				ILL6	IAA-amino acid hydrolase, putative similar to IAA-amino acid hydrolase GI:3421384 from [Arabidopsis thaliana]
AT1G73740	0.00026188	up	22.219624					hypothetical protein predicted by genemark.hmm
AT5G22530	0.000190587	up	21.726728				AtTLP6	putative Tub family protein contains Pfam profiles: PF00646 F-box domain, PF01167 Tub family
AT4G37710	0.001713485	up	21.510754					hypothetical protein similar to hypothetical protein GB:AAD50049 GI:5734784 from [Arabidopsis thaliana]
AT2G43660	4.87195E-05	up	21.446054					hypothetical protein predicted by genemark.hmm
AT2G30750	0.006111431	up	21.41539				ATERF-8	AP2 domain containing protein, putative similar to AP2 domain containing protein RAP2.5 GI:2281635 from (Arabidopsis thaliana); supported by cDNA: gi_10567107_dbj_AB036884.1_AB036884
AT1G22110	0.000885609	up	20.148888					receptor-like serine/threonine kinase, putative similar to receptor-like serine/threonine kinase GB:AAC50043 GI:2465923 from [Arabidopsis thaliana]
AT5G24110	0.001446053	up	20.04331					hypothetical protein contains similarity to DNA-binding protein (PcMYB1) GI:2224896 from [Petroselinum crispum]
AT4G12120	0.000897589	up	19.712877				TAF7	Serine/arginine-rich protein, putative similar to GI:6572475 from [Arabidopsis thaliana]
AT5G18290	0.001084539	up	19.224548					hypothetical protein contains similarity to 2-ketocyclohexanecarboxyl-CoA hydrolase GI:3243084 from [Rhodopseudomonas palustris]
AT2G18670 /// AT2G18680	0.002359667	up	19.093266					hypothetical protein predicted by genemark.hmm
AT2G03020 /// AT4G16540	0.001440628	up	18.630407					hypothetical protein contains similarity to ABC transporter protein GI:6626257 from [Methanobacterium thermoautotrophicum]
AT2G31230	0.000889151	up	17.97217				BGLU45	beta-glucosidase, putative similar to beta-glucosidase GI:804655 from (Hordeum vulgare)
AT2G41810	0.00362326	up	16.917448					hypothetical protein similar to putative protein GB:CAA18164 [Arabidopsis thaliana]; supported by cDNA: gi_13878144_gb_AF370335.1_AF370335
AT1G21120	0.004661717	up	16.770685					dirigent protein, putative similar to dirigent protein GB:AAF25365 GI:6694709 from [Thuja plicata]
AT1G69890	0.000910835	up	16.280914				ATMGL	methionine/cystathionine gamma lyase, putative similar to methionine gamma-lyase GB:CAA04124.1 GI:2330885 from [Trichomonas vaginalis]; supported by cDNA: gi_15450931_gb_AY054546.1_
AT5G58770	0.000398326	up	16.170107				PMI2	hypothetical protein
AT1G14550	0.012523931	up	16.054934				DIN9	phosphomannose isomerase, putative similar to phosphomannose isomerase GI:10834550 from [Arabidopsis thaliana]
AT5G47070	0.000783487	up	15.69646					putative protein hypothetical proteins - Arabidopsis thaliana
AT3G53600	0.00240443	up	15.66346				CCoAMT	putative S-adenosyl-L-methionine:trans-caffeoyl-Coenzyme A 3-O-methyltransferase similar to S-adenosyl-L-methionine:trans-caffeoyl-Coenzyme A 3-O-methyltransferase GB:AAA62426 [Arabidopsis thaliana] (function=disease resistance); supported by cDNA: gi_682
AT2G31945	0.001105544	up	15.010266					hypothetical protein similar to GB:AAB61488 [Arabidopsis thaliana];supported by full-length cDNA: Ceres:34864.
AT5G20230	0.001408073	up	14.819119				ATGSTU12	putative glutathione transferase similar to glutathione transferase GB:CAA09188 [Alopecurus myosuroides]
AT5G57050	0.000975366	up	14.75806					putative polygalacturonase similar to polygalacturonase precursor (pectinase) GB:P35336 [Actinidia chinensis]
AT1G33600	0.001960174	up	14.609875					heat shock protein DnaJ, putative similar to heat shock protein DnaJ GI:5020005 from [Rhodothermus marinus]
AT2G39400	0.03163219	up	14.59029				AJH2	c-Jun coactivator-like protein (AJH2) identical to c-Jun coactivator-like protein (AJH2) GB:AF087412 [Arabidopsis thaliana]; supported by cDNA: gi_15724151_gb_AF411778.1_AF411778
AT5G10690	0.000624251	up	14.358138					unknown protein
AT5G64230	0.000470407	up	14.128161					hypothetical protein predicted by genemark.hmm;supported by full-length cDNA: Ceres:1321.
AT5G24660	0.008070807	up	13.942926					putative UDP-N-acetylglucosamine--N-acetylmuramyl-(pentapeptide)-pyrophosphoryl-undecaprenol N-acetylglucosamine transferase similar to putative UDP-N-acetylglucosamine--N-acetylmuramyl-(pentapeptide)-pyrophosphoryl-undecaprenol N-acetylglucosamine transf
AT2G26560	0.005313282	up	13.930524					putative glucosyltransferase similar to glucosyltransferase GB:AAB36653 from [Nicotiana tabacum]
AT1G61810	0.001541871	up	13.695766					putative choline kinase similar to GB:AAC49376 from [Glycine max]
AT1G67400 /// AT3G43400	0.000783487	up	13.664049					putative DnaJ protein similar to dnaJ-like protein GB:CAA72705 [Arabidopsis thaliana]; Pfam HMM hit:DnaJ, prokaryotic heat shock protein
AT2G44580	0.011745628	up	13.299507				ASHH1	unknown protein C-terminal portion similar to KIAA1090 protein GB:BAA83042 [Homo sapiens]
AT1G06800	0.000909821	up	13.266749					unknown protein contains similarity to seed protein B32E GI:19046 from [Hordeum vulgare]
AT1G64160	0.004423013	up	13.206986					putative DNA-binding protein similarity to short region of heat shock transcription factors: GB:CAB10177, GB:AAC31792, GB:CAA16745, GB:CAA16744 [Arabidopsis thaliana]; Pfam HMM hit: HSF-type DNA-binding domain;supported by full-length cDNA: Ceres:9552.
AT1G07500	0.000424695	up	12.859392					hypothetical protein predicted by genemark.hmm
AT5G46080	0.000976583	up	12.389764				CCR2	cinnamoyl CoA reductase, putative similar to cinnamoyl CoA reductase GB:AAF43141 GI:7239228 from [Populus tremuloides]; supported by full-length cDNA: Ceres: 32255.
AT5G52670	0.002882038	up	12.337866				GAD4	putative glutamate decarboxylase ; supported by cDNA: gi_13605709_gb_AF361836.1_AF361836
AT4G13550	0.002554126	up	12.229065				ISPD	putative sugar nucleotide phosphorylase ; supported by cDNA: gi_12697582_dbj_AB037876.1_AB037876
AT2G34650	0.000471695	up	11.988659					predicted by genefinder
AT5G37490	0.001790739	up	11.507387					hypothetical protein predicted by genscan
AT3G05030	0.000822181	up	11.376742					unknown protein ; supported by full-length cDNA: Ceres: 41621.
AT2G41230	0.003126527	up	10.790828					hypothetical protein contains NLS (PS50079) and proline-rich domain (PS50099).; supported by cDNA: gi_15724285_gb_AF412083.1_AF412083
AT4G28460	0.000694415	up	10.49137					RUB1-conjugating enzyme, putative similar to gi:6635457
AT5G67350	0.004249801	up	10.427293					unknown protein
AT5G23380	0.001507366	up	10.41747					unknown protein
AT4G11660	0.000909444	up	10.262974					putative DnaJ protein ;supported by full-length cDNA: Ceres:10312.
AT3G12510	0.001562901	up	9.992443					hypothetical protein predicted by genscan; supported by cDNA: gi_17063175_gb_AY062110.1_
AT4G00940	0.00082477	up	9.901258					Expressed protein ; supported by full-length cDNA: Ceres: 7152.
AT5G64890	0.006697245	up	9.857258					hypothetical protein predicted by genscan; supported by cDNA: gi_15294291_gb_AF410337.1_AF410337
AT1G31280	0.003190879	up	9.170031					hypothetical protein predicted by grail
AT3G03440	0.007097013	up	9.161164				PLP2	similar to latex allergen from Hevea brasiliensis ;supported by full-length cDNA: Ceres:1999.
AT5G50780	0.004508262	up	8.896694					putative carbonic anhydrase
AT5G03370	0.000470407	up	8.2023735				CTF2A	putative monooxygenase ;supported by full-length cDNA: Ceres:34214.
AT3G53540	0.002910527	up	8.180874				NIP2;1	putative aquaporin (plasma membrane intrinsic protein)
AT1G11100	0.005168512	up	7.753471				ATKT1	high affinity K+ transporter (AtKUP1/AtKT1p) identical to GB:AF029876; supported by cDNA: gi_2654087_gb_AF033118.1_AF033118
AT1G35210	0.002101625	up	7.7423534					putative pseudouridine synthase
AT2G26000	0.000739338	up	7.6571493				HSI2	putative VP1/ABI3 family regulatory protein
AT4G23180	0.002060344	up	7.636688					putative C3HC4 zinc finger protein ; supported by cDNA: gi_15010633_gb_AY045618.1_
AT1G44350	0.002973031	up	7.3187284				CYP71A12	putative cytochrome P450
AT1G51920	0.017313924	up	7.148298				ATERF15	ethylene reponse factor-like AP2 domain transcription factor
AT1G70500	0.003828674	up	7.125528					Expressed protein ; supported by full-length cDNA: Ceres: 258917.
AT1G55300	0.002309162	up	7.094453					hypothetical protein predicted by genscan
AT3G03310	0.000424419	up	7.0165286					putative alanine acetyl transferase
AT1G76700	0.003499105	up	6.9893537					Expressed protein ; supported by full-length cDNA: Ceres: 269036.
AT5G17780	0.003967331	up	6.892463				PID	putative protein kinase contains a protein kinase domain profile (PDOC00100); supported by cDNA: gi_7208441_gb_AF232236.1_AF232236
AT1G53440	0.001779904	up	6.8313956					putative protein with C-terminal RING finger
AT5G22690	0.000697835	up	6.802188					putative phospholipase ; supported by cDNA: gi_14532651_gb_AY039950.1_
AT4G09100	0.001486773	up	6.764435				EEL	putative bZIP transcription factor contains a bZIP transcription factor basic domain signature (PDOC00036);supported by full-length cDNA: Ceres:114632.
AT5G24570	0.004124512	up	6.6951885					unknown protein
AT5G48175	0.000878635	up	6.5405593					unknown protein similar to T11A07.10
AT2G25460	0.002805524	up	6.4940944					putative beta-1,3-glucanase, C terminal fragment C terminal homology only
AT1G02220	0.021944106	up	6.4810905					putative RING zinc finger protein
AT3G06660	0.001083083	up	6.4441056					unknown protein ; supported by cDNA: gi_15450510_gb_AY052357.1_
AT5G67340	0.008943931	up	6.2249756				APRR9	hypothetical protein predicted by genefinder; supported by cDNA: gi_10280999_dbj_AB046953.1_AB046953
AT2G29870 /// AT2G34390	0.033002205	up	6.1255407				CCA1	MYB-related transcription factor (CCA1)
AT2G32030	0.000881454	up	6.083295					putative lamin similar to blue copper-binding lamin GB:S71273 from [Arabidopsis thaliana];supported by full-length cDNA: Ceres:6695.
AT5G39580	0.021177657	up	5.969698					unknown protein
AT3G11210	0.001334746	up	5.847546					unknown protein ;supported by full-length cDNA: Ceres:38495.
AT5G14230	0.000638304	up	5.788244					unknown protein ;supported by full-length cDNA: Ceres:2355.
AT5G03560	0.002114421	up	5.7730703					unknown protein ; supported by cDNA: gi_15809941_gb_AY054239.1_
AT3G03900	0.002060344	up	5.5962167					unknown protein similar to putative protein GB:CAB40988 [Arabidopsis thaliana]
AT1G03770	0.00180385	up	5.5818567					putative adenylylsulfate kinase similar to GB:S47640 [Arabidopsis thaliana]
AT4G39890	0.001657291	up	5.543165				NHX2	putative sodium proton exchanger similar to sodium proton exchanger Nhx1 GB:AAD16946 [Arabidopsis thaliana]
AT1G15040	0.001178052	up	5.4950895				PDK	putative pyruvate dehydrogenase kinase similar to pyruvate dehydrogenase kinase GB:AAC97601. [Arabidopsis thaliana]
AT1G78460	0.0029923	up	5.3554363					hypothetical protein predicted by genemark.hmm; supported by cDNA: gi_14334461_gb_AY034922.1_
AT1G30370	0.012271293	up	5.289045					hypothetical protein predicted by genefinder
AT1G28600	0.014422428	up	5.239096					unknown protein identical to GB:AAD56318 (Arabidopsis thaliana)
AT5G41620	0.000722367	up	5.218584					hypothetical protein similar to putative protein GB:CAA18199 [Arabidopsis thaliana]
AT4G26880	0.007163932	up	5.213519					putative protein kinase similar to protein kinase (APK1A) GB:Q06548 [Arabidopsis thaliana]; contains Pfam profile: PF00069 Eukaryotic protein kinase domain
AT4G39230	0.002797608	up	5.170057					unknown protein ; supported by full-length cDNA: Ceres: 21357.
AT5G09570	0.005095818	up	5.06938				ATMYB65	putative transcription factor similar to GAMyb protein GB:CAA61021 [Hordeum vulgare]
AT4G26200	0.005809662	up	4.9973574					hypothetical protein predicted by genscan;supported by full-length cDNA: Ceres:100676.
AT4G26500	0.001414006	up	4.9214706					unknown protein ; supported by cDNA: gi_15982827_gb_AY057521.1_
AT5G41610	0.000560826	up	4.899086					hypothetical protein predicted by genscan+
AT5G61190	0.003842516	up	4.873436				ATMRP7	ABC transporter, putative similar to AtMRP4 (transport of glutathione-conjugates into the vacuole) GB:CAA05625 [Arabidopsis thaliana]; contains Pfam profile: PF00005 ABC transporter
AT5G25475	0.000886423	up	4.66144					hypothetical protein predicted by genemark.hmm; supported by cDNA: gi_15146180_gb_AY049231.1_
AT3G20660	0.001999927	up	4.6312847				CYP72A8	putative cytochrome P450 similar to GB:Q05047 from [Catharanthus roseus]; supported by cDNA: gi_15529168_gb_AY052208.1_
AT3G54510	0.00852875	up	4.6003423				AtRABA1g	ras-related GTP-binding protein contains Pfam profile: PF00071 ras family
AT4G32300	0.002411771	up	4.5619884					hypothetical protein predicted by genemark.hmm
AT4G29070	0.001030927	up	4.5214596					hypothetical protein predicted by genscan+
AT3G15060	0.001952256	up	4.4981146				ATSYTF	unknown protein contains Pfam profile: PF00168 C2 domain; supported by cDNA: gi_15983786_gb_AY056799.1_
AT5G57190	0.00111891	up	4.4704494					unknown protein ; supported by cDNA: gi_14532501_gb_AY039875.1_
AT1G04100	0.001629458	up	4.4054356				ATOCT4	hypothetical protein contains similarity to transporter proteins
AT2G46420	0.003088097	up	4.283445					unknown protein ; supported by cDNA: gi_16649058_gb_AY059899.1_
AT2G22360	0.006078816	up	4.2751894					hypothetical protein similar to unknown protein GB:AAF14829 from [Arabidopsis thaliana];supported by full-length cDNA: Ceres:124466.
AT1G17330	0.003591348	up	4.261802					unknown protein similar to putative protein kinase GB:CAB10257 from [Arabidopsis thaliana]; supported by cDNA: gi_14334661_gb_AY035004.1_
AT4G08230	0.004047718	up	4.224487				AtMYB15	myb-related transcription factor, putative similar to myb-related transcription factor GB:CAA66952 from [Lycopersicon esculentum]
AT1G66480	0.011708413	up	4.195859					AMP-binding protein, putative similar to AMP-binding protein GB:CAA96521 from [Brassica napus] (Plant Mol. Biol. (1997) 33 (5), 911-922)
AT3G11840	0.005741971	up	4.140571				RPL15	50S ribosomal protein L15, chloroplast precursor identical to GB:P25873 from [Arabidopsis thaliana];supported by full-length cDNA: Ceres:10926.
AT1G60550	0.007825783	up	4.0996494					unknown protein
AT3G11440	0.002668794	up	4.096467				GAPA	glyceraldehyde 3-phosphate dehydrogenase A subunit (GapA) identical to glyceraldehyde 3-phosphate dehydrogenase A subunit (GapA) GB:M64114 (Gene 104, 133-138 (1991)); supported by cDNA: gi_166701_gb_M64114.1_ATHGAPAA
AT1G50400	0.006068443	up	4.095157					unknown protein similar to unknown protein GB:AAD14522 from [Arabidopsis thaliana]
AT3G26070 /// AT3G26080	0.008311855	up	4.014878					unknown protein GC donor splice site at exon 16 ; supported by cDNA: gi_17063188_gb_AY062117.1_
AT3G13100	0.007063367	up	3.9649405				AIG2	AIG2-like protein similar to AIG2 protein GB:P54121 from [Arabidopsis thaliana];supported by full-length cDNA: Ceres:28511.
AT1G23180	0.002859793	up	3.953376				ADT4	putative chloroplast prephenate dehydratase similar to bacterial PheA gene products
AT4G26700	0.005200221	up	3.944				ATSEN1	putative protein tRNA intron endonuclease - Arabidopsis thaliana, EMBL:AB036339; supported by cDNA: gi_6635255_dbj_AB036339.1_AB036339
AT4G22640	0.002535919	up	3.9354615					hypothetical protein
AT2G30320	0.004378328	up	3.881355				CYP94B3	cytochrome P450-like protein cytochrome P450 CYP94A1 - Vicia sativa,PIR2:T08014
AT1G09480 /// AT1G09490	0.001353216	up	3.8018095					RNA polymerase III subunit - like protein RNA polymerase III subunit, Homo sapiens, gb:CAB41919;supported by full-length cDNA: Ceres:157800.
AT5G50570 /// AT5G50670	0.001105544	up	3.7737699					calcium dependent protein kinase - like strong similarity to calcium dependent protein kinase CP4, Arabidopsis thaliana, EMBL:AF130252
AT4G37295	0.006486604	up	3.7378557					putative protein predicted protein, Oryza sativa, EMBL:AP000367;supported by full-length cDNA: Ceres:1514.
AT1G04310	0.000932534	up	3.7329938					putative protein predicted proteins, Arabidopsis thaliana
AT4G19460	0.004206007	up	3.732621					zinc finger - like protein Zat11 zinc finger protein, Arabidopsis thaliana, EMBL:ATRZAT11
AT4G30340	0.001993154	up	3.7222428					embryonic abundant protein -like embryonic abundant protein EMB34, Picea glauca, PIR:T09281; supported by full-length cDNA: Ceres: 6529.
AT3G22750	0.009571609	up	3.716461					putative protein Various predicted proteins, Arabidopsis thaliana
AT4G14560	0.005753261	up	3.7128816					nematode resistance protein-like protein Hs1pro-1 nematode resistance gene, Beta procumbens, EMBL:BPU79733;supported by full-length cDNA: Ceres:149697.
AT3G09030	0.000743333	up	3.6943989					putative protein
AT5G54300	0.012075392	up	3.6178808				UVI4-LIKE	putative protein hypothetical protein At2g42260 - Arabidopsis thaliana, EMBL:AC002561
AT1G67070	0.00678207	up	3.590831					beta-glucosidase-like protein several beta-glucosidases - different species
AT4G32590	0.002968215	up	3.5526884					putative protein hypothetical proteins At2g44600 - Arabidopsis thaliana, EMBL:AAC27462
AT3G19970	0.003948349	up	3.5313914					hypothetical protein
AT1G76970	0.011434331	up	3.523525					putative protein
AT4G18280	0.002089313	up	3.5132759					contains similarity to a protein kinase domain (Pfam: pkinase.hmm, score: 166.20) and to legume lectins beta domain (Pfam: lectin_legB.hmm, score: 139.32)
AT1G73090	0.005702582	up	3.4991996					predicted protein of unknown function similar to A. thaliana protein T10M13.21, GenBank accession number AF001308
AT4G24110	0.013706835	up	3.4579499					hypothetical protein similar to A. thaliana hypothetical protein F15K9.11, GenBank accession number AC005278
AT1G23830	0.001632983	up	3.4395182					putative alcohol dehydrogenase similar to plant short chain alcohol dehydrogenase
AT3G49000	0.008181482	up	3.405253					putative receptor-like protein kinase
AT5G47220	0.031022115	up	3.3936985					hypothetical protein
AT2G18600	0.01477773	up	3.3898935					putative protein RING-H2 zinc finger protein ATL6 - Arabidopsis thaliana,PID:g4928403
AT5G24340	0.004454608	up	3.339096				ATMPK5	MAP kinase (ATMPK5) possible internal deletion at position 161, missing one A residue; reference GI:457401; supported by cDNA: gi_457401_dbj_D21841.1_ATHATMPK5
AT2G14520	0.03986957	up	3.288007				AT-HSFB2B	heat shock transcription factor - like protein heat shock transcription factor HSF29, Glycine max, PIR2:S59541
AT2G31990	0.006724387	up	3.2822258				SEC1B	putative protein acetylcholine regulator unc-18 - Caenorhabditis elegans, PIR2:S36747;supported by full-length cDNA: Ceres:94950.
AT3G26650	0.033983175	up	3.2603862					putative protein hypothetical protein, Synechocystis sp., PIR2:S76637
AT3G21810	0.039842643	up	3.2217777				IAA1	auxin-responsive protein IAA1 ;supported by full-length cDNA: Ceres:33860.
AT3G48520	0.011428962	up	3.2196076					triacylglycerol lipase like protein
AT3G09020	0.005132727	up	3.208698					hypothetical protein ; supported by cDNA: gi_16323154_gb_AY057681.1_
AT1G06770	0.006954939	up	3.2047276					unknown protein multidrug resistance protein, Homo sapiens, PIR2:S71841
AT5G47110	0.009209775	up	3.198157					glycine-rich cell wall protein-like glycine-rich protein 1.0 precursor, Phaseolus vulgaris, PIR1:S01821; supported by cDNA: gi_14030676_gb_AF375429.1_AF375429
AT5G58210	0.007685649	up	3.182116					putative protein predicted protein, Arabidopsis thaliana,PID:G3834322; supported by full-length cDNA: Ceres: 6321.
AT4G36550	0.003298556	up	3.173999					putative protein various predicted proteins, Arabidopsis thaliana;supported by full-length cDNA: Ceres:42503.
AT4G34240	0.002066824	up	3.1411664					putative protein chitinase / lysozyme PZ precursor, Nicotiana tabacum, PIR2:S51591
AT2G20950	0.002413488	up	3.1130922					putative protein various predicted proteins, Arabidopsis thaliana
AT5G52960	0.001960174	up	3.052222				CRK10	serine/threonine kinase -like protein serine/threonine kinase, Brassica oleracea; supported by cDNA: gi_13506744_gb_AF224705.1_AF224705
AT4G18890	0.0029923	up	2.988089					putative polygalacturonase polygalacturonase (EC 3.2.1.15) - avocado, EMBL:X66426
AT5G12930	0.00245443	up	2.9676137					putative protein gene T22C1.1, Caenorhabditis elegans, Z75550
AT1G63720	0.005773597	up	2.9522028					putative protein predicted proteins, Arabidopsis thaliana
AT3G23790	0.007854914	up	2.9513254				ACS7	1-aminocyclopropane-1-carboxylate synthase -like protein ACC synthase , Malus domestica, U73816
AT3G02860	0.007559991	up	2.9231858				ATSUFE	putative protein Uvi31, Schizosaccharomyces pombe, G1381578;supported by full-length cDNA: Ceres:112720.
AT4G34340	0.006689496	up	2.9174583				ATFIM1	fimbrin-like protein (ATFIM1) ; supported by cDNA: gi_2905892_gb_U66424.1_ATU66424
AT1G18680	0.004479121	up	2.9109547					STIG1 like protein STIG1 protein - common tobacco, EMBL:X77823
AT5G64900	0.00108055	up	2.9100628				HSP21	heat shock protein 21
AT5G06190	0.002368419	up	2.9056833					receptor protein kinase like protein lectin receptor-like serine/threonine kinase lecRK1, Arabidopsis thaliana, PIR2:S68589
AT5G16900	0.007734011	up	2.8862517					hypothetical protein
AT1G61280	0.013399097	up	2.8689408					putative protein
AT1G05190	0.008878504	up	2.8673995					putative protein conserved hypothetical protein AF0205, Archaeoglobus fulgidus, PIR2:E69275
AT2G37150	0.024855075	up	2.8660936					hypothetical protein ;supported by full-length cDNA: Ceres:249331.
AT5G08380	0.001838252	up	2.8597853					putative protein putative phosphatase, Gallus gallus, gb:AJ006529
AT3G51500	0.00397403	up	2.853111				ATDGK7	putative protein diacylglycerol kinase iota - Homo sapiens,PID:g3676530; supported by cDNA: gi_13430523_gb_AF360174.1_AF360174
AT1G06850	0.018775038	up	2.8526137					putative protein hypothetical protein - Arabidopsis thaliana,PID:g3738329;supported by full-length cDNA: Ceres:17578.
AT3G06483	0.007198097	up	2.8335721					putative protein BEM46 PROTEIN, Schizosaccharomyces pombe, PID:D1022245
AT5G23080	0.000847379	up	2.8016896					S-receptor kinase -like protein serine/threonine-specific protein kinase PK10 precursor, Oryza sativa, PIR2:S50767
AT3G22260	0.019554036	up	2.8014631					hypothetical protein ; supported by cDNA: gi_15028324_gb_AY045965.1_
AT1G74320	0.002790173	up	2.7633991				ALDH3I1	putative aldehyde dehydrogenase aldehyde dehydrogenase (NAD+), Rattus norvegicus, PIR2:A41028; supported by cDNA: gi_15451105_gb_AY054633.1_
AT4G29530	0.035157017	up	2.7544181				TAF8	hypothetical protein ;supported by full-length cDNA: Ceres:115167.
AT3G47490	0.001091397	up	2.7433364				LHT7	amino acid permease - like protein Lily mRNA, Lilium longiflorum, gb:D21814;supported by full-length cDNA: Ceres:36461.
AT2G41070	0.007950301	up	2.730358					putative protein
AT5G02430	0.009147886	up	2.7298582				LEJ2	putative protein ;supported by full-length cDNA: Ceres:13475.
AT4G28550	0.003297276	up	2.7081606					Expressed protein ; supported by full-length cDNA: Ceres: 263956.
AT1G47270	0.003277212	up	2.6891034					putative protein predicted protein, Arabidopsis thaliana;supported by full-length cDNA: Ceres:207350.
AT3G15150	0.009250204	up	2.6847954				SQE3	squalene epoxidase - like protein squalene epoxidase, Panax ginseng, AB003516;supported by full-length cDNA: Ceres:118648.
AT1G26620	0.025763141	up	2.662584					putative protein
AT3G44720	0.00741876	up	2.652891					NAD(P)H oxidoreductase, isoflavone reductase - like protein Pyrc2, Pyrus communis, AF071477
AT4G37760	0.006513093	up	2.633858					Expressed protein ; supported by full-length cDNA: Ceres: 147351.
AT3G23250	0.019863352	up	2.6284368				AtRABH1c	small GTP-binding protein - like SR1 Nt-rab6, Nicotiana tabacum, L29273; supported by cDNA: gi_14423429_gb_AF386952.1_AF386952
AT1G64660	0.004257335	up	2.623557					putative protein hypothetical protein T6H20.90 - Arabidopsis thaliana, EMBL:AL096859; supported by cDNA: gi_16648747_gb_AY058150.1_
AT2G30070	0.014215199	up	2.6218162					putative protein Hsp70 binding protein HspBP1 - Homo sapiens, EMBL:AF093420
AT3G18370	0.001446053	up	2.5947037					putative protein rab11 binding protein, Bos taurus, EMBL:AF117897
AT4G23820	0.006989571	up	2.5696435					putative protein ; supported by full-length cDNA: Ceres: 39035.
AT2G33735	0.007567472	up	2.5669665					putative protein ; supported by cDNA: gi_14326524_gb_AF385716.1_AF385716
AT3G28430	0.009526052	up	2.5349207				FFC	signal recognition particle 54CP protein precursor ; supported by cDNA: gi_15293130_gb_AY050999.1_
AT3G01210	0.001084539	up	2.525005					putative protein ;supported by full-length cDNA: Ceres:29990.
AT1G10250 /// AT1G10450	0.004360735	up	2.5177948					putative protein contains similarity to inositol polyphosphate 5'-phosphatase
AT3G12300	0.039705437	up	2.5131183					unknown protein ; supported by cDNA: gi_15450646_gb_AY052691.1_
AT5G08565	0.004623523	up	2.496227					unknown protein ;supported by full-length cDNA: Ceres:6487.
AT5G04250	0.012087433	up	2.4822495				ATAGAL1	alpha-galactosidase - like protein alpha-galactosidase, coffee, PIR:T50781
AT1G54260	0.013107718	up	2.4327657					Expressed protein ; supported by full-length cDNA: Ceres: 252311.
AT5G04980	0.002437145	up	2.3999877					putative protein
AT1G61640	0.004494373	up	2.3866603					putative protein predicted proteins, Arabidopsis thaliana and Oryza sativa;supported by full-length cDNA: Ceres:254744.
AT3G01070	0.010550801	up	2.3792853					putative protein predicted proteins
AT2G14880	0.004317884	up	2.376809					ankyrin - like protein 270 kDa ankyrin G isoform, Rattus norvegicus, EMBL:AF102552
AT2G30580	0.006617225	up	2.376152					putative protein ;supported by full-length cDNA: Ceres:21085.
AT4G03140	0.03748203	up	2.3744328					receptor protein kinase -like protein serine/threonine-specific receptor protein kinase (EC 2.7.1.-), Arabidopsis thaliana, PIR:S71277
AT3G28930	0.004378399	up	2.3666923				AT-AER	quinone oxidoreductase -like protein probable quinone oxidoreductase (EC 1.6.5.5) P1, Arabidopsis thaliana, PIR:S57611
AT5G39660	0.008370288	up	2.3646576					spindle pole body protein-like spindle pole body protein - Homo sapiens, EMBL:AF042379; supported by cDNA: gi_14532825_gb_AY040037.1_
AT3G45590	0.00658096	up	2.3514497					putative protein predicted proteins, Arabidopsis thaliana
AT1G76710	0.01764324	up	2.3479517				SIP1;2	putative protein similar to unknown protein (gb|AAF26804.1)

AT1G77570	0.039004486	up	2.3366601				ATBCB	blue copper binding protein ;supported by full-length cDNA: Ceres:7767.
AT5G14910	0.004223302	up	2.3273177				ANAC089	NAM (no apical meristem)-like protein ; supported by cDNA: gi_14326464_gb_AF385685.1_AF385685
AT4G02880	0.002896973	up	2.3183203					unknown protein
**AT5G03940**	**0.004428263**	**up**	**2.2901552**				**EMB1705**	**putative protein similar to unknown protein (emb|CAB92117.1)**
AT1G21160	0.011092954	up	2.278103					unknown protein ; supported by cDNA: gi_14532613_gb_AY039931.1_
AT1G08490	0.003190791	up	2.2676976					disease resistance protein-like
AT5G22290	0.004085849	up	2.2630596				TGH	unknown protein
AT2G25920	0.036399268	up	2.258056					putative protein similar to unknown protein (gb AAD11584.1)
AT1G71230	0.025855219	up	2.2478292					unknown protein
AT5G22340	0.025855219	up	2.2402391				WRKY30	unknown protein ;supported by full-length cDNA: Ceres:6469.
**AT1G73880 /// AT1G73885**	**0.011492198**	**up**	**2.237636**					**putative protein similar to unknown protein (pir |D72332)**
AT3G09830	0.006643208	up	2.234114					unknown protein ;supported by full-length cDNA: Ceres:141953.
AT2G17110	0.010379666	up	2.2319005					putative protein similar to unknown protein (emb CAB62461.1);supported by full-length cDNA: Ceres:268701.
AT1G53170	0.033266027	up	2.2301989					Expressed protein ; supported by full-length cDNA: Ceres: 32341.
AT4G28830	0.006631438	up	2.2248187					putative protein predicted proteins, Arabidopsis thalina
AT5G02020	0.003852752	up	2.2246642					peroxidase ATP24a
AT4G38980	0.001142363	up	2.2215548				CDF2	promoter-binding protein like ascorbate oxidase promoter-binding protein, winter squash, PIR:T09661; supported by cDNA: gi_15983796_gb_AY056804.1_
**AT5G02150**	**0.013067**	**up**	**2.216737**					**RING zinc finger protein-like ;supported by full-length cDNA: Ceres:207148.**
AT4G36910	0.002358117	up	2.2112536				ATCHX18	Na+/H+ antiporter-like protein
AT3G61800	0.001618492	up	2.2054513					putative protein strong similarity to unknown protein (gb|AAF24581.1)
AT2G46670 /// AT2G46790	0.04205231	up	2.1943243				CML37	putative protein contains similarity to calmodulin;supported by full-length cDNA: Ceres:99348.
AT2G02500	0.009948741	up	2.1872895					unknown protein ;supported by full-length cDNA: Ceres:21404.
AT3G60200	0.000945634	up	2.1801543					serine/threonine protein kinase-like protein
AT5G23520	0.005178333	up	2.1753192					protein serine threonine kinase-like
**AT1G08410**	**0.001097499**	**up**	**2.1747131**					**Lil3 protein**
AT3G49370	0.006678334	up	2.1600504				ATERF-2	ethylene responsive element binding factor 2 (ATERF2) (sp|O80338) ;supported by full-length cDNA: Ceres:3012.
AT5G62620	0.001594597	up	2.1573105					Expressed protein ; supported by full-length cDNA: Ceres: 7015.
AT4G11330	0.005888908	up	2.155881					putative protein contains similarity to squamosa promoter binding protein;supported by full-length cDNA: Ceres:113229.
AT2G25735	0.031028936	up	2.1447499					putative protein strong similarity to unknown protein (pir||T05518)
AT2G30470	0.032547507	up	2.1417863					putative protein similar to unknown protein (emb|CAA71173.1)
AT4G23860	0.011488632	up	2.103852					putative protein similar to unknown protein (emb|CAA71173.1);supported by full-length cDNA: Ceres:19542.
**AT4G31020**	**0.011858638**	**up**	**2.0878263**					**putative protein similar to unknown protein (pir |S77140); supported by full-length cDNA: Ceres: 14354.**
AT5G44650	0.00412115	up	2.0823836					putative protein contains similarity to RNA-binding protein
AT5G41400	0.003171321	up	2.0771468					cotton fiber expressed protein 1-like protein
AT3G56290	0.007833657	up	2.076949				ABI2	protein phosphatase 2C ABI2 (PP2C) (sp|O04719)
AT1G17520	0.011235356	up	2.0742455				PSD2	phosphatidylserine decarboxylase
AT3G25920	0.008670353	up	2.0711167					similar to unknown protein (sp|P23253)
AT5G65290	0.009341002	up	2.0693245					dehydrodolichyl diphosphate - like protein dehydrodolichyl diphosphate, Arabidopsis thaliana, EMBL:ATH277136
**AT3G14075**	**0.00250145**	**up**	**2.0657163**					**putative protein various predicted proteins, Arabidopsis thaliana**
AT5G17410	0.022468716	up	2.063168					putative protein strong similarity to unknown protein (gb AAD55296.1)
AT4G30620	0.011194455	up	2.0409334					unknown protein ; supported by cDNA: gi_15293016_gb_AY050942.1_
AT3G27610	0.03347033	up	2.038728				PROPEP2	unknown protein ;supported by full-length cDNA: Ceres:9242.
AT4G18260	0.013122132	up	2.0369008				ATPEP1	unknown protein ; supported by full-length cDNA: Ceres: 25655.
AT3G57860	0.017864032	up	2.0301824					unknown protein
AT4G02410	0.024171032	up	2.028109					receptor protein kinase-like protein
**AT5G22370**	**0.019079464**	**up**	**2.0138943**					**putative protein strong similarity to unknown protein (pir||T00518)**
AT1G26900	0.007249471	up	2.0022485					unknown protein

Supplementary Table S1 As-responsive genes in *Arabidopsis* Col-0 and Ws-2 ecotypes (Col200 -Ws100 Commonly Up)

AGI	Col-200 q-value	Col-200 FC	Ws-100 q-value	Ws-100 FC	Gene Symbol	Target Description
AT1G52560	1.24229E-05	1616.5184	0.002145344	91.674576		chloroplast-localized small heat shock protein, putative similar to chloroplast-localized small heat shock protein GI:6601536 from [Funaria hygrometrica]
AT1G53540	0.001838252	1482.3334	0.001442748	210.24763		17.6 kDa heat shock protein (AA 1-156) identical to GI:4376161 from (Arabidopsis thaliana) (Nucleic Acids Res. 17 (19), 7995 (1989))
AT4G10250	1.24229E-05	1371.7506	0.001442748	158.6542	ATHSP22.0	heat shock protein 22.0 ; supported by cDNA: gi_511795_gb_U11501.1_ATU11501
AT3G46230	0.007950301	1116.181	0.010616389	134.5163	ATHSP17.4	heat shock protein 17 ; supported by cDNA: gi_15294149_gb_AF410266.1_AF410266
AT5G12030	0.004873737	1084.2805	0.010031367	115.714584	AT-HSP17.6A	heat shock protein 17.6A
AT5G59720	0.000263285	1058.1091	0.0025088	37.05618	HSP18.2	heat shock protein 18 ;supported by full-length cDNA: Ceres:97197.
AT5G12020	0.003779694	738.46375	0.00909261	121.96679	HSP17.6II	heat shock protein 17.6-II ;supported by full-length cDNA: Ceres:2281.
AT4G25200	0.000938459	543.69604	0.002705098	38.29138	ATHSP23.6-MITO	Arabidopsis mitochondrion-localized small heat shock protein (AtHSP23.6-mito) ; supported by cDNA: gi_1669865_gb_U72958.1_ATU72958
AT1G26380	0.000586765	497.56696	0.004986228	64.8633		hypothetical protein similar to reticuline oxidase-like protein GB:CAB45850 GI:5262224 from [Arabidopsis thaliana]; supported by cDNA: gi_13430839_gb_AF360332.1_AF360332
AT2G47180	0.000403253	459.57462	0.005716885	55.173183	ATGOLS1	putative galactinol synthase ;supported by full-length cDNA: Ceres:124236.
AT5G37670	0.000302628	450.71524	0.003620768	20.38656		low-molecular-weight heat shock protein - like cytosolic class I small heat-shock protein HSP17.5, Castanea sativa, EMBL:CSA9880
AT4G37370	0.005097881	422.19272	0.000612937	82.682365	CYP81D8	cytochrome P450 - like protein cytochrome P450, Glycyrrhiza echinata, AB001379;supported by full-length cDNA: Ceres:253698.
AT1G16030	0.000541825	413.37848	0.0025088	21.185383	HSP70B	heat shock protein hsp70, putative similar to heat shock protein hsp70 GI:1771478 from [Pisum sativum]
AT1G07400 /// AT	0.011078638	389.3115	0.009533367	22.152338		heat shock protein, putative similar to heat shock protein GI:19617 from [Medicago sativa];supported by full-length cDNA: Ceres:32795.
AT1G72660	0.000147438	311.9792	0.001992044	21.743382		putative GTP-binding protein similar to GTP-binding protein GB:AAB53256 [Arabidopsis thaliana]
AT3G09640	0.000245147	299.91833	0.001810002	50.935005	APX2	putative ascorbate peroxidase strong similarity to ascorbate peroxidase GB:CAA56340
AT3G60140	1.24229E-05	298.64627	0.000612937	54.32797	DIN2	beta-glucosidase-like protein several beta-glucosidases - different species; supported by cDNA: gi_10834547_gb_AF159376.1_AF159376
AT2G23270	0.000245147	269.3431	0.004857321	20.694763		hypothetical protein predicted by genefinder; similar to GPl2464853lgnllPIDle353149lZ99707;supported by full-length cDNA: Ceres:40943.
AT1G54050	0.00050108	261.0225	0.008135078	39.312378		heat-shock protein, putative similar to heat-shock protein GI:472939 from [Helianthus annuus];supported by full-length cDNA: Ceres:97415.
AT2G44460	0.020400725	188.8112	0.001099773	426.6296		putative beta-glucosidase
AT4G37290	0.007003678	160.69086	0.001938161	34.698418		hypothetical protein ;supported by full-length cDNA: Ceres:41783.
AT4G22690 /// AT	0.000707875	148.998	0.012106664	16.88155	CYP706A1	cytochrome P450 - like protein flavonoid 3',5'-hydroxylase Hf1, Petunia x hybrida, PIR2:S38985
AT2G26150	0.001573711	128.34378	0.005567456	14.212848	ATHSFA2	putative heat shock transcription factor
AT2G46240	0.001498734	124.78607	0.005044704	16.97928	BAG6	hypothetical protein predicted by genscan and genefinder
AT1G74310	0.00365712	121.5919	0.006968671	13.853434	ATHSP101	heat shock protein 101 (HSP101) identical to heat shock protein 101 GI:6715468 GB:AAF26423 from [Arabidopsis thaliana]
AT4G21320	0.001964642	113.11928	0.010792479	13.722548	HSA32	hypothetical protein
AT2G29500	0.007524988	100.28438	0.005567558	33.662014		putative small heat shock protein ;supported by full-length cDNA: Ceres:25828.
AT1G03070	0.000385722	92.9563	0.011538637	7.3816705		putative glutamate/aspartate-binding peptide Belongs to the PFl01027 Uncharacterized protein family UPF0005 with 7 transmembrane domains
AT5G64510	0.007840016	84.797935	0.038061347	2.6037652		putative protein similar to unknown protein (pir lT03813)
AT2G32120	0.000269321	83.49448	0.004813203	17.157768	HSP70T-2	70kD heat shock protein ;supported by full-length cDNA: Ceres:98979.
AT4G23493	0.000470407	82.37466	0.002808379	12.517303		Expressed protein ; supported by full-length cDNA: Ceres:270232
AT2G19310	0.000826175	81.74177	0.011176319	9.024175		putative small heat shock protein ;supported by full-length cDNA: Ceres:99763.
AT2G04040	0.000826175	74.82664	0.003779925	17.528654	ATDTX1	hypothetical protein similar to hypothetical protein GB:AAC27412
AT5G26220	0.002862363	71.982544	0.001089257	220.93124		putative protein cation transport protein chaC, Escherichia coli, PIR:G64868
AT4G25380	0.00021261	62.388603	0.010465828	11.900874		putative protein zinc finger protein ZNF216 - Mus musculus,PID:g3643807
AT3G10020	0.001451737	56.08211	0.007912322	5.6126356		unknown protein predicted by genefinder, multiple est matches;supported by full-length cDNA: Ceres:7073.
AT1G60750	0.009092798	50.36276	0.003083379	7.310755		auxin-induced protein, putative similar to auxin-induced atb2 GI:6562980 from [Arabidopsis thaliana]
AT2G43000	0.006980976	45.7885	0.00935342	10.305442	ANAC042	NAM (no apical meristem)-like protein
AT3G09350	0.002643562	45.167686	0.004813203	7.009724		hypothetical protein predicted by genscan+;supported by full-length cDNA: Ceres:19154.
AT5G39050	0.037348744	44.933346	0.003857305	57.33315		acyltransferase - like protein Anthocyanin 5-aromatic acyltransferase, Gentiana triflora, EMBL:AB010708; supported by cDNA: gi_15450468_gb_AY052335.1_
AT1G14200	0.001446053	43.71331	0.024437424	2.4237578		unknown protein location of EST TAT5A03; 5' end, gblZ26679
AT5G57220	0.009198523	41.740616	0.006825261	11.1432295	CYP81F2	cytochrome P450
AT5G10695	0.000843909	41.057728	0.00579036	5.7971644		Expressed protein ; supported by full-length cDNA: Ceres: 103171.
AT5G12110	0.000975366	40.578598	0.006200098	13.276968		elongation factor 1B alpha-subunit (emblCAB64729.1)
AT4G11370	0.000945634	39.31794	0.007051962	8.036543	RHA1A	RING-H2 finger protein RHA1a -like protein ;supported by full-length cDNA: Ceres:21591.
AT5G25450	0.001092906	38.98022	0.004857321	4.92778		ubiquinol--cytochrome-c reductase-like protein ubiquinol--cytochrome-c reductase (EC 1.10.2.2) - Arabidopsis thaliana, PIR:T05357
AT2G41730	0.017066669	38.512753	0.002792891	16.12534		hypothetical protein predicted by genefinder
AT3G02800	0.000644954	34.25881	0.003779925	3.297418		unknown protein
AT2G21640	0.000542602	33.705643	0.006891858	18.648829		unknown protein
AT2G20560	0.0025333	32.95371	0.009193028	4.3273587		putative heat shock protein ;supported by full-length cDNA: Ceres:25528.
AT5G48570	0.003224576	32.293762	0.010823317	10.457688		peptidylprolyl isomerase
AT1G05680	0.01634421	30.495426	0.003357299	30.744236		putative indole-3-acetate beta-glucosyltransferase similar to indole-3-acetate beta-glucosyltransferase GB:AAD32293
AT3G49580	0.024441678	30.039654	0.001442748	16.516396		putative protein ;supported by full-length cDNA: Ceres:26235.
AT1G69930	0.015220757	30.032288	0.005060274	27.361132	ATGSTU11	putative glutathione transferase similar to glutathione transferase GB:CAA09188 [Alopecurus myosuroides]
AT4G26270	0.000858683	29.113058	0.012519204	4.3654456		pyrophosphate-dependent phosphofructo-1-kinase pyrophosphate-dependent phosphofructo-1-kinase, Prunus armeniaca, U93272; supported by cDNA: gi_15146207_gb_AY049245.1_
AT5G51440	0.008233422	26.9698	0.009418224	5.000131		mitochondrial heat shock 22 kd protein-like ; supported by full-length cDNA: Ceres: 268536.
AT3G21700	0.002359667	26.347706	0.002792891	2.3859348		putative SGP1 monomeric G-protein similar to GB:CAB54517 from [Arabidopsis thaliana];supported by full-length cDNA: Ceres:30989.
AT3G24500	0.004667494	26.04942	0.006460856	5.1495824	ATMBF1C	ethylene-responsive transcriptional coactivator, putative similar to GB:AAD46402 from [Lycopersicon esculentum] (Plant J. 18 (6), 589-600 (1999));supported by full-length cDNA: Ceres:158734.
AT4G12400	0.001950325	23.58999	0.010111201	4.2134852		stress-induced protein sti1 -like protein stress-induced protein sti1 -Glycine max,PID:g872116
AT3G28210	0.024435835	23.493662	0.005592544	13.273975	PMZ	zinc finger protein (PMZ), putative identical to putative zinc finger protein (PMZ) GB:AAD37511 GI:5006473 [Arabidopsis thaliana]
AT4G28390	0.000909821	23.060575	0.02217697	3.2167835	AAC3	ADP,ATP carrier-like protein ADP,ATP carrier protein-rice, PIR2:S33630;supported by full-length cDNA: Ceres:42036.
AT5G35320	0.000796205	23.000053	0.011047057	4.583494		putative protein ;supported by full-length cDNA: Ceres:38281.
AT5G15450	0.000245147	22.98054	0.00909261	2.601854	APG6	HSP100/ClpB, putative similar to HSP100/ClpB GI:9651530 [Phaseolus lunatus]
AT1G30070	0.000796205	22.43633	0.006206627	4.9448967		unknown protein ;supported by full-length cDNA: Ceres:270659.
AT1G73480	0.001167618	22.041246	0.03450541	3.744537		lysophospholipase homolog, putative similar to lysophospholipase homolog GI:2801536 from [Oryza sativa]; supported by cDNA: gi_15028212_gb_AY045929.1_
AT3G12580	0.00672625	21.900354	0.012977538	8.756469	HSP70	heat shock protein 70 identical to heat shock protein 70 GB:CAA05547 GI:3962377 [Arabidopsis thaliana]; supported by cDNA: gi_15809831_gb_AY054183.1_
AT4G33660	0.000311463	21.2332	0.005046397	5.0524025		Expressed protein ; supported by full-length cDNA: Ceres: 109432.
AT1G74360	0.006774846	20.726498	0.007637197	4.6798587		putative receptor protein kinase similar to brassinosteroid insensitive 1 GB:AAC49810 (putative receptor protein kinase); contains Pfam profiles: PF00560 Leucine Rich Repeat (17 repeats), PF00069 Eukaryotic protein kinase domain; supported by cDNA: gi_158
AT5G48850	0.030471845	20.67396	0.005060274	67.18814		putative protein similar to unknown protein (gblAAC72543.1)
AT1G22985	0.001302828	20.093536	0.012744497	2.624774		Expressed protein ; supported by full-length cDNA: Ceres: 31252.
AT2G43500	0.0426542	19.27055	0.005085019	22.752289		hypothetical protein predicted by genefinder
AT4G15120	0.00093031	18.794651	0.001810002	3.3142655		hypothetical protein ;supported by full-length cDNA: Ceres:36129.
AT4G21680	0.001982334	18.639536	0.0045258	11.761998		peptide transporter - like protein peptide transporter (ptr1) - Hordeum vulgare,AF023472
AT3G09410	0.000734108	18.406157	0.005151427	8.84384		putative pectinacetylesterase similar to pectinacetylesterase precursor GB:CAA67728 [Vigna radiata]
AT1G33110	0.003467166	17.068272	0.002792891	5.6554046		unknown protein
AT1G32940	0.007003678	16.73965	0.005847406	4.5894156	ATSBT3.5	subtilisin-like serine protease contains similarity to subtilase; SP1 GI:9957714 from [Oryza sativa]
AT4G15420	0.001105544	16.612692	0.0025088	4.0118685		UFD1 like protein ; supported by full-length cDNA: Ceres: 207652.
AT2G34500	0.001387465	16.595163	0.007051962	2.3245656	CYP710A1	putative cytochrome P450
AT1G36370	0.003552384	16.441391	0.003807531	8.291472	SHM7	putative hydroxymethyltransferase similar to serine hydroxymethyltransferage GB:P50433 from [Solanum tuberosum];supported by full-length cDNA: Ceres:122515.
AT1G56170	0.002142909	16.363232	0.01456871	3.8584595	HAP5B	transcription factor, putative similar to Transcription factor GB:CAA74053 GI:2398533 from [Arabidopsis thaliana];supported by full-length cDNA: Ceres:26157.
AT5G47830	0.00082477	16.009993	0.003531471	3.6727734		unknown protein
AT1G28190	0.001912657	15.773435	0.01764908	3.038323		hypothetical protein predicted by genemark.hmm
AT4G14680	0.000712948	15.127505	0.008902759	3.619927	APS3	ATP-sulfurylase ; supported by cDNA: gi_459143_gb_U06275.1_ATU06275
AT5G52640	0.006292802	15.0205	0.012977538	4.9299955	HSP81-1	heat-shock protein ; supported by cDNA: gi_166769_gb_M62984.1_ATHHSP83
AT1G32870	0.000470407	14.882087	0.003559839	6.5732327	ANAC013	NAM protein, putative similar to NAM protein GI:6066594 from [Petunia hybrida]; supported by cDNA: gi_14334571_gb_AY034959.1_
AT2G40340 /// AT	0.00700688	14.69672	0.002792461	5.5215964		AP2 domain transcription factor
AT2G22240	0.001402915	14.672411	0.004857321	2.1130645		putative myo-inositol 1-phosphate synthase ; supported by cDNA: gi_15450746_gb_AY053415.1_
AT2G33710	0.003068686	14.459347	0.005834543	3.8164232		putative AP2 domain transcription factor
AT1G05560	0.047921535	14.319082	0.001810002	20.453411	UGT1	UDP-glucose:indole-3-acetate beta-D-glucosyltransferase, putative similar to UDP-glucose:indole-3-acetate beta-D-glucosyltransferase GI:2149127 from (Arabidopsis thaliana); supported by cDNA: gi_13661274_gb_AF196777.1_AF196777
AT5G62020	0.000889151	14.284025	0.008786433	2.3091547	AT-HSFB2A	heat shock factor 6
AT1G55530	0.000945487	13.555468	0.004723555	2.0483563		hypothetical protein similar to RING-H2 finger protein RHC1a GB:AAC69854 GI:3790583 from [Arabidopsis thaliana]; supported by cDNA: gi_14517545_gb_AY039608.1_
AT5G09800	0.003890375	13.186417	0.015549773	2.4218974		putative protein various predicted proteins, Arabidopsis thaliana
AT2G29460	0.006409871	13.120436	9.92153E-05	8.043762	ATGSTU4	putative glutathione S-transferase ; supported by cDNA: gi_14423533_gb_AF387004.1_AF387004
AT3G17611	0.002741069	13.0765705	0.001495422	4.496536		hypothetical protein similar to hypothetical protein GB:CAB57979 from [Lycopersicon esculentum]
AT5G13080	0.001256445	12.948419	0.012700422	3.6305032	WRKY75	WRKY-like protein WRKY DNA binding protein - Solanum tuberosum, EMBL:AJ278507
AT1G26800	0.000608712	12.828422	0.009193028	2.8331447		hypothetical protein predicted by genscan+;supported by full-length cDNA: Ceres:250127.
AT2G25140	0.00026188	12.681578	0.016411927	2.7643929	CLPB-M	HSP100/ClpB, putative similar to HSP100/ClpB GI:9651530 [Phaseolus lunatus]
AT4G33666	0.013689891	12.215115	0.004351474	6.6011057		Expressed protein ; supported by full-length cDNA: Ceres: 38416.
AT1G76070	0.000909821	12.126417	0.005686936	2.374957		hypothetical protein predicted by genemark.hmm;supported by full-length cDNA: Ceres:39494.
AT3G19200	0.002647656	11.608624	0.027324757	2.962994		hypothetical protein predicted by genemark.hmm
AT3G48020	0.002701426	11.537107	0.004718718	2.80207		hypothetical protein
AT1G66080	0.000471695	11.525722	0.008838028	3.4977138		hypothetical protein
AT3G59080	0.002142909	11.501334	0.014548975	2.8922431		putative protein CND41, chloroplast nucleoid DNA binding protein - Nicotiana tabacum, EMBL:D26015; supported by cDNA: gi_15983375_gb_AF424562.1_AF424562
AT2G23320	0.00267055	11.477117	0.003783142	3.5820549	WRKY15	putative WRKY-type DNA-binding protein ; supported by cDNA: gi_13506742_gb_AF224704.1_AF224704
AT4G09150	0.001084539	11.466653	0.003689248	2.7655509		putative protein
AT3G09010	0.006353002	11.457114	0.00523089	2.5668051		putative receptor ser/thr protein kinase similar to receptor kinase GB:S70769 [Arabidopsis thaliana]; supported by full-length cDNA: Ceres: 124301.
AT1G35660	0.001307161	11.389222	0.015866674	2.5452518		unknown protein
AT1G76600	0.0025333	11.325969	0.002131859	5.258236		unknown protein ;supported by full-length cDNA: Ceres:29624.
AT1G24090	0.001581638	11.285892	0.015612933	2.3682191		unknown protein
AT1G67810	0.005681347	10.963973	0.01467628	7.9180627		F12A21.6 hypothetical protein
AT5G18040	0.000877574	10.888895	0.03231286	1.9400253		putative protein similar to unknown protein (pirllT09939);supported by full-length cDNA: Ceres:23754.
AT5G02780	0.003743039	10.716657	0.005486214	5.008062		putative protein In2, Zea mays, EMBL:X58573
AT1G60730	0.006880031	10.229778	0.006129834	7.6718106		auxin-induced protein, putative similar to auxin-induced atb2 GI:6562980 from [Arabidopsis thaliana]; supported by cDNA: gi_13605500_gb_AF361576.1_AF361576
AT1G55450	0.002060783	10.219311	0.03633175	2.0310924		hypothetical protein similar to embryo-abundant protein GB:L47672 GI:1350530 from [Picea glauca]; supported by cDNA: gi_14335021_gb_AY037190.1_
AT2G47520	0.003499105	10.187152	0.001992044	12.572565		putative AP2 domain transcription factor
AT3G17110	0.000822181	10.044105	0.029733595	2.859964		hypothetical protein
AT4G00550	0.001044592	10.033606	0.014004797	1.6757871	DGD2	predicted protein of unknown function
AT5G18470	0.004963383	9.979825	0.009193028	3.3810012		putative protein S-receptor kinase PK3 precursor, maize, PIR:T02753;supported by full-length cDNA: Ceres:154037.
AT1G18570	0.003274166	9.87029	0.019853469	2.5624964	MYB51	myb factor, putative similar to myb factor GI:1946266 from [Oryza sativa]; supported by cDNA: gi_3941465_gb_AF062887.1_AF062887
AT2G37430	0.007996882	9.818258	0.029733595	1.9329764		putative C2H2-type zinc finger protein likely a nucleic acid binding protein
AT2G15490	0.010184507	9.751528	0.014865178	5.410069	UGT73B4	putative glucosyltransferase
AT4G23570	0.000708953	9.730607	0.014052022	2.3226044	SGT1A	phosphatase like protein phosphoprotein phosphatase (EC 3.1.3.16) PPT - rat
AT2G43620	0.00996064	9.724916	0.011762494	3.55099		putative endochitinase
AT3G63380	0.027703678	9.696678	0.01189597	16.733858		Ca2+-transporting ATPase -like protein Ca2+-transporting ATPase, calmodulin-stimulated, wild cabbage, PIR:T14453
AT2G17900	0.000972307	9.657567	0.006678033	1.8101444	SDG37	putative SET-domain transcriptional regulator contains SET domain (PS50280; PF00856)
AT3G25900	0.000534029	9.594861	0.006200098	1.8930228	ATHMT-1	homocysteine S-methyltransferase AtHMT-1 identical to GB:AAF23821 from [Arabidopsis thaliana]
AT4G23190	0.004408076	9.542179	0.020383343	5.5095997	CRK11	serine/threonine kinase - like protein serine/threonine kinase, Brassica oleracea
AT2G32020	0.003308343	9.402495	0.002754528	6.68918		putative alanine acetyl transferase ;supported by full-length cDNA: Ceres:21201.
AT2G29450	0.007524552	9.367345	0.01910552	2.9835923	ATGSTU5	glutathione S-transferase identical to GB:X89216;supported by full-length cDNA: Ceres:6528.
AT5G58070	0.001331788	9.177923	0.00523089	4.71759		outer membrane lipoprotein - like outer membrane lipoprotein, Citrobacter freundii, PIR:I40710;supported by full-length cDNA: Ceres:17098.
AT1G03905	0.003171321	9.129602	0.024466237	2.6492767		unknown protein similar to NBD-like protein GB:AAD20643
AT4G30390	0.000734108	8.932392	0.009211053	2.3836586		putative protein ; supported by full-length cDNA: Ceres: 13139.
AT3G23170	0.001323655	8.905876	0.026795128	1.9538847		unknown protein ;supported by full-length cDNA: Ceres:92314.
AT4G27940	0.003060068	8.884243	0.00655279	3.162614		putative protein protein kinase LePK7, Lycopersicon esculentum, gb:U89684;supported by full-length cDNA: Ceres:147875.
AT3G47380	0.013138001	8.879975	0.006285763	4.4517636		putative protein pectinesterase homolog - Pinus radiata,PIR2:T08112
AT5G38900	0.008530006	8.858418	0.011270585	3.6852117		frnE protein - like frnE protein, Deinococcus radiodurans, PIR:E75491;supported by full-length cDNA: Ceres:10982.
AT3G55090	0.030512102	8.8476715	0.004294924	41.932697		ABC transporter - like protein ATP-binding cassette-sub-family G-member 2, Mus musculus, EMBL:AF140218
AT1G26420	0.026473703	8.778974	0.01502979	3.2641606		hypothetical protein similar to reticuline oxidase-like protein GB:CAB45850 GI:5262224 from [Arabidopsis thaliana]
AT3G53230	0.00422674	8.721958	0.003779925	3.6665065		CDC48 - like protein transitional endoplasmic reticulum ATPase, Arabidopsis thaliana, PIR:S60112
AT4G01630	0.004634941	8.664469	0.022753201	4.4591556	ATEXPA17	putative expansin
AT2G21940	0.000708953	8.652879	0.01873045	1.463894		putative shikimate kinase precursor
AT5G05410	0.007568589	8.469836	0.009211053	4.0743513	DREB2A	DREB2A (dbjlBAA33794.1) ; supported by cDNA: gi_3738229_dbj_AB007790.1_AB007790
AT3G26910	0.001743827	8.328714	0.005459929	2.9900117		unknown protein
AT3G16050	0.001353216	8.319357	0.025680445	1.9997506	A37	putative ethylene-inducible protein similar to ethylene-inducible protein GB:M88254 from [Hevea brasiliensis]; supported by cDNA: gi_4103951_gb_AF029980.1_AF029980
AT1G66690 /// AT	0.03281982	8.09392	0.006026509	5.9637747	PXMT1	unknown protein
AT1G72900	0.013457635	8.030582	0.003341037	6.087739		virus resistance protein, putative similar to virus resistance protein GI:558886 from [Nicotiana glutinosa]
AT5G56750	0.001100269	7.862225	0.030439291	2.661564		pollen specific protein SF21 ;supported by full-length cDNA: Ceres:36495.
AT2G30140	0.025202116	7.8478107	0.002145344	4.155505		putative glucosyltransferase
AT2G43570	0.002781657	7.714513	0.014753928	3.512283		endochitinase isolog
AT3G26980	0.001305777	7.694663	0.04608828	1.5145146	MUB4	geranylgeranylated protein, putative similar to ATGP4 GB:AAD00115 from [Arabidopsis thaliana]
AT3G50910	0.002813752	7.694597	0.013046356	2.6399696		putative protein ; supported by cDNA: gi_14517539_gb_AY039605.1_
AT1G01940	0.000287634	7.63313	0.003637845	1.9202101		hypothetical protein predicted by genemark.hmm
AT2G20900	0.001563541	7.5975337	0.034441717	1.6333756		putative diacylglycerol kinase ;supported by full-length cDNA: Ceres:15863.
AT3G57810	0.001080427	7.587575	0.012148861	2.4321978		putative protein ;supported by full-length cDNA: Ceres:29384., Ceres:101256.
AT5G37710	0.00026188	7.520488	0.014730349	1.7879428		calmodulin-binding heat-shock protein -like calmodulin-binding heat-shock protein, common tobacco, PIR:T04107
AT5G02230	0.002459923	7.464695	0.0143959	4.0185785		putative protein putative hydrolase At2g32150 - Arabidopsis thaliana, EMBL:AC006223;supported by full-length cDNA: Ceres:36724.
AT3G08760	0.000722313	7.3828735	0.004813203	2.351606	ATSIK	putative protein kinase contains Pfam profile: PF00069 Eukaryotic protein kinase domain (2 copies); supported by cDNA: gi_14581444_gb_AY026759.1_
AT2G36460	0.000858683	7.3622584	0.01137017	2.9999268		putative fructose bisphosphate aldolase ;supported by full-length cDNA: Ceres:13024.
AT1G10170	0.001708278	7.268069	0.002824896	6.24273	ATNFXL1	hypothetical protein similar to transcriptional repressor NF-X1 in Homo sapiens (splQ12986lNFX1_HUMAN); similar to EST gblT21002
AT4G03320	0.002116537	7.2352014	0.006927837	2.6575887	TIC20-IV	putative chloroplast protein import component similar to P. sativum Tic20 chloroplast protein import component, GenBank accession number AF095285;supported by full-length cDNA: Ceres:111207.
AT2G16900	0.00082477	7.2173133	0.008154522	2.9515014		hypothetical protein predicted by genefinder; supported by cDNA: gi_14532491_gb_AY039870.1_
AT3G14200	0.005728748	7.217087	0.019512951	1.702337		hypothetical protein predicted by genemark.hmm
AT2G15120 /// AT	0.011695122	7.162367	0.014720583	2.7396767		unknown protein
AT1G15430	0.001105544	7.1571813	0.004813203	1.7484375		hypothetical protein predicted by genscan+; supported by cDNA: gi_15293248_gb_AY051058.1_
AT5G64660	0.001787189	7.109759	0.04811496	1.6538552		putative protein strong similarity to unknown protein (emblCAB89350.1)
AT1G17170	0.02150356	7.0995274	0.007138968	3.533991	ATGSTU24	putative glutathione transferase One of three repeated putative glutathione transferases. 72% identical to glutathione transferase [Arabidopsis thaliana] (gil4006934)
AT2G41880	0.001466356	7.07903	0.04811496	1.6213237	GK-1	putative guanylate kinase ; supported by cDNA: gi_7861794_gb_AF204675.1_AF204675
AT4G36430	0.002963268	7.0356903	0.005592544	1.9977249		peroxidase like protein
AT5G35735	0.001327001	7.027627	0.016263295	1.6949842		Expressed protein ; supported by full-length cDNA: Ceres: 32450.
AT5G20910	0.003284511	6.9680734	0.002754528	2.7459292		ABI3-interacting protein 2 ;supported by full-length cDNA: Ceres:30983.
AT2G35730	0.003130757	6.959513	0.018098654	3.216781		Expressed protein ; supported by full-length cDNA: Ceres: 5598.
AT3G22840	0.001057559	6.9342084	0.010792479	3.6369483	ELIP1	early light-induced protein identical to early light-induced protein GB:AAB88391 from [Arabidopsis thaliana];supported by full-length cDNA: Ceres:14490.

AT1G08940	0.00355729	6.923542	0.00957574	1.6744338		unknown protein Similar to Saccharomyces hypothetical protein YDR051c (gb|Z49209). ESTs gb|T44436,gb|42252 come from this gene
AT4G11280	0.001749253	6.923268	0.030901639	1.6614738	ACS6	ACC synthase (AtACS-6) ; supported by cDNA: gi_16226285_gb_AF428292.1_AF428292
AT1G67970	0.001084539	6.9121294	0.027885096	2.3701916	AT-HSFA8	putative heat shock transcription factor contains Pfam profile: PF00447 HSF-type DNA-binding domain; N-terminal portion similar to heat shock transcription factor proteins: GB:CAA74397 [Arabidopsis thaliana], GB:S25478 [Lycopersicon esculentum]
AT1G29195	0.000787158	6.8243556	0.009211053	1.9071878		Expressed protein ; supported by full-length cDNA: Ceres: 39178.
AT3G12050	0.00104422	6.800877	0.021672826	1.862836		unknown protein ;supported by full-length cDNA: Ceres:38478.
AT4G38940	0.0008626	6.774174	0.00870844	1.6915754		putative protein more than 30 predicted proteins, Arabidopsis;supported by full-length cDNA: Ceres:40069.
AT1G19020	0.003060474	6.7413096	0.010031367	2.4272146		Expressed protein ; supported by full-length cDNA: Ceres: 31015.
AT1G56300	0.00079421	6.698275	0.02726845	3.446818		DnaJ protein, putative contains Pfam profile: PF00226: DnaJ domain;supported by full-length cDNA: Ceres:25796.
AT5G39040	0.00646568	6.692638	0.0087585	2.8646965	ATTAP2	ABC transporter -like protein TAP-like ABC transporter, Rattus norvegicus, EMBL:AB027520
AT4G36988 /// AT	0.001020155	6.688371	0.02057561	1.7860364	HSF4	heat shock transcription factor HSF4 ; supported by cDNA: gi_1619920_gb_U68017.1_ATU68017
AT1G08050	0.007874557	6.6607094	0.004723555	3.1708329		unknown protein
AT5G19855	0.004963383	6.508829	0.002754528	3.4412465		Expressed protein ; supported by full-length cDNA: Ceres: 42402.
AT2G15480	0.013727974	6.43081	0.008060786	4.0582786	UGT73B5	putative glucosyltransferase
AT4G05020	0.008267647	6.4204555	0.002145344	2.990145	NDB2	coded for by A. thaliana cDNA W43435 ; supported by cDNA: gi_14532463_gb_AY039856.1_
AT5G65300	0.000843909	6.3968587	0.008131621	1.7181101		unknown protein ; supported by cDNA: gi_13877834_gb_AF370180.1_AF370180
AT5G25930	0.00597662	6.3506045	0.002808379	3.2607408		receptor-like protein kinase - like receptor-like protein kinase 5, Arabidopsis thaliana, PIR:S27756
AT3G22370	0.007774778	6.3370113	0.002243847	4.676477	AOX1A	alternative oxidase 1a precursor identical to GB:Q39219 from [Arabidopsis thaliana];supported by full-length cDNA: Ceres:116257.
AT5G57910	0.001398458	6.325237	0.007175001	2.5634038		putative protein similar to unknown protein (emb|CAB79781.1);supported by full-length cDNA: Ceres:31005.
AT3G08690	0.001618378	6.313121	0.001810002	2.925943	UBC11	E2, ubiquitin-conjugating enzyme 11 (UBC11) identical to gi:12643427, SP:P35134;supported by full-length cDNA: Ceres:21455.
AT1G74590	0.004747035	6.22931	0.008154522	3.4928033	ATGSTU10	putative glutathione S-transferase similar to putative glutathione S-transferase GB:CAA10060 [Arabidopsis thaliana]; contains Pfam profile: PF00043 Glutathione S-transferases;supported by full-length cDNA: Ceres:40734.
AT4G11740	0.00126274	6.1958423	0.010031367	1.8644724	SAY1	putative protein Fly Fas-associated factor (FFAF), Drosophila melanogaster, AB013610; supported by cDNA: gi_14596000_gb_AY042788.1_
AT1G62180	0.00881536	6.158038	0.003027713	3.0113497	40270	5'-adenylylphosphosulfate reductase, putative similar to 5'-adenylylphosphosulfate reductase GI:1336168 from [Arabidopsis thaliana];supported by full-length cDNA: Ceres:9002.
AT1G05060	0.001563781	6.1502156	0.03154573	2.2845755		hypothetical protein predicted by genscan;supported by full-length cDNA: Ceres:37455.
AT3G25250	0.007685649	6.1321654	0.004813203	3.6136935	AGC2-1	protein kinase, putative contains Pfam profile: PF00069 Eukaryotic protein kinase domain
AT5G19875	0.000881389	6.100293	0.007637197	2.490947		Expressed protein ; supported by full-length cDNA: Ceres: 59.
AT5G51830	0.005010757	6.0772004	0.00464869	2.7406878		fructokinase 1 ; supported by cDNA: gi_13878052_gb_AF370289.1_AF370289
AT3G07370	0.001578597	6.0127196	0.008618869	2.121748	ATCHIP	hypothetical protein similar to serologically defined colon cancer antigen 7 GB:5031963 [Homo sapiens]; supported by cDNA: gi_14596038_gb_AY042807.1_
AT3G01650	0.000757088	5.982498	0.005191353	2.2950904	RGLG1	unknown protein similar to hypothetical protein GB:AAD30238 [Arabidopsis thaliana], contains zinc finger domain, C3HC4 type (RING finger)
AT1G57980 /// AT	0.002859793	5.9714127	0.014678935	2.9489338	ATPUP18	unknown protein
AT1G07350	0.002559056	5.9504557	0.025271554	1.9904466		transformer-SR ribonucleoprotein, putative similar to GB:Y09506 from [Nicotiana tabacum] (Plant Mol. Biol. 35 (3), 261-269 (1997)); supported by cDNA: gi_15292732_gb_AY050800.1_
AT4G01870	0.02150356	5.869762	0.001810002	7.280087		predicted protein of unknown function similar to bacterial tolB proteins but unclear if T7B11.13 is involved in viral transport
AT2G38470	0.007778357	5.8347106	0.012645463	2.4252894	WRKY33	putative WRKY-type DNA binding protein
AT4G17500	0.003059956	5.815963	0.001810002	1.4761107	ATERF-1	ethylene responsive element binding factor 1 (frameshift !) ; supported by cDNA: gi_3434966_dbj_AB008103.1_AB008103
AT3G05360	0.010873992	5.7833385	0.0025088	8.808497		putative disease resistance protein similar to Cf-2 disease resistance protein GB:AAC15780 from [Lycopersicon pimpinellifolium]
AT2G41160	0.001440628	5.760647	0.009193028	2.2558112		unknown protein
AT3G26980	0.000630096	5.7470655	0.04608828	1.5145146	MUB4	geranylgeranylated protein, putative similar to ATGP4 GB:AAD00115 from [Arabidopsis thaliana]
AT1G13990	0.001557723	5.7404447	0.01109956	2.259651		unknown protein ;supported by full-length cDNA: Ceres:93312.
AT1G68450	0.018119657	5.7369075	0.03707499	1.7852197		unknown protein
AT2G39650	0.00205078	5.7368617	0.003341037	2.3651538		unknown protein
AT3G11820	0.000909821	5.730924	0.009211053	1.8152895	SYP121	putative syntaxin contains Pfam profile: PF00804 syntaxin;supported by full-length cDNA: Ceres:38899.
AT1G74750	0.012686064	5.7178	0.012432217	2.6773546		hypothetical protein predicted by genemark.hmm
AT1G22280	0.000910835	5.702435	0.00655279	1.8435355		protein phosphatase type 2C, putative similar to protein phosphatase type 2C GI:4336436 from [Lotus japonicus];supported by full-length cDNA: Ceres:119977.
AT2G43630	0.000851369	5.702243	0.028360214	1.5365561		unknown protein predicted by genefinder
AT4G22530	0.012947262	5.669963	0.001817905	4.1268563		putative protein embryo-abundant protein (EMB34), Picea glauca, PID:g1350531
AT4G25390	0.002172168	5.654144	0.011154271	2.601488		receptor kinase-like protein receptor-like protein kinase, RLK3 - Arabidopsis thaliana,PID:e1363211
AT1G61370	0.000905666	5.644454	0.007220829	1.9773818		receptor protein kinase (IRK1), putative similar to receptor protein kinase (IRK1) GI:836953 from [Ipomoea trifida]
AT2G22880	0.010988856	5.6160583	0.015793946	1.6883855		hypothetical protein predicted by genefinder
AT3G22930	0.001573711	5.599487	0.004883206	2.77272		calmodulin, putative similar to calmodulin GB:AAA34015 [Glycine max]; supported by cDNA: gi_16226343_gb_AF428309.1_AF428309
AT4G01960	0.001562901	5.575146	0.010823317	1.8645436		predicted protein of unknown function ;supported by full-length cDNA: Ceres:13256.
AT5G53970	0.006275633	5.549804	0.012977538	2.7374244		tyrosine aminotransferase
AT1G09940	0.00346449	5.5372143	0.032179214	1.6038649	HEMA2	putative glutamyl-tRNA reductase 2 precursor similar to GB:P49294 and to A. thaliana HEMA2 (gb|U27118)
AT5G23490 /// AT	0.001742449	5.515984	0.018755503	1.411521		putative protein similar to unknown protein (gb AAF01580.1)
AT1G53990	0.002968766	5.511708	0.016187336	2.1660986	GLIP3	lipase, putative similar to lipase GI:1145627 from [Arabidopsis thaliana]
AT1G06570	0.001140098	5.4537516	0.004857321	2.5699785	PDS1	4-hydroxyphenylpyruvate dioxygenase (HPD) identical to 4-hydroxyphenylpyruvate dioxygenase (HPD) SP:P93836 [Arabidopsis thaliana (Mouse-ear cress)]; supported by cDNA: gi_2145038_gb_AF000228.1_ATAF000228
AT5G27760	0.00348535	5.420125	0.003064734	2.5793848		putative protein predicted protein, Arabidopsis thaliana; supported by full-length cDNA: Ceres: 108472.
AT3G15770	0.001225199	5.36305	0.018233245	1.8759767		hypothetical protein
AT2G19460	0.00141773	5.3598127	0.001099773	3.1671228		hypothetical protein predicted by genefinder; supported by cDNA: gi_14334633_gb_AY034990.1_
AT5G22270	0.026436593	5.3566923	0.001099773	2.9641564		putative protein similar to unknown protein (gb|AAF02129.1);supported by full-length cDNA: Ceres:35419.
AT3G61630	0.011024843	5.3501983	0.005459929	9.42608	CRF6	putative protein transcription factor Pti6 - Lycopersicon esculentum, PIR:T07728;supported by full-length cDNA: Ceres:100688.
AT5G43450	0.006478163	5.2521577	0.008131621	7.2099986		1-aminocyclopropane-1-carboxylate oxidase ; supported by cDNA: gi_14596164_gb_AY042870.1_
AT5G11100	0.000471695	5.251591	0.034766145	1.8868402	ATSYTD	CLB1 - like protein CLB1 protein, Lycopersicon esculentum, PIR:T04143
AT2G41170	0.001791029	5.2156096	0.017854294	2.652405		unknown protein
AT4G31860	0.001579277	5.2104955	0.004255812	2.481961		protein phosphatase 2C - like protein protein phosphatase 2C, Schizosaccharomyces pombe, PIR2:S54297; supported by cDNA: gi_16209697_gb_AY057611.1_
AT3G13430	0.001387465	5.2063174	0.040928684	1.6441813		unknown protein contains Pfam profile: PF00097 Zinc finger, C3HC4 type (RING finger)
AT2G03430	0.000822181	5.2036185	0.02482941	1.718265		putative ankyrin
AT4G36500	0.002502246	5.184551	0.003654565	2.3548484		putative protein
AT2G29990	0.002451659	5.1784625	0.041927945	4.572731	NDA2	putative NADH dehydrogenase (ubiquinone oxidoreductase)
AT2G30130	0.00174245	5.1288705	0.04143313	2.099409	ASL5	hypothetical protein predicted by genscan and genefinder; supported by full-length cDNA: Ceres: 29970.
AT3G07090	0.002011951	5.090347	0.004723555	2.1130183		unknown protein
AT2G23420	0.001562901	5.046167	0.001810002	3.485524		unknown protein
AT4G15110	0.000424419	5.04235	0.04390554	1.5838691	CYP97B3	cytochrome P450 like protein
AT1G30410 /// AT	0.002535919	5.029305	0.02127979	1.6058626	ATMRP13	ABC transporter, putative contains Pfam profile: PF00005: ABC transporter
AT4G11360	0.001005777	5.0074396	0.006285763	1.7834704	RHA1B	RING-H2 finger protein RHA1b ;supported by full-length cDNA: Ceres:37097.
AT1G02850	0.029454969	5.0026	0.00614898	6.65686		beta-glucosidase, putative similar to beta-glucosidase GI:5030906 from [Polygonum tinctorium]; supported by cDNA: gi_15146265_gb_AY049274.1_
AT5G39090	0.002141383	4.9605017	0.010111201	2.5638225		acyltransferase -like protein anthocyanin acyltransferase, Perilla frutescens, EMBL:AB029340
AT3G04710	0.000905666	4.9317565	0.01513091	1.4378417		ankyrin-like protein similar to ankyrin proteins: GB:CAA48803 [Mus musculus], GB:CAA40279 [Homo sapiens], GB:AAB47551 [Rattus norvegicus]; Pfam HMM hit: Ank repeat;supported by full-length cDNA: Ceres:104284.
AT5G38895	0.000882553	4.922811	0.03232837	1.5274475		Expressed protein ; supported by cDNA: gi_14334947_gb_AY035147.1_
AT4G21510	0.000827021	4.912649	0.015549773	2.581565		hypothetical protein ;supported by full-length cDNA: Ceres:36892.
AT4G15550	0.02488472	4.865418	0.005967305	3.8040023	IAGLU	glucosyltransferase like protein ; supported by cDNA: gi_2149126_gb_U81293.1_ATU81293
AT1G66590 /// AT	0.00082477	4.8500686	0.021690784	1.6103892		hypothetical protein predicted by genefinder
AT1G09140	0.002020568	4.8481755	0.011905072	2.6505113	ATSRP30.1	putative SF2/ASF splicing modulator, Srp30 similar to GB:CAB42558
AT2G21130	0.000909821	4.812939	0.039218377	1.477206		cyclophilin (CYP2) Same as GB: AF020434;supported by full-length cDNA: Ceres:35643.
AT5G03490	0.019005341	4.809644	0.005459929	3.1537395		putative protein
AT1G12200	0.006046396	4.7326474	0.00614898	2.5796251		unknown protein similar to unknown protein GB:AAF34848 GI:6997186 from [Arabidopsis thaliana]
AT4G21990	0.016709248	4.7127633	0.002808379	4.565843	40271	PRH26 protein ;supported by full-length cDNA: Ceres:36866.
AT2G26070	0.001450998	4.70506	0.009211053	1.8953685	RTE1	hypothetical protein predicted by genscan
AT5G48180	0.004408076	4.692685	0.007151878	3.2168124		putative protein contains similarity to jasmonate inducible protein;supported by full-length cDNA: Ceres:19110.
AT4G30210	0.001529735	4.6027627	0.005264052	1.4737931	ATR2	NADPH-ferrihemoprotein reductase (ATR2)
AT4G36010	0.011376068	4.600115	0.004813203	2.4929636		thaumatin-like protein thaumatin-like protein, Arabidopsis thaliana, Pir2:S71175; supported by cDNA: gi_13430505_gb_AF360165.1_AF360165
AT3G03270	0.00026188	4.591512	0.03850095	1.966454		unknown protein ; supported by full-length cDNA: Ceres: 118261.
AT5G26760	0.000547957	4.5752945	0.006556409	2.1919413		putative protein
AT1G25275	0.00174245	4.57022	0.008060786	1.6561023		Expressed protein ; supported by full-length cDNA: Ceres: 21980.
AT3G55430	0.001057559	4.528353	0.007051962	1.9962955		beta-1,3-glucanase - like protein probable beta-1,3-glucanase, Triticum aestivum, PIR:T06268; supported by full-length cDNA: Ceres: 8980.
AT5G13750	0.02874122	4.495829	0.003039575	5.378085	ZIFL1	transporter-like protein ;supported by full-length cDNA: Ceres:27439.
AT4G10955 /// AT	0.003115624	4.4739356	0.008800991	2.557326	UGE5	UDP-galactose 4-epimerase - like protein UDP-galactose 4-epimerase, Cyamopsis tetragonoloba, AJ005082;supported by full-length cDNA: Ceres:15608.
AT1G42990	0.001744274	4.4703197	0.004813203	2.2766745	ATBZIP60	bZIP transcription factor, putative contains Pfam profile: PF00170: bZIP transcription factor; supported by cDNA: gi_15028322_gb_AY045964.1_
AT2G03760	0.003222619	4.3897696	0.001224887	8.924994	ST	putative steroid sulfotransferase ; supported by cDNA: gi_599639_emb_Z46823.1_ATTS4391
AT5G13200	0.012037558	4.387305	0.037199095	1.955931		ABA-responsive protein - like ABA-responsive protein, Hordeum vulgare, EMBL:AF026538
AT4G32440	0.000590764	4.3728666	0.012842812	1.6362481		putative protein predicted protein, Arabidopsis thaliana, gb:AC002337
AT4G25130	0.001204418	4.2791886	0.018928252	1.7793951		protein-methionine-S-oxide reductase ;supported by full-length cDNA: Ceres:36536.
AT1G30700	0.016434489	4.2274823	0.007792773	3.2392075		putative reticuline oxidase-like protein similar to GB:P30986 from [Eschscholzia californica] (berberine bridge-forming enzyme), ESTs gb|F19886, gb|Z30784 and gb|Z30785 come from this gene; supported by cDNA: gi_16930506_gb_AF419607.1_AF419607
AT2G26530	0.002743091	4.2121563	0.036666166	1.538531	AR781	AR781, similar to yeast pheromone receptor identical to GB:D88743, corrected a frameshift found in the original record (at 69530 bp), sequence submitted has been verified from 10 sequence electropherograms. The translation now starts from an upstream ATG.
AT3G63310	0.002460952	4.2020564	0.00523089	2.316115		putative protein S1R protein - Homo sapiens, EMBL:AF113127
AT1G72940	0.019152671	4.1945424	0.002354162	3.8270748		disease resistance protein, putative similar to disease resistance protein GI:9758876 from [Arabidopsis thaliana]
AT2G32190 /// AT	0.002483074	4.1846642	0.041404147	1.7367222		unknown protein ; supported by full-length cDNA: Ceres: 40344.
AT4G24380	0.001540727	4.1774125	0.004857321	2.4825797		putative protein dihydrofolate reductase - Schizosaccharomyces pombe,PID:e1320950;supported by full-length cDNA: Ceres:27155.
AT5G40690	0.002950685	4.1611137	0.001810002	3.912769		putative protein predicted proteins, Arabidopsis thaliana;supported by full-length cDNA: Ceres:11539.
AT3G25610	0.003550869	4.1040187	0.048414003	2.1913593		ATPase II, putative similar to GB:AAD34706 from [Homo sapiens] (Biochem. Biophys. Res. Commun. 257 (2), 333-339 (1999))
AT5G02490	0.003847103	4.091026	0.015866674	2.5220516		dnaK-type molecular chaperone hsc70.1 - like dnaK-type molecular chaperone hsc70.1, Arabidopsis thaliana, PIR:S46302
AT1G62300	0.011161139	4.089654	0.006617936	2.7867045	WRKY6	unknown protein similar to putative DNA-binding protein GI:7268215 from [Arabidopsis thaliana]; supported by cDNA: gi_12658409_gb_AF331712.1_AF331712
AT3G22160	0.006515347	4.080265	0.010978133	1.9976082		unknown protein ;supported by full-length cDNA: Ceres:8081.
AT4G04610	0.00231819	4.0766215	0.003341037	2.665911	40269	5-adenylylsulfate reductase ;supported by full-length cDNA: Ceres:40330.
AT2G41100	0.009003662	4.062367	0.015793946	4.560125	TCH3	calmodulin-like protein identical to GB:D45848; supported by cDNA: gi_15983405_gb_AF424577.1_AF424577
AT5G35200	0.001507366	4.0618625	0.025302233	1.5260396		putative protein clathrin assembly protein short form, Rattus norvegicus, EMBL:AF041373
AT1G66880	0.017146872	4.047913	0.00495198	3.202141		protein kinase, putative contains Pfam profile: PF00069: Eukaryotic protein kinase domain
AT4G15130	0.003063447	4.0243344	0.010057126	1.8108155		putative phosphocholine cytidylyltransferase
AT1G75270	0.006035101	4.02002	0.005686936	2.3186395	DHAR2	GSH-dependent dehydroascorbate reductase 1, putative similar to GI:6939839 from [Oryza sativa];supported by full-length cDNA: Ceres:35711.
AT3G06250	0.009433251	4.010609	0.04119606	2.212893	FRS7	unknown protein similar to far-red impaired response protein GB:AAD51282 [Arabidopsis thaliana]; supported by cDNA: gi_13430715_gb_AF360270.1_AF360270
AT5G64250	0.020908365	3.9877963	0.001810002	5.42497		2-nitropropane dioxygenase-like protein ;supported by full-length cDNA: Ceres:207555.
AT2G31260	0.001618492	3.9800444	0.002890685	2.1334946	APG9	unknown protein
AT5G55700	0.000476072	3.9442213	0.009937104	1.8570061	BMY6	beta-amylase ; supported by cDNA: gi_15027954_gb_AY045834.1_
AT5G42220	0.000667495	3.925874	0.003559839	1.7024632		putative protein similar to unknown protein (pir||T30561)
AT2G28570	0.003686076	3.9193926	0.040867075	1.8635544		unknown protein ;supported by full-length cDNA: Ceres:36244.
AT5G09590	0.000572085	3.9142442	0.024485148	1.4882586	mtHSC70-2	heat shock protein 70 (Hsc70-5) ; supported by cDNA: gi_6746589_gb_AF217458.1_AF217458
AT5G46710	0.002575971	3.8912964	0.009211053	3.1072104		putative protein similar to unknown protein (pir||T05076);supported by full-length cDNA: Ceres:42747.
AT3G04010	0.001565076	3.8731725	0.005121905	1.6721939		putative beta-1,3-glucanase similar to beta-1,3-glucanase GB:S12402 [Nicotiana sp], GB:CAA03908 [Citrus sinensis], GB:S44364 [Lycopersicon esculentum]
AT1G13195	0.001562901	3.864351	0.004800609	2.250805		Expressed protein ; supported by full-length cDNA: Ceres: 36399.
AT2G38870	0.000999124	3.8553689	0.039202303	1.560125		putative protease inhibitor ;supported by full-length cDNA: Ceres:11662.
AT5G65240	0.009091699	3.8544903	0.014768215	1.9601941		receptor-like protein kinase
AT3G21630	0.001993154	3.8233132	0.009211053	1.464881	CERK1	putative protein kinase similar to serine/threonine protein kinase Pto GB:AAB47421 [Lycopersicon esculentum] (Plant Cell 9 (1), 61-73 (1997))
AT1G67360	0.001713485	3.8030398	0.032103967	1.6387762		stress related protein, putative similar to stress related protein GI:5802955 from [Vitis riparia];supported by full-length cDNA: Ceres:14043.
AT2G15760	0.021154236	3.8026793	0.0279809	1.6009247		hypothetical protein ;supported by full-length cDNA: Ceres:5.
AT3G57880	0.002892027	3.8012354	0.005521644	2.7598932		anthranilate phosphoribosyltransferase-like protein anthranilate phosphoribosyltransferase - Pisum sativum, PIR:T06460; supported by cDNA: gi_14030690_gb_AF375436.1_AF375436
AT5G08350	0.005826406	3.77179	0.040020224	2.4236143		putative protein FH protein interacting protein FIP1, Arabidopsis thaliana, EMBL:AF174428;supported by full-length cDNA: Ceres:19508.
AT2G37760	0.004453586	3.743002	0.008618869	1.6582278		putative alcohol dehydrogenase ; supported by cDNA: gi_16604706_gb_AY059798.1_
AT1G63440	0.011445848	3.729112	0.001810002	2.8072526	HMA5	ATP dependent copper transporter, putative similar to ATP dependent copper transporter GB:AAD29109 GI:4760370 [Arabidopsis thaliana]
AT4G22740	0.000757088	3.7147589	0.023780236	1.7791928		Expressed protein ; supported by full-length cDNA: Ceres: 20948.
AT3G23920	0.001380261	3.711792	0.005686936	1.8778027	BMY7	beta-amylase, putative similar to beta-amylase GB:CAB58423 [Arabidopsis thaliana]; supported by cDNA: gi_14194172_gb_AF367293.1_AF367293
AT5G57510	0.007524988	3.7114735	0.032103967	1.4438672		unknown protein
AT2G19450	0.003832129	3.7076342	0.003973629	2.380555	TAG1	diacylglycerol O-acyltransferase ; supported by cDNA: gi_15450799_gb_AY054480.1_
AT2G39350	0.006530769	3.694496	0.006885885	4.2735066		putative ABC transporter
AT4G04620	0.002371014	3.6600225	0.023593634	1.8619728	ATG8B	putative symbiosis-related protein ;supported by full-length cDNA: Ceres:119712.
AT2G30550	0.001259501	3.656799	0.042459674	2.6504567		putative lipase ; supported by full-length cDNA: Ceres: 207043.
AT1G33590	0.005041556	3.6558754	0.018542966	2.3712652		hypothetical protein similar to putative disease resistance protein GB:AAC14512 GI:2739389 from [Arabidopsis thaliana];supported by full-length cDNA: Ceres:105146.
AT3G13650	0.000972307	3.6372836	0.015046874	1.6441295		dirigent protein, putative similar to dirigent protein GB:AAF25365 from [Thuja plicata]
AT1G34260	0.001167618	3.6329613	0.005044704	1.6933972		hypothetical protein predicted by genemark.hmm
AT1G55920	0.014784975	3.5876799	0.009418224	2.6110933	AtSerat2;1	serine acetyltransferase identical to GB:CAA84371 from [Arabidopsis thaliana] (Eur. J. Biochem. 227 (1-2), 500-509 (1995)); supported by cDNA: gi_926938_gb_L42212.1_ATHSAT1G
AT4G20830	0.021869462	3.559227	0.004425827	2.7878697		reticuline oxidase -like protein reticuline oxidase, Eschscholzia californica, PIR:A41533; supported by cDNA: gi_15983492_gb_AF424621.1_AF424621
AT5G13330	0.048630085	3.5386696	0.03807527	2.0117412	RAP2.6L	putative protein AP2 domain containing protein RAP2.6, Arabidopsis thaliana, EMBL:AF003099
AT2G47000	0.009116313	3.5166118	0.000612937	3.3320487	MDR4	putative ABC transporter related to multi drug resistance proteins and P-glycoproteins
AT5G39610	0.03787985	3.501431	0.01994895	3.4927855	ANAC092	NAM / CUC2 - like protein CUC2, Arabidopsis thaliana, EMBL:ATAB2560;supported by full-length cDNA: Ceres:113779.
AT5G14040 /// AT	0.006070661	3.499188	0.004857321	2.032302	ATGCH	GTP cyclohydrolase II; 3,4-dihydroxy-2-butanone-4-phoshate synthase (emb|CAA03884.1) supported by cDNA: gi_940382_dbj_D45165.1_ATHGTPCII
AT5G63790	0.02309942	3.4984243	0.006582444	3.3951726	ANAC102	putative protein contains similarity to NAC-domain protein;supported by full-length cDNA: Ceres:6637.
AT4G24160	0.0123502	3.4884481	0.002854609	3.192145		putative protein CGI-58 protein - Homo sapiens,PID:g4929585
AT5G62520	0.00240443	3.4854274	0.044646982	3.2297592	SRO5	putative protein similar to unknown protein (pir||T07711);supported by full-length cDNA: Ceres:23920.
AT3G09440	0.003403394	3.480867	0.006363932	2.3630974		heat-shock protein (At-hsc70-3) identical to (At-hsc70-3) (cytosolic Hsp70) GB:CAA76606 [Arabidopsis thaliana]; supported by cDNA: gi_15292924_gb_AY050896.1_
AT3G21690	0.048208486	3.4752479	0.004624899	4.6950874		integral membrane protein, putative contains Pfam profile: PF01554 uncharacterized membrane protein family
AT1G02920 /// AT	0.011208107	3.4354463	0.005737556	3.4030962	ATGSTF7	glutathione S-transferase, putative similar to glutathione S-transferase GI:860955 from [Hyoscyamus muticus]; supported by cDNA: gi_15215607_gb_AY050332.1_
AT4G33540	0.004689048	3.4326422	0.003341037	2.6197655		putative protein
AT2G47710	0.002269515	3.4324977	0.00562872	1.7345293		unknown protein ;supported by full-length cDNA: Ceres:2847.
AT5G66880	0.001611023	3.4205697	0.041825876	1.4786288	SNRK2-3	protein kinase, 41K (EC 2.7.1.-) (pir||S71172) ; supported by cDNA: gi_166818_gb_L05562.1_ATHPROKINB
AT5G53400	0.000787158	3.4187388	0.009235052	1.4267327		putative protein contains similarity to nuclear movement protein nudC;supported by full-length cDNA: Ceres:38105.
AT4G04800	0.005260802	3.4146485	0.02042957	1.7272323		putative protein similar to transcriptional repressor;supported by full-length cDNA: Ceres:39698.
AT1G63090	0.002545516	3.4129992	0.027620252	1.5209233	ATPP2-A11	unknown protein
AT3G54950	0.007386654	3.392267	0.01168574	1.8833853	PLA IIIA	putative protein patatin, Solanum tuberosum, PIR2:A29810
AT3G29400	0.001990669	3.3825605	0.020610642	1.5185513	ATEXO70E1	hypothetical protein predicted by genemark.hmm
AT4G13180	0.013403568	3.3751235	0.01561967	2.5424325		short-chain alcohol dehydrogenase like protein short-chain alcohol dehydrogenase -Picea abies, PIR2:S34678;supported by full-length cDNA: Ceres:748.
AT4G27680	0.0007381	3.3667576	0.009807889	1.5427109		putative protein MSP1 protein, Saccharomyces cerevisia, PIR2:A49506;supported by full-length cDNA: Ceres:142160.
AT1G09100	0.000843909	3.3629904	0.049827304	1.4015555	RPT5B	putative 26S protease regulatory subunit 6A Similar to probable Mg-dependent ATPase (pir|S56671). ESTs gb|T46782,gb|AA04798 come from this gene; supported by cDNA: gi_15215787_gb_AY050423.1_
AT4G25670 /// AT	0.00093031	3.354905	0.033089496	2.068701		hypothetical protein ; supported by cDNA: gi_14517537_gb_AY039604.1_
AT5G03030	0.005724885	3.3546615	0.008216102	1.7939476		DNAJ protein - like DNAJ domain-containing protein, Homo sapiens, EMBL:AF126743; supported by full-length cDNA: Ceres: 19432.
AT5G44090	0.001866582	3.3415306	0.025274463	1.7962979		protein phosphatase 2A 62 kDa B regulatory subunit (gb AAD45158.1) ; supported by cDNA: gi_5533378_gb_AF165429.1_AF165429
AT2G29480	0.017952483	3.3363395	0.001810002	6.5522604	ATGSTU2	putative glutathione S-transferase ; supported by cDNA: gi_11096001_gb_AF288184.1_AF288184
AT4G21580	0.002467695	3.3358843	0.005603423	1.7558168		putative NADPH quinone oxidoreductase Pig3 Homo sapiens, PID:G2754812;supported by full-length cDNA: Ceres:4621.

AT4G35860	0.001437696	3.3333259	0.010111201	1.6855295	ATGB2	GTP-binding protein GB2 ;supported by full-length cDNA: Ceres:17725.
AT5G51460	0.002283728	3.326084	0.028123936	2.0554028	ATTPPA	trehalose-6-phosphate phosphatase ; supported by cDNA: gi_2944177_gb_AF007778.1_AF007778
AT2G27580	0.000827021	3.3192258	0.021125508	1.4824126		putative zinc finger protein ;supported by full-length cDNA: Ceres:14203.
AT4G08320	0.00178796	3.288932	0.006585094	1.4111686		putative protein (fragment) glutamine-rich tetratricopeptide repeat (TPR) containing protein (SGT) - Rattus norvegicus,PID:e1285298
AT1G02400	0.011277421	3.2884407	0.01502979	1.9110034	ATGA2OX6	dioxygenase, putative similar to dioxygenase GI:1666096 from [Marah macrocarpus]
AT4G39670	0.004849955	3.2733302	0.004813203	2.3981562		putative protein
AT5G59820	0.009489067	3.2692618	0.01033256	1.8939687	RHL41	zinc finger protein Zat12 ;supported by full-length cDNA: Ceres:40576.
AT2G33700	0.003607981	3.2657604	0.028448531	1.5238624		putative protein phosphatase 2C
AT4G34131 /// A	0.04629548	3.2587414	0.005571554	3.8549662	UGT73B2	glucosyltransferase -like protein immediate-early salicylate-induced glucosyltransferase, Nicotiana tabacum, PIR2:T03747;supported by cDNA gi:14334981
AT1G10960	0.005451453	3.249995	0.019490035	1.6557564	ATFD1	ferredoxin precusor isolog ;supported by full-length cDNA: Ceres:20637.
AT4G17615	0.006716564	3.2451632	0.007676405	1.5202327	CBL1	calcineurin B-like protein 1 ; supported by cDNA: gi_3309081_gb_AF076251.1_AF076251
AT3G13910	0.004545327	3.2419353	0.007876929	2.0715015		hypothetical protein predicted by genscan+supported by full-length cDNA: Ceres:17883.
AT2G35900	0.002359667	3.2274098	0.04131575	2.1548634		hypothetical protein predicted by genefinder
AT3G50260	0.013788533	3.2203612	0.010966293	2.7634714	ATERF#011	putative protein EREBP-3 homolog, Stylosanthes hamata, EMBL:U91982; supported by cDNA: gi_15010555_gb_AY045579.1_
AT4G35110	0.00204376	3.214319	0.024132153	1.4441714		putative protein pEARLI 4, Arabidopsis thaliana, PATCHX:G871782; supported by cDNA: gi_14326526_gb_AF385717.1_AF385717
AT3G12520	0.010221995	3.2028604	0.009727794	3.0792313	SULTR4;2	sulphate transporter, putative similar to high affinity sulphate transporter GB:X96431 GI:1217966 [Hordeum vulgare]; supported by cDNA: gi_11933424_dbj_AB052775.1_AB052775
AT1G56120 /// A	0.00392374	3.184307	0.006206627	1.5872374		wall-associated kinase 2, putative similar to receptor-like serine/threonine kinase GB:AAC50043 GI:2465923 from [Arabidopsis thaliana]
AT5G51740	0.001142363	3.184089	0.045082368	1.4915662		putative protein contains similarity to peptidase
AT2G27200	0.002061708	3.1838436	0.016362675	1.890985		putative nucleotide-binding protein
AT1G75280	0.006583484	3.1583445	0.003341037	3.7522888		NADPH oxidoreductase, putative similar to GI:1708420 from [Arabidopsis thaliana] (J. Biol. Chem. 270 (44), 26224-26231 (1995));supported by full-length cDNA: Ceres:11944.
AT3G26450	0.005291885	3.15833	0.015931785	3.5542526		major latex protein, putative similar to major latex protein type1 GB:CAA63026 [Arabidopsis thaliana]; supported by full-length cDNA: Ceres: 8841.
AT1G32920	0.002652926	3.1531074	0.010334739	1.7226404		unknown protein ; supported by cDNA: gi_15450636_gb_AY052686.1_
AT3G28740	0.04905238	3.1479602	0.01166408	2.823098		cytochrome P450, putative contains Pfam profile: PF00067 cytochrome P450; supported by cDNA: gi_15292830_gb_AY050849.1_
AT1G66760	0.003286153	3.1184726	0.008030331	2.7989445		MATE efflux family protein, putative contains TIGRfam profile: TIGR00797: MATE efflux family protein
AT5G04760	0.002718624	3.1169524	0.030921742	1.9227521		I-box binding factor - like protein I-box binding factor (LeMYBI gene), Lycopersicon esculentum, EMBL:LES243339;supported by full-length cDNA: Ceres:6170.
AT3G08590	0.000624251	3.1119323	0.002145344	2.026129		putative 2,3-bisphosphoglycerate-independent phosphoglycerate mutase similar to 2,3-bisphosphoglycerate-independent phosphoglycerate mutase GB:Q42908 [Mesembryanthemum crystallinum];supported by full-length cDNA: Ceres:125616.
AT4G26400	0.000471695	3.1008077	0.029206607	1.6885422		putative protein RING zinc finger protein, Gallus gallus
AT1G32530	0.000822181	3.098868	0.005567558	1.6913619		unknown protein
AT4G11850	0.000722973	3.0976381	0.028936565	1.5030146	PLDGAMMA1	putative phospholipase D-gamma phospholipase D-gamma - Arabidopsis thaliana,PID:g2653885; supported by cDNA: gi_2653884_gb_AF027408.1_AF027408
AT4G10040	0.002222592	3.0947607	0.030838741	1.5698816	CYTC-2	cytochrome c several plant cytochrome c (for instance cucurbit, PIR1:CCPU);supported by full-length cDNA: Ceres:31115.
AT3G14660	0.030613959	3.0796664	0.007912322	5.5311065	CYP72A13	putative cytochrome P450 similar to GB:Q05047 from [Catharanthus roseus]
AT1G72680	0.002704469	3.0657954	0.012842812	2.061285		putative cinnamyl-alcohol dehydrogenase similar to cinnamyl-alcohol dehydrogenase GB:AAC35846 [Medicago sativa]
AT5G50210	0.001568571	3.0527754	0.024017146	1.5830164	QS	putative protein similar to unknown protein (gb|AAF26953.1);supported by full-length cDNA: Ceres:15702.
AT5G54390	0.001972574	3.043103	0.012667717	2.162674	AHL	putative protein 3 (2'), 5'-bisphosphate nucleotidase protein-like; also similar to yeast halotolerance protein HAL2;supported by full-length cDNA: Ceres:253770.
AT3G53150	0.023241706	3.0386004	0.003701695	2.032815	UGT73D1	glucosyltransferase - like protein glucosyltransferase IS10a, salicylate-induced, Nicotiana tabacum, PIR:T03745
AT2G02220	0.003071624	3.0378993	0.006024542	2.0495827	ATPSKR1	putative protein kinase
AT4G27830	0.025053091	3.0335014	0.004425827	3.7182941		putative beta-glucosidase beta-glucosidase BGQ60 precursor - barley, PIR2:A57512; supported by cDNA: gi_15982821_gb_AY057518.1_
AT1G73730	0.008443	3.0311124	0.004258467	2.1580987	EIL3	ethylene-insensitive3-like3 (EIL3) identical to ethylene-insensitive3-like3 (EIL3) GB:AF004215 [Arabidopsis thaliana] (Cell 89 (7), 1133-1144 (1997))
AT1G16540	0.00910621	3.0306141	0.048087057	1.8408856	ABA3	hypothetical protein contains similarity to molybdopterin cofactor sulfurase GI:8978317 from [Bos taurus]; supported by cDNA: gi_13123672_gb_AF325457.1_AF325457
AT1G11400	0.000424419	3.0075889	0.024900489	1.8264543	PYM	hypothetical protein predicted by genemark.hmm;supported by full-length cDNA: Ceres:108306.
AT5G22060	0.000858013	2.988493	0.011457519	1.5538598	ATJ2	Expressed protein ; supported by cDNA: gi_535587_gb_L36113.1_ATHATJ
AT3G25230	0.001490481	2.979923	0.024791084	1.6137936	ROF1	rotamase FKBP (ROF1) identical to rotamase FKBP (ROF1) GB:U49453 [Arabidopsis thaliana] (Mol. Gen. Genet. 252 (5), 510-517 (1996)); supported by cDNA: gi_1354206_gb_U49453.1_ATU49453
AT1G31350	0.006643208	2.9679344	0.040544126	1.530707		hypothetical protein similar to hypothetical protein GB:AAF24593 GI:6692128 from [Arabidopsis thaliana]
AT2G24100	0.005645529	2.9614995	0.021346945	2.066123		unknown protein ; supported by cDNA: gi_14596216_gb_AY042896.1_
AT5G59030	0.000534384	2.9559426	0.04500876	1.7800863	COPT1	copper transport protein
AT1G09070	0.00597662	2.9473643	0.023065167	1.5180867	(AT)SRC2	unknown protein Similar to Glycine SRC2 (gb|AB000130). ESTs gb|H76869,gb|T21700,gb|ATTS5089 come from this gene; supported by cDNA: gi_15010557_gb_AY045580.1_
AT5G49930	0.004701317	2.9371066	0.021050703	1.4761382	EMB1441	putative protein strong similarity to unknown protein (gb|AAF56406.1)
AT2G25950	0.003180703	2.9272761	0.010260057	1.6821352		unknown protein ;supported by full-length cDNA: Ceres:39666.
AT2G33590	0.002125124	2.924367	0.031751476	1.7891067		putative cinnamoyl-CoA reductase highly similar to F4P9.37;supported by full-length cDNA: Ceres:11254.
AT1G20640	0.002072365	2.9126823	0.008111879	1.6939242		nodule inception protein, putative similar to nodule inception protein GI:6448579 from (Lotus japonicus)
AT4G33050	0.03092335	2.9099145	0.03154573	1.6519818	EDA39	putative protein
AT4G28480	0.001083083	2.9082122	0.021672826	1.4067031		heat-shock protein heat-shock protein hsp40-human, PIR2:JN0912; supported by cDNA: gi_14596114_gb_AY042845.1_
AT1G71330 /// A	0.001990669	2.9054816	0.003559839	5.5282493	ATNAP5	putative ABC transporter contains Pfam profile: PF00005 ABC transporter
AT3G57750	0.010478274	2.9014015	0.010014894	1.5404141		protein kinase - like protein wall-associated kinase 1, Arabidopsis thaliana, AJ009696;supported by full-length cDNA: Ceres:105330.
AT5G47230	0.00330901	2.874979	0.009807889	1.7954566	ERF5	ethylene responsive element binding factor 5 (ATERF5) (sp|O80341) ; supported by cDNA: gi_14326511_gb_AF385709.1_AF385709
AT1G33490	0.001562901	2.8747094	0.012163703	1.7267799		unknown protein ;supported by full-length cDNA: Ceres:92215.
AT1G31335	0.005701123	2.8734157	0.040544126	1.5584881		Expressed protein ; supported by full-length cDNA: Ceres: 109156.
AT3G12630	0.001626333	2.852564	0.007819225	1.694065		unknown protein ; supported by full-length cDNA: Ceres: 11684.
AT3G19010	0.003875213	2.8457048	0.005143304	1.8984054		hypothetical protein contains similarity to flavonol synthase (FLS) GB:Q41452 from [Solanum tuberosum], contains Pfam profile: PF00671 Iron/Ascorbate oxidoreductase family;supported by full-length cDNA: Ceres:41506.
AT1G29400	0.003870141	2.8344085	0.011686415	2.020828	AML5	RNA-binding protein MEI2, putative similar to GI:6650523 from [Arabidopsis thaliana]
AT1G10050	0.004220344	2.8341463	0.019014608	1.7753282		putative xylan endohydrolase similar to GB:AAD27896 to endoxylanases gi|1255238 (Thermoanaerobacterium thermosulfurigenes), gi|1813595 (Hordeum vulgare) and others
AT1G04990	0.002060344	2.8309326	0.033373956	1.5038373		zinc finger protein 2, putative similar to GB:AAD33770 from [Arabidopsis thaliana]; supported by cDNA: gi_15081720_gb_AY048253.1_
AT2G29060	0.011413972	2.8284044	0.022391045	2.0834138		putative SCARECROW gene regulator
AT2G47460	0.00248279	2.819402	0.003083379	1.7189106	MYB12	putative MYB family transcription factor ; supported by cDNA: gi_3941419_gb_AF062864.1_AF062864
AT5G56340	0.007216559	2.8094404	0.018134188	1.5612581		putative protein similar to unknown protein (pir||T05064)
AT1G10650	0.002727576	2.8037045	0.009211053	1.691775		S-ribonuclease binding protein SBP1, putative similar to S-ribonuclease binding protein SBP1 GI:6760451 from [Petunia hybrida];supported by full-length cDNA: Ceres:108776.
AT3G06420	0.004001373	2.7881358	0.0206897	1.6777713	ATG8H	hypothetical protein similar to symbiosis related proteins: GB:AAB53650 [Laccaria bicolor], GB:P87068 [Laccaria bicolor];supported by full-length cDNA: Ceres:142335.
AT3G44110	0.001713485	2.7765706	0.018233245	1.4714066	ATJ3	dnaJ protein homolog atj3 ;supported by full-length cDNA: Ceres:40976.
AT2G24180	0.008122208	2.7746882	0.014233842	1.6598915	CYP71B6	putative cytochrome P450 identical to GB:D78604; supported by cDNA: gi_15450907_gb_AY054534.1_
AT5G12880	0.011072388	2.7607687	0.014768215	1.8382453		putative protein hydroxyproline-rich glycoprotein, kidney bean, PIR:A29356
AT3G24100	0.001440628	2.7562423	0.040319856	1.8943764		unknown protein
AT4G26080	0.004634871	2.7558897	0.009211053	1.9928635	ABI1	protein phosphatase ABI1 ; supported by cDNA: gi_14334799_gb_AY035073.1_
AT3G14690	0.026138026	2.751237	0.006460856	7.119299	CYP72A15	putative cytochrome P450 similar to GB:Q05047 from [Catharanthus roseus]; supported by cDNA: gi_15292786_gb_AY050827.1_
AT4G01700	0.01295694	2.7451317	0.018669752	1.494204		putative chitinase similar to peanut type II chitinase, GenBank accession number X82329, E.C. 3.2.1.14
AT3G27150	0.03801087	2.7411456	0.003779925	2.5897033		unknown protein contains Pfam profile: PF01344 kelch motif
AT5G56600	0.003869486	2.7303548	0.041097052	1.766553	PFN3	profilin-like protein ; supported by full-length cDNA: Ceres: 102364.
AT5G59540	0.021871964	2.7270353	0.016187336	3.4350045		1-aminocyclopropane-1-carboxylate oxidase - like protein 1-aminocyclopropane-1-carboxylate oxidase kidney bean, PIR:T10818; supported by cDNA: gi_15983482_gb_AF424616.1_AF42461
AT1G21760	0.000645502	2.7156243	0.013415542	1.9326829		unknown protein Contains PF|00646 F-box domain. ESTs gb|Z37267, gb|R90412, gb|Z37268 and gb|T88189 come from this gene;supported by full-length cDNA: Ceres:11543.
AT3G19910	0.001215955	2.7118473	0.010260057	1.5277917		hypothetical protein predicted by genemark;supported by full-length cDNA: Ceres:108568.
AT2G41705	0.008768661	2.6956408	0.025294408	1.6897581		Expressed protein ; supported by cDNA: gi_13605678_gb_AF361820.1_AF361820
AT3G23605	0.008138582	2.6907125	0.005567558	1.9564288		Expressed protein ; supported by full-length cDNA: Ceres: 7103.
AT1G60940	0.008967559	2.679956	0.022462012	1.9776024	SNRK2-10	putative serine/threonine-protein kinase similar to SERINE/THREONINE-PROTEIN KINASE ASK1 GB:P43291 from [Arabidopsis thaliana];supported by full-length cDNA: Ceres:95559.
AT5G40580	0.0007381	2.6663756	0.015811684	1.5677394	PBB2	20S proteasome beta subunit PBB2 ;supported by full-length cDNA: Ceres:38185.
AT1G05890	0.002772258	2.6545272	0.02077622	1.7992041		unknown protein
AT3G07720	0.009323138	2.6523762	0.009211053	3.829392		unknown protein similar to hypothetical protein GB:S33464 [Arabidopsis thaliana]; supported by cDNA: gi_14517447_gb_AY039559.1_
AT5G20520	0.001670648	2.6467319	0.025587441	1.4892739	WAV2	putative protein BEM46 bem1/bud5 suppressor, Schizosaccharomyces pombe, bem1/bud5 suppressor, EMBL:SP29892;supported by full-length cDNA: Ceres:36716.
AT5G62910	0.007888611	2.6346576	0.019898033	2.5258887		putative protein predicted proteins, Arabidopsis thaliana
AT1G17145	0.001218186	2.630678	0.009631788	1.8139901		Expressed protein ; supported by full-length cDNA: Ceres: 6612.
AT1G77670	0.000534029	2.6221235	0.003779925	1.9982278		putative aminotransferase similar to kynurenine aminotransferase GB:S48737 [Rattus norvegicus], similar to aspartate aminotransferase GB:P53001 [Bacillus subtilis];supported by full-length cDNA: Ceres:35179.
AT1G02750	0.006201174	2.6142306	0.007614888	2.3943422		hypothetical protein predicted by genemark.hmm
AT3G06760	0.006292802	2.6105149	0.005060274	1.613232		unknown protein
AT1G04960	0.003468541	2.609707	0.031961158	1.6973187		unknown protein ;supported by full-length cDNA: Ceres:39874.
AT2G43820	0.035561923	2.5910678	0.002792461	3.099281	GT	putative glucosyltransferase ;supported by full-length cDNA: Ceres:34407.
AT5G04340	0.010034787	2.587418	0.005592544	2.2092888	C2H2	putative c2h2 zinc finger transcription factor
AT1G52250	0.038766704	2.5722525	0.039171	2.0801628		dynein light chain, putative similar to dynein light chain 1, cytoplasmic SP:Q15701 [Homo sapiens]
AT1G02410	0.001096664	2.549327	0.024485148	1.4718441		hypothetical protein contains similarity to cytochrome c oxidase assembly protein cox11 GI:1244782 from [Saccharomyces cerevisiae]
AT3G47550	0.002535562	2.5458772	0.010470351	1.6118188		putative protein PIT1 - Arabidopsis thaliana, GB: AF130849
AT2G38290	0.024675947	2.5446486	0.007676405	2.5047357	ATAMT2	putative ammonium transporter
AT1G45976	0.001259501	2.52496	0.014067003	1.5528734	SBP1	S-ribonuclease binding protein SBP1, putative similar to S-ribonuclease binding protein SBP1 GI:6760451 from [Petunia hybrida]; supported by cDNA: gi_11692935_gb_AF324720.1_AF324720
AT4G31290	0.00093031	2.5061357	0.01109956	1.9922144		predicted protein cation transport protein ChaC, Escherichia coli, PIR2:G64868;supported by full-length cDNA: Ceres:39740.
AT1G21110 /// A	0.017271753	2.499821	0.033668105	1.5383109		O-methyltransferase, putative similar to GB:AAF28353 from [Fragaria x ananassa]
AT1G68795	0.031693053	2.493601	0.022331033	2.6687415	CLE12	CLE12 CLAVATA3/ESR-Related 12; supported by full-length cDNA: Ceres: 31888.
AT1G25400	0.014825144	2.4928083	0.029557064	2.481706		unknown protein similar to unknown protein GI:6714347 from [Arabidopsis thaliana];supported by full-length cDNA: Ceres:103226.
AT3G47540	0.003315416	2.4912367	0.008271794	2.3073933		endochitinase-like protein BASIC ENDOCHITINASE CHB4 PRECURSOR - Brassica napus, SWISSPROT:CHI4_BRANA
AT3G21250	0.029351955	2.4848895	0.047130916	1.5276469	ATMRP6	unknown protein similar to MRP-like ABC transporter GB:AAC49791 from [Arabidopsis thaliana]
AT2G28000	0.001322674	2.4814663	0.001992044	1.4499989	CPN60A	putative rubisco subunit binding-protein alpha subunit ;supported by full-length cDNA: Ceres:25773.
AT4G25900	0.0029923	2.479444	0.040691927	1.533274		possible apospory-associated like protein Pennisetum ciliare possible apospory-associated mRNA clone pSUB C, PID:g549984;supported by full-length cDNA: Ceres:37817.
AT3G48880	0.000697835	2.4738455	0.017656097	1.5444282		putative protein N7 protein - Medicago truncatula, EMBL:CAA76808;supported by full-length cDNA: Ceres:99337.
AT1G62390	0.000975366	2.4707446	0.012197621	1.5258775		unknown protein similar to putative cytoskeletal protein GI:1732517 from [Arabidopsis thaliana]; supported by cDNA: gi_1732516_gb_U62745.1_ATU62745
AT4G25230	0.004657667	2.4688241	0.014998885	1.9741031	RIN2	putative protein
AT5G06839	0.011347864	2.467032	0.045228474	1.5315229		bZIP transcription factor-like protein
AT2G31955	0.006415154	2.4579377	0.027885096	1.6300201	CNX2	molybdopterin synthase (CNX2) ;supported by full-length cDNA: Ceres:33192.
AT1G15230	0.001083083	2.4529166	0.002145344	1.6065555		unknown protein ESTs gb|R30529, gb|Z48463, gb|Z48467, gb|AA597369 and gb|AA394772 come from this gene; supported by cDNA: gi_16209679_gb_AY057603.1_
AT5G63330	0.000511264	2.4499087	0.037614495	1.4026419		putative protein contains similarity to kinase
AT2G07180	0.0148241	2.4494226	0.046081476	1.4239048		putative protein kinase contains a protein kinase domain profile (PDOC00100)
AT5G45710	0.003841657	2.4451132	0.037936978	1.4716272	AT-HSFA4C	heat shock transcription factor
AT2G24500	0.007831488	2.4437373	0.002754528	2.453981	FZF	putative C2H2-type zinc finger protein likely a nucleic acid binding protein; supported by cDNA: gi_4585204_gb_AF095588.1_AF095588
AT5G04930	0.015964143	2.4402792	0.018211536	1.5443736	ALA1	Expressed protein ; supported by cDNA: gi_9909197_gb_AF175769.1_AF175769
AT3G56200	0.03744888	2.4350393	0.008060786	2.621062		putative protein neuronal glutamine transporter - Rattus norvegicus, EMBL:AF075704
AT2G34660	0.02159418	2.4326234	0.000612937	2.8254018	ATMRP2	ABC transporter (AtMRP2) identical to GB:AF014960; transports glutathione conjugates; supported by cDNA: gi_2909780_gb_AF020288.1_AF020288
AT1G59590	0.013948233	2.4306955	0.009193028	2.3312154	ZCF37	hypothetical protein predicted by genemark.hmm;supported by full-length cDNA: Ceres:99553.
AT1G75370	0.004558383	2.427	0.007178671	1.4092503		sec14 cytosolic factor, putative similar to SP:P24859 from [Kluyveromyces lactis]
AT5G60800	0.045243375	2.4245064	0.017073514	2.0786757		putative protein farnesylated protein GMFP5, Glycine max, EMBL:U64916
AT4G17900	0.001964642	2.4203465	0.023353059	1.6344873		putative protein
AT1G69840	0.00601392	2.4108381	0.010792479	1.4836447		unknown protein similar to hypothetical protein GB:CAA10289 [Cicer arietinum]
AT5G67580	0.015550653	2.4105508	0.043658298	1.9253439	ATTRB2	putative protein contains similarity to MYB-like DNA-binding protein; supported by cDNA: gi_14532517_gb_AY039883.1_
AT1G68300	0.002969	2.4093451	0.005249115	1.5142174		unknown protein ;supported by full-length cDNA: Ceres:27109.
AT2G44670	0.005773597	2.406736	0.003118423	2.9586985		unknown protein ;supported by full-length cDNA: Ceres:40641.
AT1G77450	0.03747708	2.38904	0.015415192	2.5373318	ANAC032	GRAB1-like protein similar to GRAB1 protein GB:CAA09371, a novel member of the NAC domain family;supported by full-length cDNA: Ceres:36478.
AT3G55600	0.001435934	2.3813279	0.006060074	1.8295563		putative protein predicted proteins, Arabidopsis thaliana
AT4G32160	0.006439745	2.3801165	0.037818104	1.4265548		putative protein predicted gene T10G3.5, Caenorhabditis elegans, PATCHX:E308128; supported by cDNA: gi_16604600_gb_AY059745.1_
AT3G60300	0.00219493	2.3774245	0.013221123	1.5274693		putative protein RING finger protein AO7, Mus musculus, EMBL:AF171060
AT3G07565	0.002772231	2.3744814	0.01166079	1.5710111		unknown protein similar to unknown protein, 3' partial GB:AAF02161 [Arabidopsis thaliana]
AT2G26300	0.001215955	2.3724775	0.003559839	1.8088921	GPA1	G protein alpha subunit 1 (GPA1) identical to GB:M32887; supported by cDNA: gi_14326501_gb_AF385704.1_AF385704
AT5G02290	0.001005777	2.367523	0.015941067	1.458237	NAK	serine/threonine-specific protein kinase NAK ;supported by full-length cDNA: Ceres:27477.
AT1G79710	0.022504218	2.3591294	0.006285763	1.9585185		hypothetical protein similar to hypothetical protein GB:AAC12874 (Synechococcus PCC7942)
AT2G21620	0.011297009	2.3539782	0.009974133	1.632167	RD2	unknown protein ;supported by full-length cDNA: Ceres:31655.
AT1G01720	0.01711272	2.3474545	0.008730779	2.2796404	ATAF1	NAC domain protein, putative similar to NAC domain protein NAM GB:AAD17313 GI:4325282 from [Arabidopsis thaliana];supported by full-length cDNA: Ceres:20909.
AT3G48890	0.00111891	2.3347208	0.021693233	1.4070354	ATMP2	putative progesterone-binding protein homolog Atmp2 ; supported by cDNA: gi_4960153_gb_AF153283.1_AF15328
AT1G51420	0.04330352	2.3266501	0.011804807	2.2267382		unknown protein
AT3G18290	0.009171248	2.3126633	0.003355231	2.9686215	EMB2454	zinc finger protein, putative contains Pfam profiles: PF00097 Zinc finger, C3HC4 type (RING finger) (2 copies)
AT4G33070	0.04388918	2.310839	0.006556409	4.202299		pyruvate decarboxylase-1 (Pdc1)
AT2G13960 /// A	0.032142073	2.3037908	0.008943213	1.4350462		putative MYB family transcription factor
AT1G05410	0.00219493	2.303685	0.0214611	1.4498984		hypothetical protein predicted by genemark.hmm;supported by full-length cDNA: Ceres:38810.
AT5G42350 /// A	0.00053161	2.2967145	0.017809508	1.5697482		putative protein similar to unknown protein (gb|AAF07802.1);supported by full-length cDNA: Ceres:271310.
AT3G13330	0.011286099	2.290635	0.002648022	2.1718156		hypothetical protein predicted by genscan+
AT2G45170	0.003071624	2.2890892	0.010792479	1.561489	AtATG8e	putative microtubule-associated protein ; supported by cDNA: gi_15724331_gb_AF412106.1_AF412106
AT5G41180	0.003274166	2.2811024	0.013242396	1.5964147		receptor kinase-like protein
AT5G24270	0.026240636	2.2805083	0.005915129	2.3179274	SOS3	calcium sensor homolog (gb|AAC26110.1) ; supported by cDNA: gi_11065946_gb_AF192886.1_AF192886
AT1G04810	0.003576818	2.279132	0.010417465	1.7697662		26S proteasome regulatory subunit, putative similar to GB:AAC04490 from [Arabidopsis thaliana]
AT5G11260	0.000534384	2.2790005	0.00523089	2.011679	HY5	HY5(bZIP transcription factor family) identical to HY5 protein GI:2251084 from [Arabidopsis thaliana]
AT3G26690	0.006191024	2.2755702	0.015657164	1.5132066	ATNUDT13	MutT-like protein contains Pfam profile: PF00293 Bacterial mutT protein
AT4G24290	0.001736286	2.273847	0.0491593	1.4449754		putative protein ; supported by cDNA: gi_15810434_gb_AY056256.1_
AT5G07860	0.042238872	2.257782	0.003952722	3.5968716		N-hydroxycinnamoyl benzoyltransferase - like protein N-hydroxycinnamoyl benzoyltransferase, Ipomoea batatas, EMBL:AB035183
AT3G53930	0.005338477	2.252871	0.015042232	1.7709655		putative protein serine/threonine-specific protein kinase ULK1 - Mus musculus,PIR:JW0051
AT3G12800	0.004211289	2.2410285	0.011133249	1.4431617		unknown protein similar to unknown protein GB:AAD37022 from [Arabidopsis thaliana]
AT2G27490	0.002275947	2.226759	0.01109956	1.549969	ATCOAE	unknown protein
AT1G07030	0.014112391	2.2265818	0.015866674	1.5415996		mitochondrial carrier protein, putative similar to mitochondrial carrier protein GI:3378495 from [Ribes nigrum]
AT5G64760	0.001694575	2.226308	0.004274498	1.8381593		proteasome regulatory subunit-like ; supported by cDNA: gi_17063180_gb_AY062113.1_
AT2G47890	0.005819835	2.2233658	0.004351474	3.317156		putative zinc-finger protein (B-box zinc finger domain)
AT3G16330	0.000242192	2.2224863	0.009211053	1.8879161		unknown protein
AT5G19400	0.003880734	2.216706	0.012977538	1.7033038		putative protein predicted proteins, Homo sapiens
AT5G02840	0.002813752	2.2129462	0.020342216	1.4154202	LCL1	putative protein various Myb related proteins, Arabidopsis thaliana; supported by cDNA: gi_14596212_gb_AY042894.1_
AT3G22890	0.009625791	2.2087622	0.002808379	2.4438875	APS1	ATP sulfurylase, putative similar to ATP sulfurylase GB:AAF19185 [Arabidopsis thaliana];supported by full-length cDNA: Ceres:20342.
AT3G06780	0.004896319	2.204414	0.01475492	2.031691		hypothetical protein predicted by genemark.hmm; supported by cDNA: gi_15450370_gb_AY052286.1_
AT5G13360 /// A	0.001761854	2.201669	0.021783395	1.6343828		auxin reponsive - like protein Nt-gh3 deduced protein, Nicotiana tabacum, EMBL:AF123503
AT4G01070	0.01968488	2.1943295	0.002824896	2.1788137	GT72B1	putative flavonol glucosyltransferase similar to Manihot esculenta flavonol 3-O-glucosyltransferase 5, GenBank accession number Q40287;supported by full-length cDNA: Ceres:1204.
AT3G54130	0.006097663	2.1778438	0.012667717	1.4198004		Machado-Joseph disease MJD1a -like protein Machado-Joseph disease MJD1a protein, Homo sapiens, PIR:S50830
AT3G05390	0.00257732	2.174342	0.023272073	1.6184288		hypothetical protein predicted by genscan+
AT5G11950	0.003679115	2.1732218	0.008618869	1.4221596		lysine decarboxylase - like protein lysine decarboxylase, Eikenella corrodens, EMBL:U89166
AT1G70530	0.003277496	2.1584659	0.0387124	1.6047335		putative protein kinase similar to C-terminal region of s-receptor kinase precursor GB:JQ1677 [Brassica napus]; Pfam HMM hit: Eukaryotic protein kinase domain
AT1G64200	0.00900512	2.1552682	0.013086765	1.7646818	VHA-E3	H+-transporting ATPase protein, putative similar to H+-transporting ATPase chain E GB:CAB43050 GI:4850294 from [Arabidopsis thaliana]
AT3G07870	0.007408435	2.1530743	0.006688091	2.0257452		unknown protein ; supported by cDNA: gi_15983478_gb_AF424614.1_AF424614
AT1G68410	0.012295961	2.1463923	0.02413027	1.7232889		putative protein phosphatase similar to protein phosphatase-2C GB:AAC36697 from [Mesembryanthemum crystallinum]; supported by cDNA: gi_15292894_gb_AY050881.1_

AT1G75860	0.008353614	2.13914	0.002792891	2.6483433		unknown protein
AT2G15790	0.009574787	2.1357067	0.016196333	1.511384	SQN	hypothetical protein ; supported by cDNA: gi_13442982_gb_AY026065.1_
AT2G34910	0.013138001	2.1338983	0.01978298	1.5202911		hypothetical protein predicted by genefinder
AT1G28280	0.004085816	2.133757	0.021695841	1.4256146		hypothetical protein predicted by genemark.hmm;supported by full-length cDNA: Ceres:20991.
AT3G46600	0.003204085	2.1331685	0.03698339	2.1949909		scarecrow-like protein scarecrow-like 11 - Arabidopsis thaliana, EMBL:AF036307;supported by full-length cDNA: Ceres:35201.
AT3G50930	0.01627059	2.129695	0.002145344	5.5516553		BCS1 protein-like protein Homo sapiens h-bcs1 (BCS1) mRNA, nuclear gene encoding mitochondrial protein which is involved in the expression of functional mitochondrial ubiquinol-cytochrome c reductase complex probably via the control of expression of Riesk
AT4G11220	0.001084539	2.1218314	0.015204758	1.4000825	BTI2	putative protein 24 kDa seed maturation protein - Glycine max,PID:g4102690;supported by full-length cDNA: Ceres:23536.
AT4G22590 /// AT	0.002359667	2.12152	0.002145344	3.5014973		trehalose-6-phosphate phosphatase - like protein trehalose-6-phosphate phosphatase (AtTPPA), PID:g2944178
AT2G41630	0.000734108	2.116439	0.008999672	1.4129922	TFIIB	transcription factor IIB (TFIIB) identical to GB:P48512; contains a transcription factor TFIIB repeat signature (PDOC00624);supported by full-length cDNA: Ceres:2657.
AT2G30250	0.011810336	2.114799	0.01502979	2.282873	WRKY25	putative WRKY-type DNA binding protein ; supported by cDNA: gi_15027912_gb_AY045813.1_
AT5G55200	0.000902111	2.1054637	0.020442348	1.5620148		chaperone GrpE-like protein ;supported by full-length cDNA: Ceres:20790.
AT1G62420 /// AT	0.004949881	2.0898647	0.032768417	1.4047712		unknown protein
AT5G24800	0.001083083	2.0829258	0.001810002	4.2311873	BZO2H2	transcription factor-like protein light-induced protein CPRF-2 - Petroselinum crispum, PIR:S16321
AT3G11270	0.006078816	2.0829206	0.012096576	1.4934204	MEE34	26S proteasome regulatory subunit S12, putative nearly identical to 26S proteasome regulatory subunit S12 (MOV34) SP:O24412 [Arabidopsis thaliana (Mouse-ear cress)]
AT5G05100	0.002714328	2.076963	0.027508326	1.4921435		putative protein strong similarity to unknown protein (gb|AAF19572.1); supported by cDNA: gi_16649096_gb_AY059918.1_
AT1G68820	0.02766306	2.0751333	0.004097793	1.841439		hypothetical protein C-term similar to C-term of apoptosis inhibitor GB:AAC70265 [Lymantria dispar nucleopolyhedrovirus]; supported by cDNA: gi_13878072_gb_AF370299.1_AF370299
AT4G02200	0.002336344	2.0698037	0.019982234	1.6080672		drought-induced-19-like 1 similar to drought-induced-19, GenBank accession number X78584 similar to F2P16.10, GenBank accession number 2191179 identical to T10M13.20
AT4G34150	0.017287666	2.0555015	0.040544126	1.4385464		putative protein hydroxyproline-rich glycoprotein precursor, Nicotiana tabacum, PIR2:S06733; supported by cDNA: gi_15724315_gb_AF412098.1_AF412098
AT3G06810	0.010334071	2.0545487	0.012952536	1.8436083	IBR3	acetyl-coA dehydrogenase, putative similar to acetyl-coenzyme A dehydrogenase GB:NP_031408 from [Mus musculus]
AT2G39110	0.017271753	2.0538616	0.007138968	2.5718005		putative protein kinase contains a protein kinase domain profile (PDOC00100)
AT4G38540	0.024763353	2.0455086	0.006620886	1.7371444		monooxygenase 2 (MO2)
AT1G01120	0.021212697	2.044808	0.007475819	2.5622244	KCS1	fatty acid elongase 3-ketoacyl-CoA synthase 1 identical to GB:AAC99312 GI:4091810 from [Arabidopsis thaliana]
AT2G02180	0.00489168	2.0406225	0.011761585	1.4400284	TOM3	unknown protein ;supported by full-length cDNA: Ceres:12568.
AT1G63830	0.001167618	2.0309398	0.027929148	1.5658517		unknown protein similar to putative protein GB:CAA20468 [Arabidopsis thaliana]
AT5G66810	0.003348466	2.0272918	0.041115638	1.4682397		putative protein similar to unknown protein (gb|AAB71479.1|)
AT4G32070	0.020400725	2.017099	0.00655279	1.7380496		putative protein predicted protein, Arabidopsis thaliana, PATCHX:G1732517
AT2G33310	0.00380895	2.003946	0.01237766	1.5519952	IAA13	auxin regulated protein (IAA13) GB:S58499;supported by full-length cDNA: Ceres:13741.
AT3G30775	0.028741183	1.7564306	0.005994618	4.61892	ERD5	proline oxidase, mitochondrial precursor (osmotic stress-induced proline dehydrogenase) identical to GB:P92983 from [Arabidopsis thaliana] (Plant Cell 8 (8), 1323-1335 (1996))
AT2G19800	0.031733155	1.8409374	0.026346834	3.9925911	MIOX2	unknown protein predicted by genscan;supported by full-length cDNA: Ceres:254633.
AT5G17760	0.004908105	1.9617585	0.009211053	2.7185016		BCS1 - like protein h-bcs1, Homo sapiens, EMBL:AF026849; supported by cDNA: gi_15810570_gb_AY056324.1_
AT4G10955 /// AT	0.003143723	1.7993052	0.008800991	2.557326	UGE5	predicted protein
AT3G07700	0.005920455	1.930073	0.006363932	2.4100285		unknown protein similar to hypothetical proteins GB:P73627, GB:Q55884, GB:P73121 [Synechocystis sp.]; supported by full-length cDNA: Ceres: 95043.
AT2G39570	0.001629458	1.9258249	0.005715475	2.3114882		unknown protein ; supported by cDNA: gi_15010737_gb_AY045670.1_
AT2G38210 /// AT	0.03986957	1.5789692	0.027476324	2.1817803	ATPDX1.1	similar to SOR1 from the fungus Cercospora nicotianae
AT1G50460	0.01771369	1.8547616	0.025302233	2.1382282		hexokinase, putative similar to chloroplast outer envelope hexokinase 1 GB: AAF18584 GI:6594672 from [Spinacia oleracea]
AT3G25400	0.038783308	1.6281828	0.024485148	2.1319084		unknown protein
AT4G34890 /// AT	0.023121065	1.6854993	0.009533367	2.1077242		xanthine dehydrogenase - like protein xanthine dehydrogenase, Gallus gallus, PIR:XOCHDH
AT3G06500	0.04319324	1.502794	0.011828709	2.0437212		neutral invertase, putative similar to neutral invertase GB:CAA76145 from [Daucus carota] (Physiol. Plantarum (1999) 107, 159-165)

Supplementary Table S1 As-responsive genes in *Arabidopsis* Col-0 and Ws-2 ecotypes (Col200-Ws100 Commonly Downp)

AGI	Col-200 q-value	Col-200 FC	Ws-100 q-value	Ws-100 FC	Gene Symbol	Target Description
AT4G22610	0.001521358	0.006923948	0.011457519	0.353392305		putative protein AIR1, Arabidopsis thaliana, PID:g3695017;supported by full-length cDNA: Ceres:5348.
AT5G19890	0.00028439	0.006965864	0.011761585	0.135227364		peroxidase ATP N ;supported by full-length cDNA: Ceres:40493.
AT5G04950	0.001005475	0.007601082	0.03355945	0.141233423		nicotianamine synthase (dbj|BAA74589.1)
AT3G58990	0.000471695	0.008013408	0.012277073	0.209357427		3-isopropylmalate dehydratase-like protein (small subunit) 3-isopropylmalate dehydratase, small subunit - Thermotoga maritima, PIR:A72363
AT4G13770 /// AT5G65110	0.000424419	0.008330586	0.012740123	0.202051469	CYP83A1	cytochrome P450 monooxygenase (CYP83A1) ; supported by cDNA: gi_3164127_dbj_D78599.1_D78599
AT3G01260	0.000471695	0.008518133	0.002792891	0.123657482		putative aldose 1-epimerase shows similarity to aldose epimerases
AT4G11310 /// AT4G11320	0.002227739	0.009419107	0.026255853	0.257707437		cysteine proteinase contains similarity to cysteine proteinase RD21A (thiol protease) GI:435619, SP:P43297 from [Arabidopsis thaliana]
AT3G45700	0.000652063	0.009885874	0.017985906	0.128281492		putative transporter protein peptide transport protein - Hordeum vulgare, PIR:T04378
AT3G13760	4.87195E-05	0.01009771	0.006620886	0.372679465		hypothetical protein predicted by genemark.hmm
AT4G31910	0.000182259	0.011094901	0.01166408	0.334319297		putative protein anthranilate N-hydroxycinnamoyl/benzoyltransferase,Dianthus caryophyllus, Z84384; supported by cDNA: gi_15450358_gb_AY052280.1_
AT5G01740	0.000190587	0.013352242	0.001810002	0.031898978		putative protein wound-inducible protein wun1 protein - Solanum tuberosum, PIR:JQ0398;supported by full-length cDNA: Ceres:248967.
AT5G61420	0.000269321	0.014395271	0.022239825	0.352486896	MYB28	putative transcription factor MYB28 ; supported by cDNA: gi_5823328_gb_AF175998.1_AF175998
AT3G27170	0.000389376	0.017495936	0.003083379	0.292839695	CLC-B	CLC-b chloride channel protein identical to CLC-b chloride channel protein GB:CAA96058 from [Arabidopsis thaliana] (J. Biol. Chem. 271 (52), 33632-33638 (1996))
AT5G52790	0.001611023	0.017656077	0.023353059	0.24951828		putative protein similar to unknown protein (pir||T04987)
AT4G00700	0.000608712	0.01801451	0.020722393	0.653293786		putative phosphoribosylanthranilate transferase
AT2G17820	0.000897589	0.019035078	0.008135078	0.242742644	ATHK1	putative histidine kinase histidine kinase domain from residues 490 to 1190; supported by cDNA: gi_3399696_dbj_AB010914.1_AB010914
AT5G19970	0.000879474	0.019174395	0.01033256	0.068243925		putative protein
AT4G08300	0.000446479	0.021648564	0.002705098	0.046157252		nodulin-like protein nodulin gene MtN21 - Medicago truncatula, PID:e1172471
AT3G46270	0.001053062	0.022231513	0.01851731	0.490891267		putative protein serine/threonine-specific receptor protein kinase (EC 2.7.1.-), Arabidopsisthaliana, PIR:S71277
AT3G02850	0.004506142	0.023386086	0.009193028	0.03121222	SKOR	stelar K+ outward rectifying channel (SKOR) identical to stelar K+ outward rectifying channel (SKOR) GB:AJ223358 [Arabidopsis thaliana]
AT5G15830	0.000679271	0.024812626	0.008838028	0.239922304	ATBZIP3	bZIP DNA-binding protein-like putative bZIP DNA-binding protein - Capsicum chinense, EMBL:AF127797
AT5G10580	0.001016433	0.026425958	0.009235052	0.34129192		putative protein predicted protein, Arabidopsis thaliana; supported by cDNA: gi_14326488_gb_AF385697.1_AF385697
AT2G40900	0.000407333	0.027224517	0.008216102	0.444457719		putative integral membrane protein nodulin
AT5G59780	0.001100269	0.027666201	0.00629379	0.256858996	MYB59	MYB27 protein - like MYB27 protein, Arabidopsis thaliana, PIR:T46166; supported by cDNA: gi_3941479_gb_AF062894.1_AF062894
AT5G25160	0.000362017	0.029742201	0.019157518	0.434729439	ZFP3	ZFP3 zinc finger protein ; supported by cDNA: gi_790676_gb_L39646.1_ATHZFPC
AT2G28780	0.005759002	0.030064154	0.017184246	0.157926229		hypothetical protein predicted by genscan and genefinder
AT4G12030	0.000534029	0.030898064	0.017696602	0.30250973		putative transport protein Na(+) dependent transporter (Sbf family) - Aquifex aeolicus, PIR2:E70482; supported by cDNA: gi_15215838_gb_AY050449.1_
AT1G13420	0.00127196	0.031239353	0.00437724	0.244469154		steroid sulfotransferase, putative similar to steroid sulfotransferase 1 GI:3420004 from (Brassica napus);supported by full-length cDNA: Ceres:150484.
AT3G45680	0.001440628	0.034884121	0.044967987	0.400896211		putative transporter protein peptide transport protein - Hordeum vulgare, PIR:T04378
AT2G37460	0.001626333	0.035046088	0.018669752	0.479706634		nodulin-like protein ;supported by full-length cDNA: Ceres:16393.
AT5G47450	0.001648161	0.035147275	0.014103841	0.316323941	AtTIP2;3	membrane channel protein-like; aquaporin (tonoplast intrinsic protein)-like
AT1G74090	0.000827021	0.03539003	0.002808379	0.194260515		putative flavonol sulfotransferase similar to FLAVONOL 4'-SULFOTRANSFERASE GB:P52837 from [Flaveria chloraefolia];supported by full-length cDNA: Ceres:41006.
AT1G32450	0.000783487	0.036502876	0.029213972	0.414414943		peptide transporter PTR2-B, putative similar to SP:P46032 from [Arabidopsis thaliana]
AT2G33830	0.007405147	0.038177491	0.023611085	0.249389414		putative auxin-regulated protein ;supported by full-length cDNA: Ceres:1711.
AT3G45160	0.000628484	0.038979828	0.019157518	0.418469293		hypothetical protein
AT5G61590	0.000534384	0.039159645	0.00810806	0.137604466		ethylene responsive element binding factor - like ethylene responsive element binding factor 5, Arabidopsis thaliana, SWISSPROT:ERF5_ARATH;supported by full-length cDNA: Ceres:19893.
AT2G43100	0.000893239	0.040601381	0.018245112	0.395691035		3-isopropylmalate dehydratase, small subunit ; supported by cDNA: gi_16974632_gb_AY060594.1_
AT3G62040	0.000836392	0.040928697	0.012103445	0.369973786		hypothetical protein contains non-consensus GA donor splice site at exon 2
AT1G62280	0.007152865	0.041869537	0.037883233	0.119527251	SLAH1	hypothetical protein predicted by genemark.hmm
AT4G36220	0.002275986	0.042299916	0.008602032	0.280190789	FAH1	ferulate-5-hydroxylase (FAH1) ; supported by cDNA: gi_1488254_gb_U38416.1_ATU38416
AT3G26330	0.001178052	0.042366252	0.02139672	0.571665175	CYP71B37	cytochrome P450, putative contains Pfam profile: PF00067 cytochrome P450
AT3G63210	0.00093031	0.042508829	0.003341037	0.322468956	MARD1	putative protein putative protein At2g25690 - Arabidopsis thaliana, EMBL:AC006053;supported by full-length cDNA: Ceres:40080.
AT4G31730	0.000628484	0.042606248	0.014896033	0.427022988	GDU1	hypothetical protein
AT4G08290	0.003171321	0.043112814	0.008060786	0.155472427		nodulin-like protein nodulin gene MtN21 - Medicago truncatula, PID:e1172471;supported by full-length cDNA: Ceres:40485.
AT1G68740	0.000287634	0.043605566	0.012842812	0.190735045		putative receptor protein similar to viral receptors GB:AAF13258 (Cricetulus griseus), (SYG1) GB:AAD17206 (Mus musculus), GB:AAF03485 (Cricetulus griseus); supported by cDNA: gi_15982932_gb_AY057574.1_
AT4G36670	0.005802603	0.044918476	0.01033256	0.069632069		sugar transporter like protein
AT2G46690	0.001557352	0.045034083	0.000612937	0.325053791		putative auxin-regulated protein
AT1G16400 /// AT1G16410	0.000492482	0.045290829	0.001810002	0.149474943	CYP79F2	putative cytochrome P450 similar to gb|AF069494 cytochrome P450 from Sinapis alba and is a member of the PF|00067 Cytochrome P450 family. EST gb|F14190 comes from this gene; supported by cDNA: gi_15208670_gb_AY035021.2_
AT1G67110	0.001541871	0.045684805	0.004852493	0.089132518	CYP735A2	cytochrome P450, putative similar to cytochrome P450 (CYP72B) GI:404687 from [Catharanthus roseus]
AT3G03190	0.001100269	0.046070317	0.010584146	0.27907748	ATGSTF11	glutathione S-transferase identical to glutathione S-transferase GB:AAB09584 from [Arabidopsis thaliana]; supported by cDNA: gi_1575751_gb_U70672.1_ATU70672
AT4G39675	0.004071192	0.046108857	0.018036846	0.160485926		Expressed protein ; supported by full-length cDNA: Ceres: 14423.
AT1G49500	0.001380261	0.047481834	0.004813203	0.102901546		unknown protein ;supported by full-length cDNA: Ceres:33047.
AT3G50740	0.000787158	0.048369021	0.018233245	0.25174781	UGT72E1	UTP-glucose glucosyltransferase - like protein UTP-glucose glucosyltransferase, Manihot esculenta, PIR:S41951; supported by cDNA: gi_15146271_gb_AY049277.1_
AT1G20160	0.001853667	0.048422412	0.009211053	0.245738845	ATSBT5.2	subtilisin-like serine protease similar to subtilisin-type protease precursor GI:14150446 from [Glycine max];supported by full-length cDNA: Ceres:3907.
AT2G38380 /// AT2G38390	0.00093031	0.049648365	0.037883233	0.43982062		peroxidase
AT2G21560	0.001632807	0.050739382	0.010009809	0.434856175		unknown protein
AT3G20015	0.001302025	0.051996267	0.003341037	0.353132657		hypothetical protein, 5' partial
AT1G11210	0.002480303	0.052106578	0.029329354	0.267395172		hypothetical protein similar to hypothetical protein GB:AAD50003 GI:5734738 from [Arabidopsis thaliana];supported by full-length cDNA: Ceres:37592.
AT5G60890	0.001401019	0.052967614	0.011525319	0.190818016	ATR1	Myb transcription factor homolog (ATR1)
AT1G28130	0.001440628	0.053234876	0.03019216	0.527587024	GH3.17	auxin-regulated GH3 protein, putative similar to auxin-regulated GH3 protein GI:18590 from (Glycine max); supported by cDNA: gi_15450364_gb_AY052283.1_
AT2G23030	0.003968458	0.0544121	0.013914318	0.163139216	SNRK2-9	putative protein kinase contains a protein kinase domain profile (PDOC00100)
AT2G31790	0.000470407	0.054578345	0.0068489	0.254449508		putative glucosyltransferase ; supported by cDNA: gi_15810476_gb_AY056277.1_
AT4G33550	0.002811891	0.054688063	0.04883703	0.699738452		putative protein predicted protein, Arabidopsis thaliana
AT2G16750	0.001322674	0.055952013	0.025487116	0.553353853		putative protein kinase contains a protein kinase domain profile (PDOC00100)
AT1G22500	0.001773371	0.05598502	0.006778613	0.139875732		RING-H2 zinc finger protein ATL5, putative similar to RING-H2 zinc finger protein ATL5 GI:4928401 from [Arabidopsis thaliana]
AT1G31770	0.001599981	0.057390301	0.003660176	0.316032453		ABC transporter, putative contains Pfam profile: PF00005: ABC transporter;supported by full-length cDNA: Ceres:95290.
AT3G61380	0.003758996	0.058296818	0.025274463	0.232790876		putative protein hypothetical protein At2g45900 - Arabidopsis thaliana, EMBL:AC004665
AT3G28050	0.000975366	0.060849855	0.014208565	0.33058919		unknown protein ;supported by full-length cDNA: Ceres:6530.
AT2G25160	0.002685893	0.061530279	0.018690482	0.228510188	CYP82F1	putative cytochrome P450
AT2G43550	0.002108652	0.06292167	0.012604449	0.195740759		putative trypsin inhibitor ;supported by full-length cDNA: Ceres:85.
AT3G25790	0.000519851	0.063140214	0.011209538	0.374408084		unknown protein ;supported by full-length cDNA: Ceres:157740.
AT2G01530	0.00088781	0.06360184	0.011828709	0.348847963	MLP329	unknown protein ; supported by cDNA: gi_15450398_gb_AY052300.1_
AT1G22550	0.002677135	0.067709533	0.018669752	0.587609007		peptide transporter, putative similar to peptide transporter GI:9757725 from [Arabidopsis thaliana]
AT2G22930	0.000869684	0.068480088	0.005802488	0.353321483		putative flavonol 3-O-glucosyltransferase
AT1G52140	0.002141383	0.069417969	0.008271794	0.444678681		unknown protein ;supported by full-length cDNA: Ceres:25812.
AT4G03210	0.003597147	0.069855715	0.004857321	0.501934103	XTH9	putative xyloglucan endotransglycosylase ;supported by full-length cDNA: Ceres:17748.
AT4G25250	0.001182455	0.070355237	0.015512771	0.431199472		putative protein Group I Pectinesterase -Citrus sinensis, PID:g2098705;supported by full-length cDNA: Ceres:150586.
AT1G13300	0.000471695	0.071003847	0.0060313	0.244729505		unknown protein ; supported by cDNA: gi_13605874_gb_AF367336.1_AF367336
AT1G01070	0.000902111	0.071402499	0.015399912	0.647881221		unknown protein ;supported by full-length cDNA: Ceres:20749.
AT2G48080	0.002001894	0.072792542	0.006617936	0.211871724		unknown protein
AT1G30840	0.004940283	0.072877172	0.032110117	0.283242678	ATPUP4	hypothetical protein predicted by genscan+;supported by full-length cDNA: Ceres:113990.
AT2G45210	0.001688245	0.073370419	0.01212136	0.374672021		putative auxin-regulated protein
AT4G15390	0.000938459	0.073791491	0.003559839	0.142553721		HSR201 like protein
AT1G18590	0.001863618	0.076379612	0.007026021	0.205915372		flavonol 4'-sulfotransferase, putative similar to flavonol 4'-sulfotransferase GI:168168 from [Flaveria chloraefolia]
AT4G28250	0.00082477	0.077534286	0.0214611	0.495289475	ATEXPB3	putative beta-expansin/allergen protein soybean pollen allergen (cim1) protein - soybean, PIR2:S48032;supported by full-length cDNA: Ceres:33023.
AT2G24580	0.000902111	0.078021597	0.007229447	0.386114161		putative sarcosine oxidase
AT4G01450	0.001563781	0.078759978	0.025298882	0.516653472		predicted protein of unknown function similar to M. truncatula MtN21, GenBank accession number Y15293; F11O4.14 was picked up in a screen for marker I41G... (for example)
AT1G66140	0.001999927	0.078973933	0.005591058	0.418462078	ZFP4	C2H2-type zinc finger protein, putative contains Pfam profile: PF00096: Zinc finger, C2H2 type; supported by cDNA: gi_790678_gb_L39647.1_ATHZFPD
AT1G49470	0.001044592	0.081533675	0.04861288	0.690490761		unknown protein ;supported by full-length cDNA: Ceres:95546.
AT5G26310	0.000886423	0.081780994	0.022983318	0.520802682	UGT72E3	UTP-glucose glucosyltransferase - like protein UTP-glucose glucosyltransferase, cassava, PIR:S41951
AT2G01900	0.000424695	0.081855956	0.040638205	0.44000044		putative inositol polyphosphate-5-phosphatase
AT5G62720	0.00111891	0.082506086	0.005519112	0.150029473		putative protein predicted proteins, Arabidopsis thaliana; supported by full-length cDNA: Ceres: 38273.
AT5G65970	0.000710498	0.083017445	0.041526962	0.618312869	MLO10	Mlo protein-like ; supported by cDNA: gi_14091589_gb_AF369571.1_AF369571
AT1G05300	0.003598499	0.084106878	0.02292448	0.363593618	ZIP5	putative zinc transporter Similar to Arabidopsis Fe(II) transport protein (gb|U27590)
AT5G51490	0.002494718	0.085301095	0.020383343	0.383429232		pectinesterase
AT4G26130	0.000889151	0.088628227	0.00405207	0.392933299		uncharacterized protein ;supported by full-length cDNA: Ceres:7421.
AT5G14880	0.000735943	0.090284217	0.015866674	0.410729031		putative cation transport protein putative potassium transporter AtKT2p, Arabidopsis thaliana, EMBL:AF012657
AT1G15380	0.007760637	0.090326183	0.022239825	0.178723407		hypothetical protein Is a member of the PF|00903 glyoxalase family. ESTs gb|T44721, gb|T21844 and gb|AA395404 come from this gene; supported by full-length cDNA: Ceres: 5364.
AT5G62330	0.013616625	0.090396379	0.00870844	0.18238743		putative protein
AT1G73600 /// AT1G73602	0.006774846	0.090609051	0.026649283	0.227923968		phosphoethanolamine N-methyltransferase, putative similar to GI:7407189 from [Spinacia oleracea]
AT1G13080	0.002553323	0.09141311	0.02577877	0.289299299	CYP71B2	putative cytochrome P450 monooxygenase identical to gb|D78605 cytochrome P450 monooxygenase from Arabidopsis thaliana and is a member of the PF|00067 Cytochrome P450 family. ESTs gb|Z18072, gb|Z35218 and gb|T43466 come from this gene; supported by cDNA: g
AT3G16560	0.001389157	0.092125924	0.006778613	0.277489959		unknown protein contains protein phosphatase 2C domain; supported by full-length cDNA: Ceres: 249756.
AT1G72300	0.000979746	0.092531791	0.008786726	0.453343682		leucine-rich receptor-like protein kinase, putative similar to GI:3641252 from [Malus x domestica] (Plant Mol. Biol. 40 (6), 945-957 (1999))
AT3G48740	0.013091338	0.093214984	0.023611085	0.290145588		MTN3-like protein MtN3 gene product - Medicago truncatula,PID:e1169583; supported by cDNA: gi_13605687_gb_AF361825.1_AF361825
AT1G31710	0.002811891	0.094490692	0.025188968	0.577460126		copper amine oxidase, putative similar to copper amine oxidase GI:685197 from [Pisum sativum]
AT5G49660	0.008564035	0.098847273	0.010987509	0.564161112		receptor protein kinase
AT4G17340	0.001083083	0.10071274	0.027246432	0.603941978	DELTA-TIP2	membrane channel like protein ;supported by full-length cDNA: Ceres:99796.
AT1G70370	0.00026188	0.100772583	0.009807889	0.588791356		aromatic rich glycoprotein, putative similar to aromatic rich glycoprotein GI:1762428 from [Arabidopsis thaliana]; supported by cDNA: gi_1762583_gb_U63373.1_ATU63373
AT3G53100	0.000897589	0.100783877	0.018638603	0.562070041		putative protein proline-rich protein, Raphanus sativa, PIR:S16748
AT5G49360	0.005926923	0.101136368	0.009211053	0.317955288	BXL1	xylosidase
AT3G19710	0.00200049	0.102231478	0.016206117	0.15677516	BCAT4	branched-chain amino acid aminotransferase, putative similar to branched-chain amino acid aminotransferase GB:AAF07192 from [Solanum tuberosum];supported by full-length cDNA: Ceres:38004.
AT5G62680	0.000534231	0.10437045	0.005443438	0.338689551		peptide transporter
AT4G30450	0.001440628	0.105238255	0.03186935	0.395241763		glycine-rich protein glycine-rich cell wall structural protein - Petunia x hybrida,Pir2:A26099;supported by full-length cDNA: Ceres:115209.
AT4G27260	0.004206488	0.10837377	0.019147428	0.3684959	GH3.5	GH3 like protein GH3 protein, Glycine max., PIR2:S17433
AT2G28110	0.000492482	0.109034667	0.016711097	0.54020287	FRA8	hypothetical protein predicted by genscan
AT4G23410	0.001024281	0.110167053	0.010111201	0.486351281	TET5	hypothetical protein
AT1G66180	0.000715302	0.110353424	0.020093774	0.366229039		unknown protein ;supported by full-length cDNA: Ceres:99625.
AT1G07090	0.000470407	0.110558943	0.003559839	0.611800668		unknown protein ;supported by full-length cDNA: Ceres:28780.
AT1G31710	0.000881454	0.110698918	0.025188968	0.577460126		copper amine oxidase, putative similar to copper amine oxidase GI:685197 from [Pisum sativum]
AT1G25450 /// AT3G15520	0.000860735	0.111168215	0.01137017	0.681071696		fatty acid condensing enzyme CUT1, putative similar to fatty acid condensing enzyme CUT1 GI:5001734 from [Arabidopsis thaliana];supported by full-length cDNA: Ceres:99793.
AT3G48100	0.001178052	0.113010849	0.012853701	0.248763459	ARR5	response reactor 2 (ATRR2) ; supported by cDNA: gi_3273197_dbj_AB010916.1_AB010916
AT3G03680	0.011446694	0.114895263	0.015521187	0.426259549		putative phosphoribosylanthranilate transferase similar to phosphoribosylanthranilate transferase GB:CAA16616 [Arabidopsis thaliana], phosphoribosylanthranilate transferase GB:BAA13032 [Pisum sativum]
AT1G68710	0.002076549	0.115814881	0.021153206	0.288800265		ATPase, putative similar to chromaffin granule ATPase II GB:AAD03352 GI:4115341 from [Bos taurus]
AT4G34980	0.000679271	0.118393495	0.00869683	0.646006899	SLP2	subtilisin-like serine protease similar to SBT1, a subtilase from tomato plants GI:1771160 from [Lycopersicon esculentum]
AT2G15890	0.005681347	0.121044478	0.02459386	0.29598987	MEE14	unknown protein ;supported by full-length cDNA: Ceres:29905.
AT1G07560	0.001515321	0.121078495	0.011803189	0.266761171		protein kinase, putative contains Pfam profile: PF00069 Eukaryotic protein kinase domain (1 copy)
AT1G09750	0.003560312	0.121487153	0.014521212	0.32547803		hypothetical protein predicted by genscan;supported by full-length cDNA: Ceres:6295.
AT5G57480	0.000470407	0.121528759	0.01212136	0.484115704		putative protein contains similarity to AAA-type ATPase
AT5G15180	0.01163845	0.122411637	0.04215532	0.605291226		prx10 peroxidase - like protein prx10 peroxidase, Spinacia oleracea, EMBL:SOY16776
AT2G42760	0.000470407	0.12432405	0.018718654	0.380523962		unknown protein
AT3G07310	0.005648061	0.125120162	0.010260057	0.145114433		unknown protein ; supported by full-length cDNA: Ceres: 37200.
AT1G76790	0.000485256	0.127210949	0.013086765	0.599266234		putative catechol O-methyltransferase similar to catechol O-methyltransferase GB:CAA55358 [Vanilla planifolia];supported by full-length cDNA: Ceres:9016.
AT4G35030	0.000728709	0.129552247	0.010543715	0.417899837		protein kinase - like leucine rich repeat receptor-like kinase, Oryza sativa, PATCHX:E267533
AT5G62920	0.001446053	0.130099709	0.008131621	0.375701392	ARR6	response regulator 6 (ARR6) ; supported by cDNA: gi_3953600_dbj_AB008489.1_AB008489
AT5G52310	0.003852752	0.1307732	0.010408012	0.544670319	COR78	low-temperature-induced protein 78 (sp|Q06738) ; supported by cDNA: gi_348691_gb_L22567.1_ATHCOR78A
AT4G03610	0.006876917	0.131600062	0.015549773	0.486561606		putative hydrolase
AT4G21870	0.001084539	0.131659126	0.005686936	0.155355516		heat shock protein - like heat shock protein 17, Triticum aestivum, PIR1:HHWT17;supported by full-length cDNA: Ceres:23223.
AT2G01890	0.001446053	0.133792151	0.029733595	0.529673417	PAP8	putative purple acid phosphatase contains metallo-phosphoesterase motif (PS50185)
AT2G22330	0.001812016	0.13408149	0.00437724	0.050462522	CYP79B3	putative cytochrome P450
AT5G07580	0.000471695	0.13689224	0.004574726	0.433767969		transcription factor-like protein ethylene responsive element binding factor 5 - Arabidopsis thaliana, EMBL:AB008107; supported by cDNA: gi_15529283_gb_AY052266.1_
AT1G53830	0.00126274	0.13737277	0.032450467	0.639786465	ATPME2	pectin methylesterase (PMEU1), putative similar to pectin methylesterase (PMEU1) GI:1222551 from [Lycopersicon esculentum]; supported by cDNA: gi_13605622_gb_AF361637.1_AF361637
AT2G36690	0.00240443	0.137450043	0.004356068	0.128740502		putative giberellin beta-hydroxylase contains similarities to GA beta-20-hydroxylase from tobacco (GB:3327245) and to ethylene forming enzyme from Picea glauca (GB:L42466)
AT3G52060	0.002643562	0.137615127	0.004813203	0.230008987		putative protein hypothetical protein F10M6.70 - Arabidopsis thaliana,PIR3:T05399; supported by cDNA: gi_15293266_gb_AY051067.1_
AT5G65660	0.000722973	0.138150102	0.007220829	0.403920729		unknown protein ;supported by full-length cDNA: Ceres:25419.
AT1G76410	0.011352806	0.138416226	0.018938925	0.21702441	ATL8	putative RING zinc finger protein contains Pfam profile: PF00097 Zinc finger, C3HC4 type (RING finger); supported by full-length cDNA: Ceres: 27464.
AT3G62270	0.000424419	0.138941436	0.015866674	0.521212352		putative protein anion exchanger 3, cardiac splice form - Rattus norvegicus, PIR:A42497
AT4G29700	0.003458546	0.139357352	0.016954023	0.50442227		nucleotide pyrophosphatase -like protein nucleotide pyrophosphatase, Oryza sativa, gb:T03293; supported by cDNA: gi_13430713_gb_AF360269.1_AF360269
AT1G28100	0.003060474	0.13985375	0.018542966	0.558584261		unknown protein
AT4G12390	0.000836392	0.139894648	0.039489757	0.697600116	PME1	putative protein pectinesterase - Citrus sinensis,PID:g2098711;supported by full-length cDNA: Ceres:27615.
AT2G47160	0.005260802	0.14155663	0.00614898	0.231730914	BOR1	putative anion exchange protein identical to GP:3522933:AC004411
AT4G39950	0.001380261	0.143087037	0.005921167	0.1012442	CYP79B2	cytochrome P450 - like protein cytochrome P450, Sinapis alba, AF069494; supported by cDNA: gi_15028134_gb_AY046017.1_
AT1G08670	0.0007381	0.145608759	0.041115638	0.345665145		hypothetical protein predicted by genscan
AT5G57070	0.003066533	0.146993396	0.008484596	0.548182591		putative protein similar to unknown protein (gb AAF21150.1)
AT5G24030	0.003437925	0.148865778	0.04608828	0.428352681	SLAH3	putative protein contains similarity to unknown protein (pir||T02898)
AT2G28720	0.000586765	0.15116448	0.038470954	0.684696225		putative histone H2B ;supported by full-length cDNA: Ceres:14965.
AT2G29660	0.000182259	0.151537974	0.008618869	0.515313547		unknown protein ;supported by full-length cDNA: Ceres:106148.
AT5G63710	0.007163932	0.151661206	0.031476613	0.68299265		receptor-like protein kinase ; supported by cDNA: gi_16974576_gb_AY060554.1_
AT5G47560	0.000941794	0.152894655	0.03387688	0.62636073	ATSDAT	sodium-dicarboxylate cotransporter-like ;supported by full-length cDNA: Ceres:107593.
AT5G27930	0.004689048	0.156024837	0.009211053	0.563686673		protein phosphatase - like protein phosphatase-2C, Mesembryanthemum crystallinum, EMBL:AF075581;supported by full-length cDNA: Ceres:23348.
AT5G56870	0.004949881	0.156892302	0.008131621	0.424480576	BGAL4	beta-galactosidase (emb|CAB64740.1) ; supported by cDNA: gi_15451017_gb_AY054589.1_
AT1G12110	0.000446479	0.156913622	0.007475819	0.319599064	NRT1.1	putative NPK1-related protein kinase 2 similar to nitrate chlorate transporter GB:Q05085 from (Arabidopsis thaliana); supported by cDNA: gi_166667_gb_L10357.1_ATHCHL1A
AT3G25560	0.000471695	0.157579081	0.002512239	0.413986398	NIK2	somatic embryogenesis receptor-like kinase, putative similar to somatic embryogenesis receptor-like kinase GB:AAB61708 from [Daucus carota]
AT4G36380	0.004154107	0.158295467	0.006060074	0.255308404	ROT3	cytochrome P450 like protein ; supported by cDNA: gi_4176419_dbj_AB008097.1_AB008097
AT3G61430	0.000787158	0.161907897	0.007413642	0.566986122	PIP1A	plasma membrane intrinsic protein 1a ;supported by full-length cDNA: Ceres:6690.
AT1G02730	0.001140098	0.162270262	0.003654565	0.42682372	ATCSLD5	hypothetical protein similar to cellulose synthase catalytic subunit GB:AAC39336 GI:2827143 from (Arabidopsis thaliana)
AT3G11280	0.000644533	0.165067782	0.01212136	0.451504164		MYB-family transcription factor, putative contains Pfam profile: PF00249 Myb-like DNA-binding domain;supported by full-length cDNA: Ceres:109398.
AT1G61667	0.00748162	0.166406684	0.037689835	0.635238214		Expressed protein ; supported by full-length cDNA: Ceres: 1860.
AT4G28270	0.01197786	0.167918854	0.014730349	0.192645021		putative protein Arabidopsis thaliana RMA1 mRNA, PID:d1029538
AT1G25230	0.001950325	0.16896768	0.013073855	0.256237467		hypothetical protein similar to putative purple acid phosphatase precursor GI:7331195 from [Glycine max]
AT4G35750	0.001779904	0.168996669	0.007138968	0.331238767		putative protein E1B 19K/Bcl-2-interacting protein Nip2, Homo sapiens, PIR2:I38864;supported by full-length cDNA: Ceres:17117.
AT3G02640	0.000556665	0.16935956	0.01109956	0.400317324		unknown protein ;supported by full-length cDNA: Ceres:4861.
AT2G46650	0.000975477	0.169409511	0.012977538	0.313682534	B5 #1	putative cytochrome b5 ;supported by full-length cDNA: Ceres:3743.
AT1G15550	0.00105364	0.173467007	0.024614355	0.481295509	GA4	gibberellin 3 beta-hydroxylase, putative similar to gibberellin 3 beta-hydroxylase GI:3982753 from [Arabidopsis thaliana]; supported by cDNA: gi_1945343_gb_L37126.1_ATHGA4A
AT1G61740	0.000307524	0.176105139	0.009876199	0.228923578		unknown protein similar to hypothetical protein GI:4079632 from [Arabidopsis thaliana]
AT3G52370	0.003738204	0.176955634	0.005822355	0.579626276	FLA15	unknown protein
AT4G40070	0.000323927	0.177473249	0.002145344	0.151400615		putative protein RING-H2 zinc finger protein ATL6 - Arabidopsis thaliana, PID:g4928403; supported by cDNA: gi_16930492_gb_AF419600.1_AF419600
AT1G74940	0.005645529	0.177538913	0.022239825	0.593197109		hypothetical protein predicted by genemark.hmm;supported by full-length cDNA: Ceres:24864.
AT5G06200	0.001452713	0.178132729	0.006885885	0.457914142		putative protein similar to unknown protein (gb|AAF00668.1)
AT5G23010	0.006308351	0.178577149	0.004857321	0.06299617	MAM1	2-isopropylmalate synthase-like; homocitrate synthase-like ; supported by cDNA: gi_12330688_gb_AF327648.1_AF327648
AT2G16980	0.005746822	0.18010937	0.021950085	0.345576653		putative tetracycline transporter protein
AT1G15740	0.002403365	0.181144774	0.02336734	0.476987158		unknown protein ; supported by cDNA: gi_14423507_gb_AF386991.1_AF386991
AT1G15670	0.000972307	0.181652993	0.006460856	0.283195192		unknown protein ; supported by cDNA: gi_15146331_gb_AY049307.1_
AT1G11450 /// AT1G11460	0.00127196	0.181924916	0.007175001	0.690771411		hypothetical protein contains similarity to nodulin-like protein GI:9294338 from [Arabidopsis thaliana]
AT1G14720	0.000722973	0.182981093	0.015866674	0.440492871	XTR2	xyloglucan endo-transglycosylase, putative similar to xyloglucan endo-transglycosylase GB:CAA63553 GI:1769907 [Arabidopsis thaliana], identical to endoxyloglucan transferase related protein GB:BAA20290, GI:2154611 [Arabidopsis thaliana];supported by full-
AT5G65210	0.000975366	0.183653404	0.004922012	0.476673005	TGA1	DNA binding protein TGA1a homolog ;supported by full-length cDNA: Ceres:31032.
AT3G27210	0.000972307	0.183983297	0.010938161	0.501827304		unknown protein ; supported by cDNA: gi_15146182_gb_AY049232.1_
AT4G29720	0.002632539	0.184782121	0.012977538	0.283124265	ATPAO5	putative protein Cs protein, Drosophila melanogaster, AF091328;supported by full-length cDNA: Ceres:158028.
AT3G09220	0.000476072	0.184884279	0.011328709	0.516961133	LAC7	putative laccase similar to laccase GB:CAA74105 [Populus balsamifera subsp. trichocarpa]; contains Pfam profile: Multicopper oxidases
AT3G23800	0.002061708	0.185187311	0.013728089	0.306177717		selenium binding protein, putative similar to selenium binding protein 2 GB:Q63836 from [Mus musculus]
AT3G44990	0.000471695	0.185887095	0.007268087	0.304227281	XTR8	xyloglucan endo-transglycosylase ; supported by cDNA: gi_15810248_gb_AY056163.1_
AT1G72200	0.004223302	0.186441219	0.03659354	0.374447297		RING-H2 zinc finger protein ATL3, putative similar to GI:4928397 from [Arabidopsis thaliana] (Plant Mol. Biol. 40 (4), 579-590 (1999))
AT4G11210	0.003323533	0.187299112	0.044042446	0.542633051		putative disease resistance response protein disease resistance response protein 206-d - Pisum sativum, PID:g508844
AT1G11530	0.008100097	0.187991869	0.013242396	0.31536542	ATCXXS1	thioredoxin h, putative similar to thioredoxin h GI:4928460 from [Hevea brasiliensis];supported by full-length cDNA: Ceres:2054.
AT5G45480	0.001262677	0.18836726	0.01109956	0.687354904		putative protein similar to unknown protein (pir||T04430)
AT1G31180 /// AT5G14200	0.003015085	0.189108261	0.008856318	0.545117881		3-methyladenine DNA glycosylase, putative Strong similarity to gb|P29102, 3-isopropylmalate dehydrogenase (IMDH) from Brassica napus. EST gb|F14478 comes from this gene;supported by full-length cDNA: Ceres:34420.
AT1G08590	0.004120271	0.190958738	0.009974133	0.510724239		putative receptor kinase, CLV1 similar to receptor-like protein kinase (Ipomoea nil) (U77888)
AT5G06610	0.001957841	0.191733723	0.008730779	0.472185221		putative protein similar to unknown protein (gb|AAD30234.1); supported by full-length cDNA: Ceres: 6949.
AT1G32640	0.005369366	0.191750973	0.021916801	0.423960159	ATMYC2	protein kinase, putative identical to bHLH protein GB:CAA67885 GI:1465368 from [Arabidopsis thaliana]; supported by cDNA: gi_14335047_gb_AY037203.1_
AT2G01670	0.001562901	0.191900623	0.044305597	0.344130781	ATNUDT17	unknown protein ;supported by full-length cDNA: Ceres:8247.
AT2G16660	0.003297276	0.192078454	0.037883233	0.695230924		nodulin-like protein
AT1G75500	0.002718624	0.192961475	0.014052022	0.641085599		nodulin-like protein similar to MtN21 GB:CAA75575 GI:2598575 from [Medicago truncatula] (Mol. Plant Microbe Interact. 9 (4), 233-242 (1996)); supported by cDNA: gi_15028078_gb_AY045896.1_
AT4G04640	0.01359119	0.193749524	0.013963202	0.331750772	ATPC1	coded for by A. thaliana cDNA AA041141 ;supported by full-length cDNA: Ceres:6687.
AT1G28440	0.001610729	0.193946503	0.042827006	0.645537785	HSL1	receptor kinase, putative similar to receptor kinase GI:4105699 from [Arabidopsis thaliana]
AT2G15620	0.0015256	0.195217617	0.006060074	0.417482183	NIR1	ferredoxin--nitrite reductase ; supported by cDNA: gi_15010613_gb_AY045608.1_
AT1G15210	0.000519851	0.195845172	0.02787128	0.617181544	ATPDR7	putative ABC transporter Similar to gb|Z70524 PDR5-like ABC transporter from Spirodela polyrrhiza and is a member of the PF|00005 ABC transporter family. ESTs gb|N97039 and gb|T43169 come from this gene
AT1G72360	0.000679271	0.196362491	0.009099189	0.302015239		putative AP2 domain transcription factor contains Pfam profile: PF00847 AP2 domain
AT3G04530	0.002494718	0.197096182	0.016263295	0.423185845	PPCK2	phosphoenolpyruvate carboxylase kinase, putative similar to phosphoenolpyruvate carboxylase kinase GB:AAF06968 from [Arabidopsis thaliana]; supported by cDNA: gi_13877127_gb_AF358915.1_AF358915
AT1G27210	0.001612004	0.198693748	0.017363697	0.624133936		unknown protein supported by cDNA: Ceres: 103034, cDNA may not be full-length in this case.; supported by cDNA: gi_15982865_gb_AY057540.1_
AT1G78370	0.001276096	0.199142639	0.03835354	0.583601177	ATGSTU20	2,4-D inducible glutathione S-transferase, putative similar to 2,4-D inducible glutathione S-transferase GI:2920666 from [Glycine max];supported by full-length cDNA: Ceres:30680.
AT1G44110	0.005681347	0.20055212	0.002705098	0.336067319	CYCA1;1	mitotic cyclin a2-type, putative similar to mitotic cyclin a2-type GI:857396 from [Glycine max]
AT5G01320 /// AT5G01330	0.000297554	0.200915209	0.005383498	0.448666979	PDC3	pyruvate decarboxylase-like protein pyruvate decarboxylase (EC 4.1.1.1) pdc1 - Arabidopsis thaliana, PIR:T05315
AT4G11190	0.004452344	0.201852033	0.032123867	0.512609764		putative disease resistance response protein disease resistance response protein 206-d - Pisum sativum, PID:g508844
AT3G23430	0.001307835	0.204295142	0.010716326	0.490711205	PHO1	unknown protein
AT1G11670	0.004858039	0.204669368	0.014301122	0.5106017		unknown protein strong similarity to gi|3367522 F8K4.9 from Arabidopsis thaliana BAC gb|AC004392. EST gb|W43487 comes from this gene
AT5G57630	0.00088781	0.204674998	0.034684673	0.577520355	CIPK21	SNF1 related protein kinase-like protein ; supported by cDNA: gi_14334389_gb_AY034100.1_
AT5G42590	0.025745204	0.205003382	0.024510367	0.231804704	CYP71A16	cytochrome P450
AT2G45720	0.001322674	0.205541573	0.00562872	0.656788653		unknown protein
AT3G19030	0.000876234	0.205712471	0.006885885	0.328910128		hypothetical protein contains similarity to phosphoserine aminotransferase GB:P19689 from [Yersinia enterocolitica];supported by full-length cDNA: Ceres:30768.
AT3G50300	0.002450444	0.205936715	0.002808379	0.576559224		anthranilate N-hydroxycinnamoyl/benzoyltransferase - like protein anthranilate N-hydroxycinnamoyl/benzoyltransferase, Dianthus caryophyllus, PIR:T10719
AT1G77760	0.001952861	0.206120119	0.00869683	0.430419391	NIA1	nitrate reductase 1 (NR1) identical to nitrate reductase 1 (NR1) GB:P11832 [Arabidopsis thaliana]; supported by cDNA: gi_15983498_gb_AF424624.1_AF424624
AT5G64570	0.001744274	0.206567269	0.007876929	0.226394881	XYL4	beta-xylosidase
AT1G56010	0.001578146	0.207196515	0.006200098	0.340616045	NAC1	NAC1 identical to NAC1 GB:AAF21437 GI:6649236 from [Arabidopsis thaliana];supported by full-length cDNA: Ceres:205648.
AT3G46630	0.006000143	0.208216355	0.015995756	0.653775906		putative protein DCL PROTEIN, CHLOROPLAST PRECURSOR (DEFECTIVE CHLOROPLASTS AND LEAVES PROTEIN) - Lycopersicon esculentum, SWISSPROT:DCL_LYCES;supported by full-length cDNA: Ceres:21432.
AT1G60140	0.000999676	0.20860539	0.007426498	0.300212112	ATTPS10	trehalose-6-phosphate synthase, putative similar to trehalose-6-phosphate synthase GI:297792 from [Kluyveromyces lactis]
AT5G23020	0.001972574	0.209579458	0.00826408	0.427497183	MAM-L	2-isopropylmalate synthase-like protein
AT5G60660	0.004194414	0.210286235	0.018233245	0.495646636	PIP2;4	mipC protein - like (aquaporin) mipC protein, common ice plant, PIR:T12440;supported by full-length cDNA: Ceres:33231.
AT1G61590	0.001176596	0.211052303	0.028119983	0.492828119		serine/threonine protein kinase, putative similar to serine/threonine protein kinase GI:1066501 from [Arabidopsis thaliana]
AT4G19160	0.001677429	0.211584165	0.012977538	0.383191871		putative protein ; supported by cDNA: gi_15292760_gb_AY050814.1_
AT1G57590	0.002596364	0.21337386	0.004097793	0.549881149		pectinacetylesterase precursor, putative similar to pectinacetylesterase precursor GI:1431629 from [Vigna radiata]
AT4G26220	0.001439311	0.213998576	0.039542343	0.648149636		caffeoyl-CoA O-methyltransferase - like protein caffeoyl-CoA O-methyltransferase, Pinus taeda, AF036095;supported by full-length cDNA: Ceres:33227.
AT4G02810	0.013027597	0.214133941	0.007614888	0.205413171		hypothetical protein
AT3G02120	0.005743616	0.215441837	0.011209538	0.448684251		Expressed protein ; supported by full-length cDNA: Ceres: 5806.
AT4G04040	0.003765744	0.215592838	0.014208565	0.247046351	MEE51	putative phosphofructokinase beta subunit
AT1G15100	0.000993292	0.215841781	0.009289766	0.598215821	RHA2A	putative RING-H2 zinc finger protein ESTs gb|N37587, gb|T04684, gb|AA394318, gb|Z35014 and gb|AA713343 come from this gene;supported by full-length cDNA: Ceres:5398.
AT5G25830	0.002459923	0.217213789	0.024017146	0.637335347		GATA transcription factor - like GATA transcription factor, Arabidopsis thaliana, PIR:T05288
AT4G31500	0.00088781	0.218506232	0.004857321	0.235483369	CYP83B1	cytochrome P450 monooxygenase ;supported by full-length cDNA: Ceres:13745.
AT1G77690	0.000287634	0.219410535	0.005859572	0.49257313		putative AUX1-like permease similar to AUX1 (regulator of root gravitropism, putative permease) GB:CAA67308 [Arabidopsis thaliana]; supported by cDNA: gi_15809995_gb_AY054266.1_
AT1G01430	0.001651861	0.22095961	0.046336472	0.695141759		hypothetical protein similar to hypothetical protein GB:CAB80917 GI:7267605 from [Arabidopsis thaliana]
AT5G62350	0.001445556	0.224175237	0.014346944	0.624631975		ripening-related protein - like ripening-related protein, Vitis vinifera, EMBL:VVI237985;supported by full-length cDNA: Ceres:9669.
AT1G74840	0.003171321	0.224535991	0.005738346	0.649886468		myb-related transcription activator, putative similar to myb-related transcription activator GI:9279717 from [Arabidopsis thaliana];supported by full-length cDNA: Ceres:117643.
AT3G54900	0.001353658	0.224885769	0.029213972	0.643268297	CXIP1	putative protein thioredoxin-like protein, Homo sapiens, AJ010841; supported by cDNA: gi_14326509_gb_AF385708.1_AF385708
AT2G34190	0.005453883	0.226042813	0.031176118	0.67313269		putative membrane transporter ; supported by cDNA: gi_14334907_gb_AY035127.1_
AT2G42850	0.036002293	0.227900671	0.027508326	0.231845377	CYP718	putative cytochrome P450
AT2G35000	0.004377057	0.230825584	0.010607494	0.493053031		putative RING zinc finger protein
AT1G75440	0.00205078	0.23176418	0.04248663	0.582872762	UBC16	E2, ubiquitin-conjugating enzyme 16 (UBC16) identical to gi:2801444, GB:AAC39325 from [Arabidopsis thaliana] (Plant Mol. Biol. 23 (2), 387-396 (1993))
AT2G32270	0.001157079	0.232179148	0.012320777	0.342911456	ZIP3	putative Fe(II) transporter identical to GB:AF033537; supported by cDNA: gi_3252869_gb_AF033537.1_AF033537
AT1G78260	0.001380261	0.232479345	0.03161624	0.647522869		RNA recognition motif-containing protein SEB-4 similar to RNA recognition motif-containing protein SEB-4 GI:8895698 from [Xenopus laevis];supported by full-length cDNA: Ceres:123429.
AT1G72160	0.001688245	0.233500777	0.016155839	0.559830295		cytosolic factor, putative similar to GI:807956 from [Saccharomyces cerevisiae]; supported by cDNA: gi_15081613_gb_AY048199.1_
AT2G01950	0.002217041	0.233872366	0.002831014	0.522499344	BRL2	putative receptor protein kinase similar to brassinosteroid insensitive protein
AT3G61490	0.001167245	0.235121069	0.008060786	0.403719141		putative protein polygalacturonase - Zea mays, EMBL:ZMPGAL3
AT1G77380	0.00993399	0.235915881	0.008943213	0.128876924	AAP3	amino acid carrier, putative similar to amino acid carrier GI:3293031 from [Ricinus communis];supported by full-length cDNA: Ceres:15825.
AT3G12870	0.000302628	0.237065173	0.019492567	0.537945607		hypothetical protein similar to oxidoreductases
AT4G30190	0.000686013	0.240449872	0.003083379	0.583252465	AHA2	H+-transporting ATPase type 2, plasma membrane ; supported by cDNA: gi_14334803_gb_AY035075.1_
AT5G63600	0.003060474	0.240691468	0.011686415	0.562264085		1-aminocyclopropane-1-carboxylic acid oxidase-like protein
AT3G47500	0.003499105	0.241282173	0.012667717	0.582366343	CDF3	H-protein promoter binding factor-2a ;supported by full-length cDNA: Ceres:113639.
AT1G43650	0.000470407	0.241733558	0.02077622	0.640420239		nodulin-like protein similar to MtN21 GB:CAA75575 GI:2598575 from [Medicago truncatula] (Mol. Plant Microbe Interact. 9 (4), 233-242 (1996))
AT5G50760	0.004377057	0.242167709	0.014067003	0.53231991		putative protein similar to unknown protein (emb|CAB86483.1)
AT1G60960	0.004223302	0.242628733	0.020383343	0.588507808	IRT3	putative iron-regulated transporter similar to iron-regulated transporter 1 GB:AAD30548 from [Lycopersicon esculentum];supported by full-length cDNA: Ceres:15980.
AT1G70300	0.000471695	0.242715	0.02338637	0.668547692	KUP6	potassium transporter, putative similar to potassium transporter GI:2654088 from [Arabidopsis thaliana]
AT2G40230	0.002697748	0.242721545	0.005143304	0.182344045		putative anthranilate N-hydroxycinnamoyl/benzoyltransferase ;supported by full-length cDNA: Ceres:105546.
AT1G55360	0.003347591	0.243662877	0.008768849	0.670652803		unknown protein
AT4G20320	0.015758706	0.24414019	0.01127415	0.351456974		CTP synthase like protein CTP synthase, Methanococcus jannaschii,PIR2:E64446
AT5G67210	0.000722973	0.244325898	0.02632178	0.685306125		putative protein similar to unknown protein (emb|CAB62301.1)
AT5G53370	0.001877999	0.246886331	0.015042232	0.491491906	ATPMEPCRF	pectinesterase ; supported by cDNA: gi_15293286_gb_AY051077.1_
AT3G50660	0.005204357	0.248700435	0.014154107	0.282994482	DWF4	steroid 22-alpha-hydroxylase (DWF4) ; supported by cDNA: gi_15724347_gb_AF412114.1_AF412114
AT2G31730	0.006015687	0.249119766	0.001089257	0.546114659		unknown protein
AT3G16240	0.003133086	0.250915503	0.01467628	0.681296417	DELTA-TIP	delta tonoplast integral protein (delta-TIP) identical to delta tonoplast integral protein (delta-TIP) GB:U39485 [Arabidopsis thaliana] (Plant Cell 8 (4), 587-599 (1996)); supported by full-length cDNA: Ceres: 19689.
AT3G51950	0.001167618	0.251029995	0.011227544	0.632768619		putative protein ;supported by full-length cDNA: Ceres:7104.
AT4G39350	0.000728709	0.253394614	0.006341341	0.604427662	CESA2	cellulose synthase catalytic subunit (Ath-A) ; supported by cDNA: gi_2827140_gb_AF027173.1_AF027173
AT3G21770	0.001100269	0.25342608	0.006927837	0.501166113		putative peroxidase almost identical to GB:CAA66965 and GB:CAA67360 from [Arabidopsis thaliana];supported by full-length cDNA: Ceres:118626.
AT4G12510 /// AT4G12520	0.001044592	0.253576937	0.01212136	0.637295501		pEARLI 1-like protein Arabidopsis thaliana pEARLI 1 mRNA, completecds, PID:g871780
AT2G31110	0.00088781	0.254512863	0.005606713	0.543278517		hypothetical protein predicted by grail
AT4G29080	0.000609375	0.256288014	0.018134188	0.545822003	PAP2	phytochrome-associated protein PAP2 ; supported by cDNA: gi_12083213_gb_AF332402.1_AF332402

AT5G09440	0.001030927	0.256740451	0.002808379	0.498205861		putative protein phi-1 – Nicotiana tabacum, EMBL:AB018441;supported by full-length cDNA: Ceres:38576.
AT5G24165	0.002549788	0.257153708	0.000925163	0.540858916		Expressed protein ; supported by full-length cDNA: Ceres: 19092.
AT1G69580	0.002652926	0.257415364	0.023085084	0.509853141		transfactor, putative similar to GI:4519671 from [Nicotiana tabacum]
AT1G23390	0.00127196	0.258715545	0.012700422	0.298244473		unknown protein similar to hypothetical protein GB:AAF27090 GI:6730669 from (Arabidopsis thaliana);supported by full-length cDNA: Ceres:101865.
AT4G09760	0.004520424	0.260655574	0.022267781	0.542619006		choline kinase GmCK2p -like protein choline kinase GmCK2p -Glycine max,PID:g1438881;supported by full-length cDNA: Ceres:126037., Ceres:7395.
AT1G53510	0.000628484	0.261444586	0.043238223	0.683310143	ATMPK18	MAP kinase ATMPK9, putative similar to GI:7106544 from (Arabidopsis thaliana)
AT2G03890	0.001900889	0.262523114	0.01168574	0.626437988		unknown protein ; supported by cDNA: gi_15810174_gb_AY056101.1_
AT5G43350 /// AT5G43370	0.000817772	0.264448753	0.035156973	0.608180946	ATPT1	inorganic phosphate transporter (dbj|BAA24282.1)
AT2G28550	0.001993154	0.265961337	0.040544126	0.597144479	TOE1	putative AP2 domain transcription factor pFAM domain (PF00847); supported by cDNA: gi_15292762_gb_AY050815.1_
AT2G24150	0.001320022	0.26651996	0.014356068	0.493902722	HHP3	hypothetical protein predicted by genefinder; supported by cDNA: gi_13877832_gb_AF370179.1_AF370179
AT1G64390	0.001206966	0.268389657	0.025626075	0.675025052	ATGH9C2	endo-beta-1,4-glucanase, putative similar to GI:4972236 from [Fragaria x ananassa] (Plant Mol. Biol. 40, 323-332 (1999)); supported by cDNA: gi_13937172_gb_AF372940.1_AF372940
AT3G10230	0.001990669	0.269014011	0.00495198	0.454459644	LYC	lycopene beta cyclase identical to lycopene beta cyclase GB:AAB53337 [Arabidopsis thaliana]; supported by cDNA: gi_1399182_gb_U50739.1_ATU50739
AT1G05570	0.007429038	0.271156564	0.01397186	0.402043345	CALS1	putative glucan synthase Highly similar to putative glucan synthase GB:AAD15408; supported by cDNA: gi_13649387_gb_AF237733.1_AF237733
AT2G47500	0.007386654	0.271644276	0.036193896	0.632928177		putative kinesin heavy chain
AT5G56040	0.000302628	0.272692086	0.005606713	0.605357877		receptor protein kinase-like protein
AT1G44970	0.001541871	0.27432607	0.015349816	0.335525613		peroxidase, putative similar to peroxidase GI:993004 from [Mercurialis annua];supported by full-length cDNA: Ceres:26375.
AT2G20610	0.000708953	0.274675901	0.004294924	0.331354649	SUR1	putative tyrosine aminotransferase ; supported by cDNA: gi_15293106_gb_AY050987.1_
AT5G11090	0.003832129	0.27622533	0.04420193	0.697699455		putative protein predicted proteins, Arabidopsis thaliana;supported by full-length cDNA: Ceres:32958.
AT2G45220	0.003054162	0.276840948	0.030843703	0.60194864		putative pectinesterase ; supported by cDNA: gi_13605695_gb_AF361829.1_AF361829
AT1G30510	0.002060783	0.280575505	0.01109956	0.402426552	ATRFNR2	ferrodoxin NADP oxidoreductase, putative similar to ferrodoxin NADP oxidoreductase GB:X99419 GI:1480346 from [Pisum sativum]supported by full-length cDNA: Ceres:40436.
AT2G38400	0.000938459	0.281846212	0.014458521	0.404996064	AGT3	putative beta-alanine-pyruvate aminotransferase ;supported by full-length cDNA: Ceres:147543.
AT1G66800	0.036520753	0.281959114	0.039984275	0.633721192		cinnamyl alcohol dehydrogenase, putative similar to cinnamyl alcohol dehydrogenase [Eucalyptus gunnii] GI:1143445
AT5G27100	0.004029455	0.28265169	0.0087585	0.639523499	ATGLR2.1	ion channel – like protein ligand gated channel-like protein, Brassica napus, EMBL:AF109392
AT3G14415 /// AT3G14420	0.010980334	0.282704844	0.009211053	0.419960734		putative glycolate oxidase similar to glycolate oxidase GB:BAA03131 from [Cucurbita sp.]
AT5G67150	0.000722973	0.283636655	0.027508326	0.571371695		anthranilate N-hydroxycinnamoyl/benzoyltransferase-like protein ;supported by full-length cDNA: Ceres:114226.
AT5G06850	0.022701401	0.285898192	0.012977538	0.649214019		anthranilate phosphoribosyltransferase-like protein ; supported by cDNA: gi_16323171_gb_AY057690.1_
AT3G14050	0.00797539	0.286294515	0.012977538	0.624507069	RSH2	putative GTP pyrophosphokinase similar to GTP PYROPHOSPHOKINASE GB:O87331 from [Corynebacterium glutamicum]; supported by cDNA: gi_7141305_gb_AF225703.1_AF225703
AT4G10270	0.00180762	0.286934635	0.030307932	0.204416358		probable wound-induced protein wound-induced protein, Lycopersicon esculentum, PIR2:S19773;supported by full-length cDNA: Ceres:1831.
AT1G75780	0.006749178	0.28798868	0.014753928	0.697203822	TUB1	tubulin beta-1 chain identical to GB:P12411 GI:135442 from [Arabidopsis thaliana](Gene 63 (1), 87-102 (1988))
AT3G49940	0.000769637	0.289015604	0.005671362	0.648644006	LBD38	putative protein hypothetical protein – Arabidopsis thaliana, EMBL:CAB38293;supported by full-length cDNA: Ceres:17840.
AT5G01600	0.004433525	0.292113858	0.02195779	0.498920038	ATFER1	ferritin 1 precursor ;supported by full-length cDNA: Ceres:1100.
AT4G15830	0.001577863	0.293725204	0.004857321	0.418850834		hypothetical protein ;supported by full-length cDNA: Ceres:39757.
AT4G28650	0.000263285	0.295088244	0.015989691	0.682106558		receptor protein kinase-like protein receptor-like protein kinase 5, Arabidopsis thaliana, PIR1:S27756
AT2G37170 /// AT2G37180	0.003832129	0.295362313	0.005443438	0.59588708	RD28	aquaporin (plasma membrane intrinsic protein 2C) water channel protein in plasma membrane;supported by full-length cDNA: Ceres:11998.
AT3G23570	0.000471695	0.295410258	0.027508326	0.643317501		unknown protein contains Pfam profile: PF01738 dienelactone hydrolase family; supported by cDNA: gi_13899072_gb_AF370531.1_AF370531
AT1G22030	0.0037144	0.295887157	0.043392863	0.6574727		hypothetical protein predicted by genscan+;supported by full-length cDNA: Ceres:39052.
AT1G34460 /// AT5G06150	0.003171321	0.298046922	0.03323194	0.631312932	CYCB1;4	cyclin, putative similar to GB:AAB02028 from [Arabidopsis thaliana]
AT5G60200	0.000778458	0.298712754	0.00695367	0.515799187		zinc finger protein – like dof6 zinc finger protein, Arabidopsis thaliana, PIR:T47501;supported by full-length cDNA: Ceres:20852.
AT1G18250	0.002116537	0.298865119	0.034977432	0.670239804	ATLP-1	pathogenesis-related group 5 protein, putative similar to pathogenesis-related group 5 protein GI:2749943 from [Brassica rapa]; supported by cDNA: gi_536824_gb_L34693.1_ATHTLP
AT5G10390	0.002507344	0.299169986	0.004813203	0.516227295		histone H3 – like protein histone H3, Arabidopsis thaliana, PIR:S06250
AT1G32700	0.007470974	0.301500026	0.013718592	0.376818687		unknown protein similar to hypothetical protein GB:AAF25968 GI:6714272 from [Arabidopsis thaliana];supported by full-length cDNA: Ceres:206224.
AT4G37540	0.002667186	0.303483477	0.008999672	0.469748127	LBD39	putative protein predicted protein, Arabidopsis thaliana;supported by full-length cDNA: Ceres:33704.
AT2G16230	0.005147143	0.304138714	0.00655279	0.687480836		putative beta-1,3-glucanase
AT5G06800	0.004491645	0.304803521	0.010792479	0.512884189		putative protein contains similarity to transfactor
AT5G12950 /// AT5G12960	0.010548574	0.304982468	0.023984758	0.54903874		putative protein putative secreted protein SCF41.30c, Streptomyces coelicolor, EMBL:SCF41_30
AT2G38170	0.00199188	0.305067181	0.018554265	0.59316238	CAX1	high affinity Ca2+ antiporter identical to GB:U57411, except a possible frameshift at base 58008. Sequence has been confirmed with 5 sequencing reads.; supported by cDNA: gi_13937168_gb_AF372938.1_AF372938
AT1G02810	0.001736286	0.305735273	0.007026021	0.426025107		pectin esterase, putative similar to pectin esterase GI:1213628 from [Prunus persica]
AT3G62390	0.003223656	0.306017385	0.036169484	0.678991724		putative protein several hypothetical proteins – Arabidopsis thaliana;supported by full-length cDNA: Ceres:126100.
AT2G43910 /// AT2G43920	0.00204376	0.308314211	0.046157576	0.678440366		unknown protein
AT1G56220	0.001725322	0.309239973	0.02217697	0.529046365		unknown protein ; supported by full-length cDNA: Ceres: 22928.
AT1G11580	0.001444793	0.309312649	0.008639098	0.626714534	ATPMEPCRA	pectin methylesterase, putative similar to pectin methylesterase GI:1617583 from [Lycopersicon esculentum]; supported by cDNA: gi_14334991_gb_AY037175.1_
AT1G72810	0.002782701	0.311321512	0.005060274	0.428780116		threonine synthase, putative similar to threonine synthase GI:4850369 from [Arabidopsis thaliana]
AT3G26510	0.009989695	0.314533558	0.019935058	0.255682767		unknown protein
AT3G02040	0.006355985	0.316111835	0.029503902	0.581292169	SRG3	hypothetical protein predicted by genefinder;supported by full-length cDNA: Ceres:40090.
AT5G40830	0.003828674	0.316519932	0.019157518	0.458364254		putative protein similar to unknown protein (emb|CAB80933.1)
AT3G50340	0.005144388	0.317285508	0.008290684	0.495773875		putative protein predicted protein, Arabidopsis thaliana
AT2G32280	0.003427175	0.318435032	0.01212136	0.577582565		hypothetical protein predicted by genscan
AT1G25550	0.000998879	0.322127485	0.005571554	0.406935284		hypothetical protein similar to putative two-component response regulator protein GI:6623883 from [Arabidopsis thaliana];supported by full-length cDNA: Ceres:1959.
AT3G58710	0.003975459	0.322648276	0.025426172	0.691769889	WRKY69	DNA-binding WRKY – like protein AR411 protein, Arabidopsis thaliana, EMBL:ATD748; supported by full-length cDNA: Ceres: 100465.
AT4G11050	0.009219267	0.322718029	0.025188968	0.585995035	ATGH9C3	putative glucanase endo-beta-1,4-D-glucanase (Cel8) – Lycopersiconesculentum (tomato), PID:g4165132
AT4G05190	0.002086512	0.324067981	0.004813203	0.590050463	ATK5	kinesin – like protein kinesin like protein A, Arabidopsis thaliana, gb:Q07970
AT1G24280	0.007028592	0.32412944	0.007637197	0.303705228	G6PD3	glucose-6-phosphate 1-dehydrogenase strongly similar to GB:S71245, location of EST gb|Z35060 and gb|T04591
AT2G25060	0.007530315	0.325108923	0.012696948	0.47122813		similar to early nodulins
AT1G22880	0.017259173	0.326755388	0.017523976	0.637022323	ATCEL5	putative endo-1,4-beta-D-glucanase similar to GB:AAB65156 and GB:AAA96135
AT1G19000	0.002633012	0.328243653	0.021802861	0.572021447		Myb-related transcription activator, putative similar to MybSt1 GI:7705206 from [Solanum tuberosum];supported by full-length cDNA: Ceres:41381.
AT5G06270	0.001805252	0.329588679	0.022468494	0.508278639		putative protein similar to unknown protein (gb|AAF02129.1);supported by full-length cDNA: Ceres:104017.
AT5G36710 /// AT5G36800	0.001328039	0.329877841	0.040297158	0.595639904		putative protein similar to unknown protein (gb|AAF32471.1)
AT2G25450	0.003149439	0.330353173	0.048418745	0.671889352		putative dioxygenase ; supported by cDNA: gi_15292706_gb_AY050787.1_
AT5G48110	0.012947262	0.331902369	0.02838752	0.410618597		terpene synthase
AT5G19110	0.009291781	0.33203673	0.022141106	0.613874296		dermal glycoprotein – like dermal glycoprotein precursor, carrot, PIR:T14329
AT5G60490	0.012666835	0.332313408	0.027508326	0.556795012	FLA12	arabinogalactan protein – like arabinogalactan protein Pop14A9, Populus alba x Populus tremula, EMBL:AF183809;supported by full-length cDNA: Ceres:15239.
AT1G78120	0.004763732	0.332401766	0.024163691	0.445437473		hypothetical protein similar to unknown protein GB:AAD22995 [Arabidopsis thaliana]
AT3G51720	0.006422833	0.333053369	0.03542568	0.434144284		putative protein several putative myosin heavy chain-like proteins – Arabidopsis thaliana;supported by full-length cDNA: Ceres:99756.
AT5G08020	0.001611023	0.333529026	0.014301122	0.472875081		replication factor A – like protein replication protein A1 (Os-RPA1), Oryza sativa, EMBL:AF009179
AT5G55170	0.008106869	0.334375772	0.03988024	0.664213196	SUM3	ubiquitin-like protein
AT1G05260	0.002608024	0.337597311	0.032110117	0.669955254	RCI3	putative peroxidase Strong similarity to Arabidopsis peroxidase ATPEROX7A (gb|X98321);supported by full-length cDNA: Ceres:114862.
AT5G42180	0.001599981	0.337716944	0.012320777	0.614961824		peroxidase (emb|CAA66960.1)
AT3G61820	0.001244453	0.337778247	0.001810002	0.603849784		putative protein nucleoid DNA-binding protein cnd41, chloroplast – Nicotiana tabacum, EMBL:D26015; supported by cDNA: gi_14532549_gb_AY039899.1_
AT1G10140	0.01394511	0.338349431	0.018036846	0.370321611		unknown protein similar to EST gb|AA598098;supported by full-length cDNA: Ceres:23916.
AT2G31085	0.004666241	0.340492274	0.019510278	0.315798815	CLE6	CLE4 CLAVATA3/ESR-Related 4 (CLE4); supported by full-length cDNA: Ceres: 270513.
AT2G37440	0.004249801	0.340517283	0.003559839	0.582294147		unknown protein
AT4G33260 /// AT4G33270	0.001999927	0.340683872	0.039218377	0.592311372	CDC20.1	WD-repeat protein -like protein WD-repeat protein -Daucus carota,PID:g2253631; supported by cDNA: gi_6491863_gb_AF029263.1_AF029263
AT4G21350	0.0108643	0.340926842	0.03154573	0.668222645	B80	putative protein arm repeat containing protein (ARC1), Brassica napus, PID:g2558938
AT3G54400	0.001229917	0.341740211	0.006617936	0.649307812		nucleoid DNA-binding – like protein nucleoid DNA-binding protein cnd41, chloroplast, common tobacco, PIR:T01996; supported by full-length cDNA: Ceres: 8987.
AT1G53140	0.012886987	0.345606512	0.037870865	0.502565749		dynamin-like protein similar to GB:U25547 from [Glycine max]
AT2G45180	0.003210347	0.345909173	0.014672298	0.549069248		unknown protein identical to GB:AAB82643supported by full-length cDNA: Ceres:17187.
AT1G21050	0.001322513	0.346878078	0.014598037	0.617979406		hypothetical protein predicted by genemark.hmm;supported by full-length cDNA: Ceres:3200.
AT5G47720	0.001321425	0.347349885	0.025260273	0.564103542		acetoacyl-CoA-thiolase ;supported by full-length cDNA: Ceres:93821.
AT5G47950	0.015838902	0.347441098	0.027754312	0.53292774		acetyl-CoA:benzylalcohol acetyltranferase-like protein
AT2G10560 /// AT5G04860	0.001652505	0.347566796	0.037583027	0.680712256		unknown protein
AT3G47160	0.007380267	0.349710534	0.03460869	0.547526685		RNA-binding protein-like protein various RNA-binding proteins;supported by full-length cDNA: Ceres:9763.
AT2G38760	0.04338362	0.349934849	0.003341037	0.440334111	ANNAT3	putative annexin ;supported by full-length cDNA: Ceres:3294.
AT2G45830	0.002457038	0.350077322	0.028494747	0.65500882	DTA2	unknown protein
AT2G27360	0.001578146	0.351911232	0.018669752	0.546607888		putative lipase
AT1G66200	0.00093031	0.35372081	0.01502979	0.661142731	ATGSR2	glutamine synthetase, putative similar to glutamine synthetase [Raphanus sativus] GI:1526564;supported by full-length cDNA: Ceres:2662.
AT5G66490	0.040996823	0.354081531	0.01851467	0.284329948		putative protein similar to unknown protein (pir||T08411);supported by full-length cDNA: Ceres:93374.
AT1G63800	0.001972994	0.356349915	0.039433565	0.690592902	UBC5	E2, ubiquitin-conjugating enzyme 5 (UBC5) identical to gi:431269, SP:P42749
AT3G17998 /// AT3G18000	0.000644533	0.356627848	0.029733595	0.554424068	NMT1	methyltransferase, putative similar to methyltransferase GB:AAC01738 from [Amycolatopsis mediterranei]
AT3G17998 /// AT3G18000	0.000644533	0.356627848	0.020355968	0.530762969	NMT1	methyltransferase, putative similar to methyltransferase GB:AAC01738 from [Amycolatopsis mediterranei]
AT3G22240	0.001225199	0.358506269	0.024409804	0.663723892		unknown protein ; supported by full-length cDNA: Ceres: 368.
AT1G20190	0.006033067	0.358562817	0.010440407	0.415943047	ATEXPA11	expansin S2 precursor, putative similar to GB:U30460 from [Cucumis sativus];supported by full-length cDNA: Ceres:11011.
AT2G37130	0.002142909	0.358890577	0.045228474	0.588059948		putative peroxidase ATP2a ;supported by full-length cDNA: Ceres:3571.
AT2G47440	0.00127196	0.359315394	0.032103967	0.684343159		unknown protein similar to GP|2104534|AF001308 (T10M13.11)
AT4G17800	0.00141128	0.360059467	0.010031367	0.623162916		hypothetical protein
AT2G41310	0.026776718	0.361097207	0.02774886	0.508342691	ATRR3	putative two-component response regulator 3 protein identical to GB:AB010917, contains a response regulator receiver domain; supported by cDNA: gi_3273199_dbj_AB010917.1_AB010917
AT1G14340	0.001140098	0.361648481	0.008441845	0.481349303		hypothetical protein predicted by genemark.hmm;supported by full-length cDNA: Ceres:9495.
AT5G13840	0.001167618	0.361782851	0.04174164	0.516334767		cell cycle switch protein
AT5G62710	0.006018318	0.362360131	0.0114901	0.616966023		receptor – like protein kinase – like protein serine threonine kinase RLK1, Sorghum bicolor, EMBL:SBRLK1
AT2G30860	0.00053161	0.362895397	0.03267884	0.673570767	ATGSTF9	glutathione S-transferase identical to GB:Y12295; supported by cDNA: gi_13926309_gb_AF372905.1_AF372905
AT3G57040	0.019900344	0.363224665	0.005160912	0.28812477	ARR9	responce reactor 4 ; supported by cDNA: gi_3273201_dbj_AB010918.1_AB010918
AT3G18490	0.011292788	0.36457454	0.01609429	0.583334597		putative chloroplast nucleoid DNA-binding protein similar to GB:BAA22813 from [Nicotiana tabacum] (Plant Cell 9 (9), 1673-1682 (1997)), contains Pfam profile: PF00026 Eukaryotic aspartyl protease
AT2G19580	0.003033525	0.371022546	0.002792891	0.653946235	TET2	putative senescence-associated protein 5 contains a transmembrane 4 family signature; rare (GC) splice donor consensus found instead of (GT) at intron 2.
AT1G64640	0.001993154	0.371841292	0.003559839	0.398950393		unknown protein
AT4G36860	0.006513093	0.372295718	0.009211053	0.647625191		putative protein
AT2G46550	0.001912657	0.372743323	0.024533466	0.586007191		unknown protein ; supported by full-length cDNA: Ceres: 122665.
AT3G57230	0.000889151	0.375401205	0.037199095	0.692335178	AGL16	MADS-box transcription factor-like protein MADS-box transcription factor DEFH125 – Antirrhinum majus, PIR:T17029
AT5G25810	0.012407175	0.375995585	0.018036846	0.372260128	TNY	transcription factor TINY
AT3G18170	0.004121604	0.377246783	0.018542966	0.553621693		hypothetical protein
AT4G14130	0.001446053	0.377327023	0.037289992	0.653196023	XTR7	xyloglucan endotransglycosylase-related protein XTR-7 ;supported by full-length cDNA: Ceres:33554.
AT1G31940	0.009011808	0.377545539	0.03779119	0.573085082		unknown protein ;supported by full-length cDNA: Ceres:25047.
AT2G04240	0.010327619	0.379798849	0.01033256	0.514965622	XERICO	putative RING zinc finger protein ; supported by cDNA: gi_13265496_gb_AF324691.2_AF324691
AT3G45210	0.001562901	0.381006375	0.021321462	0.600479603		putative protein several hypothetical proteins – Arabidopsis thaliana; supported by cDNA: gi_15724293_gb_AF412087.1_AF412087
AT5G23860	0.00082477	0.382424533	0.006617936	0.575699803	TUB8	Expressed protein ; supported by cDNA: gi_15451225_gb_AY054693.1_
AT3G14190	0.007031506	0.382842632	0.015399912	0.445028817		hypothetical protein predicted by genscan+;supported by full-length cDNA: Ceres:112723.
AT5G54160	0.000734108	0.389051045	0.011136082	0.688835031	ATOMT1	O-methyltransferase ;supported by full-length cDNA: Ceres:33790.
AT4G00630	0.001599981	0.390870393	0.015589097	0.600789714	KEA2	putative potassium/H+ antiporter
AT4G10390	0.007874557	0.391215491	0.01934751	0.589647848		putative protein kinase CLV1 receptor kinase, Arabidopsis thaliana, gb:U96879
AT4G33150	0.012160913	0.393628493	0.011828709	0.55317971	LKR	lysine-ketoglutarate reductase/saccharopine ; supported by cDNA: gi_2052507
AT1G21440	0.013647696	0.394060065	0.04922577	0.566458529		hypothetical protein similar to carboxyvinyl-carboxyphosphonate phosphorylmutase GB:O49290 from [Arabidopsis thaliana]
AT3G27220	0.00186498	0.395043344	0.019492567	0.451094723		unknown protein ; supported by cDNA: gi_15081754_gb_AY048270.1_
AT1G43710	0.001075934	0.397462836	0.003568761	0.625605273	EMB1075	histidine decarboxylase, putative similar to histidine decarboxylase GB:BAA78331 GI:4996105 from [Brassica napus]; supported by cDNA: gi_15011301_gb_AF389349.1_AF389349
AT3G12950	0.016952898	0.398397962	0.019510278	0.62395947		unknown protein
AT1G12090	0.001585624	0.398535875	0.021153206	0.69613368	ELP	pEARLI 1-like protein may be induced when levels of Aluminum become toxic or other stresses become present in the plant;supported by full-length cDNA: Ceres:5712.
AT3G04940	0.02279394	0.398652205	0.020714253	0.593144929	ATCYSD1	putative cysteine synthase similar to cysteine synthase GB:BAA78562 [Arabidopsis thaliana];supported by full-length cDNA: Ceres:37566.
AT3G17998 /// AT3G18000	0.014835004	0.39947509	0.029733595	0.554424068	NMT1	unknown protein contains Pfam profile PF00398 Ribosomal RNA adenine dimethylases
AT3G17998 /// AT3G18000	0.014835004	0.39947509	0.020355968	0.530762969	NMT1	unknown protein contains Pfam profile PF00398 Ribosomal RNA adenine dimethylases
AT3G15680	0.021069938	0.401997073	0.010987509	0.665729098		putative zinc finger protein contains Pfam profile: PF00641 Zn-finger in Ran binding protein and others;supported by full-length cDNA: Ceres:8265.
AT4G37160	0.010702401	0.403243286	0.027508326	0.640686529	SKS15	pectinesterase like protein
AT1G12080	0.002072365	0.403759094	0.012853701	0.679736504		unknown protein ; supported by full-length cDNA: Ceres: 270281.
AT5G58350	0.008831373	0.40388802	0.021916801	0.690931394	WNK4	MAP kinase ; supported by cDNA: gi_14532571_gb_AY039910.1_
AT4G14465	0.002252138	0.404259603	0.013341632	0.64614251		Expressed protein ; supported by full-length cDNA: Ceres: 36844.
AT1G08280	0.003552384	0.405286985	0.007051962	0.632444144		hypothetical protein contains similarity to alpha2,8-sialyltransferase GI:929684 from [Mus musculus]
AT1G23870	0.008546504	0.406205124	0.023611085	0.367970954	ATTPS9	trehalose 6-phosphate synthase, putative similar to trehalose 6-phosphate synthase GB:CAA09463 GI:3647365 from [Yarrowia lipolytica]
AT1G75220	0.00082477	0.406472935	0.02058046	0.663869607		integral membrane protein, putative similar to GI:1209755 from [Beta vulgaris] (Plant Physiol. 110 110 (2), 511-520 (1996)); supported by cDNA: gi_15724239_gb_AF412060.1_AF412060
AT2G17710	0.005341495	0.406894353	0.022441871	0.482587984		unknown protein ;supported by full-length cDNA: Ceres:1011.
AT2G47930	0.01163845	0.408209901	0.020803593	0.680927003	AGP26	unknown protein ; supported by cDNA: gi_14326466_gb_AF385686.1_AF385686
AT3G21220	0.001957762	0.408308807	0.01978298	0.693945719	ATMKK5	MAP kinase kinase 5 identical to GB:BAA28831 from [Arabidopsis thaliana]; supported by cDNA: gi_3219272_dbj_AB015316.1_AB015316
AT5G25460	0.003612749	0.408875643	0.004274498	0.525606774		putative protein hypothetical protein – Ricinus communis, EMBL:Z81012;supported by full-length cDNA: Ceres:1351.
AT5G35750	0.001325433	0.412119036	0.010166872	0.58008769	AHK2	histidine kinase-like protein ; supported by cDNA: gi_13537195_dbj_AB046869.1_AB046869
AT3G10740	0.017375616	0.414660969	0.024295934	0.53924728	ASD1	putative alpha-L-arabinofuranosidase contains similarity to alpha-L-arabinofuranosidase A precursor GB:P42254 [Aspergillus niger]; supported by cDNA: gi_13937190_gb_AF372949.1_AF372949
AT2G04795	0.010219285	0.415699162	0.010111201	0.523648873		Expressed protein ; supported by cDNA: gi_13926282_gb_AF372895.1_AF372895
AT1G08560	0.004667494	0.416723688	0.013251605	0.555538118	SYP111	putative syntaxin-related protein (U39451) BAC F22O13 has a deletion of a cytosine at position 7887; supported by cDNA: gi_1184164_gb_U39451.1_ATU39451
AT1G14210	0.006546611	0.418362429	0.021321462	0.642959052		ribonuclease contains similarity to RNase GI:7768564 from [Nicotiana tabacum]
AT2G13820	0.000847379	0.418435078	0.009211053	0.654995606		putative nonspecific lipid-transfer protein precursor ; supported by full-length cDNA: Ceres: 24286.
AT3G03130	0.00093031	0.421019624	0.04199731	0.525456033		hypothetical protein predicted by genefinder
AT1G74660	0.008415733	0.421383422	0.039842952	0.571740317	MIF1	hypothetical protein predicted by genefinder;supported by full-length cDNA: Ceres:14583.
AT3G01120	0.001101445	0.421638683	0.010399547	0.689243148	MTO1	putative cystathionine gamma-synthase similar to cystathionine gamma-synthase GB:AAB41235 from [Arabidopsis thaliana]; supported by cDNA: gi_1389725_gb_U43709.1_ATU43709
AT5G63780	0.001632983	0.421990997	0.020342216	0.606703565	SHA1	putative protein similar to unknown protein (gb|AAD32776.1)
AT4G35090	0.003801287	0.42377559	0.020383343	0.639488655	CAT2	catalase ; supported by cDNA: gi_15451165_gb_AY054663.1_
AT4G23800	0.002899901	0.424324684	0.006060074	0.563251384		98b like protein 98b – Daucus carota (carrot),PID:d1033793; supported by cDNA: gi_11908077_gb_AF326886.1_AF326886
AT1G70290	0.0382701	0.425706364	0.013753682	0.295113342	ATTPS8	trehalose-6-phosphate synthase, putative similar to trehalose-6-phosphate synthase GI:297792 from [Kluyveromyces lactis]
AT4G32290	0.001105544	0.427787795	0.00745668	0.457237963		putative protein predicted protein, Arabidopsis thaliana, PATCHX:G2252634
AT2G45430	0.001805252	0.428061888	0.022718003	0.613345214		putative AT-hook DNA-binding protein highly similar to hypothetical protein gi2245139:gnl:PID:e327087:Z97344
AT4G12880	0.003310761	0.428916007	0.009669296	0.646508489		blue copper-binding protein, 15K (lamin) ;supported by full-length cDNA: Ceres:41510.
AT1G11545	0.001618492	0.429055317	0.003083379	0.563752644		endo-xyloglucan transferase, putative similar to endo-xyloglucan transferase GI:2244732 from [Gossypium hirsutum];supported by full-length cDNA: Ceres:7729.
AT5G27950	0.001487294	0.430851278	0.019585207	0.654853674		putative protein kinesin heavy chain-like protein, potato, PIR:T07397
AT1G24100	0.001864683	0.431279066	0.014052022	0.610273565	UGT74B1	putative indole-3-acetate beta-glucosyltransferase similar to GB:AAB64022; supported by cDNA: gi_14423541_gb_AF387008.1_AF387008
AT3G07130	0.011237277	0.433798414	0.040544126	0.558174604	ATPAP15	putative purple acid phosphatase similar to purple acid phosphatase GB:CAA06921 [Ipomoea batatas]
AT5G43390 /// AT5G43400	0.002297393	0.43397112	0.025188968	0.566672505		putative protein strong similarity to unknown protein (emb|CAB86628.1)
AT5G22830	0.007461631	0.435458592	0.049258627	0.685560391	GMN10	putative protein similar to unknown protein (ref|NP_015265.1); supported by cDNA: gi_12007446_gb_AF322255.1_AF322255
AT5G41670	0.008355174	0.435771564	0.008673582	0.443385502		6-phosphogluconate dehydrogenase ;supported by full-length cDNA: Ceres:109329.
AT3G01690	0.017544674	0.436956372	0.003559839	0.467492511		unknown protein similar to putative protein GB:CAA22987 [Arabidopsis thaliana]
AT5G18680	0.001999927	0.438487869	0.034820847	0.620627871	AtTLP11	tub family-like protein tubby-like protein – Lemna paucicostata, EMBL:AB023895
AT3G10260	0.002545516	0.438772404	0.044967987	0.622736043		unknown protein similar to unknown protein GB:AAC62889 [Arabidopsis thaliana];supported by full-length cDNA: Ceres:15153.
AT5G50400	0.016303474	0.438827723	0.007876929	0.525173399	ATPAP27	putative protein contains similarity to unknown protein (pir||T05510)
AT5G13390	0.003774856	0.439481526	0.009533367	0.576573053	NEF1	putative protein
AT5G04470	0.007019318	0.440623281	0.043203797	0.661855156	SIM	putative protein ;supported by full-length cDNA: Ceres:13812.
AT2G36090	0.010379666	0.440690214	0.005089877	0.483840455		hypothetical protein predicted by genscan and genefinder
AT2G41490	0.008264208	0.441358232	0.02058046	0.504351545	GPT	putative UDP-N-acetylglucosamine--dolichyl-phosphate N-acetylglucosaminephosphotransferase ;supported by full-length cDNA: Ceres:111048.
AT1G47330	0.000712948	0.441481807	0.024862919	0.693579012		unknown protein similar to hypothetical protein GB:CAA20583 GI:3549672 from [Arabidopsis thaliana]
AT5G65650	0.001170891	0.441589441	0.017681338	0.596931177		putative protein similar to unknown protein (emb|CAB16809.1); supported by full-length cDNA: Ceres: 118150.
AT5G03150	0.001779904	0.445044067	0.014650066	0.674887107	JKD	putative protein zinc finger protein, Arabidopsis thaliana, EMBL:AL132953
AT3G04910	0.002624963	0.44515289	0.01609429	0.681356903	WNK1	putative mitogen activated protein kinase kinase similar to mitogen activated protein kinase kinase GB:AAC32599 [Oryza sativa]
AT5G59090	0.001440628	0.448688036	0.005784473	0.609705834	ATSBT4.12	subtilisin-like serine protease contains similarity to prepro-cucumisin GI:807698 from [Cucumis melo]
AT5G19010	0.007255682	0.448834404	0.032664787	0.654071731	MPK16	MAP kinase -like protein ATMPK9, Arabidopsis thaliana, EMBL:AB038694
AT5G49270	0.000910835	0.451546508	0.02774886	0.569308272	COBL9	putative protein contains similarity to phytochelatin synthetase
AT3G01290	0.015577642	0.452351304	0.013786156	0.68470757		unknown protein similar to hypothetical protein GB:CAA10289 [Cicer arietinum]; supported by full-length cDNA: Ceres: 108086.
AT5G67420	0.013899138	0.455735172	0.012842812	0.611146904	LBD37	putative protein similar to unknown protein (emb|CAB62102.1);supported by full-length cDNA: Ceres:40250.
AT1G08650	0.02628545	0.458861822	0.031097824	0.334179632	PPCK1	putative calcium-dependent protein kinase (U90439) similar to ESTs gb|T46119, gb|H76837, and gb|H36948; supported by cDNA: gi_6318612_gb_AF162660.1_AF162660
AT5G40450	0.007686173	0.4598413	0.018844021	0.690534199		putative protein microtubule-associated protein homolog, Drosophila melanogaster, EMBL:T13564
AT1G04020	0.003693871	0.460181697	0.010217648	0.639109051	ATBARD1	unknown protein
AT1G70230	0.002741015	0.464522058	0.014419551	0.602228353		unknown protein
AT4G33960	0.018339604	0.464846149	0.030307932	0.531930823		putative protein
AT4G36640	0.003928899	0.465121363	0.01851467	0.651895369		putative protein
AT2G28200	0.016869335	0.466614981	0.045694776	0.617352545		putative zinc-finger protein similar to zinc-finger protein GB:AAC98446
AT2G28350	0.001490481	0.46849917	0.033453763	0.672602066	ARF10	unknown protein
AT4G01330	0.031764556	0.469099699	0.025188968	0.517709871		putative protein kinase
AT1G10470	0.02543755	0.470929942	0.012741539	0.596929074	ARR4	putative response regulator 3 similar to ESTs gb|T43772, emb|Z34204, gb|AA067391, and emb|Z34620, implicated in His-to-Asp phosphotransfer signaling in Arabidopsis; supported by cDNA: gi_3273195_dbj_AB010915.1_AB010915
AT4G36180	0.008389374	0.474881161	0.033650305	0.684223851		putative receptor protein kinase Cf-2.1 leucine rich repeat protein, Solanum pimpinellifolium, PATX:G1184075
AT5G61790	0.001446053	0.475336161	0.006819067	0.521090187		calnexin – like protein calnexin homolog, Arabidopsis thaliana, EMBL:AT08315;supported by full-length cDNA: Ceres:2869.
AT4G22150	0.011352806	0.476056682	0.00401479	0.626742697	PUX3	putative protein p47 – Homo sapiens,PID:g5531827
AT1G80920	0.032373402	0.476292766	0.007426498	0.419829487	J8	J8-like protein similar to DnaJ homologue J8 GB:AAC72399 GI:3851670 from [Arabidopsis thaliana];supported by full-length cDNA: Ceres:4150.
AT2G04780	0.002242509	0.477045364	0.009533367	0.693341502	FLA7	unknown protein ;supported by full-length cDNA: Ceres:11114.
AT5G56320	0.005094098	0.477653004	0.010334739	0.538362935	ATEXPA14	expansin ;supported by full-length cDNA: Ceres:42551.
AT5G12860	0.010100544	0.478766628	0.009211053	0.519612137	DIT1	2-oxoglutarate/malate translocator precursor -like protein 2-oxoglutarate/malate translocator precursor, spinach, SWISSPROT:SOT1_SPIOL; supported by cDNA: gi_13877586_gb_AF370494.1_AF370494
AT3G45240 /// AT3G45242	0.0214922	0.478822931	0.025831759	0.583845872	CPuORF61	serine threonine-protein kinase-like protein Ca2+ calmodulin-dependent protein kinase – Rattus norvegicus, PIR:A57156; supported by cDNA: gi_14334793_gb_AY035070.1_
AT1G17430	0.001200662	0.480316394	0.012977538	0.535278722		unknown protein ;supported by full-length cDNA: Ceres:32887.
AT1G16170	0.004166145	0.481259444	0.028061083	0.571446172		unknown protein ; supported by full-length cDNA: Ceres: 32430.
AT1G19920	0.002256306	0.485231636	0.006460856	0.469702829	APS2	sulfate adenylyltransferase identical to sulfate adenylyltransferase GI:487404 from [Arabidopsis thaliana];supported by full-length cDNA: Ceres:21320.
AT5G62550	0.005527436	0.485797822	0.002933816	0.45043012		unknown protein
AT4G18800	0.003115624	0.486517245	0.018365737	0.67390222	AtRABA1d	ras-like GTP-binding protein strong homology to GTP-binding protein ric2, Oryza sativa, S38741
AT1G80310	0.012415712	0.487361973	0.003654565	0.638854437		putative sulfate transporter contains Pfam profile: PF00916 sulfate transporter family;supported by full-length cDNA: Ceres:120095.
AT3G27060	0.003552384	0.48878617	0.01295069	0.475631428	TSO2	ribonucleotide reductase small subunit, putative similar to RIBONUCLEOSIDE-DIPHOSPHATE REDUCTASE SMALL CHAIN GB:P49730 from [Nicotiana tabacum]
AT5G18170	0.001853667	0.48962998	0.024049086	0.658087444	GDH1	glutamate dehydrogenase (EC 1.4.1.-) 1 (pir||S71217) ; supported by cDNA: gi_15146203_gb_AY049243.1_
AT1G07730 /// AT1G07740	0.005637503	0.490291012	0.014678935	0.554856652		disease resistance response protein, putative similar to GB:AAA33662 from [Pisum sativum] (Plant Mol. Biol. 11, 713-715 (1988))
AT5G15770	0.005115179	0.491318211	0.012347897	0.530991646	ATGNA1	acetyltransferase-like protein Glucosamine-6-phosphate acetyltransferase EMeg32 protein – Mus musculus, EMBL:AJ001006
AT2G32300	0.004661717	0.493571673	0.010260057	0.553379483	UCC1	putative uclacyanin I identical to GP:U76298; member of blue copper protein family (Phytocyanins); contains a type-1 copper (blue) protein signature (PDOC00174); supported by cDNA: gi_3399766_gb_U76298.1_ATU76298
AT5G44130	0.017636377	0.493655734	0.030577654	0.613167928	FLA13	putative protein contains similarity to surface protein; supported by cDNA: gi_16648846_gb_AY058201.1_
AT1G21920	0.028607752	0.494574028	0.024485148	0.580643281		phosphatidylinositol-4-phosphate 5-kinase, putative similar to phosphatidylinositol-4-phosphate 5-kinase GB:CAB53377 GI:5777366 from [Arabidopsis thaliana];supported by full-length cDNA: Ceres:37462.
AT1G73620	0.001988965	0.494678105	0.009622522	0.400487538		thaumatin-like protein contains Pfam profile: PF00314 thaumatin family; supported by full-length cDNA: Ceres: 17109.
AT4G28240	0.007555784	0.497036371	0.027410286	0.449473857		putative wound induced protein wound-induced protein – tomato (fragment), PIR2:S19773;supported by full-length cDNA: Ceres:20161.
AT5G61000	0.008832886	0.497603566	0.017179599	0.667533481		RNA helicase
AT1G31230	0.000910835	0.497641528	0.005785137	0.526108724	AK-HSDH	putative aspartate kinase-homoserine dehydrogenase almost identical to gb|X71364 gene for aspartate kinase homoserine dehydrogenase from Arabidopsis thaliana; contains ACT domain
AT1G27330 /// AT1G27350	0.003458546	0.498690538	0.03821641	0.682445815		unknown protein similar to EST gb|AA650671 and gb|T20610
AT1G20010	0.004397515	0.499231433	0.025975568	0.628250805	TUB5	beta tubulin 1, putative similar to GB:AAD02498 from [Arabidopsis thaliana] (Plant Mol. Biol. 39 (1), 171-176 (1999)); supported by cDNA: gi_13605518_gb_AF361585.1_AF361585
AT4G01220	0.007420145	0.499966552	0.03830221	0.676745849		hypothetical protein ; supported by cDNA: gi_13877592_gb_AF370497.1_AF370497
AT4G37490	0.001369848	0.540692928	0.004800609	0.328209728	CYC1	cyclin cyc1 ; supported by cDNA: gi_166683_gb_M80190.1_ATHCYCLIN
AT1G73640	0.015485592	0.509694127	0.0068489	0.355726722	AtRABA6a	putative ras-related GTP-binding protein contains Pfam profile: PF00071 ras family;supported by full-length cDNA: Ceres:118351.
AT5G17160	0.008013723	0.591254588	0.017243126	0.404342983		putative protein (fragment) predicted protein, Arabidopsis thaliana
AT4G29520	0.010031772	0.564090114	0.016117405	0.417338582		putative protein ;supported by full-length cDNA: Ceres:142647.
AT3G02020 /// AT5G14060	0.012094137	0.643053361	0.003341037	0.419455944		putative aspartate kinase similar to aspartate kinase GB:CAA67376 (Arabidopsis thaliana); supported by full-length cDNA: Ceres: 6203.
AT5G60680	0.014319406	0.557258693	0.029654969	0.427117192		putative protein predicted proteins, Arabidopsis thaliana;supported by full-length cDNA: Ceres:16638.
AT2G37750	0.014439714	0.501325681	0.020383343	0.428997245		unknown protein ; supported by full-length cDNA: Ceres: 28529.
AT2G17630	0.00585184	0.544267152	0.003176803	0.439531962		putative phosphoserine aminotransferase
AT4G33730	0.00255286	0.557308166	0.03789236	0.444320291		pathogenesis-related protein – like pathogenesis-related protein 1 precursor, 17.6K, Arabidopsis thaliana, PIR2:JQ1693
AT4G39940	0.026470821	0.598182435	0.02677608	0.452195743	AKN2	adenosine-5-phosphosulfate-kinase
AT5G07340	0.020718286	0.586292631	0.015093043	0.455509976		calnexin homolog
AT3G54960	0.011808138	0.561731197	0.006194339	0.456477516	ATPDIL1-3	protein disulfide-isomerase-like protein protein disulphide isomerase, Fasciola hepatica
AT3G62600	0.006057839	0.62700532	0.007026021	0.456794607		putative protein HIRA interacting protein 4 (dnaJ-like) – Homo sapiens, EMBL:AJ001309;supported by full-length cDNA: Ceres:37292.
AT1G30760	0.002846189	0.560751542	0.03851855	0.465069988		putative reticuline oxidase-like protein similar to GB:P30986 from [Eschscholzia californica] (berberine bridge-forming enzyme), ESTs gb|F19886, gb|Z30784 and gb|Z30785 come from this gene
AT1G07410	0.030516323	0.621109602	0.013295718	0.46736677	AtRABA2b	small G protein, putative similar to GB:Z49190 from [Beta vulgaris]
AT2G25880	0.030590666	0.599193593	0.030511038	0.468520637	ATAUR2	putative protein kinase contains a protein kinase domain profile (PDOC00100)
AT1G03920	0.006497784	0.630094154	0.009727794	0.47793037		putative protein kinase highly similar to Mesembryanthemum crystallinum protein kinase, gi|457689 and Nicotiana tabacum protein kinase gi|506534;supported by full-length cDNA: Ceres:110932.
AT5G05470	0.034997564	0.688032696	0.003779925	0.497676349	EIF2 ALPHA	eukaryotic translation initiation factor 2 alpha subunit-like protein ; supported by cDNA: gi_13358180_gb_AF324988.2_AF324988

Supplementary Table S1 As-responsive genes in *Arabidopsis* Col-0 and Ws-2 ecotypes (Ws100 Specifically Down)

AGI	Col-200 q-value	Regulation	Col-200 FC	Ws-100 q-value	Regulation	Ws-100 FC	Gene Symbol	Target Description
AT4G18340	0.38355526	down	0.802184766	0.017679557	down	0.279564295		beta-1,3-glucanase-like protein strong simlarity to endo-beta-1,3-beta-D-glucosidase, Nicotiana tabacum, PIR2:S46495
AT3G07350	0.36855793	down	0.757967259	0.013753682	down	0.288840581		unknown protein similar to hypothetical protein GB:AAC17612 [Arabidopsis thaliana];supported by full-length cDNA: Ceres:251012.
AT5G60760	0.16752282	up	1.5431166	0.049351502	down	0.320925642		putative protein various predicted proteins, Arabidopsis thaliana
AT5G57540	0.20291628	up	1.1746027	0.00594099	down	0.346923121		xyloglucan endotransglycosylase
AT4G25170	0.36855793	down	0.81462254	0.021623662	down	0.393522711		putative protein ;supported by full-length cDNA: Ceres:38603.
AT5G28610	0.10444952	up	1.3896573	0.030838741	down	0.397794057		putative protein
AT1G19900	0.3710031	down	0.709023393	0.01912762	down	0.403194413		unknown protein
AT5G22410	0.6096551	up	1.0393777	0.01031717	down	0.404727705		peroxidase ATP14a homolog
AT2G32150	0.81958014	down	0.93468042	0.021308355	down	0.416815852		putative hydrolase ; supported by cDNA: gi_15982855_gb_AY057535.1_
AT1G14360	0.24099225	up	1.1309868	0.006885885	down	0.425770636	ATUTR3	unknown protein ;supported by full-length cDNA: Ceres:6937.
AT5G37260	0.13735597	up	1.4591568	0.02182219	down	0.434250633	CIR1	putative protein DNA-binding protein CCA1, Arabidopsis thaliana, PIR:T02684
AT3G47960	0.0609582	up	1.4615151	0.04172557	down	0.436758714		putative peptide transporter peptide transporter (ptr1), Hordeum vulgare, AF023472; supported by cDNA: gi_13877878_gb_AF370202.1_AF370202
AT5G47100	0.2635496	down	0.873202524	0.002808379	down	0.46129089	CBL9	calcium sensor protein, calcineurin-like ; supported by cDNA: gi_15866278_gb_AF411958.1_AF411958
AT1G53680	0.20592178	down	0.782496921	0.015866674	down	0.4713706	ATGSTU28	glutathione transferase, putative similar to GI:2853219 from [Carica papaya]
AT4G05070	0.110611774	up	1.4186966	0.01243731	down	0.488575397		coded for by A. thaliana cDNA T44741

Supplementary Table S1 As-responsive genes in *Arabidopsis* Col-0 and Ws-2 ecotypes (Ws100 Specifically Up)								
AGI	Col-200 q-value	Regulation	Col-200 FC	Ws-100 q-value	Regulation	Ws-100 FC	Gene Symbol	Target Description
AT5G10180	0.20467189	down	0.344256832	0.023272073	up	9.6482525	AST68	sulfate transporter ; supported by cDNA: gi_2114105_dbj_AB003591.1_AB003591
AT3G09410	0.001886784	down	0.541825223	0.005151427	up	8.84384		putative pectinacetylesterase similar to pectinacetylesterase precursor GB:CAA67728 [Vigna radiata]
AT1G64590	0.085716315	down	0.512179684	0.002890685	up	8.418304		oxidoreductase, putative similar to PROTOCHLOROPHYLLIDE REDUCTASE GB:P21218 from [Arabidopsis thaliana]; supported by cDNA: gi_15081623_gb_AY048204.1_
AT4G36610	0.5188534	down	0.666072574	0.004857321	up	5.164671		putative protein ; supported by full-length cDNA: Ceres: 38141.
AT5G23190	0.5402539	down	0.614150758	0.012163703	up	5.120093	CYP86B1	cytochrome P450-like protein
AT1G43910	0.020795824	up	1.345091	0.002145344	up	4.9416904		unknown protein
AT5G13580	0.0973251	down	0.298833611	0.008618869	up	4.0263023		ABC transporter-like protein
AT1G68850	0.68582153	down	0.788705361	0.018554265	up	3.8923454		peroxidase ATP23a identical to peroxidase ATP23a GB:CAA70035 (Arabidopsis thaliana)
AT5G12420	0.11532025	down	0.356268066	0.02018253	up	3.7775645		putative protein similarity to various predicted proteins~Contains ATP synthase delta (OSCP) subunit signature AA211-230;Prokaryotic membrane lipoprotein lipid attachment site AA140-150
AT3G47780	0.47565055	up	1.2605184	0.003341037	up	3.6964529	ATATH6	ABC-type transport protein-like protein transport protein ABC-C, Homo sapiens, PIR2:S71363
AT2G17500	0.17823245	up	1.3213862	0.005592544	up	3.5372214		unknown protein ;supported by full-length cDNA: Ceres:105072.
AT5G36140	0.011543379	down	0.07888054	0.04883703	up	3.5131638	CYP716A2	putative protein contains similarity to cytochrome P450
AT5G09520	0.021792412	down	0.062388052	0.008154522	up	3.5036473		putative proline-rich protein surface protein C PspC, Streptococcus pneumoniae, EMBL:AF145055
AT1G14130	0.7232593	up	1.0863825	0.0025088	up	3.3540387		dioxygenase-like protein very similar to adventitious rooting related oxygenase; Very similar to 2-oxoacid dependent dioxygenase from Malus domestica, gi|3492806
AT4G24130	0.6862039	up	1.1722269	0.001810002	up	3.1835911		putative protein predicted proteins
AT3G56980	0.033487525	down	0.516859117	0.002854609	up	3.0740807	ORG3	putative protein hypothetical protein At2g41240 - Arabidopsis thaliana, EMBL:AC005662
AT1G04220	0.09262513	down	0.321872049	0.004227638	up	3.0387132		putative beta-ketoacyl-CoA synthase Strong similarity to beta-keto-Coa synthase gb|U37088 from Simmondsia chinensis
AT3G22620	0.22772369	down	0.707481411	0.002808379	up	2.9819489		hypothetical protein predicted by genemark.hmm; supported by cDNA: gi_14334663_gb_AY035005.1_
AT1G68880	0.025569398	down	0.48848399	0.009533367	up	2.9652605	ATBZIP	putative bZIP transcription factor similar to bZIP DNA-binding protein GB:AAD55394 [Lycopersicon esculentum]; contains Pfam profile: PF00170 bZIP transcription factor; supported by cDNA: gi_15278043_gb_AF400621.1_AF400621
AT5G26340	0.898253	up	1.0296892	0.005459929	up	2.9377475	MSS1	hexose transporter - like protein hexose transporter HT2, Lycopersicon esculentum, EMBL:LES132224; supported by cDNA: gi_15010579_gb_AY045591.1_
AT5G62480	0.6913417	down	0.917049565	0.002933816	up	2.9362693	ATGSTU9	glutathione S-transferase-like protein ; supported by cDNA: gi_11095991_gb_AF288179.1_AF288179
AT5G52810	0.013057182	down	0.533829154	0.00935342	up	2.8786209		putative protein contains similarity to ornithine cyclodeaminase;supported by full-length cDNA: Ceres:114678.
AT3G50400	0.021764142	down	0.076825662	0.015037157	up	2.7990916		putative protein RXF26, Arabidopsis thaliana, EMBL:AB008020
AT3G10500	0.52964795	down	0.872029269	0.0025088	up	2.7816527	ANAC053	unknown protein N-terminus similar to unknown protein GB:AAD25613 [Arabidopsis thaliana]; supported by full-length cDNA: Ceres: 38529.
AT1G01610	0.010323489	down	0.110348359	0.001810002	up	2.7479565	ATGPAT4	hypothetical protein similar to unknown protein GI:3335359 from [Arabidopsis thaliana]
AT3G26470	0.5163396	up	1.0962435	0.019258955	up	2.5943437		unknown protein ;supported by full-length cDNA: Ceres:4836.
AT5G41040	0.009140977	down	0.242722629	0.00869683	up	2.482972		N-hydroxycinnamoyl/benzoyltransferase-like protein ; supported by cDNA: gi_14334525_gb_AY034954.1_
AT1G64170	0.027494289	down	0.270789088	0.007676224	up	2.4809413	ATCHX16	hypothetical protein similar to hypothetical protein GB:AAF24561 GI:6692096 from [Arabidopsis thaliana]
AT5G38790	0.16707535	up	1.2279953	0.007175001	up	2.4777346		putative protein predicted protein, Arabidopsis thaliana
AT3G01420	0.09588655	down	0.443461124	0.01033256	up	2.4722967	ALPHA-DOX1	feebly-like protein contains similarity to feebly protein GB:S70648 [Lycopersicon esculentum]; supported by cDNA: gi_14595998_gb_AY042787.1_
AT5G66985	0.8002216	down	0.9275925	0.009820315	up	2.404206		Expressed protein ; supported by full-length cDNA: Ceres: 4410.
AT2G16760	0.91938937	down	0.977955418	0.006206627	up	2.375681		hypothetical protein predicted by genefinder;supported by full-length cDNA: Ceres:118464.
AT2G48140	0.035528384	down	0.321757651	0.04023467	up	2.356584	EDA4	unknown protein ;supported by full-length cDNA: Ceres:4074.
AT4G23700	0.21370786	down	0.407867037	0.01502979	up	2.3530939	ATCHX17	putative Na+/H+-exchanging protein Na+/H+-exchanging protein slr1595 - Synechocystis sp., EMBL:D90902
AT5G27280	0.058677036	down	0.749953334	0.006778613	up	2.3308218		putative protein predicted proteins, Arabidopsis thaliana, Drosophila melanogaster and Saccharomyces cerevisiae;supported by full-length cDNA: Ceres:23239.
AT4G14440	0.014129692	down	0.117731521	0.011962639	up	2.3215673		carnitine racemase like protein
AT4G34138	0.06574477	down	0.534273769	0.021623662	up	2.3187842	UGT73B1	predicted protein
AT4G34120	0.173979	up	1.2718893	0.003083379	up	2.301853	LEJ1	putative protein hypothetical protein, Arabidopsis thaliana, PATCHX:E353194; supported by cDNA: gi_13430837_gb_AF360331.1_AF360331
AT2G48130	0.01815654	down	0.334433978	0.007268087	up	2.2741556		unknown protein ;supported by full-length cDNA: Ceres:32411.
AT4G17280	0.004605718	down	0.205091015	0.006399318	up	2.2692916		hypothetical protein
AT5G43760	0.07393983	down	0.778416745	0.010272142	up	2.2184749		beta-ketoacyl-CoA synthase ; supported by cDNA: gi_14334713_gb_AY035030.1_
AT3G57520	0.025908923	down	0.51031833	0.012631322	up	2.1786199	ATSIP2	imbibition protein homolog probable imbibition protein - Brassica oleracea, PIR:S45033; supported by cDNA: gi_15292676_gb_AY050772.1_
AT1G74460	0.00996064	down	0.063743155	0.04199731	up	2.1712277		putative lipase/acylhydrolase contains Pfam profile: PF00657 lipase/acylhydrolase with GDSL-like motif; similar to anther-specific pro-rich protein APG GB:P40603 [Brassica napus] (Plant J 1993 Jan;3(1):111-20);supported by full-length cDNA: Ceres:30001.
AT4G22070	0.48250777	down	0.684866445	0.039433565	up	2.1640954	WRKY31	putative protein DNA-binding protein ABF2, wild oat, PIR2:S61414; supported by cDNA: gi_15990589_gb_AY052648.1_
AT5G09530	0.028002966	down	0.122265382	0.043509297	up	2.1629276		periaxin - like protein periaxin, Rattus norvegicus, PIR:I58157; supported by cDNA: gi_15451135_gb_AY054648.1_
AT5G18270	0.2892227	up	1.1813635	0.019014608	up	2.1567152	ANAC087	NAM (no apical meristem)-like protein
AT3G58810	0.008764003	down	0.533484274	0.020342216	up	2.1444857	MTPA2	zinc transporter -like protein zinc transporter ZAT, Arabidopsis thaliana, EMBL:AF072858
AT1G23440	0.14771868	up	1.2558101	0.02314834	up	2.134707		hypothetical protein similar to hypothetical protein GB:AAF27077 GI:6730656 from [Arabidopsis thaliana]
AT1G23440	0.89893854	up	1.0191531	0.02314834	up	2.134707		hypothetical protein similar to hypothetical protein GB:AAF27077 GI:6730656 from [Arabidopsis thaliana]
AT3G22600	0.4939121	down	0.851513139	0.00810806	up	2.1177402		unknown protein contains Pfam profile:PF00279 LTP:Plant lipid transfer protein family;supported by full-length cDNA: Ceres:19287.
AT5G17010	0.004667494	up	1.3937246	0.008010069	up	2.0801177		sugar transporter - like protein D-XYLOSE-PROTON SYMPORTER (D-XYLOSE TRANSPORTER), Lactobacillus brevis, SWISSPROT:XYLT_LACBR
AT2G43400	0.36219597	up	1.175329	0.007993701	up	2.0781512	ETFQO	putative electron transfer flavoprotein ubiquinone oxidoreductase
AT4G14010	0.13594078	up	1.1259611	0.001992044	up	2.0693905	RALFL32	hypothetical protein ;supported by full-length cDNA: Ceres:7346.
AT3G26210	0.002939021	down	0.274180845	0.02520343	up	2.0633397	CYP71B23	cytochrome P450, putative contains Pfam profile: PF00067 cytochrome P450
AT2G39310	0.123917624	down	0.627277803	0.015197328	up	2.0610676		putative myrosinase-binding protein ;supported by full-length cDNA: Ceres:33545.
AT4G34710	0.7640269	up	1.0684768	0.016682763	up	2.0593789	ADC2	arginine decarboxylase SPE2 ; supported by cDNA: gi_14517491_gb_AY039581.1_
AT5G67480	0.19770062	up	1.3080083	0.013106542	up	2.058354	BT4	putative protein strong similarity to unknown protein (pir||T04718); supported by cDNA: gi_15529177_gb_AY052213.1_
AT5G53420	0.116177924	up	1.2246394	0.017795974	up	2.0249283		putative protein similar to unknown protein (pir||T02891);supported by full-length cDNA: Ceres:112574.
AT5G13550	0.09156216	up	1.3937199	0.006914928	up	2.020414	SULTR4;1	sulfate transporter ; supported by cDNA: gi_2626752_dbj_AB008782.1_AB008782

Supplementary Table S2 Comparative expression of genes regulated in 2 *Arabidopsis* ecotypes with As stress

Functional categories[a]	In genome	On array	Ws-2 100 μM As vs Ws-2 Control			Col-0 200 μM As vs Col-0 Control		
			Detected	Induced	Repressed	Detected	Induced	Repressed
Oxidative stress-related genes								
Alternative oxidase	6	6	2	1	0	2	1	0
Dehydroascorbate reductase	5	5	2	1	0	2	1	0
Glutaredoxin	27	25	8	2	1	8	1	0
Thioredoxins	32	30	22	1	0	22	4	0
Class III peroxidase	73	72	36	1	3	39	4	14
Glutathion-S-transferase	53	52	33	14	2	35	9	6
Glutathion-S-transferase -Tau family	28	28	21	12	0	22	7	3
Transporter-related genes								
ABC transporters	124	117	41	8	1	41	10	4
MATE	57	56	19	4	0	19	4	4
Antiporters	79	75	24	3	1	26	5	6
Aquaporin	40	40	16	0	2	16	0	9
LeOPT1	52	51	17	1	6	19	1	12
Sugar transporter	53	52	20	2	1	22	0	7
Phytohormone-related genes								
CK biosynthesis	11	11	2	0	1	3	0	3
CK receptor	5	5	4	0	1	4	0	3
CK response down	11	10	5	1	0	5	0	3
CK response up	62	61	31	1	9	37	8	16
CK signaling	21	19	17	0	1	17	0	2
CK transport	20	14	3	0	1	3	0	2
ABA receptor	3	3	1	0	0	1	0	0
ABA biosynthesis	13	13	4	0	0	5	1	0
ABA signaling	33	31	20	0	0	18	4	3
Negative regulation of ABA signaling	12	12	9	0	0	10	3	1
ET receptor	5	5	0	0	0	5	1	0
ET biosynthesis	26	20	8	0	0	11	3	0
ET signaling	66	63	17	3	2	30	8	2
Transcription factor-related genes								
AP2/EREBP	146	131	46	7	5	50	17	9
Aux/IAA	29	28	14	0	0	15	4	2
bZIP	72	66	38	5	2	38	8	7
HSF	23	23	7	3	0	9	7	0
NAC	107	95	28	8	1	28	9	3
WRKY	72	62	26	6	0	27	10	3
bHLH	127	89	34	1	1	34	1	7
C2H2	134	107	43	3	2	45	8	11
GARP-G2-like	43	40	16	0	0	17	0	6
MYB	150	142	48	2	4	50	6	13
Protein kinase-related genes								
MAPK	24	23	13	0	0	14	1	2
MAPKK	10	10	4	0	0	4	0	1
Raf (MAPKKK)	48	48	24	0	0	29	2	1
ZIK (MAPKKK)	10	10	3	0	0	4	0	3
Protein tyrosine phosphatase	19	18	9	1	0	11	2	1
Calcium-dependent protein kinase	32	34	16	0	0	16	2	1
SnRK	38	37	24	0	2	24	3	5
LRR-RLK VIII	22	19	8	1	0	9	4	0

[a]Functional categories of genes, total number of genes found within the *Arabidopsis* genome, numbers of genes present on and detected on array, and numbers of genes showing induction or repression (>2-fold change, 5% <FDR) in transcript abundance are shown in rows and columns labeled accordingly. The digits marked in red and green indicate the genes that are As-induced and As-repressed, respectively. Gray stands for no regulated genes. The intensities relates to the numbers of the regulated genes (light color, no.1-3; middle, no.4-10; darker, 11-17). The gene lists in each functional category were obtained from The Arabidopsis Information Center (http://www.arabidopsis.org/) and Arabidopsis thaliana Kinase Database (http://bioinformatics.cau.edu.cn/athKD/index.htm). ABA, Absicisc acid; ABC, ATP-binding cassette; CK, Cytokinin; ET, Ethylene; HSF, Heat shock factors; LeOPT1, oligopeptide transporters; LRR-RLK VIII, Leucine-Rich Repeats receptor like kinase; MAPK, Mitogen-activated protein kinase; MATE, Multidrug and toxic compound extrusion; SnRK, Snf1-related kinases.

Supplementary Table S3 Oxidative stress-related genes in response to As stress

AGI	Family	Col-100 q-value	Regulation	Col-100 FC Absolute	Ws-100 q-value	Regulation	Ws-100 FC Absolute	Col-200 q-value	Regulation	Col-200 FC Absolute
At1g71695	Class III peroxidase	0.009623421	up	1.6229894	0.024285028	up	1.2606744	0.014016178	up	1.6299086
At1g14540	Class III peroxidase	0.052864652	up	3.1117535	0.4304437	up	1.315416	0.029231686	up	3.2177277
At4g36430	Class III peroxidase	0.025234912	up	3.4024436	0.005592544	up	1.9977249	0.002963268	up	7.0356903
At5g51890	Class III peroxidase	0.31583008	down	0.865570646	0.10244181	up	1.3144463	0.97194546	up	1.0051214
At4g26010	Class III peroxidase	0.4338229	down	0.860033429	0.19510646	up	1.1319033	0.092003785	up	1.2319552
At5g22410	Class III peroxidase	0.11176211	up	1.2489964	0.01031717	down	0.404727705	0.6096551	up	1.0393777
At4g08770	Class III peroxidase	0.13211519	up	1.2492714	0.5453687	down	0.926922282	0.11078271	up	1.2464876
At5g67400	Class III peroxidase	0.5420455	down	0.845640575	0.42191243	down	0.912884942	0.3198591	up	1.141518
At2g43480	Class III peroxidase	0.07592735	up	1.5261446				0.13939662	up	1.340784
At5g39580	Class III peroxidase	0.03397972	up	6.7273602				0.021177657	up	5.969698
At1g14550	Class III peroxidase	0.050427955	up	7.249209				0.012523931	up	16.054934
At5g66390	Class III peroxidase	0.1807912	up	1.3479922	0.023780236	up	1.4486605	0.2481897	down	0.808662653
At1g68850	Class III peroxidase	0.28472003	up	2.0894983	0.018554265	up	3.8923454	0.68582153	down	0.788705361
At4g33420	Class III peroxidase	0.04146793	down	0.6459385	0.17107089	up	1.1733886	0.002958026	down	0.438081784
At5g14130	Class III peroxidase	0.03182949	down	0.126350785	0.3651273	up	1.1644521	0.0088989	down	0.080285927
At5g24070	Class III peroxidase	0.8770867	up	1.0208443	0.12847503	down	0.826198695	0.012590182	down	0.697389705
At4g11290	Class III peroxidase	0.049089372	up	1.2348944	0.35051146	down	0.888177794	0.00362326	down	0.642097244
At2g39040	Class III peroxidase	0.3121839	up	1.288929	0.05228163	down	0.571428996	0.20591095	down	0.637455035
At5g64120	Class III peroxidase	0.017662406	up	1.4882114	0.8132941	down	0.902009316	0.30806345	down	0.906917193
At1g34510	Class III peroxidase	0.9445653	down	0.978888891	0.45011154	down	0.812876087	0.20539625	down	0.88908844
At5g64100	Class III peroxidase	0.35256758	down	0.938286108	0.43019918	down	0.901031916	0.4947556	down	0.927563935
At4g30170	Class III peroxidase	0.16224606	down	0.895851848	0.014273183	down	0.719235027	0.006854149	down	0.63420859
At3g49960	Class III peroxidase	0.61322355	down	0.876667981	0.07935232	down	0.78878269	0.123917624	down	0.833011999
At1g30870	Class III peroxidase	0.51343215	down	0.839580089	0.5615146	down	0.901947241	0.43297797	down	0.902609679
At3g28200	Class III peroxidase	0.20774283	down	0.824592923	0.6526163	down	0.915800815	0.004223302	down	0.52463029
At2g18980	Class III peroxidase	0.1407412	down	0.802941528	0.05453878	down	0.823980672	0.003681218	down	0.421697162
At5g17820	Class III peroxidase	0.053191107	down	0.770201163	0.60953987	down	0.946685333	0.005636592	down	0.763410994
At1g49570	Class III peroxidase	0.38687757	down	0.770188113	0.38071796	down	0.697466411	0.017486969	down	0.254142911
At3g01190	Class III peroxidase	0.1596593	down	0.759092563	0.044646982	down	0.803332609	0.02095191	down	0.575865102
At4g21960	Class III peroxidase	0.07490218	down	0.705358744	0.07299163	down	0.827838075	0.009291781	down	0.49933344
At1g44970	Class III peroxidase	0.045904167	down	0.692647361	0.015349816	down	0.335525613	0.001541871	down	0.27432607
At1g05260	Class III peroxidase	0.007118106	down	0.565304898	0.032110117	down	0.669955254	0.002608024	down	0.337597311
At5g42180	Class III peroxidase	0.015063508	down	0.541708816	0.012320777	down	0.614961824	0.001599981	down	0.337716944
At2g37130	Class III peroxidase	0.04977266	down	0.49300811	0.045228474	down	0.588059948	0.002142909	down	0.358890577
At3g21770	Class III peroxidase	0.013568438	down	0.451967943	0.006927837	down	0.501166113	0.001100269	down	0.25342608
At2g35380	Class III peroxidase	0.05720024	down	0.160607998	0.16013359	down	0.387746618	0.011755955	down	0.073645615
At5g15180	Class III peroxidase	0.012721254	down	0.121785339	0.04215532	down	0.605291226	0.01163845	down	0.122411637
At5g19890	Class III peroxidase	0.007430282	down	0.065716375	0.011761585	down	0.135227364	0.00028439	down	0.006965864
At5g64110	Class III peroxidase	0.21836959	down	0.481625157				0.004747035	down	0.308340582
At2g41480	Class III peroxidase				0.122177	down	0.487127984			
At5g02790	GST	0.88073874	up	1.0288192	0.6919936	up	1.08323	0.7354013	up	1.040551
At3g43800	GST	0.6448041	up	1.1307299	0.013728089	up	1.3316107	0.61103976	up	1.1107816
At2g47730	GST	0.12885205	up	1.3738304	0.010260057	up	1.5210145	0.07318565	up	1.4175034
At2g29440	GST	0.048887838	up	1.3909805	0.019460352	up	1.3525316	0.054005675	up	1.3859166
At1g17180	GST	0.48177263	up	1.4301051	0.004857321	up	5.226189	0.070109054	up	2.9220455
At2g02390	GST	0.09897293	up	1.4609307	0.68328476	up	1.157265	0.012188795	up	1.7409939
At1g78340	GST	0.41729745	up	1.5341747	0.005592544	up	3.8900702	0.0972118	up	2.4947934
At2g29490	GST	0.24035396	up	1.6378978	0.00631125	up	3.542958	0.1022186	up	1.9862201
At2g29420	GST	0.24313708	up	1.7121366	0.0058567	up	2.621116	0.11768151	up	1.9823742
At2g29480	GST	0.04835256	up	2.6640196	0.001810002	up	6.5522604	0.017952483	up	3.3363395
At1g75270	GST	0.027359206	up	2.6973143	0.005686936	up	2.3186395	0.006035101	up	4.02002
At1g74590	GST	0.017765895	up	4.0109096	0.008154522	up	3.4928033	0.004747035	up	6.22931
At2g29450	GST	0.04491807	up	4.0317054	0.01910552	up	2.9835923	0.007524552	up	9.367345
At1g17170	GST	0.081035584	up	4.042193	0.007138968	up	3.533991	0.02150356	up	7.0995274
At2g29460	GST	0.029082093	up	6.7264175	9.92153E-05	up	8.043762	0.006409871	up	13.120436
At5g02780	GST	0.013832902	up	8.729493	0.005486214	up	5.008062	0.003743039	up	10.716657
At1g69930	GST	0.069407366	up	9.870241	0.005060274	up	27.361132	0.015220757	up	30.032288
At3g09270	GST	0.34361264	up	1.3246387				0.19013186	up	1.4160527
At1g69920	GST	0.000390698	up	567.8651				0.000182259	up	745.64
At1g27130	GST	0.8882594	up	1.0214627	0.9692111	up	1.0018381	0.528857	down	0.967231175
At1g78380	GST	0.7631704	up	1.0578488	0.01109956	up	1.4401522	0.9901012	down	0.997845552
At5g16710	GST	0.302329	down	0.898452335	0.22593026	up	1.1674773	0.550108	down	0.95551002
At1g78320	GST	0.034891054	down	0.41908865	0.50916106	up	1.1366547	0.006324846	down	0.156180963
At5g62480	GST	0.0642922	down	0.34264259	0.002933816	up	2.9362693	0.6913417	down	0.917049565
At1g02950	GST	0.6924051	up	1.0178201	0.9594534	down	0.994421198	0.88322353	down	0.990025199
At1g53680	GST	0.6379078	down	0.942128442	0.015866674	down	0.4713706	0.20592178	down	0.782496921
At5g41210	GST	0.18898496	down	0.868213145	0.09489916	down	0.86432941	0.15201947	down	0.867225142
At2g30870	GST	0.045301057	down	0.83841813	0.34444752	down	0.955355931	0.009994289	down	0.71052223
At1g27140	GST	0.19000201	down	0.832571669	0.012892112	down	0.555617013	0.012526467	down	0.58482104
At1g17190	GST	0.06844585	down	0.601517798	0.05624612	down	0.653945679	0.006231648	down	0.300888976
At2g30860	GST	0.007445758	down	0.547949829	0.03267884	down	0.673570767	0.00053161	down	0.362895397
At1g78370	GST	0.01103637	down	0.128113663	0.03835354	down	0.583601737	0.001276096	down	0.199142639
At3g03190	GST	0.003941437	down	0.054642333	0.010584146	down	0.27907748	0.001100269	down	0.046070317
At1g49860	GST	0.027506806	down	0.039429083	0.060600605	down	0.242049474	0.016766641	down	0.047243326
At3g62760	GST	0.038216308	down	0.668695891				0.014913993	down	0.628064602
At1g10370	GST				0.007138968	up	2.160197			
At3g43800	GST::Tau family	0.6448041	up	1.1307299	0.013728089	up	1.3316107	0.61103976	up	1.1107816
At2g29440	GST::Tau family	0.048887838	up	1.3909805	0.019460352	up	1.3525316	0.054005675	up	1.3859166
At1g17180	GST::Tau family	0.48177263	up	1.4301051	0.004857321	up	5.226189	0.070109054	up	2.9220455
At1g78340	GST::Tau family	0.41729745	up	1.5341747	0.005592544	up	3.8900702	0.0972118	up	2.4947934
At2g29490	GST::Tau family	0.24035396	up	1.6378978	0.00631125	up	3.542958	0.1022186	up	1.9862201
At2g29420	GST::Tau family	0.24313708	up	1.7121366	0.0058567	up	2.621116	0.11768151	up	1.9823742
At2g29480	GST::Tau family	0.04835256	up	2.6640196	0.001810002	up	6.5522604	0.017952483	up	3.3363395
At1g74590	GST::Tau family	0.017765895	up	4.0109096	0.008154522	up	3.4928033	0.004747035	up	6.22931
At2g29450	GST::Tau family	0.04491807	up	4.0317054	0.01910552	up	2.9835923	0.007524552	up	9.367345
At1g17170	GST::Tau family	0.081035584	up	4.042193	0.007138968	up	3.533991	0.02150356	up	7.0995274
At2g29460	GST::Tau family	0.029082093	up	6.7264175	9.92153E-05	up	8.043762	0.006409871	up	13.120436
At1g69930	GST::Tau family	0.069407366	up	9.870241	0.005060274	up	27.361132	0.015220757	up	30.032288
At3g09270	GST::Tau family	0.34361264	up	1.3246387				0.19013186	up	1.4160527
At1g69920	GST::Tau family	0.000390698	up	567.8651				0.000182259	up	745.64
At1g27130	GST::Tau family	0.8882594	up	1.0214627	0.9692111	up	1.0018381	0.528857	down	0.967231175
At1g78380	GST::Tau family	0.7631704	up	1.0578488	0.01109956	up	1.4401522	0.9901012	down	0.997845552
At1g78320	GST::Tau family	0.034891054	down	0.41908865	0.50916106	up	1.1366547	0.006324846	down	0.156180963
At5g62480	GST::Tau family	0.0642922	down	0.34264259	0.002933816	up	2.9362693	0.6913417	down	0.917049565
At1g53680	GST::Tau family	0.6379078	down	0.942128442	0.015866674	down	0.4713706	0.20592178	down	0.782496921
At1g27140	GST::Tau family	0.19000201	down	0.832571669	0.012892112	down	0.555617013	0.012526467	down	0.58482104
At1g17190	GST::Tau family	0.06844585	down	0.601517798	0.05624612	down	0.653945679	0.006231648	down	0.300888976
At1g78370	GST::Tau family	0.01103637	down	0.128113663	0.03835354	down	0.583601737	0.001276096	down	0.199142639
At1g10370	GST::Tau family				0.007138968	up	2.160197			
At4g22260	ROS-Alternative Oxidase (AOX)	0.95769054	up	1.0113246	0.19849765	up	1.0463938	0.594936	up	1.0778748
At3g22370	ROS-Alternative Oxidase (AOX)	0.058531422	up	3.0135972	0.002243847	up	4.676477	0.007774778	up	6.3370113
At1g07890	ROS-Ascorbate Peroxidase (APX)	0.5319627	up	1.045205	0.22881696	up	1.0811132	0.2613195	up	1.0523505
At3g09640	ROS-Ascorbate Peroxidase (APX)	0.004846979	up	57.950687	0.001810002	up	50.935005	0.000245147	up	299.91833
At4g35000	ROS-Ascorbate Peroxidase (APX)	0.62220806	up	1.0665071	0.061635487	down	0.795854743	0.7621532	up	1.0363522
At4g08390	ROS-Ascorbate Peroxidase (APX)	0.2973339	up	1.0668044	0.026649283	down	0.798162566	0.19648695	up	1.0644243
At2g33740	ROS-Blue copper protein	0.040974054	up	1.5048143	0.091011584	up	1.2898992	0.000693663	up	2.3063912
At5g20230	ROS-Blue copper protein	0.004063893	up	10.959043				0.001408073	up	14.819119
At1g72230	ROS-Blue copper protein	0.01375573	down	0.345855038	0.049068242	up	1.3858222	0.001337301	down	0.140583328
At4g12880	ROS-Blue copper protein	0.06851604	down	0.686524243	0.009669296	down	0.646508489	0.003310761	down	0.428916007
At3g27200	ROS-Blue copper protein	0.54209644	up	1.1191286				0.9964211	down	0.999254856
At1g75270	ROS-Dehydroascorbate Reductase (DHAR)	0.027359206	up	2.6973143	0.005686936	up	2.3186395	0.006035101	up	4.02002
At5g16710	ROS-Dehydroascorbate Reductase (DHAR)	0.302329	down	0.898452335	0.22593026	up	1.1674773	0.550108	down	0.95551002
At2g40300	ROS-Ferritin				0.012760867	down	0.659069615	0.004447613	up	1.6990316
At3g11050	ROS-Ferritin	0.062419467	up	1.2118914				0.003222619	up	1.7474413
At3g56090	ROS-Ferritin	0.010643841	down	0.463124992	0.023155449	down	0.701410719	0.001446554	down	0.415525081
At5g01600	ROS-Ferritin	0.022054264	down	0.408615811	0.02195779	down	0.498920038	0.004433525	down	0.292113858
At2g20270	ROS-Glutaredoxin (GLR)	0.1931259	up	1.3066924	0.28701898	up	1.0491693	0.068742126	up	1.5713081
At1g28480	ROS-Glutaredoxin (GLR)	0.17015953	up	1.8395559	0.006778613	up	2.9634044	0.074723825	up	2.2388492
At5g40370	ROS-Glutaredoxin (GLR)	0.014016773	up	1.9867524	0.7274738	up	1.0467678	0.002197085	up	2.2537131
At5g63030	ROS-Glutaredoxin (GLR)	0.30738154	up	1.1843034	0.53146183	down	0.86721153	0.25267133	up	1.1702977
At5g20500	ROS-Glutaredoxin (GLR)	0.6924051	down	0.966853086	0.026917968	down	0.864400836	0.046397198	down	0.848626718
At1g77370	ROS-Glutaredoxin (GLR)	0.03941183	down	0.864504259	0.9904544	down	0.998627287	0.018592509	down	0.796711621
At1g03850	ROS-Glutaredoxin (GLR)	0.34404483	down	0.736158508	0.029133752	down	0.374499351	0.16761093	down	0.667973445
At1g06830	ROS-Glutaredoxin (GLR)	0.9498411	up	1.0159025				0.8335552	down	0.936106605
At4g33040	ROS-Glutaredoxin (GLR)				0.00655279	up	2.6491234			
At2g25080	ROS-Glutathione Peroxidase (GPX)	0.9487344	up	1.0146849	0.91405886	up	1.0116549	0.25154093	up	1.2713213
At2g31570	ROS-Glutathione Peroxidase (GPX)	0.19351944	up	1.2676136	0.058553934	up	1.130015	0.013251347	up	1.3702636
At4g11600	ROS-Glutathione Peroxidase (GPX)	0.8092025	down	0.974356211	0.052346665	up	1.3841023	0.33515236	down	0.920046234
At2g43350	ROS-Glutathione Peroxidase (GPX)	0.43585673	up	1.0729157	0.4039578	down	0.913318244	0.22345178	down	0.900580343

At1g63460	ROS-Glutathione Peroxidase (GPX)	0.2028634	down	0.741175124	0.6449537	down	0.974926166	0.007716105	down	0.659730654
At3g63080	ROS-Glutathione Peroxidase (GPX)	0.05140126	down	0.637107796	0.6359278	down	0.956842573	0.006555511	down	0.568521122
At3g24170	ROS-Glutathione Reductase (GR)	0.13595785	up	1.4277598	0.005459929	up	1.6304837	0.10199557	up	1.4137892
At3g54660	ROS-Glutathione Reductase (GR)	0.20695868	up	1.2630018	0.90233606	down	0.991435976	0.013788533	up	1.5969698
At1g20630	ROS-Glutathione Reductase (GR)	0.08414417	up	1.4966578				0.2012185	up	1.2159444
At4g35090	ROS-Glutathione Reductase (GR)	0.019288165	down	0.551710745	0.020383343	down	0.639488655	0.003801287	down	0.42377559
At1g20620	ROS-Glutathione Reductase (GR)	0.005863104	down	0.453387256	0.30240706	down	0.659574308	0.003881403	down	0.211501091
At3g52880	ROS-Monodehydroascorbate Reductase (MDAR)	0.04876648	up	1.1822935	0.16761628	up	1.127347	0.016744645	up	1.3170571
At3g09940	ROS-Monodehydroascorbate Reductase (MDAR)	0.8812133	down	0.978131714	0.84254634	down	0.985367587	0.36252972	down	0.885044883
At5g03630	ROS-Monodehydroascorbate Reductase (MDAR)	0.20315675	down	0.834348527	0.2339204	down	0.902063913	0.083509006	down	0.677228359
At3g27820	ROS-Monodehydroascorbate Reductase (MDAR)	0.15684745	down	0.812489128	0.1194164	down	0.880961256	0.13458902	down	0.817507078
At1g63940	ROS-Monodehydroascorbate Reductase (MDAR)	0.025673691	down	0.619973411	0.016206117	down	0.630335276	0.001446053	down	0.580177246
At5g47910	ROS-NADPH oxidase	0.03271642	up	1.9796085	0.09733662	up	1.1499012	0.011985579	up	2.2205114
At5g51060	ROS-NADPH oxidase	0.64735854	up	1.0529886	0.059710395	up	1.3399713	0.91291815	down	0.98515616
At1g64060	ROS-NADPH oxidase	0.2056929	down	0.872765916	0.25097507	up	1.1342543	0.005658592	down	0.611514874
At1g09090	ROS-NADPH oxidase	0.09038103	down	0.71713844	0.6710089	down	0.914920198	0.008808441	down	0.432469386
At4g25090	ROS-NADPH oxidase	0.026803706	down	0.490987748	0.14812656	down	0.793713661	0.01699483	down	0.511138866
At5g67590	ROS-NADPH oxidase-like	0.0857445	up	1.1886952	0.42207268	up	1.0522457	0.115687534	up	1.1291788
At1g23020	ROS-NADPH oxidase-like	0.87815374	down	0.96763896	0.190202	up	1.6927042	0.029260986	down	0.528610795
At1g65980	ROS-Peroxiredoxin (PrxR)	0.08020317	up	1.2760978	0.028529903	up	1.3460001	0.009134709	up	1.4061611
At5g06290	ROS-Peroxiredoxin (PrxR)	0.028693317	up	2.1468074	0.0746986	up	1.4519497	0.000722367	up	3.9690216
At3g06050	ROS-Peroxiredoxin (PrxR)	0.9874275	down	0.998101113	0.31673098	down	0.97127748	0.80038786	up	1.0256644
At3g11630	ROS-Peroxiredoxin (PrxR)	0.6749684	down	0.981779739	0.07441278	down	0.874907708	0.054091796	down	0.8278034
At3g52960	ROS-Peroxiredoxin (PrxR)	0.1918908	down	0.96685804	0.011804807	down	0.654212254	0.16128184	down	0.86237377
At5g18100	ROS-Superoxide Dismutase (SOD)	0.06314771	up	1.8511335	0.059431165	up	1.3473322	0.00214195	up	2.6308188
At1g08830	ROS-Superoxide Dismutase (SOD)	0.08968925	up	3.3496253	0.9461076	up	1.0339446	0.07025985	up	3.0603576
At5g23310	ROS-Superoxide Dismutase (SOD)	0.7472724	up	1.0702741	0.02338637	down	0.663278419	0.33653212	up	1.1759167
At3g10920	ROS-Superoxide Dismutase (SOD)	0.042358685	up	1.2363936	0.67675096	down	0.967061124	0.04592033	up	1.1767008
At2g28190	ROS-Superoxide Dismutase (SOD)	0.12885053	up	2.1290357	0.5388162	down	0.87168394	0.14492743	up	1.8437167
At4g25100	ROS-Superoxide Dismutase (SOD)	0.004114812	down	0.012027371	0.9753538	down	0.976363509	0.006570482	down	0.13714603
At5g39950	ROS-Thioredoxins (Trx)	0.053899117	up	1.1532015	0.122184746	up	1.0905998	0.05737649	up	1.1617637
At1g19730	ROS-Thioredoxins (Trx)	0.022452045	up	1.2054126	0.798106	up	1.0223184	0.39069057	up	1.0569099
At2g41680	ROS-Thioredoxins (Trx)	0.035624176	up	1.3967369	0.5735695	up	1.0558746	0.001167245	up	1.8456438
At3g08710	ROS-Thioredoxins (Trx)	0.008896088	up	1.5238706	0.06322342	up	1.3024608	0.001869104	up	2.3642504
At1g45145	ROS-Thioredoxins (Trx)	0.085724846	up	1.6317937	0.0486931	up	1.2921976	0.01083229	up	2.4194837
At4g29670	ROS-Thioredoxins (Trx)	0.040168848	up	1.8147097	0.08859029	up	1.5152771	0.017246287	up	2.2500746
At1g62180	ROS-Thioredoxins (Trx)	0.03197057	up	4.206999	0.003027713	up	3.0113497	0.00881536	up	6.158038
At2g42580	ROS-Thioredoxins (Trx)	0.92267096	down	0.989210483	0.16413827	up	1.1122062	0.9882252	up	1.0019221
At2g04700	ROS-Thioredoxins (Trx)	0.043610018	down	0.865526745	0.074639946	up	1.050474	0.095745526	up	1.2566731
At4g37200	ROS-Thioredoxins (Trx)	0.41477734	up	1.1257938	0.92227525	down	0.996468913	0.09711855	up	1.2872571
At4g03520	ROS-Thioredoxins (Trx)	0.079853825	up	1.2457188	0.054635543	down	0.919798027	0.038412135	up	1.2243139
At1g03680	ROS-Thioredoxins (Trx)	0.17571731	up	1.442367	0.20689444	down	0.775478802	0.035935428	up	1.4322208
At5g42980	ROS-Thioredoxins (Trx)	0.40978175	down	0.95520399	0.19638719	up	1.0887308	0.051658634	down	0.85719353
At1g50320	ROS-Thioredoxins (Trx)	0.12291895	down	0.862083532	0.7748233	up	1.0301903	0.048510697	down	0.842551833
At2g15570	ROS-Thioredoxins (Trx)	0.039065346	down	0.801307477	0.14696038	up	1.111325	0.025692265	down	0.798845381
At1g31020	ROS-Thioredoxins (Trx)	0.23735368	down	0.771933129	0.0777375	up	1.1344833	0.4085174	down	0.853433397
At4g04950	ROS-Thioredoxins (Trx)	0.46092004	up	1.088449	0.18441711	down	0.906050533	0.93102163	down	0.991605071
At1g53300	ROS-Thioredoxins (Trx)	0.68753916	down	0.9437724	0.5179259	down	0.931891759	0.051052466	down	0.754620959
At3g20560	ROS-Thioredoxins (Trx)	0.4412959	down	0.890639689	0.17434804	down	0.848150163	0.06297998	down	0.818959167
At1g76760	ROS-Thioredoxins (Trx)	0.20141509	down	0.822802355	0.606419	down	0.933073441	0.078972094	down	0.798284264
At3g51030	ROS-Thioredoxins (Trx)	0.10088843	down	0.770515396	0.014414949	down	0.821176162	0.01252705	down	0.593659984
At3g15360	ROS-Thioredoxins (Trx)	0.023229754	down	0.5562384	0.015386242	down	0.611234353	0.006091931	down	0.510349635

Supplementary Table S3 Oxidative stress-related genes in response to As stress (continued)

Functional categories	In genome	On array	Col-0 with100 uM As/Control		Ws-2 with 100 uM As/Control				Col-0 with 200 uM As/Control		
			Detected	Induced	Repressed	Detected	Induced	Repressed	Detected	Induced	Repressed
Reactive oxygen species (ROS) network											
Alternative Oxidase (AOX)	6	6	2	1	0	2	1	0	2	1	0
Ascorbate Peroxidase (APX)	9	9	4	0	0	4	1	0	4	1	0
Blue copper protein	9	9	5	1	1	3	0	0	5	2	2
Dehydroascorbate Reductase (DHAR)	5	5	2	1	0	2	1	0	2	1	0
Ferritin	4	4	3	0	2	3	0	1	4	0	2
Glutaredoxin (GLR)	27	25	8	0	0	8	2	1	8	1	0
Glutathione Peroxidase (GPX)	8	8	6	0	0	6	0	0	6	0	0
Glutathione Reductase (GR)	5	5	5	0	1	4	0	0	5	0	2
Monodehydroascorbate Reductase (MDAR)	5	5	5	0	0	5	0	0	5	0	0
NADPH oxidase	10	10	5	0	1	5	0	0	5	1	1
NADPH oxidase-like	9	8	2	0	0	2	0	0	2	0	0
Peroxiredoxin (PrxR)	11	11	5	1	0	5	0	0	5	1	0
Superoxide Dismutase (SOD)	8	8	6	0	0	6	0	0	6	1	1
Thioredoxins (Trx)	32	30	22	1	0	22	1	0	22	4	0
Class III peroxidase	73	72	39	2	5	36	1	3	39	4	14
Glutathion-S-transferase	53	52	35	7	4	33	14	2	35	9	6
GST-Tau family	28	28	22	5	2	21	12	0	22	7	3
Total No.				19	16		33	7		33	31

Supplementary Table S4 Transporter-related genes in response to As stress

AGI Locus	family	Col-100 Corrected p-value	Regulation	Col FC Absolute	Ws-100 Corrected p-value	Regulation	Ws-100 FC Absolute	Col-200 Corrected p-value	Col-200 regulation	Col-200 FC Absolute
At1g54115		0.21158007	up	1.101068	0.009131826	up	1.1749021	0.038728878	up	1.1725571
At1g79610		0.05395756	up	1.8779945	0.07815227	up	1.5611835	0.028814591	up	2.1057847
At3g04090		0.32459417	up	1.1579959	0.068930574	up	1.1043521	0.026976224	up	1.2547541
At5g18290								0.001084539	up	19.224548
At1g01790		0.066289656	down	0.612015667	0.19849765	down	0.697817715	0.01676219	down	0.536960206
At1g20840		0.006927019	down	0.674944638	0.019457972	down	0.700389487	0.001389125	down	0.42284142
At1g54370		0.04467999	down	0.710596753	0.27910066	down	0.852775114	0.004773038	down	0.659101934
At3g56950		0.047578275	down	0.779580176	0.050934315	down	0.858027803	0.00219383	down	0.543266564
At4g00630		0.006649351	down	0.577398909	0.015589097	down	0.600789714	0.001599981	down	0.390870393
At5g47560		0.007057693	down	0.349065263	0.03387688	down	0.62636073	0.000941794	down	0.152894655
At1g03905	ABC	0.023315016	up	4.632699	0.024466237	up	2.6492767	0.003171321	up	9.129602
At5g02270	ABC	0.27425432	up	1.1287733	0.026149169	up	1.3245384	0.007849352	up	1.3186111
At5g64840	ABC	0.26807445	up	1.2384684	0.09797752	up	1.2564026	0.013208684	up	1.3086519
At1g65410	ABC	0.35523075	up	1.1201066	0.21405847	down	0.854191041	0.17714316	up	1.1837405
At5g44110	ABC	0.071047835	down	0.847577294	0.30607623	up	1.1145184	0.64582366	down	0.948854377
At1g67940	ABC	0.04417463	down	0.688269614	0.065120086	down	0.786404204	0.019822992	down	0.643490084
At1g30400	ABC transporters	0.19924903	up	1.345271	0.006778613	up	1.8223221	0.20779787	up	1.2994262
At1g53390	ABC transporters	0.6866126	up	1.1363412	0.33988953	up	1.142231	0.41146353	up	1.2142726
At1g59870	ABC transporters	0.30707812	up	1.3934298	0.038864918	up	1.3731543	0.054132383	up	1.47563
At2g34660	ABC transporters	0.10209596	up	1.7987231	0.000612937	up	2.8254018	0.02159418	up	2.4326234
At2g36910	ABC transporters	0.15772107	up	1.2078185	0.038061347	up	1.3211617	0.019299613	up	1.3369589
At2g39350	ABC transporters	0.5157218	up	1.2435905	0.006885885	up	4.2735066	0.006530769	up	3.694496
At2g39480	ABC transporters	0.024252024	up	1.4900566	0.04141387	up	1.2346042	0.002435544	up	2.4895666
At2g47000	ABC transporters	0.036625702	up	2.4820302	0.000612937	up	3.3320487	0.009116313	up	3.5166118
At3g21250	ABC transporters	0.16180256	up	1.6561939	0.047130916	up	1.5276469	0.029351955	up	2.4848895
At3g47730	ABC transporters	0.1604318	up	1.4641966	0.027471347	up	1.708468	0.044502713	up	1.7183337
At3g55090	ABC transporters	0.028425088	up	20.627628	0.004294924	up	41.932697	0.030512102	up	8.8476715
At3g59140	ABC transporters	0.47487378	up	1.3654039	0.018233245	up	1.7781616	0.30539733	up	1.5228065
At3g62150	ABC transporters	0.009550108	up	3.1418796	0.3634016	up	1.1691364	0.00227261	up	4.968078
At5g39040	ABC transporters	0.03863179	up	3.9286916	0.0087585	up	2.8646965	0.00646568	up	6.692638
At3g47780	ABC transporters	0.41221875	down	0.738950346	0.003341037	up	3.6964529	0.47565055	up	1.2605184
At2g28070	ABC transporters	0.005434209	up	1.3911273	0.32642397	down	0.944203164	0.006351619	up	1.2432497
At4g25450	ABC transporters	0.1781926	up	1.2157544	0.46151605	down	0.896652384	0.019946698	up	1.5384799
At5g58270	ABC transporters	0.31493855	up	1.1122414	0.642143	down	0.965188172	0.011257024	up	1.2895192
At3g13100	ABC transporters	0.028400606	up	3.3410213				0.007063367	up	3.9649405
At5g13580	ABC transporters	0.9881599	up	1.0093598	0.008618869	up	4.0263023	0.0973251	down	0.298833611
At1g71960	ABC transporters	0.033043284	down	0.438354819	0.11348282	up	1.2784139	0.02388488	down	0.221327616
At2g36380	ABC transporters	0.0825675	down	0.734222315	0.18583687	up	1.1822816	0.007165048	down	0.599225154
At2g47800	ABC transporters	0.3058086	down	0.876697725	0.019147428	up	1.383973	0.004454608	down	0.691150823
At3g16340	ABC transporters	0.008132204	down	0.664856528	0.34000313	up	1.064008	0.000860154	down	0.309162513
At3g53480	ABC transporters	0.15202394	down	0.640528123	0.025994802	up	1.8712432	0.19919081	down	0.752663941
At3g55320	ABC transporters	0.7274907	down	0.952398096	0.29507628	up	1.0992854	0.05075064	down	0.745082974
At1g04120	ABC transporters	0.17064989	down	0.862972781	0.27616972	down	0.932090931	0.006362627	down	0.762470064
At1g15210	ABC transporters	0.007057693	down	0.438785477	0.02787128	down	0.617181544	0.000519851	down	0.195845172
At1g31770	ABC transporters	0.00186457	down	0.353486381	0.003660176	down	0.316032453	0.001599981	down	0.057390301
At2g41700	ABC transporters	0.062336802	down	0.74972101	0.025298882	down	0.803498432	0.01699483	down	0.614437134
At3g28860	ABC transporters	0.6149891	down	0.939992743	0.10005079	down	0.866728828	0.001957762	down	0.661805134
At3g62700	ABC transporters	0.00492641	down	0.561803971	0.93685836	down	0.990765668	0.004453586	down	0.505153653
At4g15230	ABC transporters	0.4297074	up	1.2908486				0.12380414	down	0.745609516
At3g28345	ABC transporters				0.21437901	up	2.0410066			
At3g53510	ABC transporters				0.08036525	up	2.6341386			
At3g10670	ABC(SufC)	0.006862041	down	0.75321334	0.16792263	down	0.854172801	0.034508687	down	0.727062487
At4g39850	ABCD(FAE)	0.024725249	up	1.64142	0.08730225	up	1.1717178	0.009345871	up	1.7249846
At3g06450	Antiporters	0.8902891	up	1.0285264	0.1679313	up	1.151369	0.43636447	up	1.1049833
At4g28390	Antiporters	0.011198661	up	8.950169	0.02217697	up	3.2167835	0.000909821	up	23.060575
At5g13490	Antiporters	0.002834522	up	2.1827867	0.10439992	up	1.361187	0.00183881	up	2.7160473
At5g17400	Antiporters	0.005021514	up	2.9381406	0.056757346	up	1.7319503	0.001084539	up	6.485056
At5g01490	Antiporters							0.02065954	up	1.9870529
At5g41610	Antiporters							0.000560826	up	4.899086
At1g64170	Antiporters	0.21202081	down	0.72516984	0.007676224	up	2.4809413	0.027494289	down	0.270789088
At3g13320	Antiporters	0.04298926	down	0.777142196	0.72482586	up	1.0383627	0.012666835	down	0.796117114
At3g51860	Antiporters	0.022711512	down	0.166911554	0.20415299	up	1.456134	0.001377628	down	0.158933543
At4g23700	Antiporters	0.14010943	down	0.265825171	0.01502979	up	2.3530939	0.21370786	down	0.407867037
At2g19600	Antiporters	0.8820476	up	1.0115296	0.07415801	down	0.799755275	0.041518595	down	0.834138973
At2g47600	Antiporters	0.5442338	up	1.0743569	0.3383224	down	0.853161227	0.052694567	down	0.740221653
At2g01980	Antiporters	0.73525393	down	0.942526612	0.83030033	down	0.932302878	0.006880031	down	0.510352084
At2g38170	Antiporters	0.027402414	down	0.536805708	0.018554265	down	0.59316238	0.00199188	down	0.305067181
At2g47160	Antiporters	0.030577628	down	0.282336795	0.00614898	down	0.231730914	0.005260802	down	0.14155663
At3g08580	Antiporters	0.09463251	down	0.858346848	0.98830193	down	0.999161703	0.018666428	down	0.807333103
At3g19490	Antiporters	0.14234363	down	0.861838869	0.57748234	down	0.927225243	0.101748556	down	0.84529982
At3g62270	Antiporters	0.001669626	down	0.161690585	0.015866674	down	0.521212352	0.000424419	down	0.138941436
At5g11800	Antiporters	0.5570443	down	0.919029714	0.029557064	down	0.74925219	0.038571697	down	0.679835858
At5g12860	Antiporters	0.06701802	down	0.666993894	0.009211053	down	0.519612137	0.010100544	down	0.478766628
At5g17630	Antiporters	0.09590786	down	0.793329495	0.006206627	down	0.531184063	0.002535919	down	0.647044608
At5g27150	Antiporters	0.017401988	down	0.655135753	0.018233245	down	0.655682671	0.003297276	down	0.51500474
At5g54800	Antiporters	0.1594945	down	0.768626333	0.11128939	down	0.753155477	0.03564637	down	0.701099731
At5g64290	Antiporters	0.23433676	down	0.792490675	0.003973629	down	0.746533286	0.004174702	down	0.558746744
At5g51710	Antiporters	0.06520963	down	0.682429515				0.029454969	down	0.550580995
At5g46110	Antiporters				0.7065468	down	0.803480807			
At3g05030	Antiporters::Na+/H+ exchanger	0.00492641	up	4.6428347				0.000822181	up	11.376742
At3g06370	Antiporters::Na+/H+ exchanger				0.0790592	up	1.4231238			
At4g18910	aquaporin	0.10508088	up	2.0760813	0.058790978	up	1.7132641	0.08884979	up	1.7695185
At4g00430	aquaporin	0.038544077	down	0.648435513	0.14026241	up	1.2145765	0.009526052	down	0.542833292
At4g19030	aquaporin	0.46287465	up	1.1035659	0.18631801	down	0.84245466	0.015220757	down	0.616148867
At1g01620	aquaporin	0.030246312	down	0.616406903	0.16490553	down	0.802574917	0.002979851	down	0.351791778
At2g25810	aquaporin	0.22822338	down	0.866406675	0.47105646	down	0.922163924	0.11598845	down	0.81126049
At2g36830	aquaporin	0.020838594	down	0.614641863	0.0640298	down	0.824840841	0.002611483	down	0.50008639
At2g45960	aquaporin	0.050507266	down	0.764645484	0.038913757	down	0.793218678	0.005980641	down	0.551510776
At3g16240	aquaporin	0.024953123	down	0.514101205	0.01467628	down	0.681296417	0.003133086	down	0.250915503
At3g26520	aquaporin	0.020377243	down	0.526939325	0.030462073	down	0.76608	0.001380261	down	0.352520967
At3g53420	aquaporin	0.027525045	down	0.49644343	0.08468962	down	0.648152535	0.004423013	down	0.337155735
At3g61430	aquaporin	0.006722895	down	0.362675143	0.007413642	down	0.566986122	0.000787158	down	0.161907897
At4g10380	aquaporin	0.013507485	down	0.538174695	0.1741431	down	0.736354305	0.000972307	down	0.53160204
At4g17340	aquaporin	0.001549744	down	0.190907004	0.027246432	down	0.603941978	0.001083083	down	0.100712744
At4g23400	aquaporin	0.06117905	down	0.50953986	0.058438372	down	0.625386254	0.007411611	down	0.296179422
At5g47450	aquaporin	0.005731739	down	0.042413172	0.014103841	down	0.316323941	0.001648161	down	0.035147275
At5g60660	aquaporin	0.037060525	down	0.399759585	0.018233245	down	0.495646636	0.004194414	down	0.210286235
At3g20320	binding protein	0.16720912	down	0.833605853	0.93093634	up	1.0095392	0.11613921	down	0.818340517
At1g62200	LeOPT1	0.06808661	up	1.306751	0.16970828	up	1.1220926	0.0353754	up	1.3225244
At4g21680	LeOPT1	0.007278231	up	14.464963	0.0045258	up	11.761998	0.001982334	up	18.639536
At3g47960	LeOPT1	0.05454097	up	1.6671965	0.04172557	down	0.436758714	0.0609582	up	1.4615151
At3g54140	LeOPT1	0.2577796	down	0.804677558	0.83821815	up	1.0303506	0.008105375	down	0.534647054
At5g46050	LeOPT1	0.30242416	up	1.4537294	0.46693698	down	0.824863702	0.5002479	down	0.766020965
At1g12110	LeOPT1	0.001822189	down	0.211468039	0.007475819	down	0.319599064	0.000446479	down	0.156913622
At1g18880	LeOPT1	0.008725406	down	0.566122131	0.401907	down	0.909229029	0.000783487	down	0.305799316
At1g22540	LeOPT1	0.05965756	down	0.509743492	0.246879	down	0.820258414	0.007152865	down	0.361553997
At1g22550	LeOPT1	0.019083822	down	0.355438251	0.018669752	down	0.587609007	0.002677135	down	0.067709533
At1g32450	LeOPT1	0.0093078	down	0.146449008	0.029213972	down	0.414414943	0.000783487	down	0.036502876
At1g69850	LeOPT1	0.08113293	down	0.749416373	0.9535459	down	0.982611896	0.044941284	down	0.816723892
At2g02040	LeOPT1	0.06444357	down	0.824237393	0.9102533	down	0.98746073	0.038506825	down	0.835146297
At3g16180	LeOPT1	0.008665065	down	0.537308557	0.8973453	down	0.990840374	0.001612004	down	0.390339488
At3g45680	LeOPT1	0.006434278	down	0.038683735	0.044967987	down	0.400896211	0.001440628	down	0.034884121
At3g45700	LeOPT1	0.009623421	down	0.2737781	0.017985906	down	0.128281492	0.000652063	down	0.009885874

At3g45710	LeOPT1	0.022019744	down	0.162846232	0.6177287	down	0.894495187	0.000470407	down	0.04511993
At5g62680	LeOPT1	0.002903	down	0.333728691	0.005443438	down	0.338689551	0.000534231	down	0.10437045
At1g72140	LeOPT1	0.013858877	down	0.544706871				0.00093031	down	0.139096916
At2g02020	LeOPT1	0.007118106	down	0.198554326				0.002108652	down	0.128075091
At3g01350	LeOPT1									
At3g45690	LeOPT1									
At1g15170	MATE	0.30315727	up	1.1446447	0.27664852	up	1.2699575	0.4348693	up	1.1043264
At1g33110	MATE	0.014156688	up	11.066255	0.002792891	up	5.6554046	0.003467166	up	17.068272
At1g47530	MATE	0.003043572	up	1.8066328	0.6085806	up	1.045855	0.000938459	up	1.965481
At1g66760	MATE	0.020838594	up	2.8115282	0.008030331	up	2.7989445	0.003286153	up	3.1184726
At2g04040	MATE	0.003928348	up	24.886911	0.003779925	up	17.528654	0.000826175	up	74.82664
At3g21690	MATE	0.080603845	up	3.6073782	0.004624899	up	4.6950874	0.048208486	up	3.4752479
At5g52450	MATE	0.09335139	up	1.5835279	0.019097894	up	1.5564363	0.0291904	up	1.8073217
At1g61890	MATE	0.088650145	up	1.5028061	0.58799547	down	0.929551867	0.006464917	up	1.8663952
At2g21340	MATE	0.4173377	up	1.2161175	0.8151534	down	0.971731363	0.0694389	up	1.416309
At3g23560	MATE	0.9285584	up	1.0184213	0.014495873	up	1.3645464	0.73830026	down	0.935052558
At3g08040	MATE	0.022175048	down	0.304445393	0.90637505	up	1.0243012	0.001799943	down	0.055137153
At5g38030	MATE	0.033460487	down	0.502517234	0.30450368	up	1.1163758	0.001294623	down	0.226117268
At5g65380	MATE	0.22433522	down	0.829562006	0.17899874	up	1.1954198	0.32268852	down	0.860158598
At1g12950	MATE	0.3123845	up	1.1330476	0.29710677	down	0.861708237	0.004428263	down	0.572055675
At3g26590	MATE	0.94660985	up	1.0067924	0.517513	down	0.959305401	0.060952723	down	0.867392136
At1g11670	MATE	0.105668455	down	0.573110175	0.014301122	down	0.5106017	0.004858039	down	0.204669368
At4g25640	MATE	0.38398257	down	0.823403092	0.1846307	down	0.891606824	0.034508687	down	0.558276128
At4g29140	MATE	0.017888853	down	0.571954541	0.092268206	down	0.604048612	0.007569512	down	0.384609615
At4g39030	MATE	0.05625427	down	0.590919564	0.54174495	down	0.909638677	0.019677049	down	0.609124577
At4g00350	MATE									
At1g19800	membrane	0.47449496	up	1.1741904	0.63658357	down	0.955852335	0.29574212	up	1.2373818
At2g37330	membrane	0.19447358	down	0.702926473	0.8393618	down	0.971563691	0.08415133	down	0.666370931
At4g04770	membrane(SufB)	0.03305794	down	0.642718463	0.008856318	down	0.686063606	0.015174736	down	0.644759252
At1g32500	membrane(SufD)	0.17776574	down	0.836548048	0.44038707	up	1.0995055	0.0428315	down	0.750760389
At1g08930	sugar transporter	0.05448858	up	1.628445	0.018098654	up	1.4516586	0.014558892	up	1.9806906
At1g79820	sugar transporter	0.020058047	up	1.6160896	0.28255752	up	1.1597048	0.001454777	up	1.7844307
At3g18830	sugar transporter	0.08329071	up	1.4959666	0.02190845	up	1.4934101	0.25082195	up	1.2598131
At5g17010	sugar transporter	0.028689183	up	1.5881659	0.008010069	up	2.0801177	0.004667494	up	1.3937246
At5g26340	sugar transporter	0.30082077	up	1.3065381	0.005459929	up	2.9377475	0.898253	up	1.0296892
At1g08920	sugar transporter	0.33804518	down	0.776472751	0.003559839	up	1.6344022	0.90335274	up	1.0227757
At2g20780	sugar transporter	0.3287238	down	0.927988983	0.36729577	up	1.0575995	0.24102637	up	1.0818851
At3g20460	sugar transporter				0.26363483	down	0.904214399	0.5387608	up	1.2129847
At1g19450	sugar transporter	0.034711327	down	0.670287785	0.05568314	up	1.192202	0.00494831	down	0.535927623
At2g48020	sugar transporter	0.00825573	down	0.649378396	0.54042506	up	1.0521623	0.00248279	down	0.645437163
At3g05150	sugar transporter	0.20682144	down	0.788836947	0.1782015	up	1.2121643	0.011260617	down	0.534629989
At5g27350	sugar transporter	0.08111051	down	0.735207082	0.040544126	up	1.4584445	0.016824119	down	0.307049601
At1g11260	sugar transporter	0.029848296	down	0.597752356	0.28518817	down	0.863559574	0.003062179	down	0.34684397
At1g54730	sugar transporter	0.032964908	down	0.750787106	0.15973128	down	0.822800324	0.001016433	down	0.432154228
At1g67300	sugar transporter	0.086579934	down	0.589601748	0.61786485	down	0.927364801	0.045640375	down	0.666237565
At1g75220	sugar transporter	0.006335135	down	0.630306273	0.02058046	down	0.663869607	0.00082477	down	0.406472935
At3g19930	sugar transporter	0.003384795	down	0.434391184	0.43177855	down	0.875872489	0.000541825	down	0.255377268
At4g02050	sugar transporter	0.01883891	down	0.347839589	0.051047396	down	0.646321082	0.00066906	down	0.222446433
At4g36670	sugar transporter	0.08160672	down	0.303711934	0.01033256	down	0.069632069	0.005802603	down	0.044918476
At5g16150	sugar transporter	0.4523029	down	0.932691567	0.01609429	down	0.838153976	0.061136693	down	0.802630187
At2g43330	sugar transporter	0.07587502	down	0.700828947				0.015174736	down	0.520141302
At5g59250	sugar transporter	0.057159767	down	0.641713014				0.038162347	down	0.672403433
At1g64200	V-Type ATPase	0.15223691	up	1.4203717	0.013086765	up	1.7646818	0.00900512	up	2.1552682
At3g01390	V-Type ATPase	0.054999277	up	1.4413338	0.38179708	up	1.0478055	0.045820504	up	1.5203729
At4g23710	V-Type ATPase	0.08371426	up	1.2768837	0.40101668	up	1.0751121	0.04073936	up	1.2959409
At5g13450	V-Type ATPase	0.6575723	up	1.022109	0.555355	up	1.0184087	0.3664933	up	1.0317035
At4g02620	V-Type ATPase	0.108115874	up	1.1043346	0.4343011	down	0.963082635	0.05049391	up	1.1455367
At3g42050	V-Type ATPase	0.025753051	down	0.799387733	0.5636183	up	1.0226146	0.01927867	down	0.764001473
At3g58730	V-Type ATPase	0.72951394	down	0.969381879	0.2676681	up	1.1075206	0.16477533	down	0.880649532
At4g11150	V-Type ATPase	0.39558798	down	0.967750676	0.5879299	up	1.0285815	0.24908444	down	0.943872259
At4g26710	V-Type ATPase	0.33481172	down	0.809429792	0.38589367	up	1.0646544	0.028014848	down	0.8118491
At5g47030	V-Type ATPase	0.47742486	down	0.955948918	0.20205002	up	1.0744698	0.032674484	down	0.825168429
At1g12840	V-Type ATPase	0.121908784	down	0.892940457	0.08273333	down	0.883728386	0.008609708	down	0.834475034
At2g16510	V-Type ATPase	0.48331526	down	0.955615849	0.4424099	down	0.950803253	0.019873291	down	0.784415177
At2g28520	V-Type ATPase	0.08675257	down	0.861645942	0.010032766	down	0.774101295	0.004643171	down	0.747736899
At4g38510	V-Type ATPase	0.053377904	down	0.805898338	0.9499189	down	0.995982208	0.007031506	down	0.722558075
At5g55290	V-Type ATPase	0.015640091	down	0.73172802	0.29507628	down	0.912475197	0.004809665	down	0.603834871
At1g15700	V-Type ATPase				0.21354029	down	0.818334088			

Supplementary Table S4 Transporter-related genes in response to As stress (continued)

Functional categories	In genome	On array	Col-0 with100 uM As/Control			Ws-2 with 100 uM As/Control			Col-0 with 200 uM As/Control		
			Detected	Induced	Repressed	Detected	Induced	Repressed	Detected	Induced	Repressed
ABC transporters	124	117	41	6	3	41	8	1	41	10	4
V-Type ATPase	22	18	15	0	0	16	0	0	15	1	0
aquaporin	40	40	16	0	5	16	0	2	16	0	9
MATE	57	56	19	3	1	19	4	0	19	4	4
LeOPT1	52	51	19	1	8	17	1	6	19	1	12
Antiporters	79	75	24	4	3	24	3	1	26	5	6
sugar transporter	53	52	21	0	2	20	2	1	22	0	7

Supplementary Table S5 Hormone-related genes in response to As stress

AGI Locus	Gene Family	Group	Col-100 Corrected p-value	Regulation	Col-100 FC Absolute	Ws-100 Corrected p-value	Regulation	Ws-100 FC Absolute	Col-200 corrected p-value	Regulation	Col-200 FC Absolute
At1g16540	ABA biosynthesis	ABA	0.05029753	up	2.4151847	0.048087057	up	1.8408856	0.00910621	up	3.0306141
At1g52340	ABA biosynthesis	ABA	0.00492641	up	1.3779511	0.58181006	up	1.0532119	0.002299141	up	1.3714616
At3g43600	ABA biosynthesis	ABA	0.011261709	down	0.682241932	0.6219716	down	0.945403961	0.007760637	down	0.547329587
At5g67030	ABA biosynthesis	ABA	0.4711093	down	0.889950343	0.11452172	down	0.775040713	0.33206424	down	0.86082537
At2g27150	ABA biosynthesis	ABA	0.470574	down	0.873795604	N.D.		N.D.	0.10263796	down	0.655076614
At4g23400	ABA response down	ABA	0.06117905	down	0.50953986	0.058438372	down	0.625386254	0.007411611	down	0.296179422
At1g18890	ABA signaling	ABA	0.04146793	up	1.6788054	0.27467182	up	1.2044518	0.033225384	up	1.542133
At1g69270	ABA signaling	ABA	0.43139648	up	1.125102	0.099653035	up	1.3755283	0.03469252	up	1.4576324
At2g26300	ABA signaling	ABA	0.004752152	up	1.6189718	0.003559839	up	1.8088921	0.001215955	up	2.3724775
At4g17615	ABA signaling	ABA	0.025101064	up	1.7496245	0.007676405	up	1.5202327	0.006716564	up	3.2451632
At4g33950	ABA signaling	ABA	0.03911629	up	3.1918435	0.3569852	up	1.1374819	0.00081214	up	5.824634
At1g74740	ABA signaling	ABA	0.9645376	down	0.994830661	0.5758577	up	1.0904814	0.9453545	up	1.0072519
At1g64060	ABA signaling	ABA	0.2056929	down	0.872765916	0.25097507	up	1.1342543	0.005658592	down	0.611514874
At4g34000	ABA signaling	ABA	0.07423415	down	0.709121134	0.12126096	up	1.1439152	0.011745628	down	0.646154534
At1g49720	ABA signaling	ABA	0.13811272	down	0.694642852	0.6556096	down	0.965144296	0.009410014	down	0.495146941
At2g17800	ABA signaling	ABA	0.25921035	down	0.918345072	0.039118987	down	0.768267421	0.03173529	down	0.765566298
At3g19290	ABA signaling	ABA	0.69340616	down	0.94274902	0.94597065	down	0.988797908	0.001861962	down	0.64823934
At3g56860	ABA signaling	ABA	0.123916775	down	0.875117266	0.08273333	down	0.860756905	0.014638152	down	0.692497037
At2g26980	ABA signaling, CY response up	ABA	0.020050004	down	0.48608336	0.09632188	down	0.716915771	0.00170818	down	0.352881473
At1g69700	ABA signaling?	ABA	0.018315261	up	1.5162084	0.5036621	up	1.0588971	0.03268449	up	1.4607244
At5g13200	ABA signaling?	ABA	0.07003081	up	2.52697	0.037199095	up	1.955931	0.012037558	up	4.387305
At5g42560	ABA signaling?	ABA	0.05160944	up	1.4252784	0.17539644	down	0.844970264	0.16026857	up	1.1884406
At4g24960	ABA signaling?	ABA	0.27447295	up	1.1840432	0.010057126	up	1.7705814	0.8416987	down	0.969882811
At1g74520	ABA signaling?	ABA	0.015094969	down	0.504676484	0.043980394	down	0.707606165	0.007165048	down	0.426219779
At5g13630	Abscisic acid (ABA) receptor	ABA	0.32255948	up	1.3011258	0.06281277	down	0.607524039	0.34919223	up	1.31839
At1g72770	negative regulation of ABA signaling	ABA	0.031716086	up	1.6951458	0.038224865	up	1.2003968	0.001759288	up	2.1867292
At4g26080	negative regulation of ABA signaling	ABA	0.024590416	up	2.0437226	0.009211053	up	1.9928635	0.004634871	up	2.7558897
At2g39550	negative regulation of ABA signaling	ABA	0.6638712	down	0.983421384	0.77819157	up	1.0286187	0.07825631	up	1.1166614
At5g40280	negative regulation of ABA signaling	ABA	0.025883554	up	1.1340384	0.061819937	down	0.819320562	0.031952076	up	1.1415162
At1g17550	negative regulation of ABA signaling	ABA	0.77507657	down	0.959382894	0.97614807	up	1.0037894	0.9701633	down	0.99524789
At2g22430	negative regulation of ABA signaling	ABA	0.34597304	down	0.906161208	0.3730695	up	1.0964649	0.00491667	down	0.552699359
At5g01810	negative regulation of ABA signaling	ABA	0.8443792	down	0.967523993	0.0784847	up	1.1000541	0.3173745	down	0.852754461
At3g11410	negative regulation of ABA signaling	ABA	0.014511883	down	0.537927463	0.32342663	down	0.843659728	0.001986831	down	0.464937548
At3g59380	negative regulation of ABA signaling	ABA	0.12492131	down	0.872712065	0.8037693	down	0.981577363	0.3991736	down	0.935611322
At3g48040	negative regulation of ABA signaling	ABA	N.D.		N.D.	0.15378568	down	0.771906256	N.D.		N.D.
At5g57050	negative regulation of ABA signaling	ABA	N.D.		N.D.	N.D.		N.D.	0.000975366	up	14.75806
At1g25490	positive regulation of ABA signaling	ABA	0.13781546	up	1.1108752	0.26606202	down	0.925306741	0.8013261	down	0.989590791
At5g03280	positive regulation of ABA signaling, ET signaling	ABA	0.20024967	down	0.872055352	0.023530344	down	0.684265988	0.033781596	down	0.695711896
At3g30180	BR biosynthesis	BR	0.013319875	up	1.5106839	0.4610432	down	0.914049712	0.008256495	up	1.4433881
At5g16010	BR biosynthesis	BR	0.05342799	up	1.4294555	0.15860233	up	1.3193555	0.42996737	down	0.905872919
At3g55360	BR biosynthesis	BR	0.554218	down	0.944999159	0.039218377	up	1.3639644	0.011695122	down	0.705698274
At2g38050	BR biosynthesis	BR	0.028886646	down	0.636748528	0.11557215	down	0.777813281	0.003337218	down	0.408622023
At3g50660	BR biosynthesis, BR downregulated	BR	0.015969412	down	0.352866369	0.014154107	down	0.282994482	0.005204357	down	0.248700435
At4g36380	BR biosynthesis, BR downregulated	BR	0.025604112	down	0.29862381	0.006060074	down	0.255308404	0.004154107	down	0.158295467
At5g05690	BR biosynthesis, BR downregulated	BR	0.9288986	down	0.992944731	0.42686012	down	0.909331055	0.31998065	down	0.941933031
At2g30070	BR downregulated	BR	N.D.		N.D.	N.D.		N.D.	0.014215199	up	2.6218162
At1g06180	BR downregulated	BR	0.052699264	up	2.170592	0.28327307	up	1.2629335	0.009472011	up	2.9043186
At1g18570	BR downregulated	BR	0.00977634	up	11.894185	0.019853469	up	2.5624964	0.003274166	up	9.87029
At1g27730	BR downregulated	BR	0.28777975	up	1.2282759	0.16600026	up	1.2187562	0.7631018	up	1.050481
At2g40540	BR downregulated	BR	0.04876648	up	1.4283993	0.061943416	up	1.3247063	0.23645681	up	1.312355
At3g09010	BR downregulated	BR	0.049289647	up	4.4277954	0.00523089	up	2.5668051	0.006353002	up	11.457114
At2g04160	BR downregulated	BR	0.16300477	down	0.77415559	0.45830047	up	1.028787	0.016982565	down	0.767567745
At1g22360	BR downregulated	BR	0.49782962	down	0.956691257	0.61704224	down	0.944107157	0.002344732	down	0.666096488
At1g77760	BR downregulated	BR	0.010167131	down	0.255452206	0.00869683	down	0.430419391	0.001952861	down	0.206120119
At4g14130	BR downregulated	BR	0.013878533	down	0.64263123	0.037289992	down	0.653196023	0.001446053	down	0.377327023
At4g36220	BR downregulated	BR	0.007224579	down	0.042861774	0.008602032	down	0.280190789	0.002275986	down	0.042299916
At5g65310	BR downregulated	BR	0.11176211	down	0.64320673	0.043392863	down	0.767060048	0.14620435	down	0.698394642
At5g47220	BR downregulated, ET signaling	BR	N.D.		N.D.	N.D.		N.D.	0.031022115	up	3.3936985
At4g30270	BR downregulated, GA inducible, SAG	BR	0.034999218	down	0.446602687	0.045148496	down	0.573452301	0.024363363	down	0.514194202
At5g62000	BR downregulated, IAA signaling	BR	0.09335139	down	0.658720955	0.8812009	up	1.040662	0.007380267	down	0.422588273
At5g62000	BR downregulated, IAA signaling	BR	0.04930014	down	0.582646447	0.8812009	up	1.040662	0.007380267	down	0.422588273
At5g62000	BR downregulated, IAA signaling	BR	0.09335139	down	0.658720955	0.8812009	up	1.040662	0.008719094	down	0.558802165
At5g62000	BR downregulated, IAA signaling	BR	0.04930014	down	0.582646447	0.8812009	up	1.040662	0.008719094	down	0.558802165
At5g62000	BR downregulated, IAA signaling	BR	0.09335139	down	0.658720955	0.042803843	down	0.761180524	0.007380267	down	0.422588273
At5g62000	BR downregulated, IAA signaling	BR	0.04930014	down	0.582646447	0.042803843	down	0.761180524	0.007380267	down	0.422588273
At5g62000	BR downregulated, IAA signaling	BR	0.09335139	down	0.658720955	0.042803843	down	0.761180524	0.008719094	down	0.558802165
At5g62000	BR downregulated, IAA signaling	BR	0.04930014	down	0.582646447	0.042803843	down	0.761180524	0.008719094	down	0.558802165
At3g12500	BR downregulated, JA and ET response	BR	0.040810503	down	0.131954363	0.5589554	down	0.882838113	0.007151266	down	0.08875878
At2g26710	BR inactivation, BR upregulated	BR	0.06829043	down	0.637787805	N.D.		N.D.	0.013226045	down	0.513393776
At4g18710	BR signaling	BR	0.04945017	up	1.1924531	0.004979775	up	1.3499936	0.09365631	up	1.2209078
At4g30610	BR signaling	BR	0.008479659	down	0.385706891	N.D.		N.D.	0.006871474	down	0.423279742
At4g33430	BR signaling	BR	0.030578941	up	1.2613764	0.017437948	up	1.26714	0.002677135	up	1.4089502
At2g29400	BR signaling?	BR	0.040974054	up	1.70517	0.038061347	up	1.2709068	0.015584613	up	1.8415048
At1g10430	BR signaling?	BR	0.28975144	up	1.1343479	0.06852427	down	0.825414649	0.34845197	down	0.901844994
At1g01120	BR upregulated	BR	0.03441331	up	2.5137048	0.007475819	up	2.5622244	0.021212697	up	2.044808
At2g36220	BR upregulated	BR	0.006172375	up	1.9598743	0.077113725	up	1.4426103	0.001084539	up	2.473484
At3g30775	BR upregulated	BR	0.24834518	up	1.2502674	0.005994618	up	4.61892	0.028741183	up	1.7564306
At4g01950	BR upregulated	BR	0.38417354	up	1.2773497	0.01137017	up	1.7673346	0.15794067	up	1.4410468
At4g20780	BR upregulated	BR	0.00993676	up	3.3239126	0.018669752	up	1.3612856	0.003607981	up	3.6910787
At4g25810	BR upregulated	BR	0.60505456	up	1.0754874	0.13413322	up	2.0221298	0.017629668	up	1.7610266
At5g02490	BR upregulated	BR	0.032204952	up	2.0100696	0.015866674	up	2.5220516	0.003847103	up	4.091026
At5g51460	BR upregulated	BR	0.01168374	up	2.2079206	0.028123936	up	2.0554028	0.002283728	up	3.326084
At1g20440	BR upregulated	BR	0.9153725	down	0.986512305	0.014278783	up	1.4727645	0.5777249	up	1.055274
At5g13870	BR upregulated	BR	0.6463953	up	1.1153363	0.14907573	down	0.82982022	0.96070707	up	1.0112094
At5g37770	BR upregulated	BR	0.57046604	up	1.0775476	0.4863422	up	1.0871131	0.055493463	down	0.839998582
At1g67730	BR upregulated	BR	0.24421969	down	0.87955946	0.009211053	up	1.2289037	0.001990669	down	0.703888923
At4g08950	BR upregulated	BR	0.23419335	down	0.803951324	0.27501	up	1.3296273	0.9628849	down	0.99544643
At4g09460	BR upregulated	BR	0.06375937	down	0.701877079	0.21604915	up	1.2269533	0.028949695	down	0.710784289
At5g47370	BR upregulated	BR	0.13411227	down	0.803536912	0.02892003	up	1.1970581	0.046992697	down	0.747900867
At5g57560	BR upregulated	BR	0.0825675	down	0.624543888	0.077113725	up	1.6488928	0.18242447	down	0.797804569
At2g14900	BR upregulated	BR	0.5386429	up	1.1054871	0.04681499	down	0.854573034	0.9882252	down	0.998399167
At1g04610	BR upregulated	BR	0.00664473	down	0.504785365	0.28461397	down	0.86530356	0.000878635	down	0.500726028
At1g24625	BR upregulated	BR	0.032216173	down	0.436591807	0.006211679	down	0.72865691	0.001801345	down	0.223524726
At1g33240	BR upregulated	BR	0.14995839	down	0.702714862	0.19076446	down	0.893962064	0.003295129	down	0.250820371
At4g00360	BR upregulated	BR	N.D.		N.D.	0.9522185	up	1.039942	N.D.		N.D.
At1g65310	BR upregulated	BR	0.01179656	down	0.542379088	0.2901339	down	0.742153802	0.003143261	down	0.364907169
At2g31730	BR upregulated	BR	0.027359206	down	0.301108365	0.001089257	down	0.546114659	0.006015687	down	0.249119766
At2g43290	BR upregulated	BR	0.04598747	down	0.527813808	0.09896758	down	0.770749383	0.001133895	down	0.162888973
At3g12610	BR upregulated	BR	0.015147975	down	0.825943548	0.19303623	down	0.925081361	0.037259568	down	0.725436608
At3g16240	BR upregulated	BR	0.024953123	down	0.514101205	0.01467628	down	0.681296417	0.003133086	down	0.250915503
At4g34160	BR upregulated	BR	0.05489866	down	0.646848396	0.4741343	down	0.947875475	0.002632539	down	0.387567261
At2g32560	BR upregulated	BR	0.2821119	up	1.6269716	0.004259504	up	2.9365735	N.D.		N.D.
At5g10430	BR upregulated	BR	0.017405126	down	0.641393291	0.22940886	down	0.862495603	0.004048255	down	0.372958801
At1g64780	BR upregulated, CY response down	BR	0.012340221	down	0.340789513	0.7760804	down	0.942329438	0.001167618	down	0.101064747
At1g04240	BR upregulated, CY response up, IAA signaling	BR	0.028689183	down	0.4678743	N.D.		N.D.	0.027633926	down	0.430179464
At3g13380	Brassinosteroid (BR) receptor	BR	N.D.		N.D.	0.005686936	up	1.7895039	N.D.		N.D.
At3g15540	BR upregulated, IAA signaling	BR	0.9671054	down	0.990927465	0.6675394	up	1.0818989	0.9185016	up	1.02052
At4g39400	Brassinosteroid (BR) receptor	BR	0.19000688	down	0.795614003	0.8078459	down	0.975581393	0.008768661	down	0.702821145
At1g50430	sterol/BR biosynthesis	BR	0.76513565	down	0.95656085	0.049034085	down	0.947315341	0.31111136	down	0.903220224
At3g52940	sterol/BR biosynthesis	BR	0.029937746	down	0.721988663	0.018638603	down	0.756432341	0.003033525	down	0.549669662
At3g02580	sterol/BR biosynthesis, BR downregulated	BR	0.013868229	down	0.72997299	0.7215251	down	0.970943355	0.014755006	down	0.6656274
At1g67110	CY biosynthesis	CY	0.007278231	down	0.127081475	0.004852493	down	0.089132518	0.001541871	down	0.045684805
At3g63110	CY biosynthesis (IPT3)	CY	0.005349687	down	0.135868568	N.D.		N.D.	0.001172532	down	0.099130448
At5g20040	CY biosynthesis (IPT9)	CY	0.17270336	up	1.1359004	0.122708775	down	0.70683092	N.D.		N.D.
At5g19040	CY biosynthesis (IPT5)	CY	0.013249892	down	0.234609367	N.D.		N.D.	0.00513253	down	0.14434351
At1g22400	CY conjugation	CY	0.31062654	up	1.3861356	N.D.		N.D.	0.09903833	up	2.1441154
At5g58600	CY conjugation	CY	0.16244292	up	1.3624743	0.396423	down	0.74607323	0.5403242	up	1.1096154
At3g13790	CY cross talk	CY	0.17242496	up	1.1652873	0.826461	down	0.988088006	0.19781068	down	0.924945569
At1g12240	CY cross talk	CY	0.05965756	down	0.691508032	0.060600605	down	0.601966251	0.010100544	down	0.565829575
At1g62660	CY cross talk	CY	0.032539453	down	0.721126735	0.02024825	down	0.706522745	0.008068384	down	0.732531075
At5g11920	CY cross talk, SA response (nahG)	CY	0.48022637	up	1.2485923	0.4642235	down	0.893189856	0.97123134	down	0.987625156
At2g17820	CY receptor (AHK1)	CY	0.007119681	down	0.089766397	0.008135078	down	0.242742644	0.000897589	down	0.019035078
At5g35750	CY receptor (AHK2)	CY	0.007445758	down	0.660661649	0.010166872	down	0.58008769	0.001325433	down	0.412119036
At1g27320	CY receptor (AHK3)	CY	0.81782955	up	1.0516682	0.07584443	up	1.273648	0.7564094	down	0.947557883
At2g01830	CY receptor (CRE1/AHK4)	CY	0.06089215	down	0.76842788	0.039542343	down	0.777236364	0.008919309	down	0.456892073
At2g21060	CY response down	CY	0.009623421	up	1.7359631	0.807219	down	0.972461448	0.004908105	up	1.811407
At1g04220	CY response down	CY	0.64567614	up	1.3267518	0.004227638	up	3.0387132	0.09262513	down	0.321872049
At4g14940	CY response down	CY	0.027659256	down	0.634101497	0.39954135	up	1.0577794	0.006631438	down	0.454187885
At4g11050	CY response down	CY	0.028886646	down	0.384654827	0.025188968	down	0.585995035	0.009219267	down	0.322718029
At1g64780	CY response down, BR upregulated	CY	0.012340221	down	0.340789513	0.7760804	down	0.942329438	0.001167618	down	0.101064747
At2g22860	CY response up	CY	0.022322213	up	2.8460608	0.107750125	up	2.1268568	0.039705437	up	1.949357

At2g47270	CY response up	CY	0.0770736	up	1.820584	0.9690387	up	1.0079942	0.017456276	up	2.3384502
At3g15770	CY response up	CY	0.010307205	up	2.9686725	0.018233245	up	1.8759767	0.001225199	up	5.36305
At3g51500	CY response up	CY	N.D.		N.D.	N.D.		N.D.	0.00397403	up	2.853111
At3g16530	CY response up	CY	0.36576468	up	1.2827762	0.7274738	up	1.0645099	0.11306464	up	1.5699003
At3g59900	CY response up	CY	0.06748582	up	3.0423748	0.094614536	up	1.4800571	0.007053774	up	8.176183
At4g37790	CY response up	CY	0.035043217	up	2.9739542	0.31187212	up	1.2033141	0.011953034	up	3.6558454
At5g13330	CY response up	CY	0.09469937	up	3.1183615	0.03807527	up	2.0117412	0.048630085	up	3.5386696
At1g53180	CY response up	CY	0.8618144	down	0.960785631	0.46917298	up	1.0550166	0.08428818	up	1.2570604
At1g60010	CY response up	CY	0.4171751	up	1.3389238	0.27910066	down	0.798852592	0.19781068	up	1.5158288
At1g69530	CY response up	CY	0.4756573	up	1.1660398	0.63510823	down	0.925039687	0.0495363	up	1.7697996
At1g13740	CY response up	CY	0.40829352	up	1.150965	N.D.		N.D.	0.2952527	up	1.164124
At1g21830	CY response up	CY	0.1253861	up	1.6856793	N.D.		N.D.	0.20174898	up	1.4725507
At5g20230	CY response up	CY	0.004063893	up	10.959043	N.D.		N.D.	0.001408073	up	14.819119
At2g03730	CY response up	CY	0.4292599	up	1.0713729	0.9273815	down	0.991003374	0.011799698	up	1.506632
At3g61060	CY response up	CY	0.11206044	down	0.561196642	0.22828113	up	1.2856182	0.03607358	down	0.36395042
At2g39705	CY response up	CY	0.7896216	up	1.0832832	0.5775432	down	0.866034283	0.33513582	down	0.779025638
At3g51660	CY response up	CY	0.15812062	up	1.5147018	0.11496089	down	0.757881781	0.91036296	down	0.975101942
At1g03850	CY response up	CY	0.34404483	down	0.736158508	0.029133752	down	0.374499351	0.16761093	down	0.667973445
At1g13300	CY response up	CY	0.002960329	down	0.149288268	0.0060313	down	0.244729505	0.000471695	down	0.071003847
At1g49500	CY response up	CY	0.010088196	down	0.162262274	0.004813203	down	0.102901546	0.001380261	down	0.047481834
At1g68490	CY response up	CY	0.08798506	down	0.735640572	0.19023193	down	0.899055407	0.003009056	down	0.301287628
At1g75440	CY response up	CY	0.019543547	down	0.479152873	0.04248663	down	0.582872762	0.00205078	down	0.23176418
At1g76410	CY response up	CY	0.037369266	down	0.555082071	0.018938925	down	0.21702441	0.011352806	down	0.138416226
At2g40230	CY response up	CY	0.017765895	down	0.437879214	0.005143304	down	0.182344045	0.002697748	down	0.242721545
At4g09760	CY response up	CY	0.01630794	down	0.257624951	0.022267781	down	0.542619006	0.004520424	down	0.260655574
At4g29720	CY response up	CY	0.008399133	down	0.405810887	0.012977538	down	0.283124265	0.002632539	down	0.184782121
At4g27410	CY response up	CY	0.019029113	down	0.502633094	N.D.		N.D.	0.018592509	down	0.461060278
At1g10470	CY response up (ARR4)	CY	0.577286	down	0.872274122	0.012741539	down	0.596929074	0.02543755	down	0.470929942
At3g48100	CY response up (ARR5)	CY	0.018459689	down	0.412293232	0.012853701	down	0.248763459	0.001178052	down	0.113010849
At5g62920	CY response up (ARR6)	CY	0.068019	down	0.395110773	0.008131621	down	0.375701392	0.001446053	down	0.130099709
At1g19050	CY response up (ARR7)	CY	0.122159876	down	0.633547747	N.D.		N.D.	0.118035346	down	0.461006949
At3g57040	CY response up (ARR9)	CY	0.13801605	down	0.56240863	0.005160912	down	0.28812477	0.019900344	down	0.363224665
At2g26980	CY response up, ABA signaling	CY	0.020050004	down	0.48608336	0.09632188	down	0.716915771	0.00170818	down	0.352881473
At4g04620	CY response up, autophagy	CY	0.012877372	up	2.7389255	0.023593634	up	1.8619728	0.002371014	up	3.6600225
At1g62040	CY response up, autophagy	CY	0.89013445	up	1.0172781	0.055245418	up	1.2810491	0.36204007	down	0.905317136
At4g34750	CY response up, IAA signaling?	CY	N.D.		N.D.	0.10173202	down	0.699686603	N.D.		N.D.
At1g04240	CY response up, IAA signaling, BR upregulated	CY	0.028689183	down	0.4678743	N.D.		N.D.	0.027633926	down	0.430179464
At1g64520	CY signaling	CY	0.21973316	down	0.899612267	0.17315581	up	1.1676774	0.034293808	down	0.869596372
At3g29350	CY signaling	CY	0.15596037	down	0.880728179	0.18819998	up	1.1494955	0.08321898	down	0.87388273
At3g21510	CY signaling	CY	0.63190985	down	0.870634508	0.2157748	down	0.882677896	0.063124016	down	0.699885275
At5g62120	CY signaling (ARR23)	CY									
At5g10720	CY signaling (AHK5)	CY	0.95623535	down	0.993248493	0.86832356	down	0.980203612	0.08576854	down	0.787926818
At3g16857	CY signaling (ARR1)	CY	0.21525566	up	1.1266071	0.36888504	up	1.113071	0.6009607	up	1.0513378
At3g16857	CY signaling (ARR1)	CY	0.18693098	up	1.1451967	0.36888504	up	1.113071	0.6009607	up	1.0513378
At3g16857	CY signaling (ARR1)	CY	0.21525566	up	1.1266071	0.25264522	up	1.1720136	0.026204452	up	1.2791226
At3g16857	CY signaling (ARR1)	CY	0.18693098	up	1.1451967	0.25264522	up	1.1720136	0.026204452	up	1.2791226
At3g16857	CY signaling (ARR1)	CY	0.21525566	up	1.1266071	0.25264522	up	1.1720136	0.6009607	up	1.0513378
At3g16857	CY signaling (ARR1)	CY	0.18693098	up	1.1451967	0.25264522	up	1.1720136	0.6009607	up	1.0513378
At3g16857	CY signaling (ARR1)	CY	0.21525566	up	1.1266071	0.36888504	up	1.113071	0.026204452	up	1.2791226
At3g16857	CY signaling (ARR1)	CY	0.18693098	up	1.1451967	0.36888504	up	1.113071	0.026204452	up	1.2791226
At4g31920	CY signaling (ARR10)	CY	0.03261617	down	0.579270906	0.18951865	down	0.896476989	0.003166563	down	0.356339375
At2g25180	CY signaling (ARR12)	CY	0.30609405	down	0.948549355	0.6118014	up	1.0447695	0.030471845	down	0.799115411
At4g16110	CY signaling (ARR2)	CY	0.041056868	up	1.3438224	0.13941184	up	1.1846315	0.099796295	up	1.1460578
At1g59940	CY signaling (ARR3)	CY	0.22497697	up	1.9278741	0.043506384	down	0.355900307	0.32789215	down	0.656818376
At2g41310	CY signaling (ARR8)	CY	0.0736935	down	0.573346333	0.02774886	down	0.508342691	0.026776718	down	0.361097207
At4g37770	ET biosynthesis	ET									
At1g19770	CY transport?	CY	0.042158883	up	1.4425942	0.31239355	down	0.86178168	0.01843849	up	1.5907221
At1g30840	CY transport?	CY	0.026728887	down	0.269675148	0.032110117	down	0.283242678	0.004940283	down	0.072877172
At1g44750	CY transport?	CY	0.023249654	down	0.709994961	0.044967987	down	0.73143665	0.003037738	down	0.495475368
At4g26200	ET biosynthesis	ET							0.005809662	up	4.9973574
At1g02500	ET biosynthesis	ET	0.11374277	up	1.1978087	0.014678935	up	1.1541725	0.7474277	up	1.0155729
At1g05010	ET biosynthesis	ET	0.06520963	up	1.32648	0.10639275	up	1.4507828	0.000686013	up	2.2634623
At1g62960	ET biosynthesis	ET	0.061690945	up	1.4831822	0.043392863	up	1.6582749	0.21026368	up	1.2180284
At4g11280	ET biosynthesis	ET	0.008697626	up	4.476119	0.030901639	up	1.6614738	0.001749253	up	6.923268
At5g38530	ET biosynthesis	ET	0.041677136	up	1.4955018	0.012713693	up	1.3308188	0.005911307	up	1.8076879
At3g10050	ET biosynthesis	ET	0.7158872	up	1.0565572	0.10958507	up	1.2977126	0.86976945	down	0.979899709
At1g55880	ET biosynthesis	ET	0.90673065	up	1.0124692	0.5239229	down	0.97820357	0.27047026	down	0.928252401
At1g48420	ET biosynthesis	ET	0.1007789	down	0.778107903	0.18003419	down	0.887667852	0.055895414	down	0.746373595
At1g73730	ET signaling	ET	0.034478515	up	2.1666145	0.004258467	up	2.1580987	0.008443	up	3.0311124
At2g27050	ET signaling	ET	0.07756122	up	1.3203562	0.01851467	up	1.3555582	0.002677375	up	1.6370925
At3g24500	ET signaling	ET	0.015863087	up	16.806759	0.006460856	up	5.1495824	0.004667494	up	26.04942
At4g17490	ET signaling	ET	0.006722895	up	7.028858	0.08813379	up	3.6148252	0.001479318	up	11.462476
At4g17500	ET signaling	ET	0.015089777	up	3.6043816	0.001810002	up	1.4761107	0.003059956	up	5.815963
At5g47230	ET signaling	ET	0.032964908	up	2.3349628	0.009807889	up	1.7954566	0.00330901	up	2.874979
At3g20310	ET signaling	ET	0.7479827	down	0.91391171	0.55620885	up	1.0154511	0.8194998	up	1.0517133
At1g53170	ET signaling	ET	0.04487568	up	2.4434264				0.033266027	up	2.2301989
At2g31230	ET signaling	ET	0.005731739	up	9.990343				0.000889151	up	17.97217
At3g16770	ET signaling	ET	0.191743	up	1.4339435	0.29196712	down	0.811813444	0.32333913	up	1.3851814
At3g46060	ET signaling	ET	0.2536489	up	1.1324453	0.332509	up	1.0802703	0.5768108	down	0.977055608
At5g44790	ET signaling	ET	0.057208467	down	0.872847656	0.39057162	up	1.0861943	0.04083188	down	0.842317492
At2g44840	ET signaling	ET	0.98983175	up	1.0057124	0.04500876	down	0.534861497	0.3217889	down	0.661734976
At1g72360	ET signaling	ET	0.008263622	down	0.34162644	0.009099189	down	0.302015239	0.000679271	down	0.196362491
At3g20770	ET signaling	ET	0.06822979	down	0.897164172	0.31239355	down	0.925322239	0.003277496	down	0.715923133
At5g07580	ET signaling	ET	0.002927062	down	0.321224147	0.004574726	down	0.433767969	0.000471695	down	0.13689224
At5g47220	ET signaling, BR downregulated	ET							0.031022115	up	3.3936985
At3g01420	ET signaling, JA and ET response, JA synthesis	ET	0.4412959	down	0.661933796	0.01033256	up	2.4722967	0.09588655	down	0.443461124
At1g32230	ET signaling, JA signaling	ET	0.14647461	up	1.2123431	0.020803593	up	1.5568339	0.038783308	up	1.2059546
At5g03280	ET signaling, positive regulation of ABA signaling	ET	0.20024967	down	0.872055352	0.023530344	down	0.684265988	0.033781596	down	0.695711896
At1g04310	Ethylene receptor	ET							0.000932534	up	3.7329938
At1g66340	Ethylene receptor	ET	0.8986087	up	1.009808	0.06852427	up	1.1596117	0.8672756	up	1.0265105
At3g23150	Ethylene receptor	ET	0.13801605	up	1.7035497	0.24456906	down	0.903923425	0.55394924	up	1.2002198
At2g40940	Ethylene receptor	ET	0.81029415	up	1.0279979	0.027508326	down	0.821253448	0.03967376	down	0.859369656
At3g04580	Ethylene receptor	ET	0.62203777	up	1.0502914	0.79916346	down	0.98436862	0.1575016	down	0.853457506
At3g60140	JA and ET response	ET	0.000164516	up	134.09415	0.000612937	up	54.32797	1.24229E-05	up	298.64627
At4g16260	JA and ET response	ET	0.022255326	down	0.24008987	0.47382528	up	1.0876298	0.003033525	down	0.152729546
At4g37430	JA and ET response	ET	0.00664473	down	0.395896423	0.16749284	up	1.2660105	0.008550385	down	0.269020293
At1g11190	JA and ET response	ET				0.11717899	up	1.4880642			
At4g11650	JA and ET response	ET	0.013371039	down	0.081151106	0.81325257	down	0.906032719	0.020340016	down	0.098855559
At3g12500	JA and ET response, BR downregulated	ET	0.040810503	down	0.131954363	0.5589554	down	0.882838113	0.007151266	down	0.08875878
At3g51770	negative regulation of ET biosynthesis	ET	0.34155965	up	1.1481323	0.4603627	up	1.0512402	0.049869243	down	0.734921577
At4g02680	negative regulation of ET biosynthesis?	ET	0.018338196	down	0.612425751	0.1676469	down	0.800714686	0.004226876	down	0.45691915
At1g50640	negative regulation of ET signaling	ET	0.020354448	up	2.0013468	0.17882377	up	1.4143676	0.000878635	up	2.63416
At5g03730	negative regulation of ET signaling	ET	0.028824734	up	1.6148529	0.35398868	up	1.1096628	0.005743616	up	1.8544548
At5g25350	negative regulation of ET signaling	ET	0.33599833	up	1.1303943	0.9049837	up	1.0119004	0.01815654	up	1.377016
At3g15210	negative regulation of ET signaling	ET	0.62711954	up	1.0631994	0.24445501	down	0.819533079	0.28728223	down	0.903697152
At2g25490	negative regulation of ET signaling	ET	0.95189357	down	0.993910707	0.10042126	down	0.834374563	0.005755471	down	0.647900488
At3g04580	negative regulation of ET signaling (EIN4)	ET	0.62203777	up	1.0502914	0.79916346	down	0.98436862	0.1575016	down	0.853457506
At2g40940	negative regulation of ET signaling (ERS1)	ET	0.81029415	up	1.0279979	0.027508326	down	0.821253448	0.03967376	down	0.859369656
At1g04310	negative regulation of ET signaling (ERS2)	ET							0.000932534	up	3.7329938
At1g66340	negative regulation of ET signaling (ETR1)	ET	0.8986087	up	1.009808	0.06852427	up	1.1596117	0.8672756	up	1.0265105
At3g23150	negative regulation of ET signaling (ETR2)	ET	0.13801605	up	1.7035497	0.24456906	down	0.903923425	0.55394924	up	1.2002198
At5g25900	GA biosynthesis	GA	0.5684776	up	1.0756724	0.27016312	down	0.889005843	0.02470655	up	1.4913934
At5g51310	GA biosynthesis?	GA				0.3484621	up	1.3045228			
At1g79460	GA biosynthesis	GA	0.025234912	up	1.7866473				0.008263825	up	1.8489306
At1g15550	GA biosynthesis	GA	0.006580223	down	0.318992443	0.024614355	down	0.481295509	0.00105364	down	0.173467007
At2g36830	GA inducible	GA	0.020838594	down	0.614641863	0.0640298	down	0.824840841	0.002611483	down	0.50008639
At4g30270	GA inducible, BR downregulated, SAG	GA	0.034999218	down	0.446602687	0.045148496	down	0.573452301	0.024363363	down	0.514194202
At5g15230	GA signaling	GA	0.06829861	up	1.2497554	0.4843318	down	0.942276873	0.62868124	down	0.93333026
At1g14920	GA signaling	GA	0.19652511	down	0.814420189	0.750834	down	0.935087358	0.10639055	down	0.740866688
At2g01570	GA signaling	GA	0.07315572	down	0.547037543	0.052634854	down	0.812392231	0.004908105	down	0.491126812
At5g27320	Gibberellin receptor	GA				0.19143608	down	0.767585185			
At3g11540	GA signaling	GA	0.07132834	down	0.835783432	0.56656283	down	0.927925261	0.004062924	down	0.697994912
At3g11440	GA signaling?	GA	0.020327728	up	2.6665947				0.002668794	up	4.096467
At3g63010	Gibberellin receptor	GA	0.73855597	up	1.0664785	0.040161762	up	1.4872165	0.005924083	up	1.3576039
At3g05120	Gibberellin receptor	GA	0.104500756	down	0.714766344	0.052989222	down	0.63948477	0.004691657	down	0.381573612
At3g62980	Auxin receptor	IAA	0.07813288	up	1.2471148	0.13933222	up	1.217895	0.05935946	up	1.3114295
At3g26810	Auxin receptor	IAA	0.035043217	down	0.845001251	0.6747457	up	1.0204166	0.001562901	down	0.646161924
At4g03190	Auxin receptor	IAA	0.58104914	up	1.0222214	0.06506192	down	0.84245466	0.3628119	down	0.919110635
At1g12820	Auxin receptor	IAA	0.03811651	down	0.610892283	0.013695798	down	0.750942602	0.002455834	down	0.449162043
At3g54640	IAA biosynthesis	IAA	0.00708164	up	1.720325	0.032191306	down	0.7934654	0.001610554	up	1.6835833
At3g44320	IAA biosynthesis	IAA	0.8129272	up	1.0213798	0.99647075	down	0.999658117	0.39979854	down	0.916245202

At5g20960	IAA biosynthesis	IAA	0.90598136	up	1.0082334	0.840895	down	0.987390529	0.34389722	down	0.914907475
At5g57420	IAA signaling	IAA									
At2g20610	IAA biosynthesis	IAA	0.00870088	down	0.593250881	0.004294924	down	0.331354649	0.000708953	down	0.274675901
At2g22330	IAA biosynthesis	IAA	0.007121542	down	0.175060803	0.00437724	down	0.050462522	0.001812016	down	0.13408149
At4g31500	IAA biosynthesis	IAA	0.0186746	down	0.647352097	0.004857321	down	0.235483369	0.00088781	down	0.218506232
At4g39950	IAA biosynthesis	IAA	0.004517191	down	0.291789748	0.005921167	down	0.1012442	0.001380261	down	0.143087037
At4g02610	IAA biosynthesis?	IAA	0.81844383	down	0.985898399	0.12914571	down	0.788403033	0.3672199	down	0.935818393
At4g08790	IAA biosynthesis?	IAA	0.272708	down	0.83101425	0.1971347	down	0.844177568	0.59595424	down	0.896620467
At5g15600	IAA biosynthesis?	IAA	0.96087414	down	0.991904669	0.018360244	down	0.776158401	0.1268627	down	0.852301589
At1g24180	IAA conjugate metabolism	IAA	0.3353183	up	1.0655849	0.86620593	up	1.0089248	0.15189561	up	1.1463732
At3g02875	IAA conjugate metabolism	IAA	0.29726785	up	1.2519367	0.7815945	up	1.0731152	0.14071773	up	1.2871265
At1g68100	IAA conjugate metabolism	IAA	0.9972963	up	1.0004025	0.18273182	down	0.853806912	0.46306074	down	0.911721059
At1g28130	IAA conjugate metabolism	IAA	0.01179656	down	0.183780436	0.03019216	down	0.527587024	0.001440628	down	0.053234876
At4g27260	IAA conjugate metabolism (GH3-5)	IAA	0.049795464	down	0.336323564	0.019147428	down	0.3684959	0.004206488	down	0.10837377
At5g54510	IAA conjugate metabolism (GH3-6)	IAA	0.36538506	down	0.727410962	0.602312	down	0.8722656	0.20230684	down	0.656960901
At2g33150	IAA IBA metabolism	IAA	0.42642978	up	1.0769494	0.8701105	up	1.0136842	0.5541302	up	1.028496
At4g16760	IAA IBA metabolism	IAA	0.027479982	up	2.25072	0.03807527	up	1.3818592	0.003640319	up	2.5620205
At4g39850	IAA IBA metabolism	IAA	0.024725249	up	1.64142	0.08730225	up	1.1717178	0.009345871	up	1.7249846
At1g29260	IAA IBA metabolism	IAA	0.012296171	up	1.2708035	0.1532088	down	0.854769602	0.001483613	up	1.4624908
At3g51840	IAA IBA metabolism	IAA	0.44319686	up	1.0720305	0.94663113	down	0.99034336	0.39816815	up	1.052537
At5g56290	IAA IBA metabolism	IAA	0.08905351	up	1.2392828	0.93685836	down	0.996938303	0.28505853	up	1.0963527
At5g62810	IAA IBA metabolism	IAA	0.2599568	up	1.1474339	0.74287534	down	0.945555036	0.12233848	up	1.1762305
At4g29010	IAA IBA metabolism	IAA	0.33490512	down	0.943358581	0.42758194	down	0.926628533	0.025678808	down	0.877623711
At1g30330	IAA signaling	IAA	0.32530883	up	1.0698776	0.91915846	up	1.0131494	0.826065	up	1.0042204
At3g62980	IAA signaling	IAA	0.07813288	up	1.2471148	0.13933222	up	1.217895	0.05935946	up	1.3114295
At1g19220	IAA signaling	IAA	0.26993597	down	0.871687663	0.9618048	up	1.0084605	0.6921131	up	1.037236
At1g19850	IAA signaling	IAA	0.19039083	up	1.1809355				0.16186081	up	1.2774683
At5g25890	IAA signaling	IAA	0.36894786	up	1.1474531	0.097882	up	1.2227426	0.004031497	down	0.663812406
At4g23980	IAA signaling	IAA	0.031801376	down	0.741594838	0.92761064	up	1.0163529	0.019461403	down	0.542183386
At5g20730	IAA signaling	IAA	0.76746947	down	0.985258464	0.19280463	up	1.1424726	0.55867124	down	0.953253861
At2g28350	IAA signaling	IAA	0.045477048	down	0.653073891	0.033453763	down	0.672602066	0.001490481	down	0.46849917
At4g14550	IAA signaling	IAA	0.36874983	down	0.902733939	0.6449537	down	0.953032918	0.8825482	down	0.98437143
At5g15100	IAA transport	IAA									
At4g29080	IAA signaling	IAA	0.005417879	down	0.292323964	0.018134188	down	0.545822003	0.000609375	down	0.256288014
At4g30080	IAA signaling	IAA	0.24197279	down	0.81806343	0.11600903	down	0.800513545	0.028741183	down	0.668579651
At5g37020	IAA signaling	IAA	0.45114723	down	0.87555715	0.029526372	down	0.657331032	0.021184873	down	0.625086848
At1g59750	IAA signaling	IAA	0.56698614	down	0.879809567				0.11896691	down	0.604724787
At4g14560	IAA signaling (IAA1)	IAA							0.005753261	up	3.7128816
At1g04100	IAA signaling (IAA10)	IAA							0.001629458	up	4.4054356
At4g28640	IAA signaling (IAA11)	IAA	0.28113168	up	1.2280375				0.33794087	up	1.2039309
At2g33310	IAA signaling (IAA13)	IAA	0.04185532	up	1.7151427	0.01237766	up	1.5519952	0.00380895	up	2.003946
At1g51950	IAA signaling (IAA18)	IAA				0.100570366	up	1.5005695			
At3g04730	IAA signaling (IAA16)	IAA	0.12283363	down	0.867098661	0.07735159	up	1.3196335	0.4214596	down	0.913885401
At1g04250	IAA signaling (IAA17)	IAA	0.2682488	down	0.845619051	0.061969496	down	0.885644831	0.18823533	down	0.879219042
At3g23030	IAA signaling (IAA2)	IAA	0.024109755	up	1.9624224	0.09352766	up	1.274043	0.003317516	up	3.1038325
At5g43700	IAA signaling (IAA4)	IAA	0.7257199	down	0.905662729	0.69331086	up	1.0963825	0.7639481	down	0.927973913
At3g23050	IAA signaling (IAA7)	IAA	0.03676899	up	1.2854841	0.07224426	up	1.250493	0.003707468	up	2.081999
At2g22670	IAA signaling (IAA8)	IAA	0.26110017	down	0.934507735	0.10909683	down	0.873110654	0.010706445	down	0.754046022
At5g65670	IAA signaling (IAA9)	IAA	0.27844563	down	0.894685577	0.36111468	down	0.879315913	0.15942937	down	0.823786268
At5g62000	IAA signaling, BR downregulated	IAA	0.09335139	down	0.658720955	0.8812009	up	1.040662	0.007380267	down	0.422588273
At5g62000	IAA signaling, BR downregulated	IAA	0.04930014	down	0.582646447	0.8812009	up	1.040662	0.007380267	down	0.422588273
At5g62000	IAA signaling, BR downregulated	IAA	0.09335139	down	0.658720955	0.8812009	up	1.040662	0.008719094	down	0.558802165
At5g62000	IAA signaling, BR downregulated	IAA	0.04930014	down	0.582646447	0.8812009	up	1.040662	0.008719094	down	0.558802165
At5g62000	IAA signaling, BR downregulated	IAA	0.09335139	down	0.658720955	0.042803843	down	0.761180524	0.007380267	down	0.422588273
At5g62000	IAA signaling, BR downregulated	IAA	0.04930014	down	0.582646447	0.042803843	down	0.761180524	0.007380267	down	0.422588273
At5g62000	IAA signaling, BR downregulated	IAA	0.09335139	down	0.658720955	0.042803843	down	0.761180524	0.008719094	down	0.558802165
At5g62000	IAA signaling, BR downregulated	IAA	0.04930014	down	0.582646447	0.042803843	down	0.761180524	0.008719094	down	0.558802165
At3g15540	IAA signaling, BR upregulated	IAA	0.9671054	down	0.990927465	0.6675394	up	1.0818989	0.9185016	up	1.02052
At4g34750	IAA signaling?, CY response up	IAA				0.10173202	down	0.699686603			
At1g04240	IAA signaling, CY response up, BR upregulated (IAA3)	IAA	0.028689183	down	0.4678743				0.027633926	down	0.430179464
At4g31320	IAA signaling?	IAA	0.2755516	down	0.861672299	0.07998812	down	0.732908554	0.30671948	down	0.886176951
At2g36910	IAA transport	IAA	0.15772107	up	1.2078185	0.038061347	up	1.3211617	0.019299613	up	1.3369589
At2g47000	IAA transport	IAA	0.036625702	up	2.4820302	0.000612937	up	3.3320487	0.009116313	up	3.5166118
At1g23080	IAA transport	IAA	0.058151852	down	0.535862145	0.023412462	down	0.734827718	0.004408076	down	0.290098806
At1g70940	IAA transport	IAA	0.21242677	down	0.804337114	0.15271926	down	0.811319991	0.014697269	down	0.413960486
At2g01420	IAA transport	IAA	0.33481172	down	0.798550854	0.54659235	down	0.911956941	0.011362392	down	0.643120606
At3g28860	IAA transport	IAA	0.6149891	down	0.939992743	0.10005079	down	0.866728828	0.001957762	down	0.661805134
At2g38120	IAA transport (AUX1)	IAA	0.042158883	down	0.617314283	0.03879427	down	0.74949793	0.005805201	down	0.431569666
At1g73590	IAA transport (PIN1)	IAA	0.027359206	down	0.501129471	0.3067403	down	0.86141711	0.010413127	down	0.402013719
At5g57090	IAA transport (PIN2)	IAA	0.17440154	up	1.1489114	0.051344585	down	0.867723606	0.007405147	up	1.2792448
At5g64930	JA signaling	JA	0.027491534	up	1.1326408	0.053279974	up	1.2791337	0.041625828	up	1.2478646
At2g44050	JA signaling	JA	0.95864034	up	1.0066371	0.14906989	down	0.925053806	0.41603294	up	1.0715029
At1g32640	JA signaling	JA	0.17064989	down	0.605283898	0.021916801	down	0.423960159	0.005369366	down	0.191750973
At2g39940	JA signaling	JA	0.25984654	down	0.878497011	0.48765606	down	0.923964555	0.044480674	down	0.795356834
At2g46370	JA signaling	JA	0.017405126	down	0.66408863	0.17245765	down	0.848425551	0.002346601	down	0.51334336
At1g32230	JA signaling, ET signaling	JA	0.14647461	up	1.2123431	0.020803593	up	1.5568339	0.038783308	up	1.2059546
At4g01370	JA signaling, SA signaling	JA	0.39201948	up	1.080192	0.682465	down	0.974687369	0.053962305	up	1.519478
At1g55020	JA synthesis	JA	0.25072992	up	1.4992641	0.18824814	up	1.2611551	0.16258928	up	1.341305
At2g06050	JA synthesis	JA	0.0690906	down	0.68550738	0.53200746	up	1.1669049	0.018592509	down	0.6078121
At1g67560	JA synthesis	JA	0.21655262	up	1.2414078	0.1313609	down	0.916864335	0.06545081	down	0.714654834
At1g13280	JA synthesis	JA	0.04535332	down	0.62291002	0.032123867	down	0.641316165	0.026925595	down	0.556029478
At3g22400	JA synthesis	JA	0.05029753	down	0.69461255	0.043658298	down	0.634192099	0.02966376	down	0.605420988
At3g25780	JA synthesis	JA	0.6607891	down	0.928968297	0.08889507	down	0.729670185	0.054896846	down	0.676392057
At5g42650	JA synthesis	JA	0.8295095	down	0.949672036	0.07792385	down	0.750115124	0.008625654	down	0.428916173
At3g01420	JA synthesis, ET signaling, JA and ET response	JA	0.4412959	down	0.661933796	0.01033256	up	2.4722967	0.09588655	down	0.443461124
At1g18870	SA biosynthesis?	SA				0.038325187	up	3.2333665			
At3g53260	SA biosynthesis?	SA	0.06509251	up	1.3832511	0.6756657	up	1.0566231	0.72787476	up	1.0449263
At2g37040	SA biosynthesis?	SA	0.025744982	up	1.5343617	0.019878225	up	1.6376251	0.8583838	down	0.981153416
At3g10340	SA biosynthesis?	SA	0.23234087	up	1.2430476	0.07161653	up	1.3256371	0.005298386	down	0.399974818
At3g53150	SA dependent (nahG)	SA	0.08426506	up	2.4760013	0.003701695	up	2.032815	0.023241706	up	3.0386004
At3g13100	SA dependent (nahG)	SA	0.028400606	up	3.3410213				0.007063367	up	3.9649405
At1g14080	SA dependent (nahG)	SA	0.003349506	down	0.494734491	0.78400993	up	1.0427275	0.004378399	down	0.481640862
At3g07600	SA dependent (nahG)	SA	0.17421101	down	0.584388744	0.26190057	down	0.783491152	0.010384273	down	0.244712228
At4g35580	SA dependent (nahG)	SA	0.04180361	down	0.854669371	0.36282346	down	0.841809939	0.030700652	down	0.794707123
At5g39670	SA dependent (nahG)	SA	0.006783748	up	5.4475307						
At5g11920	SA dependent (nahG), CY cross talk	SA	0.48022637	up	1.2485923	0.4642235	down	0.893189856	0.97123134	down	0.987625156
At5g44070	SA response	SA	0.012986812	up	1.9877783	0.025619788	up	1.358041	0.003838428	up	2.4907358
At1g14790	SA response	SA									
At1g76930	SA response	SA	0.5329337	up	1.1856256	0.31729215	up	1.2493523	0.12903783	down	0.71335672
At2g34690	SA response	SA	0.014511883	down	0.745353985	0.29856658	down	0.923584554	0.010730572	down	0.767218885
At2g05520	SA response	SA	0.32858136	down	0.741048633				0.19882372	down	0.562952343
At1g64280	SA signaling	SA	0.25129095	up	1.2081263	0.1831495	up	1.1930374	0.028949695	up	1.3272696
At4g03550	SA signaling	SA	0.30054328	up	1.1763076	0.24286865	up	1.2318021	0.13817954	up	1.2391231
At4g20380	SA signaling	SA	0.24184702	up	1.141133	0.010070247	up	1.3156862	0.026487151	up	1.3702519
At5g60600	SA signaling	SA	0.32129014	up	1.1995077	0.84484935	down	0.976584149	0.086255565	up	1.3763484
At1g22070	SA signaling	SA	0.004254446	down	0.535584961	0.010576152	up	1.2275816	0.001562901	down	0.434445762
At3g48090	SA signaling	SA	0.19211647	down	0.767914623	0.12401699	down	0.794034703	0.14235955	down	0.767280931
At4g33680	SA signaling	SA	0.18250193	down	0.783088668	0.004259504	down	0.751886144	0.0318723	down	0.72706217
At4g39030	SA signaling	SA	0.05625427	down	0.590919564	0.54174495	down	0.909638677	0.019677049	down	0.609124577
At5g06960	SA signaling	SA	0.09894495	down	0.847483483	0.37549883	down	0.905282058	0.24562949	down	0.962293492
At3g52430	SA signaling	SA	0.08020317	up	1.4172504	0.5812262	up	1.1389277			
At4g01370	SA signaling, JA signaling	SA	0.39201948	up	1.080192	0.682465	down	0.974687369	0.053962305	up	1.519478

Supplementary Table S5 Hormone-related genes in response to As stress (continued)

Functional categories	In genome	On array	Col-0 with 100 uM As/Control			Ws-2 with 100 uM As/Control			Col-0 with 200 uM As/Control		
			Detected	Induced	Repressed	Detected	Induced	Repressed	Detected	Induced	Repressed
Abscisic acid (ABA) receptor	3	3	1	0	0	1	0	0	1	0	0
ABA biosynthesis	13	13	5	0	0	4	0	0	5	1	0
ABA induced	5	5	2	0	0	2	0	0	2	0	0
ABA signaling	33	31	19	1	1	20	0	0	18	4	3
negative regulation of ABA signalinc	12	12	9	1	0	9	0	0	10	3	1
Brassinosteroid (BR) receptor	3	3	1	0	0	1	0	0	1	0	0
BR biosynthesis	13	13	7	0	2	7	0	2	7	0	3
BR conjugation	1	1	0	0	0	0	0	0	0	0	0
BR downregulated	24	24	21	2	4	21	2	2	23	4	8
BR signaling	8	8	5	0	1	4	0	0	5	0	1
BR upregulated	52	51	32	4	4	32	5	0	31	5	10
CY biosynthesis	11	11	4	0	3	2	0	1	3	0	3
CY conjugation	5	5	2	0	0	1	0	0	2	0	0
CY cross talk	8	8	4	0	0	4	0	0	4	0	0
CY degradation	7	6	0	0	0	0	0	0		0	0
CY receptor	5	5	4	0	1	4	0	1	4	0	3
CY response down	11	10	5	0	2	5	1	0	5	0	3
CY response up	62	61	36	5	9	31	1	9	37	8	16
CY signaling	21	19	17	0	0	17	0	1	17	0	2
CY transport	20	14	3	0	1	3	0	1	3	0	2
Ethylene receptor	5	5	4	0	0	0	0	0	5	1	0
ET biosynthesis	26	20	8	1	0	8	0	0	11	3	0
ET signaling	66	63	28	8	2	17	3	2	30	8	2
Gibberellin receptor	3	3	2	0	0	3	0	0	2	0	1
GA biosynthesis	18	18	3	0	1	3	0	1	3	0	1
GA deactivation	3	3	0	0	0	0	0	0		0	0
GA inducible	2	2	2	0	1	2	0	0	2	0	0
GA signaling	14	14	5	1	0	4	0	0	5	1	1
Auxin receptor	4	4	4	0	0	0	0	0	4	0	1
IAA biosynthesis	16	16	10	0	2	10	0	4	10	0	4
IAA conjugate metabolism	16	15	6	0	2	6	0	1	6	0	2
IAA IBA metabolism	10	10	8	1	0	8	0	0	8	1	0
IAA signaling	58	53	33	0	2	31	0	0	35	5	7
IAA transport	13	13	9	1	0	9	1	0	9	1	4
JA biosynthesis	21	20	8	0	0	6	0	0	8	0	0
JA signaling	10	10	7	1	0	7	1	1	7	0	1
SA biosynthesis	6	6	3	0	0	4	1	0	3	0	1
SA dependent (NahG)	29	28	7	2	1	5	1	0	6	2	1
SA response	14	13	4	0	0	3	0	0	4	1	2
SA signaling	13	12	11	0	0	11	0	0	10	0	1

Supplementary Table S6 Transcription factor-related genes in response to As stress

Locus Identifier	Gene Family	Col-100 Corrected p-value	Regulation	Col-100 FC Absolute	Ws-100 Corrected p-value	Regulation	Ws-100 FC Absolute	Col-200 Corrected p-value	Regulation	Col-200 FC Absolute
AT2G36080	ABI3-VP1	0.19941716	up	1.2230055	0.09420731	down	0.760709535	0.30857134	up	1.1976726
AT2G30470	ABI3-VP1	0.10299565	up	1.6895734				0.032547507	up	2.1417863
AT5G25475	ABI3-VP1	0.010263164	up	3.6820576				0.000886423	up	4.66144
AT1G49480	ABI3-VP1	0.13595785	down	0.733376554	0.09043654	down	0.826064857	0.13180207	down	0.73413688
AT3G18990	ABI3-VP1	0.23748168	down	0.862559063	0.04681848	down	0.887922987	0.009175356	down	0.709188925
AT4G32010	ABI3-VP1	0.916117	down	0.994017703	0.8622378	down	0.988393298	0.99404263	down	0.999424631
AT1G14510	Alfin	0.114965476	up	1.2070276	0.4854606	up	1.0688838	0.000945487	up	1.5671761
AT2G02470	Alfin	0.04945017	up	1.5093583	0.033832766	up	1.2185906	0.001914149	up	1.9444537
AT5G05610	Alfin	0.2942206	up	1.2585667	0.871061	up	1.0133984	0.040580593	up	1.3765179
AT5G26210	Alfin	0.23307681	up	1.2175651	0.096623965	up	1.2430664	0.15480414	up	1.1762674
AT3G11200	Alfin	0.169432	up	1.144036	0.9718437	down	0.997985566	0.53462744	up	1.051359
AT3G42790	Alfin	0.07328491	down	0.896614518	0.075335376	up	1.0928257	0.522226	down	0.96507863
AT1G22985	AP2/EREBP	0.00758023	up	10.727437	0.012744497	up	2.624774	0.001302828	up	20.093536
AT1G43160	AP2/EREBP	0.06947312	up	1.5915443	0.59847695	up	1.0927343	0.071813546	up	1.4144913
AT1G50640	AP2/EREBP	0.020354448	up	2.0013468	0.17882377	up	1.4143676	0.000878635	up	2.63416
AT1G68840	AP2/EREBP	0.12280468	up	1.3927796	0.14696038	up	1.1829724	0.006749178	up	1.7838551
AT1G78080	AP2/EREBP	0.22420177	up	1.1999234	0.7122434	up	1.0446773	0.25352588	up	1.2282169
AT2G33710	AP2/EREBP	0.016929382	up	6.7141757	0.005834543	up	3.8164232	0.003068686	up	14.459347
AT3G25730	AP2/EREBP	0.10510567	up	1.354039	0.043506384	up	1.3777198	0.10191186	up	1.4004521
AT3G50260	AP2/EREBP	0.054360803	up	2.467264	0.010966293	up	2.7634714	0.013788533	up	3.2203612
AT4G17490	AP2/EREBP	0.006722895	up	7.028858	0.08813379	up	3.6148252	0.001479318	up	11.462476
AT4G17500	AP2/EREBP	0.015089777	up	3.6043816	0.001810002	up	1.4761107	0.003059956	up	5.815963
AT4G23750	AP2/EREBP	0.7407713	up	1.026462	0.08087204	up	1.2635096	0.23099437	up	1.0796084
AT5G05410	AP2/EREBP	0.03492866	up	4.5876107	0.009211053	up	4.0743513	0.007568589	up	8.469836
AT5G13330	AP2/EREBP	0.09469937	up	3.1183615	0.03807527	up	2.0117412	0.048630085	up	3.5386696
AT5G47230	AP2/EREBP	0.032964908	up	2.3349628	0.009807889	up	1.7954566	0.00330901	up	2.874979
AT5G52020	AP2/EREBP	0.12100748	up	1.5981723	0.060600605	up	1.4849	0.016639018	up	2.4090257
AT3G20310	AP2/EREBP	0.7479827	down	0.91391171	0.55620885	up	1.0154511	0.8194998	up	1.0517133
AT5G60120	AP2/EREBP	0.7543333	down	0.945165201	0.1678429	up	1.421414	0.3514156	up	1.051714
AT2G47520	AP2/EREBP				0.001992044	up	12.572565	0.003499105	up	10.187152
AT3G61630	AP2/EREBP				0.005459929	up	9.42608	0.011024843	up	5.3501983
AT1G22190	AP2/EREBP	0.2805934	up	1.2070838	0.18140231	down	0.925388001	0.10788474	up	1.3153229
AT1G36060	AP2/EREBP	0.016035922	up	2.1525364	0.030512834	down	0.745119171	0.005041556	up	2.1951423
AT3G16770	AP2/EREBP	0.191743	up	1.4339435	0.29196712	down	0.811813444	0.32333913	up	1.3851814
AT3G14230	AP2/EREBP	0.19735834	down	0.866762409	0.003630815	down	0.743413118	0.78036994	up	1.0252085
AT1G53170	AP2/EREBP	0.04487568	up	2.4434264				0.033266027	up	2.2301989
AT2G31230	AP2/EREBP	0.005731739	up	9.990343				0.000889151	up	17.97217
AT1G22810	AP2/EREBP							0.002179487	up	37.17402
AT5G47220	AP2/EREBP							0.031022115	up	3.3936985
AT4G36920	AP2/EREBP	0.9832113	up	1.0042213	0.022983318	up	1.446718	0.5272505	down	0.875407688
AT5G10510	AP2/EREBP	0.96023047	up	1.0091031	0.026295401	up	1.3522102	0.94346005	down	0.99365235
AT2G23340	AP2/EREBP	0.15519987	down	0.783028331	0.847974	up	1.0779551	0.25767934	down	0.869863694
AT2G44940	AP2/EREBP	0.14374064	down	0.690845571	0.114564106	up	1.4366784	0.016851155	down	0.355498676
AT5G53290	AP2/EREBP	0.12071025	down	0.849898565	0.13037248	up	1.0781499	0.003297276	down	0.607463405
AT2G44840	AP2/EREBP	0.98983175	up	1.0057124	0.04500876	down	0.534861497	0.3217889	down	0.661734976
AT3G15210	AP2/EREBP	0.62711954	up	1.0631994	0.24445501	down	0.819533079	0.28728223	down	0.903697152
AT1G13260	AP2/EREBP	0.03863179	down	0.419471005	0.033644382	down	0.709273682	0.001446127	down	0.35493186
AT1G16060	AP2/EREBP	0.004046376	down	0.491818452	0.69331086	down	0.967835628	0.007034743	down	0.476356702
AT1G25560	AP2/EREBP	0.1604318	down	0.713319727	0.024533466	down	0.604189012	0.29353857	down	0.914050297
AT1G53910	AP2/EREBP	0.1358084	down	0.776812278	0.8263813	down	0.971639873	0.038309474	down	0.688558797
AT1G72360	AP2/EREBP	0.008263622	down	0.34162644	0.009099189	down	0.302015239	0.000679271	down	0.196362491
AT2G28550	AP2/EREBP	0.016971607	down	0.517379903	0.040544126	down	0.597144479	0.001993154	down	0.265961337
AT3G54320	AP2/EREBP	0.15891878	down	0.760279993	0.5479952	down	0.900493651	0.033838376	down	0.688269236
AT3G54990	AP2/EREBP	0.81029415	down	0.938736463	0.023855403	down	0.391226052	0.050402895	down	0.54559011
AT5G07580	AP2/EREBP	0.002927062	down	0.321224147	0.004574726	down	0.433767969	0.000471695	down	0.13689224
AT5G13910	AP2/EREBP	0.110037215	down	0.661407682	0.040743794	down	0.645906166	0.05571701	down	0.579088154
AT5G19790	AP2/EREBP	0.29872033	down	0.885577616	0.16418496	down	0.772340807	0.014755006	down	0.727785512
AT5G25810	AP2/EREBP	0.027413188	down	0.43551266	0.018036846	down	0.372260128	0.012407175	down	0.375995585
AT5G51190	AP2/EREBP	0.33217403	down	0.830334856	0.05829063	down	0.626844411	0.08282979	down	0.666423733
AT5G61590	AP2/EREBP	0.002066707	down	0.092061246	0.00810806	down	0.137604466	0.000534384	down	0.039159645
AT5G61600	AP2/EREBP	0.15519987	down	0.742577878	0.08719551	down	0.552325007	0.008369246	down	0.522534865
AT4G39780	AP2/EREBP	0.01591136	down	0.488605858				0.003115624	down	0.312617104
AT1G21910	AP2/EREBP				0.04143313	up	1.612442			
AT1G71130	AP2/EREBP				0.644673	up	1.0883292			
AT1G30330	ARF	0.32530883	up	1.0698776	0.91915846	up	1.0131494	0.826065	up	1.0042204
AT1G19220	ARF	0.26993597	down	0.871687663	0.9618048	up	1.0084605	0.6921131	up	1.037236
AT1G19850	ARF	0.19039083	up	1.1809355				0.16186081	up	1.2774683
AT4G23980	ARF	0.031801376	down	0.741594838	0.92761064	up	1.0163529	0.019461403	down	0.542183386
AT5G20730	ARF	0.76746947	down	0.985258464	0.19280463	up	1.1424726	0.55867124	down	0.953253861
AT5G62000	ARF	0.04930014	down	0.582646447	0.8812009	up	1.040662	0.008719094	down	0.558802165
AT2G28350	ARF	0.045477048	down	0.653073891	0.033453763	down	0.672602066	0.001490481	down	0.46849917
AT4G30080	ARF	0.24197279	down	0.81806343	0.11600903	down	0.800513545	0.028741183	down	0.668579651
AT5G37020	ARF	0.45114723	down	0.87555715	0.029526372	down	0.657331032	0.021184873	down	0.625086848
AT1G59750	ARF	0.56698614	down	0.879809567				0.11896691	down	0.604724787
AT1G20910	ARID	0.027508864	up	1.4916657	0.8979231	up	1.0245764	0.029142287	up	1.7622313
AT1G76510	ARID	0.013507485	up	1.5386916	0.98719126	up	1.0021677	0.012313332	up	1.8473748
AT2G17410	ARID	0.121356525	up	1.1374657	0.15815406	down	0.878813082	0.018592509	up	1.2829404
AT3G13350	ARID	0.08232238	up	1.3340924				0.16910844	up	1.260784
AT3G43240	ARID	0.029986644	down	0.829182721	0.9451256	up	1.0081574	0.19029099	down	0.792678253
AT2G30130	AS2	0.011807157	up	2.606236	0.04143313	up	2.099409	0.00174245	up	5.1288705
AT2G30340	AS2	0.9674047	down	0.994335964	0.69567645	up	1.0766542	0.3013127	down	0.879030851
AT3G02550	AS2	0.025822584	down	0.771171154	0.082442135	down	0.530270407	0.02392503	down	0.663706227
AT3G49940	AS2	0.004236283	down	0.381218508	0.005671362	down	0.648644006	0.000769637	down	0.289015604
AT4G37540	AS2	0.003874918	down	0.41742011	0.008999672	down	0.469748127	0.002667186	down	0.303483477
AT5G67420	AS2	0.035721622	down	0.4321045	0.012842812	down	0.611146904	0.013899138	down	0.455735172
AT2G42430	AS2	0.0895229	up	1.5002187	0.015657164	up	2.075616			
AT2G33310	Aux/IAA	0.04185532	up	1.7151427	0.01237766	up	1.5519952	0.00380895	up	2.003946
AT3G23030	Aux/IAA	0.024109755	up	1.9624224	0.09352766	up	1.274043	0.003317516	up	3.1038325
AT3G23050	Aux/IAA	0.03676899	up	1.2854841	0.07224426	up	1.250493	0.003707468	up	2.081999
AT3G15540	Aux/IAA	0.9671054	down	0.990927465	0.6675394	up	1.0818989	0.9185016	up	1.02052
AT4G28640	Aux/IAA	0.28113168	up	1.2280375				0.33794087	up	1.2039309
AT4G14560	Aux/IAA							0.005753261	up	3.7128816
AT5G25890	Aux/IAA	0.36894786	up	1.1474531	0.097882	up	1.2227426	0.004031497	down	0.663812406
AT3G04730	Aux/IAA	0.12283363	down	0.867098661	0.07735159	up	1.3196335	0.4214596	down	0.913885401
AT5G43700	Aux/IAA	0.7257199	down	0.905662729	0.69331086	up	1.0963825	0.7639481	down	0.927973913
AT1G04250	Aux/IAA	0.2682488	down	0.845619051	0.061969496	down	0.885644831	0.18823533	down	0.879219042
AT2G22670	Aux/IAA	0.26110017	down	0.934507735	0.10909683	down	0.873110654	0.010706445	down	0.754046022
AT4G14550	Aux/IAA	0.36874983	down	0.902733939	0.6449537	down	0.953032918	0.8825482	down	0.98437143
AT4G29080	Aux/IAA	0.005417879	down	0.292323964	0.018134188	down	0.545822003	0.000609375	down	0.256288014
AT5G65670	Aux/IAA	0.27844563	down	0.894685577	0.36111468	down	0.879315913	0.15942937	down	0.823786268
AT1G04240	Aux/IAA	0.028689183	down	0.4678743				0.027633926	down	0.430179464
AT1G51950	Aux/IAA				0.100570366	up	1.5005695			
AT5G42520	BBR-BPC	0.71423984	up	1.0304891	0.43116876	up	1.0536137	0.5509343	up	1.0493531
AT2G21240	BBR-BPC	0.96087414	down	0.994426538	0.17023252	up	1.0392277	0.46595988	up	1.0680183
AT4G36780	BES1	0.21864255	up	1.3843852	0.025188968	up	1.470317	0.02782835	up	1.7917714
AT4G18890	BES1							0.0029923	up	2.988089
AT1G78700	BES1				0.122177	up	1.4521133			
AT1G03040	bHLH	0.39753908	up	1.1882586	0.22066835	up	1.147733	0.07517684	up	1.2623194
AT2G24260	bHLH	0.061858807	up	1.2664765	0.06369738	up	1.1318334	0.017051632	up	1.2089467
AT2G43140	bHLH	0.029676016	up	2.4418051	0.16174762	up	1.3435532	0.005130511	up	3.7232385
AT3G19860	bHLH	0.22424328	up	1.2485368	0.041115638	up	1.5799923	0.002718624	up	1.64722
AT5G58010	bHLH	0.41266903	up	1.2621406	0.15562163	up	1.2081137	0.014695311	up	1.7992547
AT5G62610	bHLH	0.07896495	up	1.3881354	0.019521369	up	1.3923846	0.017537137	up	1.5985615
AT1G10120	bHLH	0.42794803	down	0.891239305	0.01993971	up	1.8144194	0.6865596	up	1.033342
AT1G68810	bHLH	0.85177165	down	0.981490368	0.89798176	up	1.038012	0.7441903	up	1.0376579
AT1G01260	bHLH	0.109247796	up	1.1375731	0.32661074	down	0.970717718	0.20031458	up	1.1443107
AT1G27740	bHLH	0.19230229	up	1.3464307	0.37547603	down	0.852759697	0.047335748	up	1.383764
AT5G48560	bHLH	0.28171632	up	1.2457922	0.4480127	down	0.927855177	0.012222942	up	1.6805896
AT3G56980	bHLH	0.17049178	up	1.4692956	0.002854609	up	3.0740807	0.033487525	down	0.516859117
AT5G54680	bHLH	0.33762968	up	1.0559335	0.81338036	up	1.0261676	0.6787991	down	0.979918914
AT1G05805	bHLH	0.80572474	down	0.987906444	0.48740888	up	1.0532552	0.016994348	down	0.756104561
AT1G69010	bHLH	0.39455658	down	0.917064787	0.34158581	up	1.0702193	0.005038656	down	0.827783048
AT2G28160	bHLH	0.051023707	down	0.492552605	0.053994518	up	1.2203952	0.10633281	down	0.635666686
AT3G23690	bHLH	0.036588978	down	0.705782248	0.044772442	up	1.245165	0.010132349	down	0.609207625
AT3G25710	bHLH	0.59455276	down	0.944987996	0.24018118	up	1.1343614	0.009293309	down	0.683462671
AT4G02590	bHLH	0.04544669	down	0.704018856	0.6703682	up	1.0550411	0.008456862	down	0.551098941
AT4G16430	bHLH	0.3352782	down	0.84996394	0.9015396	up	1.021105	0.055664487	down	0.763452724
AT1G66470	bHLH	0.11229568	up	1.305008	0.024064302	down	0.632217554	0.7840073	down	0.949340171
AT1G74500	bHLH	0.00176257	up	1.3533233	0.1679313	down	0.837357815	0.13210136	down	0.850969897
AT1G32640	bHLH	0.17064989	down	0.605283898	0.021916801	down	0.423960159	0.005369366	down	0.191750973
AT1G51070	bHLH	0.03317037	down	0.520750773	0.08719576	down	0.843793703	0.006180713	down	0.499900945
AT1G68920	bHLH	0.13133398	down	0.609319134	0.7034682	down	0.904165999	0.06902937	down	0.635197137
AT2G22770	bHLH	0.13145982	down	0.659353209	0.0719886	down	0.739274021	0.010100544	down	0.393047617
AT3G07340	bHLH	0.045895007	down	0.697836804	0.9015396	down	0.967953761	0.000975366	down	0.429624573
AT4G14410	bHLH	0.021263367	down	0.802144163	0.11920269	down	0.913111588	0.070600756	down	0.847236987
AT4G17880	bHLH	0.01630794	down	0.688064273	0.7296698	down	0.922939448	0.010090958	down	0.356564662
AT5G08130	bHLH	0.067187026	down	0.776160871	0.11613953	down	0.75005211	0.00540168	down	0.438077792
AT5G46760	bHLH	0.21780924	down	0.911954446	0.40435988	down	0.84043063	0.23825231	down	0.919468617
AT5G57150	bHLH	0.048417807	down	0.338110634	0.060522664	down	0.833531089	0.005494019	down	0.333628728
AT5G65640	bHLH	0.16221902	down	0.80413331	0.5612613	down	0.915964725	0.6899769	down	0.946868465
AT2G42280	bHLH	0.30081782	down	0.692774713				0.19656216	down	0.746125111
AT1G06070	bZIP	0.25508675	up	1.2053047	0.5489442	up	1.0703208	0.09542473	up	1.1596354
AT1G42990	bZIP	0.012213546	up	2.533174	0.004813203	up	2.2766745	0.001744274	up	4.4703197
AT2G40620	bZIP	0.36883456	up	1.1053743	0.1384228	up	1.1678092	0.13739821	up	1.0899329
AT2G46270	bZIP	0.017406391	up	1.8032293	0.12570858	up	1.2916833	0.02975161	up	1.5709074
AT3G17609	bZIP	0.059516106	up	1.9264407	0.07833787	up	1.708491	0.013755606	up	2.5396667
AT3G54620	bZIP	0.075218484	up	1.5424031	0.11309255	up	1.3783766	0.004462041	up	2.1016684
AT3G56850	bZIP	0.13624766	up	1.3330724	0.025162823	up	1.491172	0.019399324	up	1.6220448
AT4G01120	bZIP	0.04089424	up	1.2695429	0.54310745	up	1.0880679	0.99366933	up	1.0003974

AT4G02640	bZIP	0.7239735	up	1.056039	0.090458676	up	1.1424979	0.14087872	up	1.236808
AT5G06839	bZIP	0.05374166	up	1.9998358	0.045228474	up	1.5315229	0.011347864	up	2.467032
AT5G11260	bZIP	0.002327396	up	1.8523738	0.00523089	up	2.011679	0.000534384	up	2.2790005
AT5G24800	bZIP	0.057504084	up	1.5077329	0.001810002	up	4.2311873	0.001083083	up	2.0829258
AT5G44080	bZIP	0.47416508	up	1.0578349	0.2331678	up	1.0701389	0.008723464	up	1.4042516
AT3G10800	bZIP	0.028327037	up	1.2364031	0.6939065	down	0.977217637	0.000975366	up	1.7153784
AT4G36730	bZIP	0.42755178	up	1.129297	0.2917233	down	0.89370209	0.5707026	up	1.0788627
AT1G03970	bZIP	0.5703296	down	0.942752309	0.10366824	down	0.795016392	0.011997066	up	1.4320607
AT1G06850	bZIP	0.08469135	up	2.0084786				0.018775038	up	2.8526137
AT2G41070	bZIP							0.007950301	up	2.730358
AT1G68880	bZIP	0.9052964	up	1.0332587	0.009533367	up	2.9652605	0.025569398	down	0.48848399
AT1G22070	bZIP	0.004254446	down	0.535584961	0.010576152	up	1.2275816	0.001562901	down	0.434445762
AT1G43700	bZIP	0.09411147	down	0.809775283	0.10295214	up	1.2885497	0.066775344	down	0.825438973
AT1G77920	bZIP	0.06684277	down	0.579737569	0.1000026	up	1.2730223	0.004903256	down	0.522004581
AT2G21230	bZIP	0.059172716	down	0.839752612	0.8592478	up	1.017687	0.12392052	down	0.886499831
AT2G31370	bZIP	0.9600089	down	0.991094127	0.43331882	up	1.0874571	0.43388608	down	0.849699249
AT4G34000	bZIP	0.07423415	down	0.709121134	0.12126096	up	1.1439152	0.011745628	down	0.646154534
AT4G35040	bZIP	0.018915795	down	0.585126209	0.74777055	up	1.0158833	0.011609195	down	0.619793118
AT1G08320	bZIP	0.007278231	down	0.384461659	0.05228163	down	0.801877677	0.001085405	down	0.295067678
AT1G49720	bZIP	0.13811272	down	0.694642852	0.6556096	down	0.965144296	0.009410014	down	0.495146941
AT1G58110	bZIP	0.24741918	down	0.872333549	0.015787998	down	0.62309217	0.006990845	down	0.557330157
AT2G40950	bZIP	0.06138568	down	0.822424557	0.17229795	down	0.809470677	0.004768945	down	0.771830055
AT3G19290	bZIP	0.69340616	down	0.94274902	0.94597065	down	0.988797908	0.001861962	down	0.64823934
AT3G58120	bZIP	0.255527	down	0.937670422	0.43927333	down	0.959980063	0.004740592	down	0.519473962
AT5G06960	bZIP	0.09894495	down	0.847483483	0.37549883	down	0.905282058	0.24562949	down	0.962293492
AT5G10030	bZIP	0.080573305	down	0.479479454	0.01719101	down	0.720105867	0.006050473	down	0.400810085
AT5G15830	bZIP	0.003831518	down	0.074837595	0.008838028	down	0.239922304	0.000679271	down	0.024812626
AT5G28770	bZIP	0.52570593	down	0.818412849	0.14675948	down	0.710432683	0.43109092	down	0.827331594
AT5G65210	bZIP	0.013034329	down	0.292939942	0.004922012	down	0.476673005	0.000975366	down	0.183653404
AT2G22850	bZIP	0.3416527	up	1.1612085				0.6124696	down	0.934543979
AT5G06950	bZIP	0.68351287	up	1.0321666	0.34018978	up	1.1563615			
AT1G32150	bZIP				0.16899805	up	1.1577297			
AT4G37730	bZIP				0.013718592	up	2.64086			
AT4G38900	bZIP				0.86181813	up	1.0361428			
AT2G35530	bZIP				0.724145	down	0.938979391			
AT2G47890	C2C2-CO-like	0.16493684	up	1.4651151	0.004351474	up	3.317156	0.005819835	up	2.2233658
AT5G53420	C2C2-CO-like	0.03559546	down	0.702184172	0.017795974	up	2.0249283	0.116177924	up	1.2246394
AT4G27900	C2C2-CO-like				0.023384077	up	1.2589282	0.19139823	up	1.139489
AT2G24790	C2C2-CO-like	0.30489737	up	1.1332759	0.9243211	down	0.981027709	0.27318	up	1.1333692
AT5G02810	C2C2-CO-like	0.11543763	up	1.3509295	0.018223353	down	0.53326826	0.011434331	up	1.9959654
AT5G24930	C2C2-CO-like	0.04089424	up	1.6269996	0.028448531	down	0.601045808	0.017654784	up	1.5457861
AT5G24470	C2C2-CO-like	0.022484737	down	0.377161057	0.2755425	up	1.1809473	0.001466356	down	0.160962963
AT1G28050	C2C2-CO-like	0.66800535	up	1.0547315	0.8946058	down	0.96570688	0.18823533	down	0.704046366
AT5G61380	C2C2-CO-like	0.48670858	up	1.1449915	0.24058259	down	0.851242989	0.09386378	down	0.687570884
AT5G48250	C2C2-CO-like	0.25165987	down	0.780139703	0.67964494	down	0.919304043	0.31356835	down	0.796700767
AT5G57660	C2C2-CO-like	0.0201691	down	0.547585899	0.02877901	down	0.737023739	0.009092798	down	0.570029004
AT5G60100	C2C2-CO-like				0.026675345	down	0.59716994			
AT4G00940	C2C2-Dof	0.011190121	up	3.2512603				0.00082477	up	9.901258
AT5G39660	C2C2-Dof							0.008370288	up	2.3646576
AT1G07640	C2C2-Dof	0.51522624	up	1.1206199	0.017168464	up	1.5597997	0.40274298	down	0.920792338
AT2G34140	C2C2-Dof	0.35165602	up	1.166068	0.8850876	up	1.0138566	0.9090221	down	0.988325113
AT1G29160	C2C2-Dof	0.18156847	up	1.1100477	0.1760544	down	0.819463302	0.128022	down	0.83937424
AT2G28510	C2C2-Dof	0.39419705	down	0.85036718	0.4173711	down	0.92346421	0.041058253	down	0.533461364
AT2G28810	C2C2-Dof	0.039817505	down	0.742807617	0.37794337	down	0.873136269	0.002718624	down	0.356900713
AT3G47500	C2C2-Dof	0.016526528	down	0.395001805	0.012667717	down	0.582366343	0.003499105	down	0.241282173
AT4G24060	C2C2-Dof	0.01456338	down	0.611215486	0.078985095	down	0.845254307	0.001106535	down	0.383788998
AT5G60200	C2C2-Dof	0.009872143	down	0.535073163	0.00695367	down	0.515799187	0.000778458	down	0.298712754
AT3G21270	C2C2-Dof				0.03978519	up	1.6786207			
AT3G55370	C2C2-Dof				0.10473607	up	1.3323045			
AT2G46590	C2C2-Dof	0.020327728	up	4.89236						
AT2G45050	C2C2-GATA	0.08903249	up	1.4331069	0.018365737	up	1.4797617	0.03801697	up	1.405143
AT3G60530	C2C2-GATA	0.047851853	up	1.3418372	0.45690855	up	1.0171618	0.09188833	up	1.1884131
AT4G32890	C2C2-GATA	0.09524209	up	1.3714318	0.6644902	up	1.1309031	0.003409535	up	1.8027093
AT3G24050	C2C2-GATA	0.012974187	up	1.964423	0.18140231	down	0.692228448	0.003852752	up	1.7265761
AT3G16870	C2C2-GATA	0.59490764	up	1.0917598	0.14671995	up	1.1980878	0.5455756	down	0.954334521
AT3G54810	C2C2-GATA	0.3276817	down	0.880053465	0.10164208	down	0.698943749	0.002001471	down	0.543287047
AT4G17570	C2C2-GATA	0.025234912	down	0.670322292	0.6228644	down	0.944576591	0.004223302	down	0.573872317
AT4G34680	C2C2-GATA				0.045398742	up	1.7669361			
AT1G27730	C2H2	0.28777975	up	1.2282759	0.16600026	up	1.2187562	0.7631018	up	1.050481
AT1G34370	C2H2	0.05625427	up	1.3356419	0.009727794	up	1.4429849	0.00697549	up	1.6756743
AT1G74250	C2H2	0.0416984	up	1.5106455	0.11891126	up	1.2371693	0.017644495	up	1.5930436
AT2G01650	C2H2	0.58867127	up	1.0383162	0.63257855	up	1.029251	0.638602	up	1.0529141
AT2G24500	C2H2	0.04581986	up	1.8960991	0.002754528	up	2.453981	0.007831488	up	2.4437373
AT2G28710	C2H2	0.45116037	up	1.3271497	0.17905521	up	1.282851	0.039851286	up	2.6204646
AT2G36930	C2H2	0.05470746	up	1.4344721	0.023343945	up	1.3260204	0.014287612	up	1.5377592
AT2G37430	C2H2	0.03435945	up	5.546898	0.029733595	up	1.9329764	0.007996882	up	9.818258
AT2G48100	C2H2	0.04089424	up	1.1402937	0.77727485	up	1.0139812	0.018899309	up	1.2588019
AT3G19580	C2H2	0.47106844	up	1.1658837	0.15690663	up	1.529603	0.5821383	up	1.1622329
AT3G57480	C2H2	0.09976182	up	1.3309482	0.061432116	up	1.2488475	0.046525728	up	1.6207224
AT3G62240	C2H2	0.19516733	up	1.1796024	0.840318	up	1.0277512	0.17744784	up	1.102368
AT4G15420	C2H2	0.009148149	up	8.268638	0.0025088	up	4.0118685	0.001105544	up	16.612692
AT4G31420	C2H2	0.84517735	up	1.0228161	0.2061393	up	1.0757797	0.27304792	up	1.1097775
AT5G03740	C2H2	0.006356678	up	1.5987219	0.9256334	up	1.0059997	0.001696735	up	1.7984861
AT5G04340	C2H2	0.020473585	up	2.6338897	0.005592544	up	2.2092888	0.010034787	up	2.587418
AT5G16470	C2H2	0.050352678	up	1.2710346	0.41848043	up	1.0328387	0.011376068	up	1.147421
AT5G26610	C2H2	0.6607891	up	1.0501531	0.36802664	up	1.0423611	0.17754805	up	1.2202102
AT5G59820	C2H2	0.09012493	up	1.6922969	0.01033256	up	1.8939687	0.009489067	up	3.2692618
AT5G64610	C2H2	0.33936566	up	1.1610464	0.72243977	up	1.0213288	0.057184316	up	1.0819458
AT5G09740	C2H2	0.7673442	down	0.94026868	0.037118092	up	1.5094318	0.87994105	up	1.0161914
AT3G02790	C2H2	0.012602412	up	1.4114349	0.8578769	down	0.979288153	0.005178383	up	1.3990228
AT1G75710	C2H2	0.7747817	down	0.96571863	0.46847364	down	0.901861424	0.61853415	up	1.0637167
AT4G25610	C2H2	0.061941784	up	1.5811368				0.039272644	up	1.5568577
AT5G61190	C2H2	0.057642967	up	2.0700047				0.003842516	up	4.873436
AT3G09290	C2H2							0.14372568	up	1.5159963
AT3G53600	C2H2							0.00240443	up	15.66346
AT5G66730	C2H2	0.85177165	up	1.025288	0.029532442	up	1.3100426	0.11215406	down	0.823605387
AT1G30970	C2H2	0.18364975	down	0.785423668	0.878208	up	1.0225708	0.035871014	down	0.686776631
AT1G67030	C2H2	0.09382488	down	0.538066308	0.1434917	up	1.6831114	0.003360893	down	0.20076098
AT2G36480	C2H2	0.026850492	down	0.807789221	0.34117928	up	1.0442618	0.032268666	down	0.813961656
AT5G43170	C2H2	0.09614699	down	0.565801466	0.65517217	up	1.0993601	0.2061211	down	0.72867868
AT5G44160	C2H2	0.1018828	down	0.71572099	0.5546306	up	1.0598912	0.001562901	down	0.251033385
AT3G44750	C2H2	0.3415977	up	1.1125635	0.010014894	down	0.769055011	0.08458632	down	0.851415627
AT5G67450	C2H2	0.8840645	up	1.0217865	0.014896033	down	0.692069397	0.42123872	down	0.900189913
AT1G03840	C2H2	0.006138975	down	0.565413413	0.6486081	down	0.954685746	0.00026188	down	0.31195264
AT1G24625	C2H2	0.032216173	down	0.436591807	0.006211679	down	0.72865691	0.001801345	down	0.223524726
AT1G66140	C2H2	0.006356678	down	0.263694972	0.005591058	down	0.418462078	0.001999927	down	0.078973933
AT1G72050	C2H2	0.5272089	down	0.868783992	0.5635021	down	0.903826612	0.030471845	down	0.814081394
AT2G28200	C2H2	0.03129053	down	0.715677656	0.045694776	down	0.617352545	0.016869335	down	0.466614981
AT2G29660	C2H2	0.007961742	down	0.289928437	0.008618869	down	0.515313547	0.000182259	down	0.151537974
AT5G03150	C2H2	0.034655966	down	0.654256982	0.014650066	down	0.674887107	0.001779904	down	0.445044067
AT5G03510	C2H2	0.061013043	down	0.572325585	0.85533065	down	0.965476771	0.009948741	down	0.437829081
AT5G22890	C2H2	0.002348479	down	0.178423337	0.062207323	down	0.542117928	0.001178052	down	0.133296426
AT5G25160	C2H2	0.010118763	down	0.119555188	0.019157518	down	0.434729439	0.000362017	down	0.029742201
AT5G40710	C2H2				0.51703084	down	0.888796296			
AT1G04990	C3H	0.024799025	up	1.7593207	0.033373956	up	1.5038373	0.002060344	up	2.8309326
AT1G21570	C3H	0.2824276	up	1.1812778	0.054075297	up	1.1943876	0.2989813	up	1.0806098
AT1G27650	C3H	0.04182609	up	1.436452	0.13236228	up	1.2075394	0.01490957	up	1.4190478
AT2G02160	C3H	0.1652155	up	1.2819782	0.030608306	up	1.3739034	0.032268666	up	1.1337626
AT2G24830	C3H	0.01775659	up	1.9270644	0.060600605	up	1.2973604	0.014269949	up	2.3127112
AT2G40140	C3H	0.00936831	up	2.1971264	0.05988935	up	1.277632	0.002677135	up	2.809268
AT3G12130	C3H	0.43185055	up	1.096451	0.1790336	up	1.098493	0.04483363	up	1.336748
AT3G47120	C3H	0.62859887	up	1.0803027	0.6624754	up	1.0838445	0.029007446	up	1.2970253
AT5G42820	C3H	0.04003752	up	1.2658337	0.21745989	up	1.346793	0.008176324	up	1.460567
AT5G51980	C3H	0.82561874	up	1.0421766	0.098883726	up	1.1751051	0.050327808	up	1.2954806
AT5G63260	C3H	0.015969412	up	1.2503517	0.025260273	up	1.4615425	0.023315819	up	1.2032446
AT2G20280	C3H	0.35088035	up	1.1616316	0.010431057	down	0.802834455	0.1323071	up	1.0935953
AT2G28450	C3H	0.09268151	up	1.7047597	0.2968883	down	0.883617268	0.009181023	up	1.7894222
AT3G19360	C3H	0.8121997	up	1.0243859	0.16530679	down	0.861706083	0.007318766	up	1.4301707
AT3G55980	C3H	0.33964473	up	1.2746626	0.5001558	down	0.87966337	0.54055214	up	1.1565623
AT3G21810	C3H	0.10213594	up	2.4515264				0.039842643	up	3.2217777
AT3G06410	C3H	0.69501305	up	1.0192848	0.004813203	up	1.5300004	0.21286601	down	0.871272001
AT5G58620	C3H	0.61839974	up	1.0577972	0.5368235	up	1.0816497	0.03756817	down	0.582065841
AT5G12850	C3H	0.00492641	down	0.827939789	0.1777534	up	1.1315222	0.03209945	down	0.56667896
AT5G56900	C3H	0.82765037	down	0.956983771	0.33452132	up	1.089041	0.87094927	down	0.971815598
AT1G75340	C3H	0.24454162	up	1.0442693	0.22059943	down	0.858713985	0.29789314	down	0.851344157
AT1G32360	C3H	0.0207706	down	0.901823605	0.8994993	down	0.989382149	0.16612987	down	0.715250178
AT2G25900	C3H	0.27994657	down	0.516686253	0.024060879	down	0.335014144	0.05706389	down	0.299136632
AT2G41900	C3H	0.07888292	down	0.796521939	0.026675345	down	0.56285833	0.021017548	down	0.525065222
AT3G12680	C3H	0.4591011	down	0.943942377	0.0971188	down	0.855910908	0.010323489	down	0.742520094
AT3G51950	C3H	0.03772402	down	0.51064008	0.011227544	down	0.632768619	0.001167618	down	0.251029995
AT3G02830	C3H				0.4943711	up	1.1418996			
AT2G47850	C3H				0.9484917	down	0.98755025			
AT1G67310	CAMTA	0.08189825	up	1.1636479	0.70104736	up	1.0331236	0.13265063	up	1.1916469
AT5G09410	CAMTA	0.022366565	up	1.405021	0.8462779	up	1.0164769	0.021330059	up	1.3639846
AT4G16150	CAMTA	0.7411907	up	1.0062178	0.6315045	up	1.0479563	0.2091851	down	0.878201296
AT2G22300	CAMTA	0.68284684	down	0.959715556	0.1229709	up	1.3410987	0.6157859	down	0.952399093
AT3G16940	CAMTA	0.4203885	down	0.942834617	0.048610236	down	0.853151837	0.084403895	down	0.871090231
AT5G64220	CAMTA	0.4313953	down	0.895011564	0.85837334	down	0.977532013	0.020428782	down	0.841560782
AT5G23090	CCAAT-Dr1	0.21670519	up	1.2907609	0.21604915	up	1.3247595	0.025886385	up	1.6894401
AT5G08190	CCAAT-Dr1	0.31848735	down	0.907686965	0.47275424	up	1.1459037	0.4929461	down	0.920185502
AT2G34720	CCAAT-HAP2	0.50941616	down	0.883338227	0.652521	up	1.0136677	0.3324696	down	0.92719799
AT2G38880	CCAAT-HAP3	0.15856373	up	1.3409289	0.19731237	up	1.241253	0.004732853	up	2.860384
AT3G53340	CCAAT-HAP3	0.12566854	down	0.84034369	0.3871457	up	1.0940708	0.9133202	up	1.006291

AT2G27470	CCAAT-HAP3	0.63615954	up	1.0607704	0.07269307	down	0.704309572	0.39604053	up	1.1396561
AT1G54830	CCAAT-HAP5	0.082504265	up	1.1969239	0.043092646	up	1.2030238	0.011310725	up	1.7457454
AT3G12480	CCAAT-HAP5	0.22868443	up	1.2027646	0.94207424	up	1.0135181	0.03132331	up	1.5032676
AT1G56170	CCAAT-HAP5				0.01456871	up	3.8584595	0.002142909	up	16.363232
AT1G07980	CCAAT-HAP5	0.14679942	up	1.529914	0.38132042	down	0.863756866	0.023659851	up	1.8627303
AT1G08970	CCAAT-HAP5	0.52614063	down	0.915850804	0.631257	down	0.949338638	0.36961752	down	0.946938043
AT3G22780	CPP	0.6060482	up	1.0536368	0.72531235	up	1.0171657	0.06295557	up	1.1939436
AT1G47870	E2F-DP	0.029798795	up	1.201467	0.16017035	down	0.854986581	0.06565226	up	1.2139618
AT5G02470	E2F-DP	0.11001721	down	0.768914568	0.21354029	down	0.770306354	0.072608426	down	0.65406664
AT1G73730	EIL	0.034478515	up	2.1666145	0.004258467	up	2.1580987	0.008443	up	3.0311124
AT2G27050	EIL	0.07756122	up	1.3203562	0.01851467	up	1.3555582	0.002677375	up	1.6370925
AT3G20770	EIL	0.06822979	down	0.897164172	0.31239355	down	0.925322239	0.003277496	down	0.715923133
AT3G20550	FHA	0.043524206	up	1.533181	0.8078135	up	1.023671	0.008564035	up	1.8598055
AT5G19280	FHA	0.08329071	up	1.564669	0.8407431	up	1.0364971	0.015521195	up	1.6970416
AT3G54350	FHA	0.088650145	down	0.871339872	0.6914468	up	1.032216	0.7863777	up	1.0167189
AT5G38840	FHA	0.7880791	up	1.030415	0.30251026	up	1.0894932	0.043892402	down	0.737685412
AT5G47790	FHA	0.07203862	down	0.686101969	0.036592	up	1.3071555	0.014888529	down	0.616102669
AT5G67030	FHA	0.4711093	down	0.889950343	0.11452172	down	0.775040713	0.33206424	down	0.86082537
AT3G16857	GARP-ARR-B	0.21525566	up	1.1266071	0.25264522	up	1.1720136	0.026204452	up	1.2791226
AT3G16857	GARP-ARR-B	0.18693098	up	1.1451967	0.25264522	up	1.1720136	0.026204452	up	1.2791226
AT3G16857	GARP-ARR-B	0.21525566	up	1.1266071	0.36888504	up	1.113071	0.6009607	up	1.0513378
AT3G16857	GARP-ARR-B	0.18693098	up	1.1451967	0.36888504	up	1.113071	0.6009607	up	1.0513378
AT3G16857	GARP-ARR-B	0.21525566	up	1.1266071	0.25264522	up	1.1720136	0.6009607	up	1.0513378
AT3G16857	GARP-ARR-B	0.18693098	up	1.1451967	0.25264522	up	1.1720136	0.6009607	up	1.0513378
AT3G16857	GARP-ARR-B	0.21525566	up	1.1266071	0.36888504	up	1.113071	0.026204452	up	1.2791226
AT3G16857	GARP-ARR-B	0.18693098	up	1.1451967	0.36888504	up	1.113071	0.026204452	up	1.2791226
AT4G16110	GARP-ARR-B	0.041056868	up	1.3438224	0.13941184	up	1.1846315	0.099796295	up	1.1460578
AT2G25180	GARP-ARR-B	0.30609405	down	0.948549355	0.6118014	up	1.0447695	0.030471845	down	0.799115411
AT4G31920	GARP-ARR-B	0.03261617	down	0.579270906	0.18951865	down	0.896476989	0.003166563	down	0.356339375
AT3G24120	GARP-G2-like	0.33408588	up	1.0458792	0.5139849	up	1.0542456	0.17882632	up	1.0901363
AT4G28610	GARP-G2-like	0.09559751	up	1.1646773	0.1709369	up	1.1796228	0.005451879	up	1.4083261
AT1G79430	GARP-G2-like	0.08934968	up	1.0577965	0.061789274	down	0.783665834	0.13004811	up	1.083884
AT4G13640	GARP-G2-like							0.05664742	up	1.3446594
AT5G42630	GARP-G2-like	0.36496028	up	1.2093997	0.11850884	up	1.2151259	0.9706039	down	0.995820145
AT3G13040	GARP-G2-like	0.015094969	down	0.642552444	0.12965502	up	1.0843831	0.004645855	down	0.654646956
AT1G13300	GARP-G2-like	0.002960329	down	0.149288268	0.0060313	down	0.244729505	0.000471695	down	0.071003847
AT1G25550	GARP-G2-like	0.031707905	down	0.461080431	0.005571554	down	0.406935284	0.000998879	down	0.322127485
AT1G68670	GARP-G2-like	0.028576817	down	0.595716654	0.8092161	down	0.979474236	0.013226045	down	0.559345995
AT1G69580	GARP-G2-like	0.035542678	down	0.597736384	0.023085084	down	0.509853141	0.002652926	down	0.257415364
AT2G01060	GARP-G2-like	0.31397572	down	0.93058289	0.81488585	down	0.988207424	0.09666524	down	0.800008448
AT2G40970	GARP-G2-like	0.12909745	down	0.700354562	0.019667035	down	0.587271099	0.0334805	down	0.570522972
AT3G10760	GARP-G2-like	0.020596076	down	0.523854089	0.20235485	down	0.900733332	0.001599981	down	0.350792314
AT3G25790	GARP-G2-like	0.005434209	down	0.15840618	0.011209538	down	0.374408084	0.000519851	down	0.063140214
AT4G37180	GARP-G2-like	0.89155746	down	0.986234436	0.025302233	down	0.688531726	0.078715526	down	0.536321229
AT5G06800	GARP-G2-like	0.015121697	down	0.383261763	0.010792479	down	0.512884189	0.004491645	down	0.304803521
AT5G29000	GARP-G2-like	0.021263367	down	0.636385129	0.40217325	down	0.894031038	0.014978156	down	0.551768767
AT1G61730	GeBP	0.31060174	down	0.84110281	0.39057162	up	1.1606777	0.25633857	down	0.818574436
AT4G25210	GeBP	0.07108612	down	0.764204888	0.5684371	up	1.026394	0.003622536	down	0.640006554
AT1G44810	GeBP	0.007118106	down	0.839459569	0.37780097	down	0.820890413	0.010351352	down	0.750727699
AT5G28040	GeBP	0.36314774	down	0.847373823	0.52799046	down	0.875044748	0.003214423	down	0.607872397
AT1G01160	GIF	0.11382361	up	1.2941928	0.85533065	up	1.0203543	0.024737623	up	1.2328094
AT5G28640	GIF	0.14977504	up	1.2197325	0.004813203	down	0.63629985	0.17514357	up	1.1196586
AT1G07530	GRAS	0.089973845	up	1.161075	0.11107803	up	1.3070296	0.012666835	up	1.2935659
AT1G21450	GRAS	0.06776047	up	1.3197502	0.03154573	up	1.5638242	0.020344038	up	1.5716348
AT1G50600	GRAS	0.5051554	up	1.0844882	0.3167452	up	1.1230955	0.28603682	up	1.0773706
AT2G29060	GRAS	0.1222948	up	1.7530407	0.022391045	up	2.0834138	0.011413972	up	2.8284044
AT3G46600	GRAS	0.8882594	up	1.0183947	0.03698339	up	2.1949909	0.003204085	up	2.1331685
AT3G60630	GRAS	0.98295677	up	1.0030205	0.9301637	up	1.0059271	0.5300542	up	1.0876969
AT4G17230	GRAS	0.10633393	up	1.5279598	0.029329354	up	1.542628	0.020181183	up	1.8765379
AT5G48150	GRAS	0.034684103	up	1.4640268	0.07433224	up	1.3528765	0.005168512	up	2.033931
AT1G07520	GRAS	0.32443297	down	0.86782263	0.024362944	up	1.8097655	0.30267578	up	1.1282823
AT5G59450	GRAS	0.3256648	up	1.0606474	0.023984758	up	1.595052	0.006749178	down	0.626790623
AT4G00150	GRAS	0.19494632	down	0.779452266	0.62803507	up	1.0771207	0.007524552	down	0.737309249
AT5G52510	GRAS	0.64567614	down	0.924842062	0.5204176	up	1.0580078	0.07651475	down	0.828671229
AT1G14920	GRAS	0.19652511	down	0.814420189	0.750834	down	0.935087358	0.10639055	down	0.740866688
AT1G50420	GRAS	0.070400946	down	0.729551528	0.302771	down	0.943113915	0.045330245	down	0.658869734
AT2G01570	GRAS	0.07315572	down	0.547037543	0.052634854	down	0.812392231	0.004908105	down	0.491126812
AT2G04890	GRAS	0.08944218	down	0.598511741	0.7951326	down	0.975777019	0.14875291	down	0.730529294
AT2G45160	GRAS	0.023470664	down	0.603283928	0.073148236	down	0.87000703	0.002515898	down	0.503667607
AT3G54220	GRAS	0.17364109	down	0.891436972	0.042638727	down	0.680439951	0.011755955	down	0.527410694
AT4G37650	GRAS	0.8585894	down	0.977736165	0.3790085	down	0.836277448	0.13508408	down	0.866267075
AT3G52910	GRF	0.1951432	up	1.2110065	0.064108595	up	1.3099442	0.061970875	up	1.3534149
AT2G36400	GRF	0.7410959	down	0.931171005	0.95874584	up	1.008236	0.94811827	up	1.0100033
AT2G22840	GRF	0.25834647	up	1.1673162	0.058438372	down	0.6136575	0.5181807	down	0.930568082
AT4G37740	GRF	0.5097171	up	1.1259298	0.10340211	down	0.800755208	0.09924387	down	0.80643711
AT1G26960	HB	0.15385881	up	1.2436771	0.082348466	up	1.2991478	0.07880948	up	1.2694347
AT1G69780	HB	0.07531454	up	1.4866649	0.6851636	up	1.030498	0.07000171	up	1.2679678
AT4G16780	HB	0.25781587	up	1.2569716	0.049937256	up	1.7265071	0.02821814	up	1.8730699
AT4G17460	HB	0.8744903	up	1.0273305	0.0110349	up	1.6405509	0.070103854	up	1.3095527
AT4G37790	HB	0.035043217	up	2.9739542	0.31187212	up	1.2033141	0.011953034	up	3.6558454
AT3G19510	HB	0.2101018	up	1.3966138	0.017820336	down	0.862044516	0.14889522	up	1.3030071
AT4G00730	HB	0.8051891	up	1.031678	0.23949635	down	0.92077021	0.06869757	up	1.296211
AT5G44180	HB	0.8452024	up	1.0179594	0.16406833	up	1.1187968	0.04095937	down	0.807180419
AT1G23380	HB	0.41340056	down	0.899900876	0.40924916	up	1.0099254	0.19027676	down	0.857455706
AT1G23380	HB	0.13269965	down	0.778662104	0.40924916	up	1.0099254	0.19027676	down	0.857455706
AT1G23380	HB	0.41340056	down	0.899900876	0.40924916	up	1.0099254	0.008509189	down	0.476875428
AT1G23380	HB	0.13269965	down	0.778662104	0.40924916	up	1.0099254	0.008509189	down	0.476875428
AT1G79840	HB	0.14967677	down	0.829419441	0.99679327	up	1.0005804	0.003952611	down	0.501130676
AT2G22430	HB	0.34597304	down	0.906161208	0.3730695	up	1.0964649	0.00491667	down	0.552699359
AT5G15150	HB	0.095528044	down	0.876830636	0.76967967	up	1.0433216	0.004336411	down	0.568140079
AT5G47370	HB	0.13411227	down	0.803536912	0.02892003	up	1.1970581	0.046992697	down	0.747900867
AT1G28420	HB	0.95129156	down	0.992851075	0.37794337	down	0.923346114	0.20653021	down	0.791944781
AT1G52150	HB	0.15797997	down	0.735793646	0.9940739	down	0.999185963	0.14840558	down	0.787506989
AT2G34710	HB	0.32170427	down	0.876245715	0.44013909	down	0.841610432	0.045721516	down	0.607167233
AT3G01220	HB	0.025822584	down	0.707810111	0.49241632	down	0.941543872	0.014965514	down	0.669021086
AT4G02560	HB	0.6145682	down	0.966903007	0.40594023	down	0.951681693	0.2987496	down	0.84318326
AT4G40060	HB	0.05992385	down	0.514587898	0.15846992	down	0.737823605	0.003774412	down	0.409500711
AT5G60690	HB	0.00664473	down	0.607899964	0.11791914	down	0.795128344	0.000972307	down	0.529177722
AT5G65310	HB	0.11176211	down	0.64320673	0.043392863	down	0.767060048	0.14620435	down	0.698394642
AT1G27050	HB	0.4837024	down	0.844974262				0.36051148	down	0.794080164
AT1G75410	HB	0.30841732	down	0.646043703				0.07626312	down	0.430300524
AT2G16400	HB	0.007118106	down	0.366901897				0.007380267	down	0.210564971
AT1G30490	HB				0.06806795	down	0.718788462			
AT3G28730	HMG	0.043124184	up	1.1673257	0.2904135	down	0.948094931	0.16074707	up	1.1686136
AT1G20696	HMG	0.18751113	up	1.1550483	0.7674185	up	1.0166395	0.76546085	down	0.980343044
AT2G17560	HMG	0.11679494	up	1.1273155	0.4328599	down	0.939246088	0.2422398	down	0.949315477
AT4G23800	HMG	0.97922176	up	1.0042812	0.006060074	down	0.563251384	0.002899901	down	0.424324684
AT1G20693	HMG	0.007854969	down	0.554188096	0.20067619	down	0.880911588	0.002633012	down	0.398824727
AT3G51880	HMG	0.88559216	down	0.976120481	0.30857697	down	0.87694713	0.4646691	down	0.890939316
AT4G35570	HMG	0.5851789	down	0.959807394	0.18951865	down	0.842926824	0.20283581	down	0.834773735
AT1G32330	HSF	0.9069646	up	1.0185771	0.6674457	up	1.0401074	0.00718152	up	1.7192837
AT2G26150	HSF	0.009699666	up	32.213947	0.005567456	up	14.212848	0.001573711	up	128.34378
AT3G51910	HSF	0.495439	up	1.2085966	0.29106015	up	1.2268379	0.001877999	up	4.4491777
AT5G45710	HSF	0.022773314	up	1.959127	0.037936978	up	1.4716272	0.003841657	up	2.4451132
AT5G62020	HSF	0.016147802	up	4.188483	0.008786433	up	2.3091547	0.000889151	up	14.284025
AT1G67970	HSF				0.027885096	up	2.3701916	0.001084539	up	6.9121294
AT4G11660	HSF	0.015872933	up	3.3011513				0.000909444	up	10.262974
AT1G77570	HSF							0.039004486	up	2.3366601
AT1G46264	HSF	0.81704473	down	0.977299483	0.050937995	down	0.700457483	0.005702582	down	0.563369554
AT4G00990	JUMONJI	0.12909745	up	1.1352531	0.21164295	up	1.0690225	0.018578192	up	1.2251837
AT5G04240	JUMONJI	0.21833208	up	1.1947814	0.4901065	up	1.1400512	0.7793511	up	1.053686
AT5G46910	JUMONJI	0.017289607	up	1.3494496	0.65683955	up	1.0940454	0.011107076	up	1.3821838
AT1G09060	JUMONJI	0.62513673	down	0.926059519	0.07003374	up	1.2813919	0.31275043	up	1.1329166
AT2G38950	JUMONJI	0.08113293	up	1.1313128	0.62061733	down	0.962183124	0.14891833	up	1.1850868
AT5G06550	JUMONJI	0.5897472	down	0.922812289	0.040498465	down	0.811622894	0.24886596	up	1.0537688
AT1G63490	JUMONJI	0.033646494	up	1.6705416				0.003048276	up	1.995757
AT1G08620	JUMONJI	0.26895985	down	0.91103954	0.16706052	up	1.0958658	0.060027044	down	0.730851882
AT1G78280	JUMONJI	0.38513097	down	0.864991144	0.361594	down	0.885503119	0.0962282	down	0.741963937
AT1G19270	LIM	0.14188676	up	1.134753	0.14622964	up	1.0732602	0.029985704	up	1.3348811
AT3G55770	LIM	0.062735796	down	0.833180653	0.061635487	up	1.2110655	0.05101957	down	0.815266086
AT1G01780	LIM	0.28001714	down	0.781032043	0.3205137	down	0.827527812	0.013662293	down	0.575606488
AT1G10200	LIM	0.007445758	down	0.658630887	0.044580936	down	0.713094388	0.00259417	down	0.47116079
AT2G39900	LIM	0.15271348	down	0.876355141	0.7388881	down	0.965643654	0.011153328	down	0.746188915
AT4G36860	LIM	0.0690906	down	0.585951943	0.009211053	down	0.647625191	0.006513093	down	0.372295718
AT5G17890	LIM	0.64687973	up	1.094686						
AT4G32551	LUG	0.02581604	up	1.4784769	0.6228644	up	1.0423312	0.006689496	up	1.4546573
AT2G32700	LUG	0.052917454	up	1.1738137	0.47123975	down	0.940190708	0.921215	up	1.0145335
AT1G71692	MADS	0.024535825	down	0.493779221	0.010417465	down	0.737078715	0.001562901	down	0.275331837
AT3G57230	MADS	0.12399584	down	0.618758046	0.037199095	down	0.692335178	0.000889151	down	0.375401205
AT4G37940	MADS				0.122177	down	0.695426928			
AT3G24500	MBF1	0.015863087	up	16.806759	0.006460856	up	5.1495824	0.004667494	up	26.04942
AT3G58680	MBF1	0.075218484	up	1.3005459	0.1645468	down	0.895102892	0.5945562	up	1.0560993
AT2G42680	MBF1	0.9611513	up	1.0070655	0.096700005	up	1.1473416	0.1551158	down	0.947313816
AT1G06180	MYB	0.052699264	up	2.170592	0.28327307	up	1.2629335	0.009472011	up	2.9043186
AT1G09770	MYB	0.02405937	up	1.6507715	0.110604584	up	1.1595376	0.005357907	up	1.6230086
AT1G18570	MYB	0.00977634	up	11.894185	0.019853469	up	2.5624964	0.003274166	up	9.87029
AT1G79180	MYB	0.16662216	up	1.5020635	0.07719705	up	1.9883642	0.28603682	up	1.3402382
AT2G23290	MYB	0.09351492	up	1.474997	0.7232607	up	1.0581082	0.1548597	up	1.2387662
AT2G36890	MYB	0.20022364	up	1.5574762	0.09890249	up	1.2948357	0.05612442	up	1.8484244
AT2G37630	MYB	0.30525875	up	1.1923814	0.5841167	up	1.0552492	0.36702862	up	1.1129123
AT2G47460	MYB	0.031087227	up	1.577212	0.003083379	up	1.7189106	0.00248279	up	2.819402
AT3G55730	MYB	0.0963727	up	1.5272094	0.009193028	up	1.5842232	0.008443	up	1.8857633
AT4G32730	MYB	0.6673781	up	1.0660484	0.15950228	up	1.0719233	0.008782304	up	1.3379415

AT4G32730	MYB	0.08711651	up	1.4326558	0.15950228	up	1.0719233	0.008782304	up	1.3379415
AT4G32730	MYB	0.6673781	up	1.0660484	0.5775432	up	1.0609605	0.09764032	up	1.2605692
AT4G32730	MYB	0.08711651	up	1.4326558	0.5775432	up	1.0609605	0.09764032	up	1.2605692
AT4G32730	MYB	0.6673781	up	1.0660484	0.15950228	up	1.0719233	0.09764032	up	1.2605692
AT4G32730	MYB	0.08711651	up	1.4326558	0.15950228	up	1.0719233	0.09764032	up	1.2605692
AT4G32730	MYB	0.6673781	up	1.0660484	0.5775432	up	1.0609605	0.008782304	up	1.3379415
AT4G32730	MYB	0.08711651	up	1.4326558	0.5775432	up	1.0609605	0.008782304	up	1.3379415
AT5G04760	MYB	0.03255845	up	1.9546126	0.030921742	up	1.9227521	0.002718624	up	3.1169524
AT5G14750	MYB	0.07584952	up	1.2479447	0.023855403	up	1.3902904	0.8895805	up	1.015888
AT5G16600	MYB	0.071260355	up	1.6658814	0.0087585	up	1.9331181	0.011755955	up	1.9436022
AT3G48920	MYB	0.7061676	down	0.845972372	0.98954374	up	1.002579	0.6298954	up	1.1924157
AT4G17785	MYB				0.008216102	up	5.340094	0.06679056	up	4.6341114
AT3G11450	MYB	0.14661726	up	1.404898	0.7426675	down	0.964176597	0.092095934	up	1.3651946
AT4G01680	MYB	0.12636058	up	1.4878578	0.4202111	down	0.666772595	0.10923968	up	1.3971417
AT5G06110	MYB	0.062735796	up	1.2704619	0.37459192	down	0.917667176	0.9897415	up	1.0016747
AT3G11440	MYB	0.020327728	up	2.6665947				0.002668794	up	4.096467
AT3G23250	MYB	0.013922243	up	4.1680384				0.019863352	up	2.6284368
AT3G28910	MYB							0.077206574	up	1.9039536
AT4G09460	MYB	0.06375937	down	0.701877079	0.21604915	up	1.2269533	0.028949695	down	0.710784289
AT5G14340	MYB	0.19933967	down	0.677335835	0.35380498	up	1.2506031	0.008233422	down	0.45186348
AT4G37260	MYB	0.89236486	up	1.0254177	0.2953809	down	0.852352587	0.47347435	down	0.930345238
AT5G45420	MYB	0.90673065	up	1.0089095	0.05983419	down	0.702432961	0.7773645	down	0.98629309
AT5G57620	MYB	0.825727	up	1.0654775	0.061406367	down	0.630002654	0.67222387	down	0.907388895
AT1G22640	MYB	0.13427755	down	0.872486225	0.7029952	down	0.948809003	0.00174245	down	0.52878558
AT2G16720	MYB	0.038943008	down	0.681038949	0.07833787	down	0.666200282	0.004107018	down	0.411971004
AT2G38090	MYB	0.028693272	down	0.646924054	0.37625074	down	0.802212373	0.002181858	down	0.412105466
AT3G11280	MYB	0.007278231	down	0.405978191	0.01212136	down	0.451504164	0.000644533	down	0.165067782
AT4G38620	MYB	0.001602469	down	0.12464585	0.055298004	down	0.347911159	0.00026188	down	0.057529568
AT5G05790	MYB	0.006862041	down	0.414423341	0.16343236	down	0.838840742	0.000876234	down	0.215306408
AT5G08520	MYB	0.5071619	down	0.890934474	0.084466904	down	0.849562671	0.7670465	down	0.972523678
AT5G10280	MYB	0.00492641	down	0.201581179	0.19920635	down	0.765266863	0.001382239	down	0.080311203
AT5G26660	MYB	0.015722107	down	0.521075118	0.54534566	down	0.93520594	0.012831397	down	0.623270813
AT5G58900	MYB	0.022452265	down	0.757402244	0.005708301	down	0.648766886	0.00255286	down	0.582534644
AT5G59780	MYB	0.006450811	down	0.134037405	0.00629379	down	0.256858996	0.001100269	down	0.027666201
AT5G60890	MYB	0.00492641	down	0.323515794	0.011525319	down	0.190818016	0.001401019	down	0.052967614
AT5G61420	MYB	0.010908958	down	0.055238075	0.022239825	down	0.352486896	0.000269321	down	0.014395271
AT5G65790	MYB	0.12820035	down	0.663752351	0.05707689	down	0.760616321	0.044833545	down	0.457370926
AT5G67300	MYB	0.007445758	down	0.548186498	0.31386453	down	0.912871192	0.002632539	down	0.389090085
AT1G57560	MYB	0.17070156	down	0.504151435				0.1713427	down	0.563663257
AT3G46130	MYB	0.04559611	down	0.213200496				0.006097663	down	0.087932272
AT3G50060	MYB				0.79502887	down	0.958436996			
AT4G34990	MYB				0.23885687	down	0.81350675			
AT1G49950	MYB-related	0.8054765	up	1.0153764	0.5067368	up	1.0828257	0.83840364	up	1.0350008
AT1G72740	MYB-related	0.0895229	up	1.2714213	0.12851948	up	1.2490172	0.120332	up	1.2982327
AT2G46410	MYB-related	0.16636303	up	1.3072813	0.06270076	up	1.3036704	0.053128738	up	1.2811177
AT5G02840	MYB-related	0.035282932	up	1.5795164	0.020342216	up	1.4154202	0.002813752	up	2.2129462
AT5G67580	MYB-related	0.027770203	up	2.0757341	0.043658298	up	1.9253439	0.015550653	up	2.4105508
AT5G37260	MYB-related				0.02182219	down	0.434250633	0.13735597	up	1.4591568
AT4G01280	MYB-related	0.75253147	down	0.955675302				0.13119905	up	1.2247659
AT4G01280	MYB-related	0.75253147	down	0.955675302				0.13119905	up	1.2247659
AT1G17520	MYB-related							0.011235356	up	2.0742455
AT2G46830	MYB-related							0.000826175	up	22.799437
AT2G36960	MYB-related	0.23005968	down	0.833015885	0.17303972	up	1.2224182	0.6393044	down	0.93403857
AT1G19000	MYB-related	0.014213896	down	0.492784234	0.021802861	down	0.572021447	0.002633012	down	0.328243653
AT1G70000	MYB-related	0.14894542	down	0.630240568	0.5425967	down	0.941257086	0.26500365	down	0.797119021
AT1G74840	MYB-related	0.017877731	down	0.416434817	0.005738346	down	0.649886468	0.003171321	down	0.224535991
AT5G47390	MYB-related	0.75125986	down	0.923905738	0.03700971	down	0.598898255	0.8999977	down	0.970765588
AT3G16350	MYB-related	0.2948571	down	0.73281392				0.013021784	down	0.51468727
AT5G52660	MYB-related				0.2838346	up	1.340235			
AT1G01010	NAC	0.42502624	up	1.303365	0.019877248	up	1.9722921	0.6840852	up	1.1222751
AT1G01720	NAC	0.19934985	up	1.4146701	0.008730779	up	2.2796404	0.01711272	up	2.3474545
AT1G32870	NAC	0.006334205	up	6.134373	0.003559839	up	6.5732327	0.000470407	up	14.882087
AT1G33060	NAC	0.94277793	up	1.0123305	0.9795033	up	1.002957	0.086807445	up	1.0880716
AT1G77450	NAC	0.1259421	up	1.8474364	0.015415192	up	2.5373318	0.03747708	up	2.38904
AT2G33480	NAC	0.019171527	up	3.4747598	0.062484823	up	1.6056293	0.004491645	up	4.283506
AT2G43000	NAC	0.017704053	up	34.480377	0.00935342	up	10.305442	0.006980976	up	45.7885
AT3G29035	NAC	0.1582903	up	1.1761868	0.047388192	up	1.4133441	0.052452914	up	1.4091548
AT3G49530	NAC	0.10496171	up	1.347564	0.30995265	up	1.1474134	0.052553944	up	1.4116734
AT5G04410	NAC	0.019998856	up	1.4763709	0.002512239	up	1.4610595	0.002846189	up	1.9130251
AT5G09330	NAC	0.17438337	up	1.2616012	0.010508021	up	1.7497278	0.027772812	up	1.4773706
AT5G13180	NAC	0.22464396	up	1.1655387	0.025274463	up	1.4534351	0.05396887	up	1.2570349
AT5G18270	NAC	0.7146046	up	1.0852723	0.019014608	up	2.1567152	0.2892227	up	1.1813635
AT5G39610	NAC	0.06329027	up	3.4441302	0.01994895	up	3.4927855	0.03787985	up	3.501431
AT5G63790	NAC	0.07721809	up	2.745548	0.006582444	up	3.3951726	0.02309942	up	3.4984243
AT1G33280	NAC	0.7116183	up	1.1113845	0.7181427	down	0.927749469	0.2091851	up	1.5051174
AT1G02220	NAC	0.06324258	up	4.846071				0.021944106	up	6.4810905
AT5G22290	NAC	0.04478758	up	1.7575122				0.004085849	up	2.2630596
AT3G10500	NAC	0.84457844	up	1.0534582	0.0025088	up	2.7816527	0.52964795	down	0.872029269
AT1G25580	NAC	0.57312703	down	0.907103445	0.26574537	up	1.2332486	0.39106655	down	0.866299344
AT1G34190	NAC	0.21780589	down	0.920135886	0.8011837	up	1.0259851	0.046165694	down	0.735178219
AT3G04070	NAC	0.84761983	down	0.95086898	0.50630045	up	1.0906081	0.008509189	down	0.376319225
AT1G56010	NAC	0.005021514	down	0.245558721	0.006200098	down	0.340616045	0.001578146	down	0.207196515
AT3G04060	NAC	0.1133793	down	0.591151304	0.15114681	down	0.72260162	0.07445046	down	0.630181788
AT4G35580	NAC	0.04180361	down	0.854669371	0.36282346	down	0.841809939	0.030700652	down	0.794707123
AT5G14000	NAC	0.2162301	down	0.848109881	0.10220738	down	0.887786849	0.03183449	down	0.815771806
AT5G24590	NAC	0.7125684	down	0.947602868	0.25279537	down	0.929396274	0.7960009	down	0.9656596
AT4G27410	NAC	0.019029113	down	0.502633094				0.018592509	down	0.461060278
AT1G34180	NAC				0.1427304	up	1.4097495			
AT1G20640	Nin-like	0.019670509	up	1.7908266	0.008111879	up	1.6939242	0.002072365	up	2.9126823
AT2G43500	Nin-like	0.12867895	up	8.402978	0.005085019	up	22.752289	0.0426542	up	19.27055
AT4G24020	Nin-like	0.36548743	up	1.2429562	0.103285484	up	1.1963758	0.005977382	up	2.7810583
AT2G17150	Nin-like	0.036851224	down	0.610396605	0.038906638	up	1.4198371	0.0011561	down	0.477242182
AT1G64530	Nin-like	0.20991397	down	0.816544631	0.0143959	down	0.740120684	0.017245572	down	0.646195787
AT1G24610	PcG	0.12315038	up	1.4592618	0.6550022	up	1.0429248	0.005010757	up	1.4496751
AT2G23380	PcG	0.121356525	up	1.153082	0.53678244	up	1.1151986	0.4089035	up	1.1988606
AT2G22740	PcG	0.6640286	down	0.974045771	0.68116	up	1.0533834	0.55394924	up	1.0486876
AT4G30860	PcG				0.066390626	up	1.2524285	0.091246866	up	1.611494
AT4G02020	PcG	0.07064607	up	1.1434503	0.56239575	down	0.967749365	0.011488632	up	1.4179446
AT1G76710	PcG	0.28969502	up	1.2562807				0.01764324	up	2.3479517
AT2G23740	PcG	0.4369288	up	1.1060436	0.6315045	up	1.0581367	0.5264675	down	0.903706707
AT2G44150	PcG	0.1769486	down	0.93005788	0.93275994	up	1.0087198	0.045138784	down	0.892759019
AT5G14260	PcG	0.11544716	down	0.731699108	0.58371216	down	0.934194761	0.061133143	down	0.648291997
AT1G73100	PcG	0.00988287	down	0.724165452				0.006300792	down	0.706603671
AT4G13460	PcG	0.31869772	down	0.796893581				0.008369246	down	0.655000067
AT5G04940	PcG	0.96087414	down	0.991568495	0.21491109	down	0.828457378			
AT1G05380	PHD	0.11105425	up	1.1899581	0.06270216	up	1.2840433	0.21612194	up	1.108788
AT1G77250	PHD	0.018351726	up	1.78759	0.09890249	up	1.2332743	0.004317884	up	2.064737
AT2G25170	PHD	0.032826807	up	1.1938318	0.38553923	up	1.0667182	0.21589598	up	1.060393
AT2G36720	PHD	0.023686396	up	1.2705446	0.069962695	up	1.1068904	0.017075658	up	1.325764
AT2G37520	PHD	0.21300994	up	1.3563523	0.007138968	up	1.3834674	0.03163219	up	1.8374002
AT5G12400	PHD	0.13087283	up	1.1027678	0.015755154	up	1.01467	0.1314716	up	1.0801383
AT5G16680	PHD	0.13133398	up	1.4529392	0.38589367	up	1.1364882	0.0648662	up	1.2548513
AT5G60410	PHD	0.51878333	up	1.2403919	0.011410872	up	1.2480712	0.010877087	up	1.6135309
AT4G22140	PHD	0.76019895	down	0.944338783	0.75144607	up	1.0192932	0.27047026	up	1.1415186
AT3G24010	PHD	0.6521256	up	1.0849899	0.3595961	down	0.901904696	0.001562901	up	1.3467091
AT4G23860	PHD							0.011488632	up	2.103852
AT1G79350	PHD	0.516425	up	1.0504498	0.7122434	up	1.0352817	0.30967578	down	0.913683665
AT1G77800	PHD	0.22862428	down	0.850329796	0.076535895	up	1.1453595	0.03804038	down	0.816120668
AT2G18090	PHD	0.5307938	down	0.948262423	0.09576801	up	1.1975389	0.1334319	down	0.900163497
AT4G14920	PHD	0.16278675	down	0.885560833	0.91405886	up	1.0078995	0.11088325	down	0.78086518
AT4G39100	PHD	0.06868177	down	0.744170485	0.46383846	up	1.0608261	0.005090104	down	0.665786586
AT3G51120	PHD	0.41217297	up	1.0236368	0.98745865	down	0.998658003	0.0322601	down	0.805093472
AT5G22760	PHD	0.3123845	up	1.1095504	0.7033037	down	0.974434351	0.44662333	down	0.938995526
AT5G44800	PHD	0.060258072	up	1.2344475	0.569397	down	0.85465089	0.62158144	down	0.919207118
AT1G54390	PHD	0.32860288	down	0.839458935	0.37165952	down	0.905757228	0.178214	down	0.885605928
AT3G05670	PHD	0.84316456	down	0.974388301	0.7034682	down	0.971378715	0.66721565	down	0.94807965
AT3G14740	PHD	0.032204952	down	0.749914135	0.02504724	down	0.57647088	0.003317516	down	0.557275059
AT1G33420	PHD	0.1115847	up	1.300439	0.036617577	up	1.5617583			
AT3G01460	PHD				0.33396924	up	1.0898149			
AT5G24330	PHD	0.15263748	up	1.5856639						
AT4G17900	PLATZ	0.02683263	up	1.5080638	0.023353059	up	1.6344873	0.001964642	up	2.4203465
AT5G46710	PLATZ				0.009211053	up	3.1072104	0.002575971	up	3.8912964
AT1G21000	PLATZ	0.057159767	down	0.70976265	0.08216482	down	0.770614021	0.8946542	down	0.984389357
AT1G32700	PLATZ	0.03182949	down	0.393965861	0.013718592	down	0.376818687	0.007470974	down	0.301500026
AT3G53370	S1Fa-like	0.23137355	up	1.328952	0.10061409	down	0.774955124	0.82597345	up	1.0294127
AT2G37120	S1Fa-like	0.9331561	up	1.0102525	0.10533365	down	0.903107249	0.60086286	down	0.948578418
AT3G09735	S1Fa-like	0.45818648	down	0.925218904	0.17140232	down	0.843959558	0.0088989	down	0.765761164
AT3G60030	SBP	0.5543729	down	0.940037278	0.2204124	up	1.0560949	0.18835825	up	1.1089102
AT1G20980	SBP	0.6777106	down	0.964403668	0.25309646	up	1.092109	0.017580891	down	0.722310376
AT2G47070	SBP	0.42469537	down	0.968280674	0.41653362	up	1.2439543	0.4026462	down	0.944940759
AT2G47070	SBP	0.42469537	down	0.968280674	0.045398742	up	1.2505912	0.4026462	down	0.944940759
AT5G18830	SBP	0.19320187	down	0.796164589	0.04985921	up	1.3228017	0.029401071	down	0.786513248
AT3G12980	TAZ	0.19354546	up	1.1063964	0.6514746	up	1.0505323	0.77426535	up	1.0300919
AT5G67480	TAZ	0.7914762	down	0.944110811	0.013106542	up	2.058354	0.19770062	up	1.3080083
AT4G37610	TAZ	0.9133913	up	1.0298823	0.29489344	down	0.902892796	0.15311742	up	1.4771298
AT1G16710	TAZ	0.36679822	down	0.887215954	0.91726506	up	1.0119452	0.044382025	down	0.649717107
AT1G79000	TAZ	0.7840057	up	1.0091865				0.4205398	down	0.909883914
AT1G58100	TCP	0.1446091	down	0.76723884	0.10573662	down	0.77012014	0.16117229	down	0.827283204
AT3G47620	TCP	0.028904302	down	0.480831138	0.025187373	down	0.752071506	0.002331507	down	0.133391275
AT5G08330	TCP	0.020473585	down	0.702263515	0.23096915	down	0.876244256	0.001577863	down	0.290230496
AT1G53320	TLP	0.07762874	up	1.2347437	0.12435566	up	1.1885208	0.04532995	up	1.2390573
AT1G47270	TLP	0.051277	up	1.8698151				0.003277212	up	2.6891034
AT1G25280	TLP	0.30960944	down	0.870741172	0.12506114	up	1.1309808	0.1583773	down	0.861727172

AT1G76900	TLP	0.008501425	down	0.905773719	0.83777034	up	1.0261424	0.037487883	down	0.87733197
AT2G18280	TLP	0.14237672	down	0.747216451	0.94597065	up	1.0411146	0.007530315	down	0.597412142
AT2G18280	TLP	0.14237672	down	0.747216451	0.94597065	up	1.0411146	0.007530315	down	0.597412142
AT2G47900	TLP	0.28427935	down	0.919415866	0.43004373	up	1.094882	0.6962993	down	0.99027422
AT3G06380	TLP	0.018443847	down	0.565059447	0.117544256	down	0.731493097	0.01692258	down	0.465614768
AT5G18680	TLP	0.017559117	down	0.515149756	0.034820847	down	0.620627871	0.001999927	down	0.438487869
AT3G10030	Trihelix	0.07309783	up	1.58918	0.23760714	up	1.1681061	0.024449278	up	1.7344359
AT3G14180	Trihelix	0.528617	up	1.0613545	0.049156822	up	1.3767989	0.147248	up	1.1577083
AT1G54060	Trihelix	0.043477625	down	0.812054659	0.9507711	up	1.002579	0.91979015	up	1.0077251
AT1G76880	Trihelix	0.002903	up	3.222504	0.019091593	down	0.656850906	0.00021261	up	6.8112373
AT1G13450	Trihelix	0.29336873	down	0.853778263	0.28711733	down	0.877074193	0.15853749	down	0.849304216
AT1G21200	Trihelix	0.2400611	down	0.810693107	0.7845101	down	0.943603819	0.056386206	down	0.697003804
AT1G33240	Trihelix	0.14995839	down	0.702714862	0.19076446	down	0.893962064	0.003295129	down	0.250820371
AT1G76890	Trihelix	0.005424962	down	0.435830687	0.15471278	down	0.764035504	0.003652526	down	0.261432372
AT3G24490	Trihelix	0.78107953	down	0.959109517	0.07262175	down	0.862679534	0.019478457	down	0.851338069
AT4G28190	ULT	0.11206044	up	1.5323572	0.4339468	up	1.1352061	0.002381268	up	1.6305194
AT2G42400	VOZ	0.022558717	up	1.6699266	0.104269825	up	1.1278989	0.003143723	up	2.290031
AT1G14410	Whirly	0.62013847	up	1.0650673	0.5982171	up	1.017142	0.75542337	up	1.0445583
AT1G13960	WRKY	0.0910784	up	1.5853317	0.005459929	up	1.2262108	0.00564791	up	2.217946
AT1G62300	WRKY	0.03607168	up	3.4481583	0.006617936	up	2.7867045	0.011161139	up	4.089654
AT1G80840	WRKY	0.07114346	up	4.7960606	0.08273333	up	1.6770331	0.03281982	up	5.8383794
AT2G23320	WRKY	0.014647626	up	7.0406013	0.003783142	up	3.5820549	0.00267055	up	11.477117
AT2G30250	WRKY	0.18138455	up	1.3832269	0.01502979	up	2.282873	0.011810336	up	2.114799
AT2G30590	WRKY	0.5976538	up	1.0935237	0.23530993	up	1.068542	0.7873251	up	1.0499876
AT2G38470	WRKY	0.019999148	up	5.0134516	0.012645463	up	2.4252894	0.007778357	up	5.8347106
AT3G01970	WRKY	0.08579823	up	5.194118	0.0618118	up	2.929946	0.028796768	up	8.027534
AT4G23810	WRKY	0.012258009	up	5.9796596	0.1642069	up	1.6460155	0.007962293	up	4.570185
AT4G31550	WRKY	0.1414928	up	1.5147952	0.06876596	up	1.2182199	0.028999507	up	1.9173558
AT4G31800	WRKY	0.06010442	up	1.7925882	0.02343393	up	1.4795367	0.026776718	up	1.9320334
AT5G13080	WRKY	0.005430143	up	8.310296	0.012700422	up	3.6305032	0.001256445	up	12.948419
AT1G69810	WRKY	0.815054	up	1.045379	0.444822	down	0.852895341	0.19625393	up	1.2460672
AT4G01720	WRKY	0.13029708	up	1.4243271				0.016315307	up	1.3220389
AT5G24110	WRKY							0.001446053	up	20.04331
AT1G68150	WRKY	0.36978564	down	0.839949334	0.21496625	up	1.1124084	0.74497145	down	0.949611343
AT3G04670	WRKY	0.8925528	down	0.989319309	0.027229914	up	1.3570063	0.47995263	down	0.960577607
AT4G22070	WRKY	0.6853088	down	0.781429424	0.039433565	up	2.1640954	0.48250777	down	0.684866445
AT4G26640	WRKY	0.70048946	down	0.968469443	0.1782015	up	1.3021418	0.025203003	down	0.865527419
AT1G29280	WRKY	0.007118106	down	0.580438334	0.061943416	down	0.738128422	0.001999927	down	0.541711927
AT1G30650	WRKY	0.012652176	down	0.535542539	0.033198237	down	0.768937922	0.014503841	down	0.297064174
AT2G04880	WRKY	0.040142998	down	0.79448494	0.2894223	down	0.91813268	0.004048324	down	0.646282486
AT2G24570	WRKY	0.007118106	down	0.556775358	0.03851855	down	0.777812676	0.001320766	down	0.473798448
AT3G58710	WRKY	0.049795464	down	0.578615103	0.025426172	down	0.691769889	0.003975459	down	0.322648276
AT4G24240	WRKY	0.0975666	down	0.714135746	0.5879299	down	0.976339487	0.053937066	down	0.690310301
AT5G15130	WRKY	0.59825325	down	0.886840956	0.9165972	down	0.989139153	0.27343628	down	0.78052661
AT5G52830	WRKY	0.028537014	down	0.611548606	0.7023148	down	0.953312747	0.028855832	down	0.538949271
AT2G34830	WRKY				0.37794337	up	1.098589			
AT4G30935	WRKY				0.042461168	up	1.4950277			
AT2G03340	WRKY				0.18347767	down	0.802440575			
AT1G69600	ZF-HD	0.30529857	up	1.1823726	0.19900863	up	1.5397837	0.024363363	up	1.6194197
AT2G18350	ZF-HD	0.33490512	up	1.1727587	0.68331057	down	0.925552448	0.027577613	up	1.7680196
AT1G74660	ZF-HD	0.03620528	down	0.634929907	0.039842952	down	0.571740317	0.008415733	down	0.421383422
AT3G21175	ZIM	0.0877155	up	1.1741929	0.6221867	up	1.0650432	0.04901916	up	1.1504533
AT1G51600	ZIM	0.53854895	down	0.920600169	0.18737882	up	1.1147228	0.028014848	up	1.2949964
AT5G20900	ZIM	0.27447295	down	0.874979515	0.5879299	up	1.0576104	0.23888578	up	1.1203889
AT1G72450	ZIM	0.042358685	up	2.1315682	0.91644496	down	0.974608429	0.04227719	up	1.9779253
AT4G32570	ZIM	0.7859748	up	1.0590625	0.05930225	down	0.685422147	0.5620967	up	1.1124655
AT4G14720	ZIM							0.005010239	up	1.7307707
AT1G19180	ZIM	0.93456316	up	1.0106316	0.77180123	down	0.97278337	0.20228264	down	0.77742803
AT3G17860	ZIM	0.65703446	up	1.0385057	0.9805162	down	0.99756773	0.11707641	down	0.819877504
AT1G74950	ZIM	0.118059635	down	0.678494223	0.11613953	down	0.730033049	0.01865371	down	0.375078832
AT4G24470	ZIM	0.087972	down	0.72103491	0.7428334	down	0.97209022	0.05605954	down	0.813354103

Supplementary Table S6 Transcription factor-related genes in response to As stress (continued)

Functional categories	In genome	On array	Col-0 with 100 µM As/Control			Ws-2 with 100 µM As/Control			Col-0 with 200 µM As/Control		
			Detected	Increase	Decrease	Detected	Increase	Decrease	Detected	Increase	Decrease
Transcription factors	1922	1648									
ABI3-VP1	60	45	6	1	0	4	0	0	6	2	0
Alfin	7	7	6	0	0	6	0	0	6	0	0
AP2/EREBP	146	131	46	10	7	47	7	5	50	17	9
ARF	23	20	10	0	0	8	0	0	10	0	1
ARID	10	9	6	0	0	5	0	0	6	0	0
AS2	42	27	7	1	3	7	2	1	6	1	3
Aux/IAA	29	28	14	0	2	13	0	0	15	4	2
BBR-BPC	7	5	2	0	0	2	0	0	2	0	0
BES1	8	7	1	0	0	2	0	0	2	1	0
bHLH	127	89	34	1	1	33	1	1	34	1	7
bZIP	72	66	38	1	3	40	5	2	38	8	7
C2C2-CO-like	37	34	10	0	1	12	2	0	11	1	1
C2C2-Dof	36	32	10	2	1	10	0	0	10	2	4
C2C2-GATA	26	18	7	0	0	8	0	0	7	0	0
C2C2-YABBY	5	5	0	0	0	0	0	0	0	0	0
C2H2	134	107	43	3	5	42	3	2	45	8	11
C3H	59	52	26	1	0	27	0	1	26	4	1
CAMTA	6	6	6	0	0	6	0	0	6	0	0
CCAAT-Dr1	2	2	2	0	0	2	0	0	2	0	0
CCAAT-HAP2	10	10	1	0	0	1	0	0	1	0	0
CCAAT-HAP3	11	11	3	0	0	3	0	0	3	1	0
CCAAT-HAP5	13	10	4	0	0	5	1	0	5	1	0
CPP	8	6	1	0	0	1	0	0	1	0	0
E2F-DP	8	7	2	0	0	2	0	0	2	0	0
EIL	6	6	3	1	0	3	1	0	3	1	0
FHA	16	13	6	0	0	6	0	0	6	0	0
GARP-ARR-B	10	9	9	0	0	11	0	0	11	0	1
GARP-G2-like	43	40	16	0	4	16	0	0	17	0	6
GeBP	21	11	4	0	0	4	0	0	4	0	0
GIF	3	3	2	0	0	2	0	0	2	0	0
GRAS	33	32	19	0	0	19	2	0	19	3	1
GRF	9	8	4	0	0	4	0	0	4	0	0
HB	87	80	27	1	1	25	0	0	27	1	4
HMG	11	11	7	0	0	7	0	0	7	0	2
HRT-like	2	2	0	0	0	0	0	0		0	0
HSF	23	23	7	3	0	7	3	0	9	7	0
JUMONJI	17	16	9	0	0	8	0	0	9	0	0
LFY	1	1	0	0	0	0	0	0		0	0
LIM	13	13	7	0	0	6	0	0	6	0	2
LUG	2	2	2	0	0	2	0	0	2	0	0
MADS	104	83	2	0	1	3	0	0	2	0	2
MBF1	3	3	3	1	0	3	1	0	3	1	0
MYB	150	142	48	3	8	47	2	4	50	6	13
MYB-related	49	39	13	1	2	12	0	1	16	4	2
NAC	107	95	28	3	1	26	8	1	28	9	3
Nin-like	14	11	5	0	0	5	1	0	5	3	1
NZZ	1	1	0	0	0	0	0	0		0	0
PcG	34	30	11	0	0	9	0	0	11	1	0
PHD	56	52	23	0	0	23	0	0	22	2	0
PLATZ	10	7	3	0	1	4	1	1	4	2	1
S1Fa-like	3	3	3	0	0	3	0	0	3	2	0
SAP	1	1	0	0	0	0	0	0		0	0
SBP	16	15	5	0	0	5	0	0	5	0	0
SRS	10	7	0	0	0	0	0	0		0	0
TAZ	9	8	5	0	0	4	1	0	5	0	0
TCP	23	17	3	0	1	3	0	0	3	0	2
TLP	11	11	9	0	0	7	0	0	9	1	2
Trihelix	26	23	9	1	1	9	0	0	9	1	2
ULT	2	1	1	0	0	1	0	0	1	0	0
VOZ	2	2	1	0	0	1	0	0	1	1	0
Whirly	2	1	1	0	0	1	0	0	1	0	0
WRKY	72	62	26	5	0	28	6	0	27	10	3
ZF-HD	16	14	3	0	0	3	0	0	3	0	1
ZIM	18	16	9	1	0	9	0	0	10	0	1

Supplementary Table S7 Protein kinase-related genes in response to As stress (continued)

Functional categories	In genome	On array	Ws-2 with 100 uM As/Control			Col-0 with 200 uM As/Control		
			Detected	Induced	Repressed	Detected	Induced	Repressed
Mitogen-activated protein kinase (MAPK)	24	23	13	0	0	14	1	2
MAPKK	10	10	4	0	0	4	0	1
Raf (MAPKKK)	48	48	24	0	0	29	2	1
ZIK (MAPKKK)	10	10	3	0	0	4	0	3
Protein tyrosine phosphatase	19	18	9	1	0	11	2	1
Calcium-dependent protein kinase	32	34	16	0	0	16	2	1
Snf1-related kinases (SnRK)	38	37	24	0	2	24	3	5
LRR-RLK VIII	22	19	8	1	0	9	4	0

Supplementary Table S7 Protein kinase-related genes in response to As stress

Gene Family	Gene names	Locus Identifier	Ws-100 Corrected p-value	Regulation	Ws-100 FC Absolute	Col-200 Corrected p-value	Regulation	Col-200 FC Absolute
MPK	MPK5	AT4G11330				0.005888908	up	2.155881
	MPK16	AT5G19010	0.032664787	down	1.5288843	0.007255682	down	2.2279932
	MPK18	AT1G53510	0.043238223	down	1.4634643	0.000628484	down	3.8249023
MKK	MKK5	AT3G21220	0.01978298	down	1.4410349	0.001957762	down	2.4491267
Raf	Raf30	AT4G38470	0.11462523	down	1.2607273	0.001237319	down	3.888232
	Raf39	AT3G22750				0.009571609	up	3.716461
	Raf43	AT3G46930	0.09990737	up	1.4913504	0.001100788	up	4.970173
ZIK	ZIK2	AT5G58350	0.021916801	down	1.4473217	0.008831373	down	2.4759338
	ZIK4	AT3G04910	0.01609429	down	1.4676596	0.002624963	down	2.2464192
	ZIK9	AT5G28080	0.06739957	down	1.966967	0.000424419	down	11.545998
Protein tyrosine	(PTP)	AT1G05000	0.09929064	down	1.301241	0.002024347	down	5.8147125
	(PTP)	AT3G02800	0.003779925	up	3.297418	0.000644954	up	34.25881
		AT3G44620	0.0720714	up	1.4176216	0.013625029	up	2.0610466
Aurora α	AtAUR2	AT2G25880	0.030511038	down	2.1343777	0.030590666	down	1.6689097
CPK	CRK4	AT5G24430	0.07939206	up	1.2736388	0.001259501	up	2.413365
	CRK6	AT3G49370				0.006678334	up	2.1600504
	CRK8	AT1G49580	0.022239825	down	1.4019369	0.001612004	down	2.007245
SnRK	SnRK2.3	AT5G66880	0.041825876	up	1.4786288	0.001611023	up	3.4205697
	SnRK2.6	AT4G33950	0.3569852	up	1.1374819	0.00081214	up	5.824634
	SnRK2.7	AT4G40010	0.030040054	down	3.648632	0.05444009	down	2.18796
	SnRK2.9	AT2G23030	0.013914318	down	6.129734	0.003968458	down	18.378265
	SnRK2.10	AT1G60940	0.022462012	up	1.9776024	0.008967559	up	2.679956
	SnRK3.3	AT4G14580	0.869629	down	1.0234052	0.002459923	down	3.1096435
	SnRK3.4	AT5G57630	0.034684673	down	1.7315407	0.00088781	down	4.8857946
	SnRK3.17	AT2G26980	0.09632188	down	1.394864	0.00170818	down	2.8338127
PPCK	PPCK1	AT1G08650	0.031097824	down	2.9924026	0.02628545	down	2.1793053
	PPCK2	AT3G04530	0.016263295	down	2.363028	0.002494718	down	5.073665

Supplementary Table S7 Protein kinase-related genes in response to As stress (LRR-RLK VIII)

		Ws-100/Control			Col-200/Control			Col-100/Control		
Locus	Subfamily	FDR	Regulation	FC Absolute	FDR	Regulation	FC Absolute	FDR	Regulation	FC Absolute
AT1G06840	LRR-VIII-1	0.039	up	1.515	0.086	up	1.668	0.267	up	1.3615478
AT5G01950	LRR-VIII-1	0.557	up	1.096	0.004	up	2.774	0.007	up	2.1218593
AT5G49760	LRR-VIII-1	0.091	up	1.179	0.152	down	1.289	0.323	down	1.1834111
AT1G16670	LRR-VIII-2	0.035	up	1.246	0.001	up	2.074	0.003	up	1.4855266
AT1G29750	LRR-VIII-2	0.072	up	1.414	0.017	up	2.421			
AT1G53430	LRR-VIII-2	0.050	up	1.356	0.006	up	7.128	0.022	up	4.542342
AT1G53440	LRR-VIII-2				0.002	up	6.831	0.007	up	4.797799
AT3G09010	LRR-VIII-2	0.005	up	2.567	0.006	up	11.457	0.049	up	4.4277954
AT3G14840	LRR-VIII-2	0.004	up	1.768	0.458	down	1.053	0.162	down	1.2434701

Supplementary Table S8 Genes that commonly regulated after long-term (Abercrombie et al., 2008) and short-term (this study) exposure to As

Genes	Regulation	Description
AT1G10960	Upregulated	ATFD1_FD1__ferredoxin 1
AT4G21990	Upregulated	APR3_ATAPR3_PRH-26_PRH26__APS reductase 3
AT3G03270	Upregulated	Adenine nucleotide alpha hydrolases-like superfamily protein
AT1G12520	Upregulated	ATCCS_CCS__copper chaperone for SOD1
AT4G19810	Downregulated	ChiC__Glycosyl hydrolase family protein with chitinase insertion domain
AT3G56090	Downregulated	ATFER3_FER3__ferritin 3
AT3G61060	Downregulated	AtPP2-A13_PP2-A13__phloem protein 2-A13
AT5G20790	Downregulated	unknown protein; FUNCTIONS IN: molecular_function unknown;
AT4G25100	Downregulated	ATFSD1_FSD1__Fe superoxide dismutase 1
AT4G31500	Downregulated	ATR4_CYP83B1_RED1_RNT1_SUR2__cytochrome P450, family 83, subfamily B, polypeptide 1
AT5G01600	Downregulated	ATFER1_FER1__ferretin 1
AT3G02040	Downregulated	AtGDPD1_GDPD1_SRG3__senescence-related gene 3
AT3G14050	Downregulated	AT-RSH2_ATRSH2_RSH2__RELA/SPOT homolog 2
AT2G15890	Downregulated	MEE14__maternal effect embryo arrest 14
AT3G01290	Downregulated	AtHIR2_HIR2__SPFH/Band 7/PHB domain-containing membrane-associated protein family
AT4G14130	Downregulated	XTH15_XTR7__xyloglucan endotransglucosylase/hydrolase 15
