# Supplementary material for: Transcriptome profiling of genes and pathways associated with arsenic toxicity and tolerance in Arabidopsis
Source: BMC Plant Biol. 2014 Apr 16;14:94. doi: 10.1186/1471-2229-14-94 (PMC4021232; doi:10.1186/1471-2229-14-94)
Supplement: Additional file 2: Figure S1 — Transcriptional changes of genes involved in sulfur assimilation, cysteine biosynthesis and phytochelatin synthesis in As-treated roots. [file 1471-2229-14-94-S2.pdf]

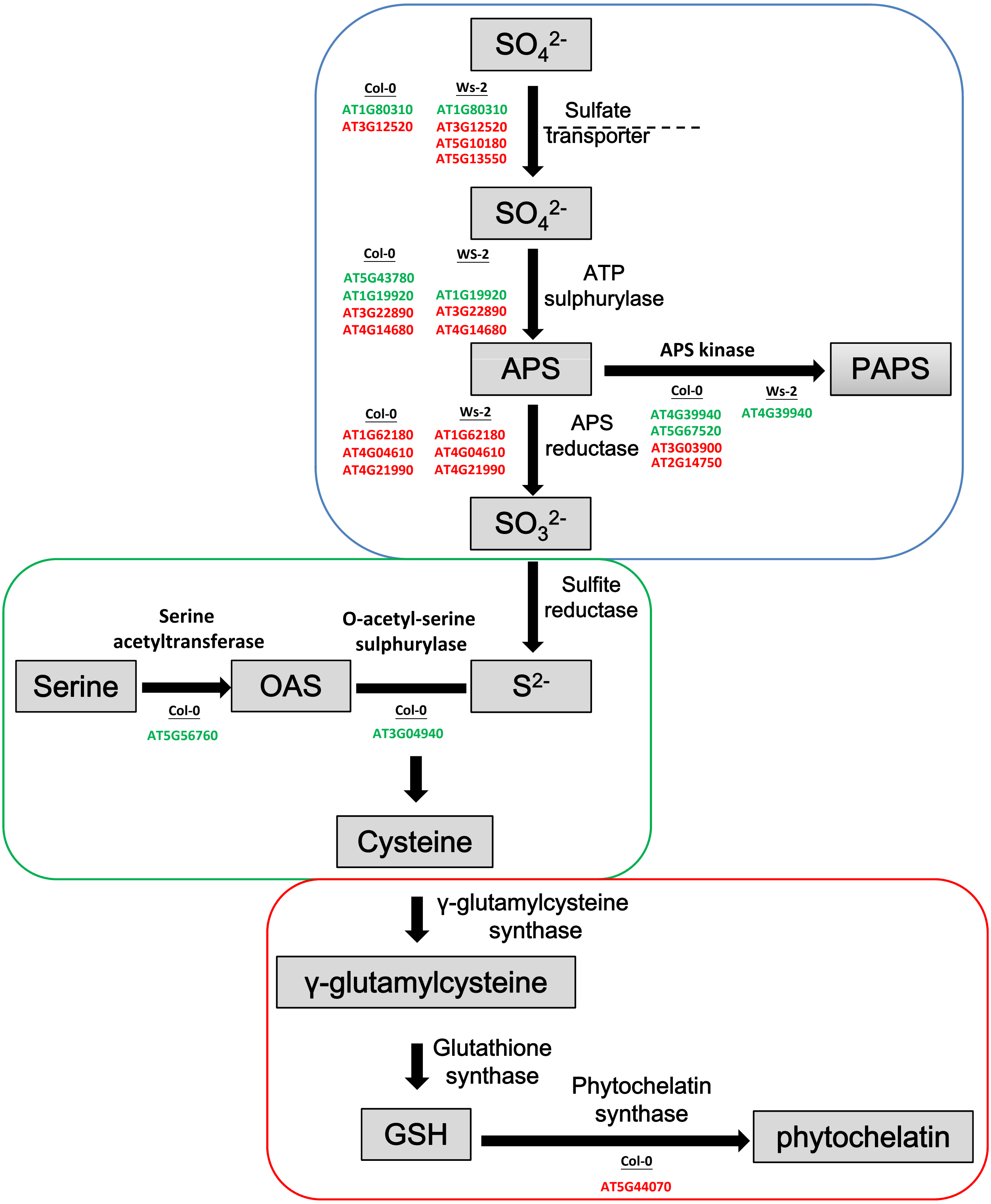

Supplementary Fig. S1 Transcriptional changes of genes involved in sulfur assimilation, cysteine biosynthesis and phytochelatin synthesis in As-treated roots. The reactions of the sulfur assimilation, cysteine synthesis and phytochelatin synthesis pathways are grouped by boxes. The metabolites are represented in gray boxes; arrows represent enzymatic reactions; names of the enzymes are given adjacent to the arrows; the AGI (*Arabidopsis* Genome Initiative) numbers of As-regulated genes are given corresponding to the Col-0 and Ws-2 ecotypes. Genes marked in red and green indicate up- and down-regulation in response to As stress, respectively.
