# Supplementary material for: Transcriptome profiling of genes and pathways associated with arsenic toxicity and tolerance in Arabidopsis
Source: BMC Plant Biol. 2014 Apr 16;14:94. doi: 10.1186/1471-2229-14-94 (PMC4021232; doi:10.1186/1471-2229-14-94)
Supplement: Additional file 3: Figure S2 — Effects of NaCl (20 mM) , CuCl2 (25 μM) , ZnSO4 (200 μM) and CdCl2 (100 μM) on the root elongation of Arabidopsis wild-type and LRR-RLK VIII mutant lines was assessed. [file 1471-2229-14-94-S3.pdf]

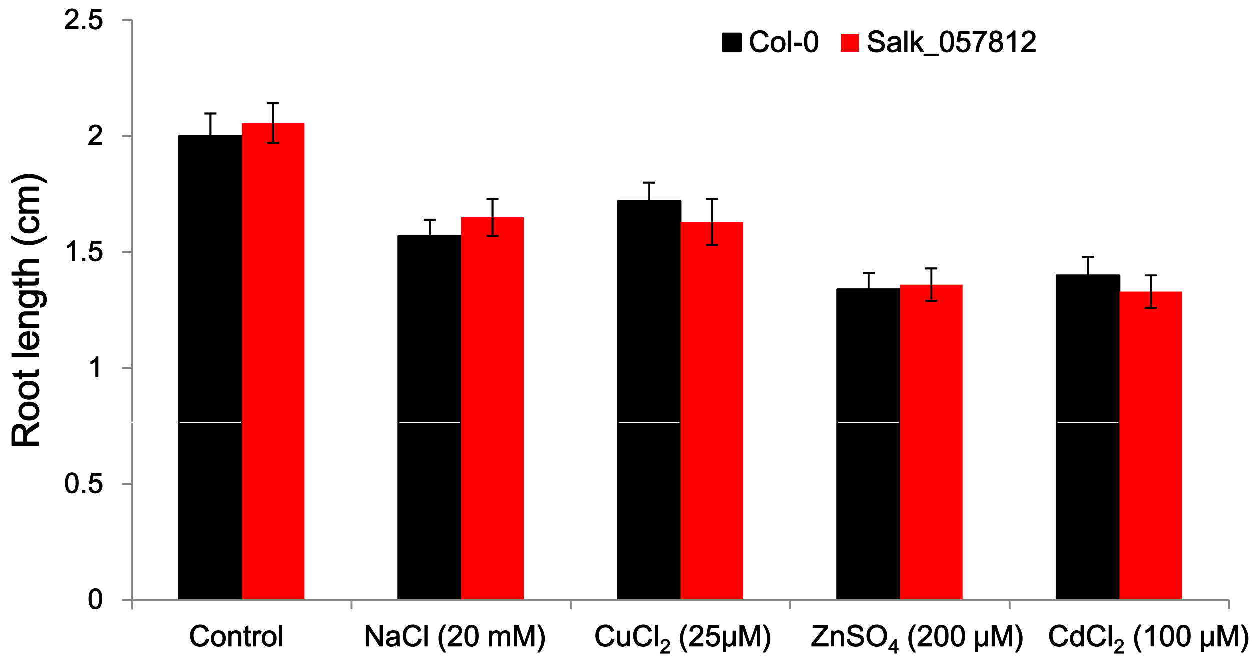

Supplementary Fig. S2 Effects of NaCl (20 mM) , $CuCl_2$ (25μM) , $ZnSO_4$ (200 μM) and $CdCl_2$ (100 μM) on the root elongation of Arabidopsis wild-type and LRR-RLK VIII mutant lines was assessed. Measurement of root elongation was similar to that described in Fig. 1. Seedlings were grown on quarter-strength MS medium for 4 d and then transferred to medium with various metals or ions and grown for an additional 4 d. As tolerance was determined by relative root growth after treatment. Root length of plants was measured after treatment with As. Root samples were collected from 2 independent experiments (each from a pool of 10 root samples). Data are mean SD.
