# Supplementary material for: Transcriptome profiling of genes and pathways associated with arsenic toxicity and tolerance in Arabidopsis
Source: BMC Plant Biol. 2014 Apr 16;14:94. doi: 10.1186/1471-2229-14-94 (PMC4021232; doi:10.1186/1471-2229-14-94)
Supplement: Additional file 4: Figure S3 — Effects of As on the growth of shoots in 2 Arabidopsis accessions. [file 1471-2229-14-94-S4.pdf]

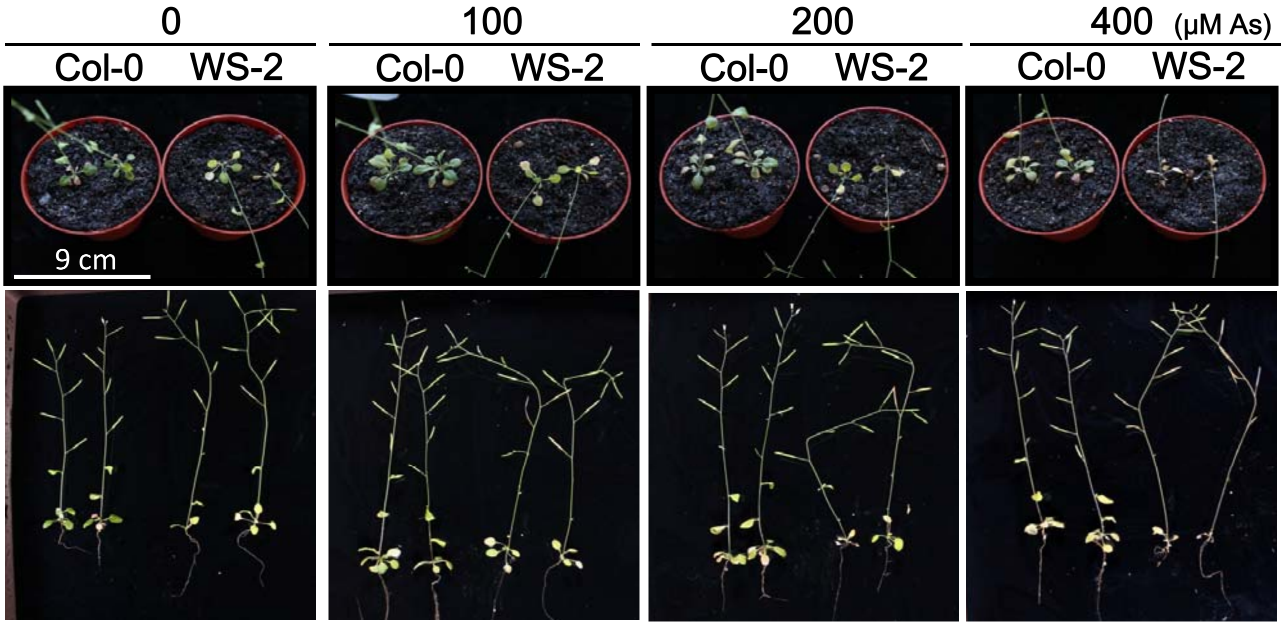

Supplementary Fig. S3 Effects of As on the growth of shoots in 2 *Arabidopsis* ecotypes. *Arabidopsis* plants with different ecotype background (Col-0 and Ws-2) were grown on soil for 4 w. The plants were treated with different concentrations of As (100, 200 and 400 μM) for additional 1 w. The As-treated plants were photographed in comparison with that of control treatment.
