# Supplementary material for: Transcriptome profiling of genes and pathways associated with arsenic toxicity and tolerance in Arabidopsis
Source: BMC Plant Biol. 2014 Apr 16;14:94. doi: 10.1186/1471-2229-14-94 (PMC4021232; doi:10.1186/1471-2229-14-94)
Supplement: Additional file 5: Figure S4 — Exploring Arabidopsis gene expression data with the (a) eFP Browser (http://www.bar.utoronto.ca/) [50] and (b) CAU Bioinformatic Center (http://bioinformatics.cau.edu.cn/cgi-bin/gbrowse/arabidopsis/). [file 1471-2229-14-94-S5.pdf]

(A)

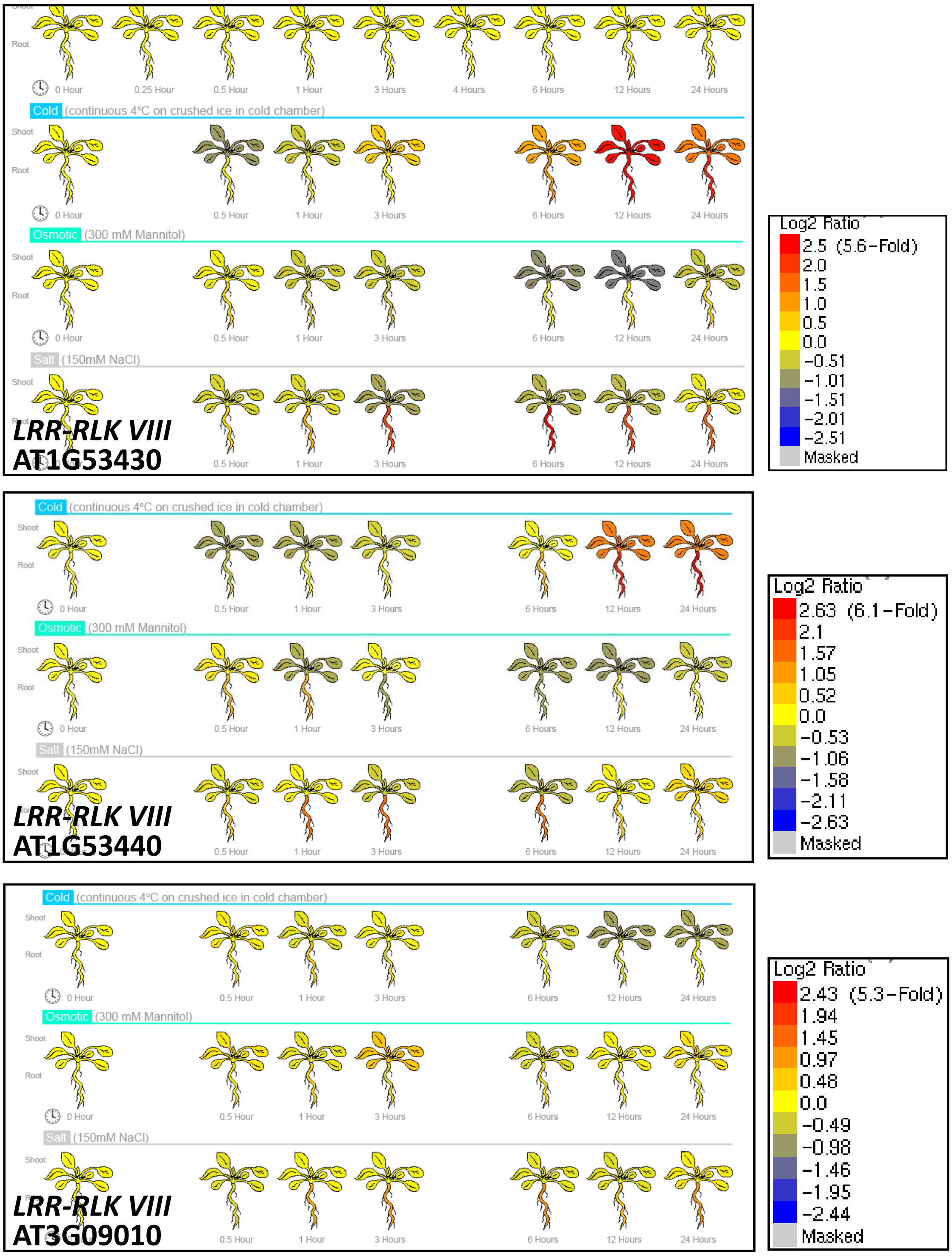


Supplementary Fig. S4 continued

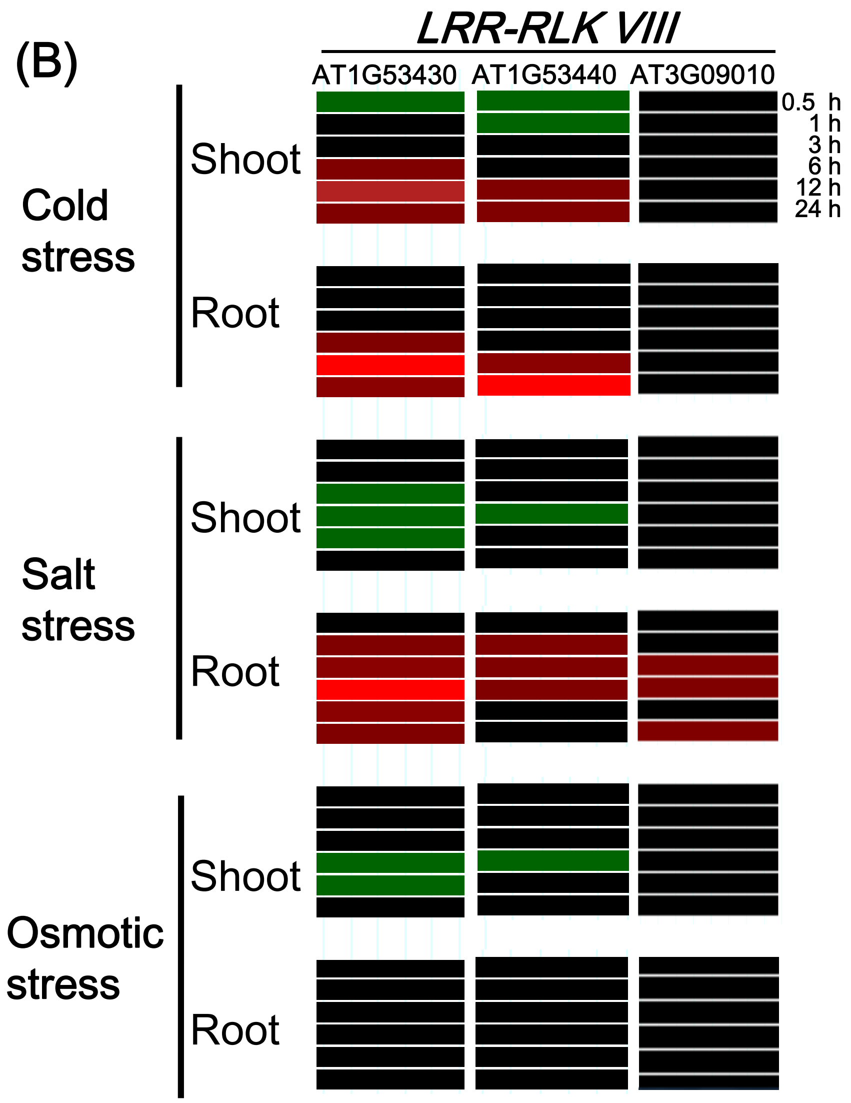


Supplementary Fig. S4 Exploring *Arabidopsis* gene expression data with the (A) eFP Browser (http://www.bar.utoronto.ca/) (Winter et al., 2007) and (B) CAU Bioinformatic Center (http://bioinformatics.cau.edu.cn/cgi-bin/gbrowse/arabidopsis/). Relative expression data for the As-responsive *LRR-RLK VIII* genes are "painted" onto a pictographic representation of the samples that were used to generate the RNA for expression profiling. The relative expression level of these *LRR-RLK VIII* genes is seen to be regulated by abiotic stresses, denoted by green or blue (down) and red (up) coloration in shoots and roots. The abiotic stresses included cold, salt and osmotic stresses. Transcript levels are expressed as a log2-fold change relative to mean expression. The list of 3 *LRR-RLK VIII* genes are as the following: AT1G53430, AT1G53440, ANDAT3G09010.
