# Supplementary material for: Transcriptome profiling of genes and pathways associated with arsenic toxicity and tolerance in Arabidopsis
Source: BMC Plant Biol. 2014 Apr 16;14:94. doi: 10.1186/1471-2229-14-94 (PMC4021232; doi:10.1186/1471-2229-14-94)
Supplement: Additional file 6: Table S9 — List of primers used in this study. [file 1471-2229-14-94-S6.pdf]

Supplementary Table S9 List of primers used in this study

Gene names	AGI number	Primer name	Ssequence (5'--3'))	Product encoded
ERF	AT2G33710	ERF-AT2G33710-F	CGTCGCTTACTCTTCAAGAA	ERF (ethylene response factor) transcription factor family
		ERF-AT2G33710-R1-333bp	GATCAAGAAGTATGTCAGGAGC	
SDG3	AT2G17900	SDG3- AT2G17900-F	GAAGTTACATCGCGATGAATGC	ATSDG37
		SDG3- AT2G17900-R1- 352bp	AATCCCTTGAGGTCTCAATTCG	
LRR-RLK VIII	AT1G53430	LRR-RLK VIII-AT1G53430-F	GTCTGTCGTGTCACAAACATAC	Protein kinase superfamily protein
		LRR-RLK VIII-AT1G53430-R1-352bp	TTAAGGACTCAGGGATTTGACC	
LRR-RLK VIII	AT1G53440	LRR-RLK VIII-AT1G53440-F	GTCTGTCGTGTCACAAACATAC	Protein kinase superfamily protein
		LRR-RLK VIII-AT1G53440-R1-362bp	TTGAGATTGCTCAAGGACTCAG	
LRR-RLK VIII	AT3G09010	LRR-RLK VIII-AT3G09010-F	ATGGCGTTGTCTTTAAGGGAG	Protein kinase superfamily protein
		LRR-RLK VIII-AT3G09010-R1-337bp	ACAACATGAGGTTCGACTTCC	
ATSGP2	AT3G21700	ATSGP2-AT3G21700-F	GAAAAACTAGCTTCCTGGCGA	Monomeric G protein
		ATSGP2-AT3G21700-R1-305bp	CTTGGTTCCTACCATAACTGG	
GTP1	AT1G72660	GTP1-AT1G72660-F	CGCAGAAGAACAAAGCTACAG	Small GTP-binding protein
		GTP1-AT1G72660-R1-320bp	AGCACCTTCAATAATCCCAGG	
UFD1	AT4G15420	UFD1-AT4G15420-F	GAGTTTACTGCTGAAGATGGC	Ubiquitin fusion degradation UFD1 family protein
		UFD1-AT4G15420-R1-326bp	TCAATGTCAGTCTCCAGAACC	
ABC	AT2G47000	ABC-AT2G47000-F	GCAGCTGCTTTTCTCCAATTG	ATP-BINDING CASSETTE B4
		ABC-AT2G47000-R1-654bp	CATGGATCCAGTTAAAACAGCG	
DHAR2	AT1G75270	DHAR2-AT1G75270–F	CGACAAACCCCAATGGTTCTT	DEHYDROASCORBATE REDUCTASE 2
		DHAR2-AT1G75270 –R1- 221bp	ATCCGTCATTAGCGTCTTTGC	
RBL14	AT3G17611	RBL14-AT3G17611-F	CTATAGACTACCGTGGAAACC	RHOMBOID-like protein 14
		RBL14-AT3G17611-R1-451bp	CACACTTGAGTAATCCTCAGC	
LRR-RLK VIII Promoter-GUS	AT1G53440	pRLK8-*Pst* I-F	TGCACTGCAGTGCAGTATTGTTGCAAAAGGGTAG	Protein kinase superfamily protein
		pRLK8-*SpeI* -R	GGACTAGTCCTTCTCTTCTTTTTCTTCTCGG	
LRR-RLK VIII T-DNA line	AT1G53430	AT1G53430- SALK_129312-LP	GGGAGTTGCAATTATGCTAGAAG	Protein kinase superfamily protein
		AT1G53430- SALK_129312-RP	ATTGGGCATATGTCTTGCAAG	
		T-DNA left-border primer (LB)	GCGTGGACCGCTTGCTGCAACT	
LRR-RLK VIII T-DNA line	AT1G53440	AT1G53440- SALK_148231-LP	TTAGAAAGCAGTCGACGAACC	Protein kinase superfamily protein
		AT1G53440- SALK_148231-RP	AAAGATCGTTCGCAATGTTTG	
		T-DNA left-border primer (LB)	GCGTGGACCGCTTGCTGCAACT	
LRR-RLK VIII T-DNA line	AT1G53440	AT1G53440- SALK_057812-LP	ATTTACCGCTTCTTCTACCGG	Protein kinase superfamily protein
		AT1G53440- SALK_057812-RP	TGTTTTATTTGCGGGACTTTG	
		T-DNA left-border primer (LB)	GCGTGGACCGCTTGCTGCAACT	
